# From Potential Prebiotic Synthons to Useful Chiral Scaffolds: A Synthetic and Structural Reinvestigation of 2-Amino-Aldononitriles

**DOI:** 10.3390/molecules29081796

**Published:** 2024-04-15

**Authors:** Esther Matamoros, Mark E. Light, Pedro Cintas, Juan C. Palacios

**Affiliations:** 1 Departamento de Química Orgánica e Inorgánica, Facultad de Ciencias, and Instituto del Agua, Cambio Climático y Sostenibilidad (IACYS)-Unidad de Química Verde y Desarrollo Sostenible, Universidad de Extremadura, 06006 Badajoz, Spain; pecintas@unex.es; 2 Departamento de Química Orgánica, Universidad de Málaga, Campus Teatinos s/n, 29071 Málaga, Spain; 3 Instituto de Investigación Biomédica de Málaga y Plataforma en Nanomedicina—IBIMA, Plataforma Bionand, Parque Tecnológico de Andalucía, 29590 Málaga, Spain; 4 Department of Chemistry, Faculty of Engineering and Physical Sciences, University of Southampton, Southampton SO17 1BJ, UK; light@soton.ac.uk

**Keywords:** aldononitrile, aminosugars, chirality, spectroscopic characterization, X-ray crystallography

## Abstract

This paper explores and revisits in detail the formation and characterization of sugar-based aminonitriles, whose ultimate origin can be traced to the interaction of biomolecules with cyanide. Although the synthesis and spectroscopic data of 2-amino-aldononitriles were reported long ago, there are both contradictory and confusing results among the published data. We have now addressed this concern through an exhaustive structural elucidation of acylated 2-amino- and 2-alkyl(aryl)amino-2-deoxyaldonitriles using mass spectrometry and FT-IR, FT–Raman, and NMR spectroscopies. Several structures could be unambiguously determined through single-crystal X-ray diffraction, which allowed us to correct other misassignments. Moreover, this study unveils how steric and electronic effects influence the acylation outcome of the amino, (alkyl, aryl)amino, or acetamido group at C-2. The chirality at the latter, which was assigned tentatively through optical rotation correlation, and hence the preferential threo stereochemistry generated during the cyanohydrin synthesis of 2-amino-2-deoxy aldononitriles have now been established with confidence.

## 1. Introduction

α-Aminonitriles are important intermediates in the synthesis of α-amino acids, α-aminoamides, thiadiazoles, and imidazole derivatives [1]. The classical Strecker synthesis and Strecker-type reactions represent the most general methods of preparing α-aminonitriles [2,3,4,5], in which an aldehyde or ketone is reacted with an amine and hydrocyanic acid or a chemical equivalent, such as potassium cyanide in acid media or trimethylsilyl cyanide [6,7,8,9,10,11,12,13]. In a prebiotic context, α-aminonitriles can also be formed via electric discharge through a mixture of anhydrous methane and ammonia and hydrolyzed to amino acids, thereby presenting a credible hypothesis within the repertoire of chemical evolution [14,15]. Recent studies on prebiotic chemistry point to a cyanosulfidic scenario and disclosing a chemoselective, high-yielding α-aminonitrile ligation that leads to α-peptides under aqueous conditions [16]. α-Aminonitriles can be acetylated in water by thioacetate anions and this acylation activates the nitrile group of the newly formed α-acetamidonitrile just enough to promote a nucleophilic attack by H_2_S, not by water. The resulting thioacid then links to the α-aminonitriles to afford the corresponding peptides. Accordingly, short *N*-acyl peptide nitriles would have been plausible substrates during early evolution by interconnecting prebiotic α-aminonitrile synthesis with the formation of biological α-peptides.

**Scheme 1:**
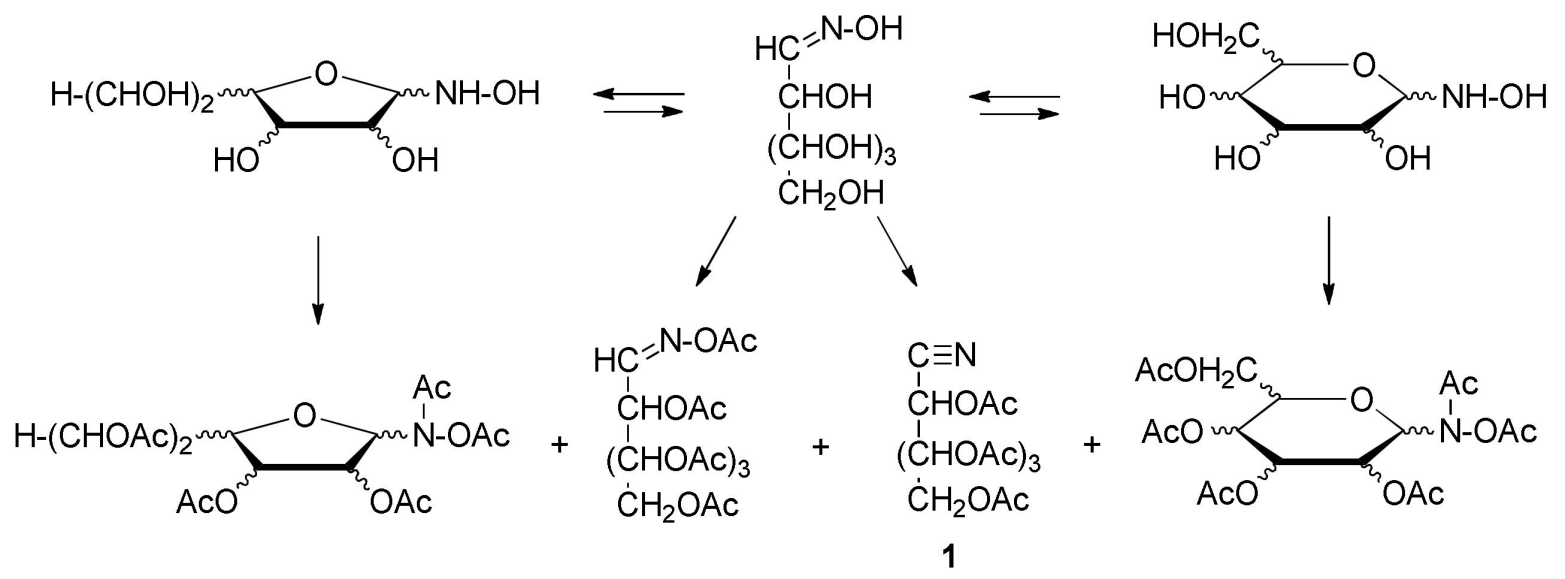
Landscape of acyclic and cyclic structures generated from aldoximes under acetylating conditions.

On the other hand, peracylated aldononitriles (**1**) have been extensively studied with gas chromatography coupled to mass spectrometry [17,18,19,20,21,22,23,24,25,26] and used in various quantitative analyses of sugar and aminosugar mixtures [27,28,29,30,31,32,33,34,35,36,37,38]. Derivatization usually involves a base-catalyzed acetylation of the corresponding aldose oxime with acetic anhydride. The use of such derivatives provides some advantages over other sugar derivatives, mainly because it was believed that a single compound preserving the overall stereochemistry of the starting sugar is produced. However, the formation of significant amounts of peracetylated acyclic and cyclic oximes, both in furanoid and pyranoid structures, has raised questions regarding the quality of the method (Scheme 1) [39].

Despite the structural uncertainty, however, the procedure proves to be advantageous in biological analyses. Thus, for example, whole-cell hydrolysates of *Bacteroides fragilis*, of the genus *Bacteroides* Castellani and Chalmers 1919, and closely related genetical species *B. vulgatus*, *B. ovatus*, *B. eggerthii*, *B. distasonis*, *B. uniformis*, *B. thetaiotaomicron*, *B. stercoris*, *B. merdae*, and *B. caccae* were employed to determine, on the basis of chemical derivatization, characteristic carbohydrate patterns using capillary gas chromatography. Seven typical peaks for peracetylated aldononitriles could be selected from carbohydrate fingerprints of the reference strains to prepare a dichotomous identification key. Therefore, the classification of an unknown strain of *Bacteroides fragilis* group can be accomplished and requires only 4–5 h after primary isolation. As a result, other sophisticated techniques under anaerobic conditions can be avoided [40].

**Scheme 2:**
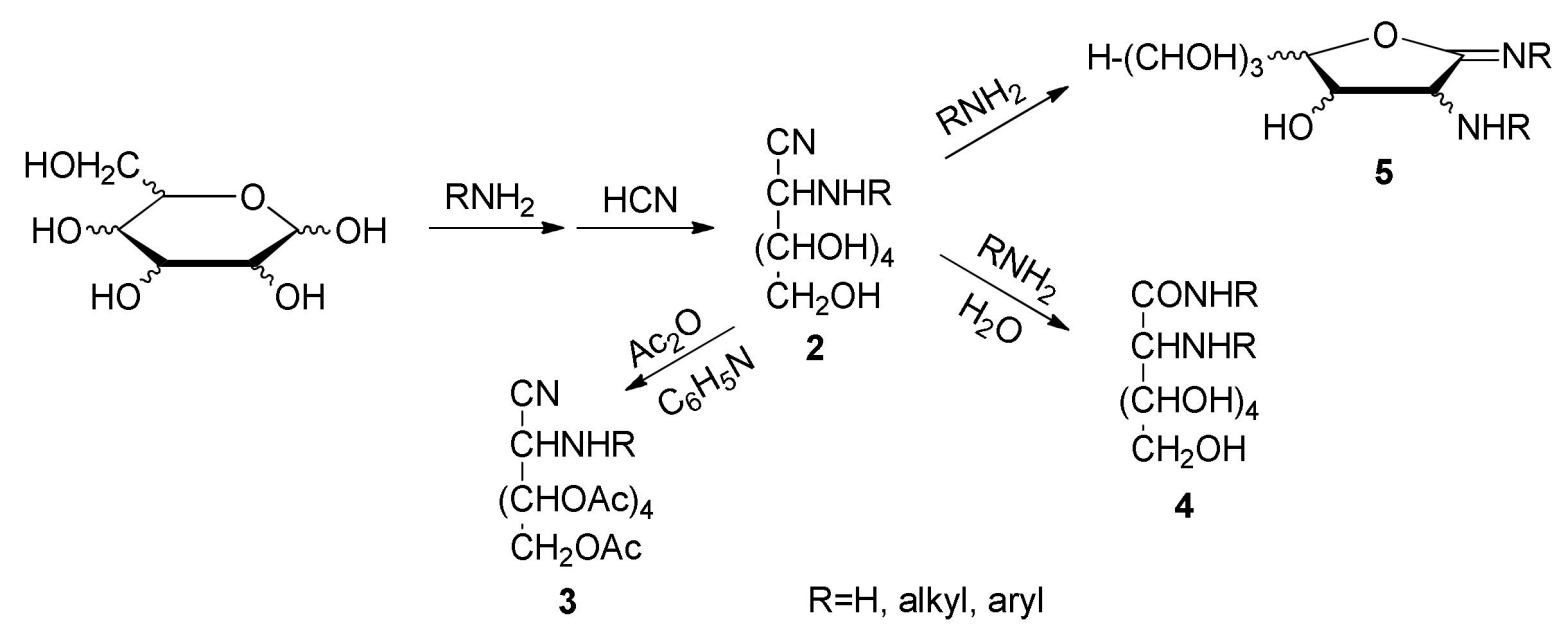
Compound set generated in the cyanohydrin synthesis and late derivatization of aminonitriles.

Also, the dehydration of per-*O*-acetylaldonamides leads to per-*O*-acetylaldononitriles [41], just like the radical fragmentation of sugar-derived β-hydroxy azides [42]. Conformational studies aided by molecular mechanics were conducted on peracylated aldononitriles, with the ultimate goal of performing a full stereochemistry elucidation of the aldose moiety starting from NMR spectroscopy [43,44,45]. In contrast, few data are available for 2-amino-2-desoxyaldononitriles (**2**) and their acyl derivatives (**3**). More recently, mass spectrometry of amino sugar aldononitrile acetate derivatives was used for isotope enrichment assessment of microbial residues in soil [35]. 2-Amino-2-deoxyaldononitriles and their *N*-alkyl and *N*-aryl derivatives are intermediates in the aminonitrile synthesis of 2-amino and 2-alkylamino-2-deoxyaldoses [46,47,48,49,50,51,52,53,54,55,56], and they are generally obtained by reacting an aldose with excess of ammonia or an amine in anhydrous methanol/ethanol with subsequent addition of anhydrous hydrogen cyanide. However, unexpected by-products like aminoamides **4** and iminolactones **5** are sometimes obtained (Scheme 2) [57,58,59,60].

Peracyl-2-amino-2-deoxyaldononitriles can be generated through the acylation of 2-amino-2-deoxyaldononitriles [49,50,51,52,53,54,55,56] and oximes of 2-amino-2-deoxyaldosas [61]. Such substances have been used for characterizing 2-amino-2-deoxyaldoses and their *N*-alkyl and *N*-aryl derivatives, as well as synthons in the preparation of 1,2-diamino-1,2-dideoxyalditols [62], which are additional starting materials for the synthesis of polyhydroxyalkyl heterocycles. The penta-acetyl derivatives of 2-deoxy-2-methylamino-L-glucononitrile (**6**) and its l-*mano* epimer (**7**) were obtained through conventional acetylation with acetic anhydride and pyridine [63,64]. Similar routes have been applied to the enantiomeric pair of penta-acetyl derivatives **8** and **9** and tetra-acetate **10** [65,66]. Likewise, a wide variety of acetyl (**8**, **11**–**17**) and benzoyl derivatives (**18**–**25**) have been reported, including the isolation of carbobenzoxyl aminonitrile **26** without hydroxyl protection [52]. 

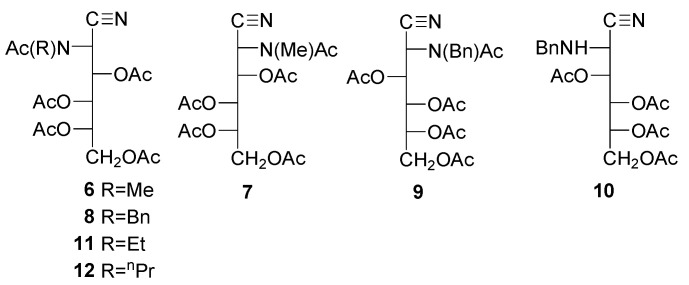

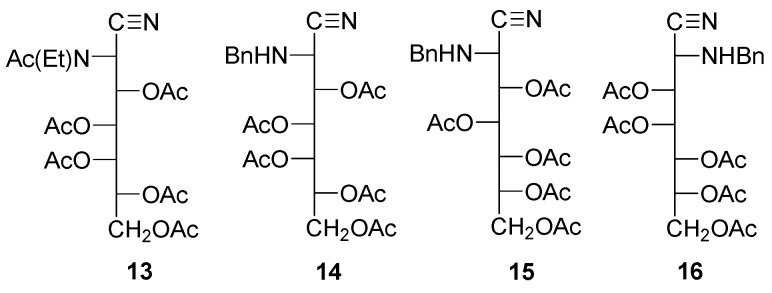





In general, however, spectral characterization is poor. Furthermore, data in the literature often indicate contradictory results. Thus, it has been reported that 2-amino-2-desoxialdononitriles bearing *N*-ethyl or *N*-propyl groups are acylated at the nitrogen atom and do not suffer acylation if the group is *N*-isopropyl or *N*-phenyl. For *N*-benzyl derivatives, the group can sometimes be acetylated (**8**,**9**) [65,66] or cannot (**10**, **14**–**16**) [49,66].

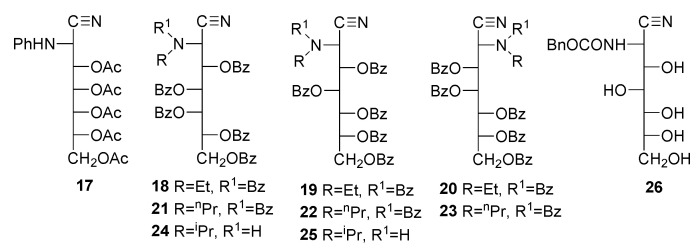



In order to clarify these and other structural issues, the present work focuses on a series of representative sugar aminonitriles along with their acetyl and benzoyl derivatives. An extensive and compelling structural analysis has been performed, where single-crystal X-ray diffraction proved to be instrumental for determining the acetylation site and the stereochemical outcome. 

## 2. Results and Discussion

### 2.1. Synthesis of Acylated Derivatives 

The preparation of aminonitriles **27**–**36** was performed as described previously [49,50,51,52,53,54,55,56] using the classical cyanohydrin synthesis, i.e., the addition of liquid hydrogen cyanide to a glycosylamine formed through reaction of the corresponding monosaccharide with an excess of ammonia, alkyl, or aryl amines. In general, isolation of the glycosylamine derivative, rather than the one-pot protocol, leads to a clean and high-yielding synthesis. As mentioned above, this strategy avoids the formation of by-products like aminoamide **4** or iminolactone **5** [57]. The problem is associated primarily with the use of alkyl amines, while cleaner reactions occur when using aniline, probably due to its lower nucleophilicity [56].

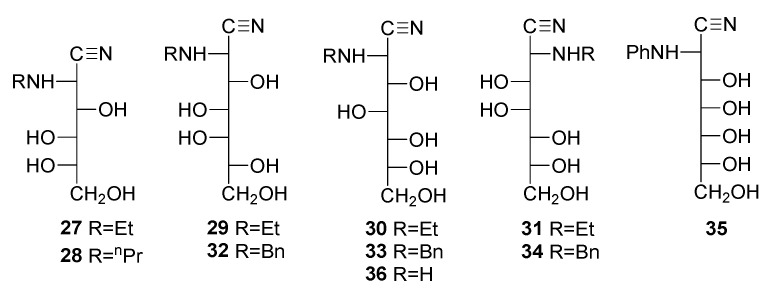



Acylation of the resulting aldonoaminonitriles was achieved using standard methods, namely the addition of acetic anhydride or benzoyl chloride to a solution/suspension of the aminonitrile in pyridine. Thus, peracetyl derivatives **11**–**13** could easily be obtained from **27**–**29**, and perbenzoylated **18**–**20** from **29**–**31**, respectively [49,50,51,52,53,54,55,56]. In contrast, the acetylation of **32** and **33** did not afford the described penta-acetyl derivatives **14** and **15** [49], and peracetyl 2-benzylamino-2-deoxyheptononitriles having d-*glycero*-l-*gluco* **37** and d-*glycero*-d-*ido* **38** configurations were obtained instead. Accordingly, the structure of the acetylated 2-benzylamino-2-deoxy-d-*glycero*-d-*galacto*-heptononitrile should be consistent with **39** rather than **16**. The same reasoning applies to tetraacetate **10**, which should be a penta-acetylated derivative.

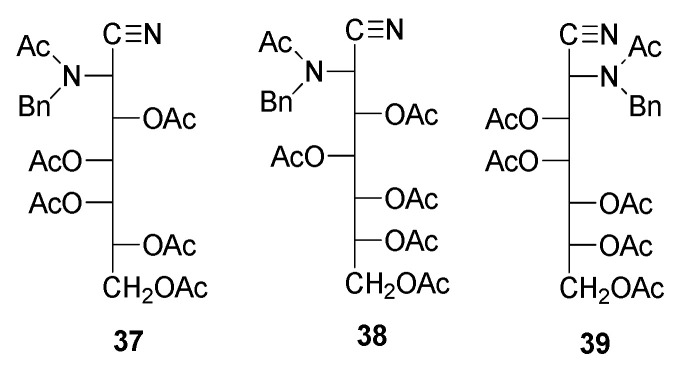



**Scheme 3:**
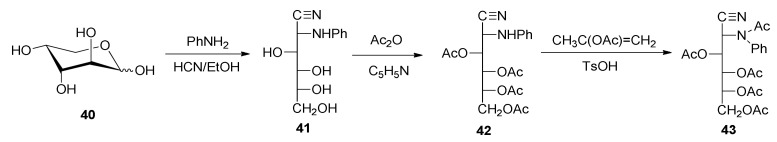
Cyanohydrin synthesis from d-arabinose and aniline followed by acetylation reactions.

Additionally, in contrast, it has been reported that acetylation of 2-deoxy-2-phenylamino-d-*allo*-heptononitrile (**35**) produces the penta-*O*-acetyl derivative **17**. In order to confirm this assessment, we have prepared 2-deoxy-2-phenylamino-d-glucononitrile (**41**) through cyanohydrin synthesis from d-arabinose (**40**) and aniline (Scheme 3). Its conventional acetylation leads to tetraacetate **42** in a quantitative yield.

**Scheme 4:**
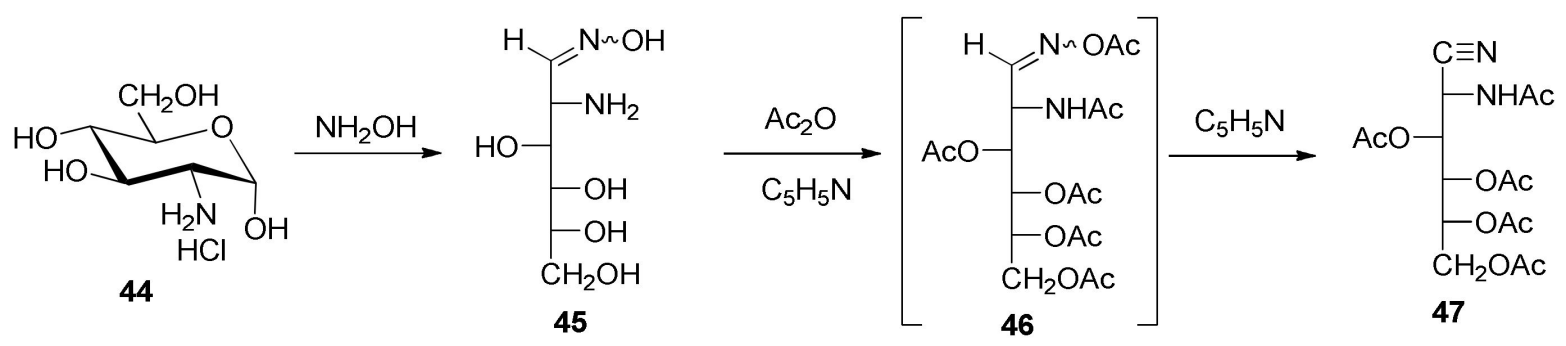
Synthesis of aminonitrile model **47** from oxime **45**.

However, the phenylamino group could be acetylated by heating **42** at reflux in isopropenyl acetate and using *p*-toluenesulfonic acid as catalyst, thereby obtaining 3,4,5,6-tetra-*O*-acetyl-2-(*N*-phenylacetamido)-2-deoxy-d-glucononitrile (**43**) in a high yield (82%). As a model compound, tetra-*O*-acetyl-2-acetamido-2-deoxy-d-glucononitrile (**47**) was also prepared through the dehydration under acetylating conditions of 2-amino-2-deoxy-d-glucose oxime (**45**), using a modification of the Restelli-Deulofeu method [61] (Scheme 4).

**Scheme 5:**
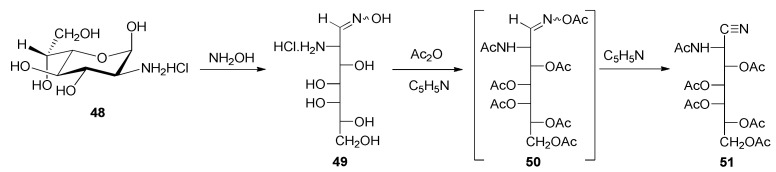
Synthetic strategy for producing peracetylated aminonitrile **51**.

Analogously, penta-*O*-acetyl-2-acetamido-2-deoxy-d-*glycero*-l-*gluco*-heptononitrile (**51**) was generated through an oxime (**49**), which derives from 2-amino-2-deoxy-α-d-*glycero*-l-*gluco*-heptopyranose hydrochloride (**48**) [67] as a starting material (Scheme 5). It is worth pointing out that the amide character of the nitrogen atom at C-2 in both **47** and **51** prevents further acylation, at least under the conditions tested.

### 2.2. Structural Elucidation 

All of the proposed structures are in agreement with their elemental analyses and spectral data, including FT-IR, FT–Raman, ^1^H and ^13^C NMR, and high-resolution mass spectra (HRMS), as well as X-ray diffractometry. 

The oft-invoked diagnostic IR absorption of the cyano group has little value due to the extremely weak or even absent C≡N stretching vibrations [68]. It is well established that substitution of the alkyl group in alkyl nitriles by oxygen-containing functional groups results in a decrease in the intensity of the C≡N stretching band. Moreover, this effect can be enhanced further by increasing the number of such substituents and by attaching them to the carbon atom that bears the C≡N group [69,70]. In agreement with these observations, *O*-protected cyano carbohydrate derivatives do not generally exhibit the typical C≡N stretching vibration or it is extremely weak [68,71,72,73,74,75,76,77,78,79]. Also, α-acetamidoalkyl nitriles, such as *N*-(cyanomethyl)acetamide or ethyl acetamidocyanacetate, show weak or negligible C≡N stretching absorption bands [70]. Unlike IR spectroscopy, Raman spectra of substituted sugar cyanides exhibit strong C≡N stretching bands; however, this technique has not yet been applied to aldonoaminonitriles and their derivatives [80].

Since IR spectra of peracetylated α-aminonitriles do not provide clear-cut indications of the C≡N group in 2-amino-aldonitriles, its presence can be inferred from Raman (ṽ_C≡N_ ~2220 cm^−1^) or ^13^C NMR spectra (δ_C≡N_ ~115 ppm). Thus, for example, the signal at ~119 ppm demonstrates unequivocally the survival of the cyano group during the synthesis of **26**. Tables S1–S3 collect a series of diagnostic signals, either stretching absorption bands or chemical shifts, for all of the compounds studied. 

### 2.3. Peracetyl Alkylaminonitriles 

**Figure 1:**
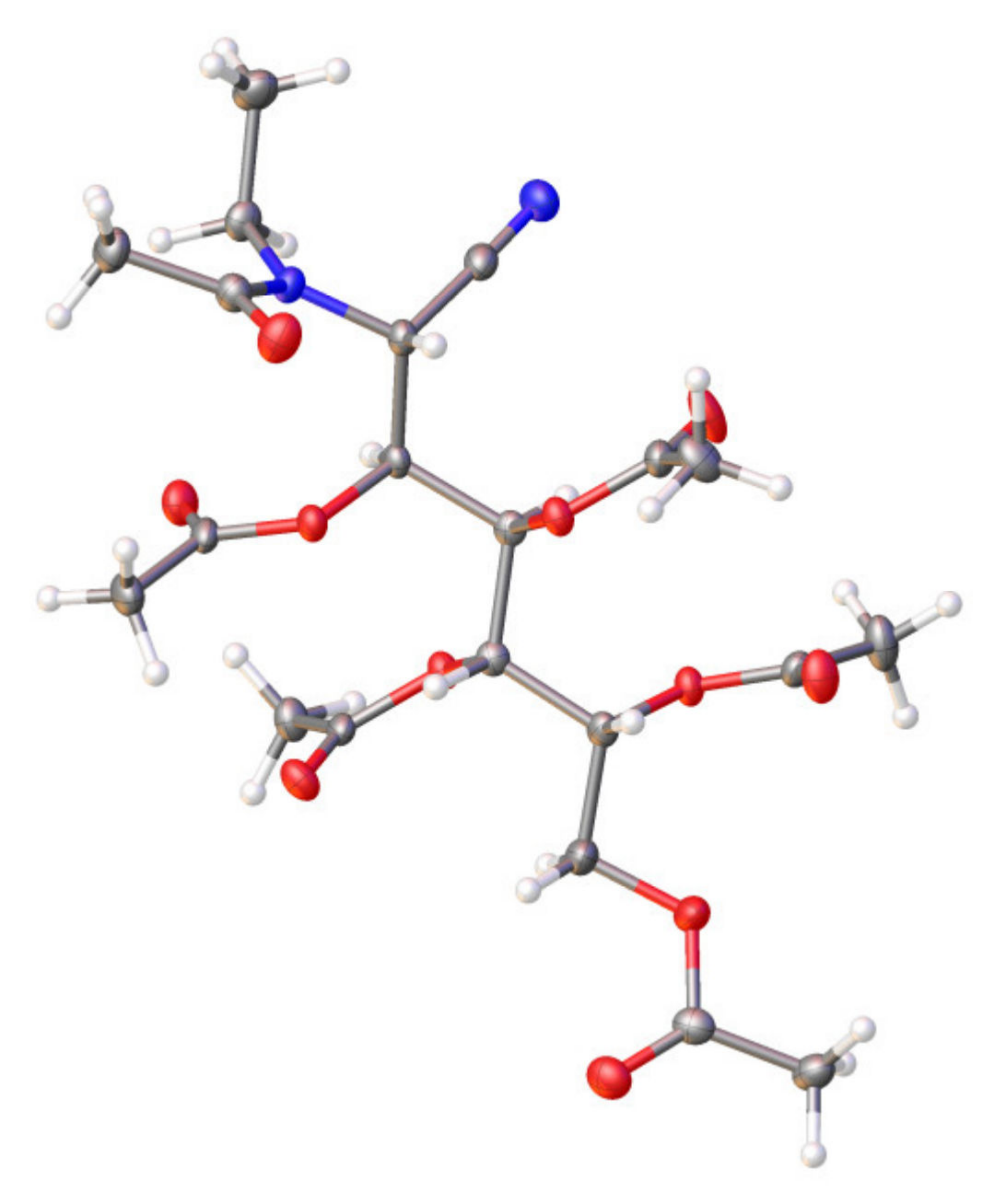
X-ray diffraction structure of **13** recorded at 120 K. Thermal ellipsoids are drawn at 35% probability.

The determination of the molecular masses of **11** [52] and **13** [50] was performed by recording their HRMS (CI mode), which showed [M + H]^+^ and [M + NH_4_]^+^ peaks. Values of *m*/*z* = 415.1706 for **11** (C_18_H_26_N_2_O_9_, MW 414.1638) and *m*/*z* = 487.1904 (C_21_H_30_N_2_O_11_, MW 486.1850) for **13** were obtained. The spectroscopic data of compounds **11** and **13** are closely similar. Their IR spectra show typical absorptions of acetate and tertiary amide at ~1750 cm^−1^ and ~1650 cm^−1^, respectively [69]. The absorption bands of the C≡N group are very weak, but they are moderately intense in the corresponding Raman spectra. Signal integration and carbon counting in the ^1^H and ^13^C NMR spectra show the presence of five acetyl groups for **11** and **12**, and six for **13** (four or five acetate groups and one acetamido group, respectively). The presence of a signal at ~115 ppm shows the presence of the C≡N group [81,82]. Finally, the structure of **13** could conclusively be demonstrated using single-crystal X-ray diffraction (Figure 1).

Along with the unequivocal constitution of the acylated aminonitriles, a second objective of this study involved confirming the stereochemistry at C-2 assigned to the parent aminonitriles. In previous work, the configuration at C-2 was tentatively based on the sign of optical rotations, in agreement with empirical rules for other acyclic compounds, such as monosaccharide nitriles, amides, hydrazides, and (alditol-1-yl)-heterocycles, although some exceptions have been found [65,78,83,84,85,86,87,88,89,90,91,92,93,94,95,96,97]. Such rules establish a direct correlation between the sign and the absolute configuration at C-2, being positive for d-configured and negative for l-configured aminonitriles. Data from the literature evidence the general trend of such a correlation for sugar 2-aminonitriles [49,50,51,52,53,54,55,56,58,65,66,89,90,91,92,93,94,95,96,97,98]. The structure determined with X-ray diffraction for **13** shows that the stereochemistry at C-2 is *R* (Figure 1), thus confirming the stereochemistry at C-2 of the starting aminonitrile **29**, whose configuration d-*glycero*-l-*gluco* has previously been assigned [50]. 

On the other hand, the stereochemistry of the amide and acetate groups in the solid state is *Z-anti*. In Table 1, some experimental parameters (crystal data) for the unit cell of **13** are collected and compared with those calculated in a vacuum and in chloroform (for theoretical calculations, see the next section). They have also been calculated in the solid state using the experimental data extracted from the crystallographic analyses.

**Scheme 6:**
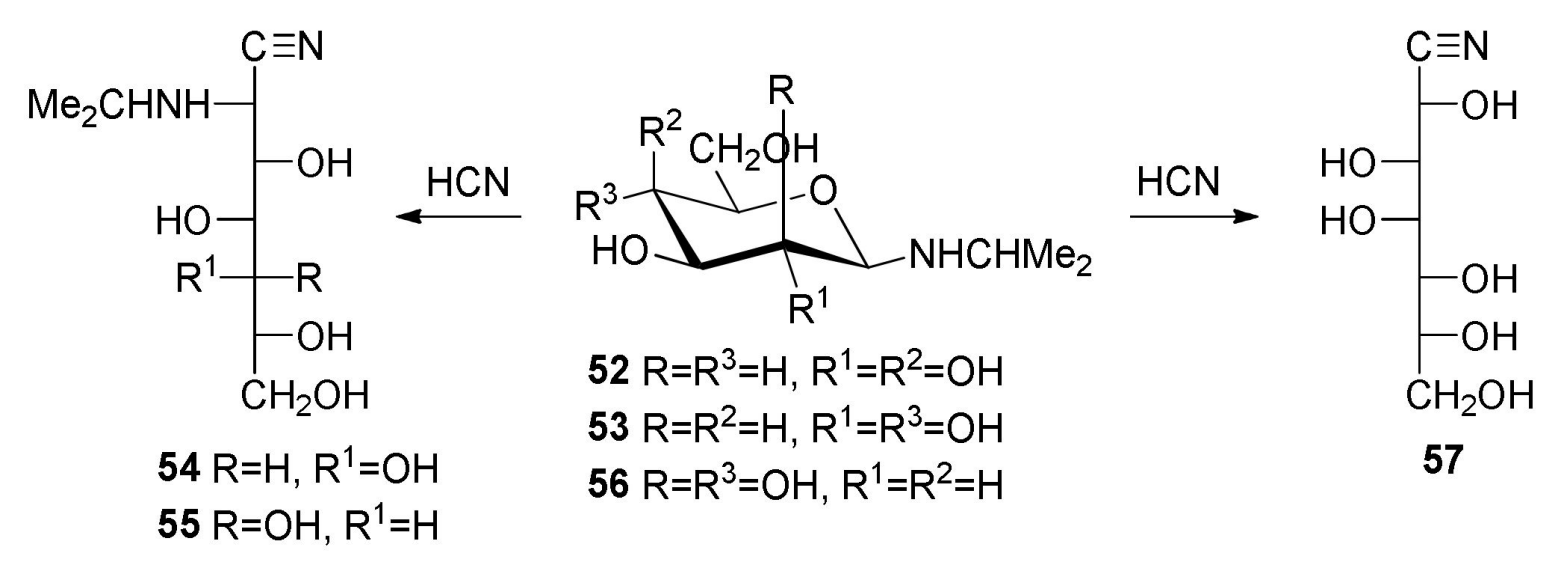
Cyanohydrin reactions of glycosylamines **52**, **53**, and **56**.

The spectroscopic data of **12**, bearing an *N*-*n*-propyl group, are similar to those of **11** and **13**, and therefore consistent with acylation at the nitrogen atom. However, when the alkyl group is isopropyl, the nitrogen atom is not acylated (**24**, **25**), probably due to steric hindrance [50]. This effect manifests itself even in the synthesis of *N*-isopropyl aminonitriles. Thus, the treatment of a solution of isopropylamine and d-galactose or d-glucose (or a solution of the previously isolated *N*-isopropyl glycosylamine, **52** or **53**) with anhydrous hydrogen cyanide leads to the corresponding nitriles (**54**, **55**) (Scheme 6) [50]. Their conventional benzoylation leads to **24** and **25**, as previously described [50], whereas the corresponding acetylation produces the penta-*O*-acetyl heptononitriles **58** and **59**. Because of the fact that the aminonitrile **55** obtained was impurified by its epimer (24%) with a d-*glycero*-d-*gulo* configuration (**60**), compound **59** was likewise obtained accompanied by its epimer **61** (21%). Although they do not show the characteristic absorption of the C≡N group in their IR spectra, the nitrile functionality agrees with the signal at ~118 ppm in the ^13^C NMR spectra.

The absence of acetylation at the nitrogen atom of **58** and **59** is supported by (a) the mass spectra showing the signal [M + H]^+^ at *m*/*z* 459 (C_20_H_30_N_2_O_10_) and not at *m*/*z* 501 (C_22_H_32_N_2_O_11_); (b) the presence of a weak signal at 3330 cm^−1^, corresponding to the NH stretching vibration, together with the absence of the amide band at ~1640 cm^−1^; (c) the proton and carbon signals in the NMR spectra indicating the presence of only five acetyl groups; and (d) the upfield chemical shift of H-2 with respect to the analogous proton of **13** (Δδ ~ 1.4 ppm).

However, when using isopropylamine and d-mannose as the starting aldoses, instead of aminonitrile **62**, d-*glycero*-d-*galacto*-heptononitrile (**57**) was obtained in a low yield (17%). The latter increased up to 45% when starting from the previously isolated *N*-isopropyl-β-d-mannopyranosylamine (**56**). The resulting nitrile **57** shows the C≡N bond stretching absorption at ~2220 cm^−1^. Acetylation and benzoylation reactions afforded the corresponding peracetyl (**63**) and perbenzoyl (**64**) derivatives, neither showing a characteristic band that can be ascribed to the stretching vibration of the NH bond at ~3300 cm^−1^ or the first amide band at ~1650 cm^−1^, irrespective of spectra recorded in the solid state or chloroform solution.

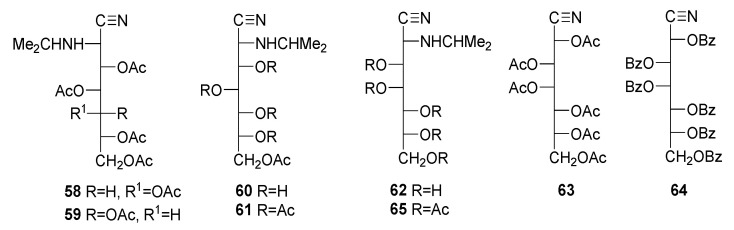



**Figure 2:**
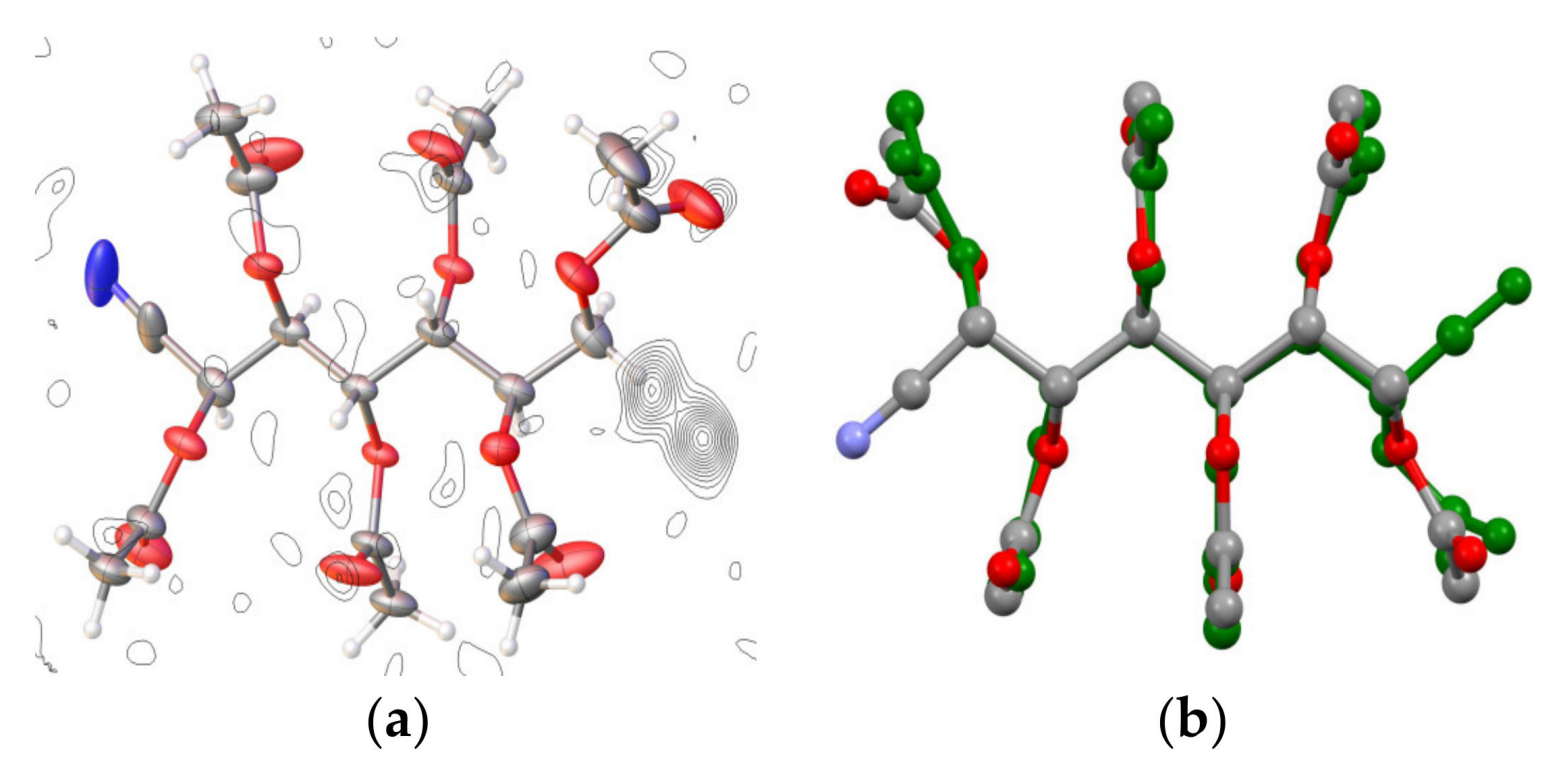
(**a**) Single-crystal X-ray diffraction of **63** at 100 K. Thermal ellipsoids are drawn at 50% probability. The residual electron density map clearly shows the existence of a disordered nitrile position. (**b**) Overlay of a rotated molecule (green) with the original, showing how they fit into a very similar volume of space.

The mass spectrum of the acetyl derivative shows a base peak at *m*/*z* 482.13, which could correspond to either the [M + H + Na]^+^ of **63** or [M + Na]^+^ of **65**. In addition, ^1^H and ^13^C NMR spectra show the absence of the isopropyl group, and the proton count indicates the presence of six acetate groups. The number of carbon atoms attributable to the alkyl chain is seven, including the cyano group. Accordingly, nitrile functionality arises from the addition of HCN to d-mannose, leading to **57**, whereas the *O*-protected acyl derivatives are **63** and **64**, respectively. Such products have also been reported in the literature [99], and the stereochemistry at C-2 is consistent with the positive value of their optical rotations [49,50,51,52,53,54,55,56,65,83,84,85,86,87,88,89,90,91,92,93,94,95,96,97]. Finally, the structures assigned to nitrile **57** and its acyl derivatives were supported by the unambiguous structure of **63** determined with X-ray diffraction. This result indicates that the configuration of the new chiral center at C-2 is *R* and confirms the stereochemistry results previously assigned to **57**, **63**, and **64**. It is noteworthy that the conformation of the molecule is such that it can easily rotate 180° and still fit into the same average packing arrangement and a very similar volume of space (see Supplementary Materials). In the X-ray structure determination, this is seen as a disordered average structure that was modeled with the nitrile group partially occupied at either end of the chain. Figure 2 clearly shows the residual electron density peaks originating from the minor orientation of the molecule. The occupancy of the nitrile was refined to a 30/70 split (and fixed in the final cycles at this value)—meaning that 30% of the time, the molecule packs in the opposite orientation. It was not necessary to employ any geometrical or thermal parameter restraints.

### 2.4. Peracetyl Benzylaminonitriles

Remarkably, the structures assigned to **14**–**16** and to the acetyl derivatives of **32**–**34** [49] cannot be distinguished from **37**–**39** through their elemental analyses, being essentially almost identical (C_24_H_30_N_2_O_10_:C, 56.90; H, 5.97; N, 5.53 versus C_26_H_32_N_2_O_11_:C, 56.93; H, 5.88; N, 5.11). This is probably one of the reasons accounting for the erroneous assignment of structures **14**–**16**.

The steric, electronic, and conformational parameters [100,101,102,103] of different substituents carried by the nitrogen atom at C-2 are collected in Table 2. The A parameter for substituent X is given by A_x_= −ΔG˚_x_, where ΔG˚x measures the variation of the free energy in the axial ⇄ equatorial equilibrium for cyclohexane carrying the X substituent. On the basis of such parameters, no clear-cut reason justifies the absence of acetylation at the nitrogen atom in compounds **32**–**34**. The steric parameters for the benzyl group are similar to those of the *n*-propyl group, and there should be no steric hindrance to nitrogen acetylation. Moreover, the value of σ* does not explain this behavior, because even though the benzyl group is electron-withdrawing, σ* = 0.22 is lower than that of hydrogen (σ* = 0.49), which enables acetylation.

To distinguish between the two types of structures, we performed a determination of the molecular mass of **37** (548.2006 for C_26_H_32_N_2_O_11_) using HRMS (CI mode). Peaks of [M + H]^+^ (*m*/*z* 549.2069) and [M + NH_4_]^+^ (*m*/*z* 566.2330) were detected, thus confirming the presence of the acetamido group. In the FT-IR spectra of **37** and **38**, two carbonyl signals are observed for the acetate and amide groups at ~1750 cm^−1^ and ~1670 cm^−1^, respectively. The absorption of the C≡N group is almost nonexistent and even the Raman absorption, appearing at ~2250 cm^−1^, is weak. The ^1^H and ^13^C-NMR spectra confirm the amide structure of **37** due to, on one hand, the absence of a signal attributable to the NH group, and on the other, the integration of the acetamido group, together with signals showing the presence of five acetate groups. The methylene protons of the benzyl group are diastereotopic and appear as doublets, with a chemical shift difference of 0.23 ppm. 

**Figure 3:**
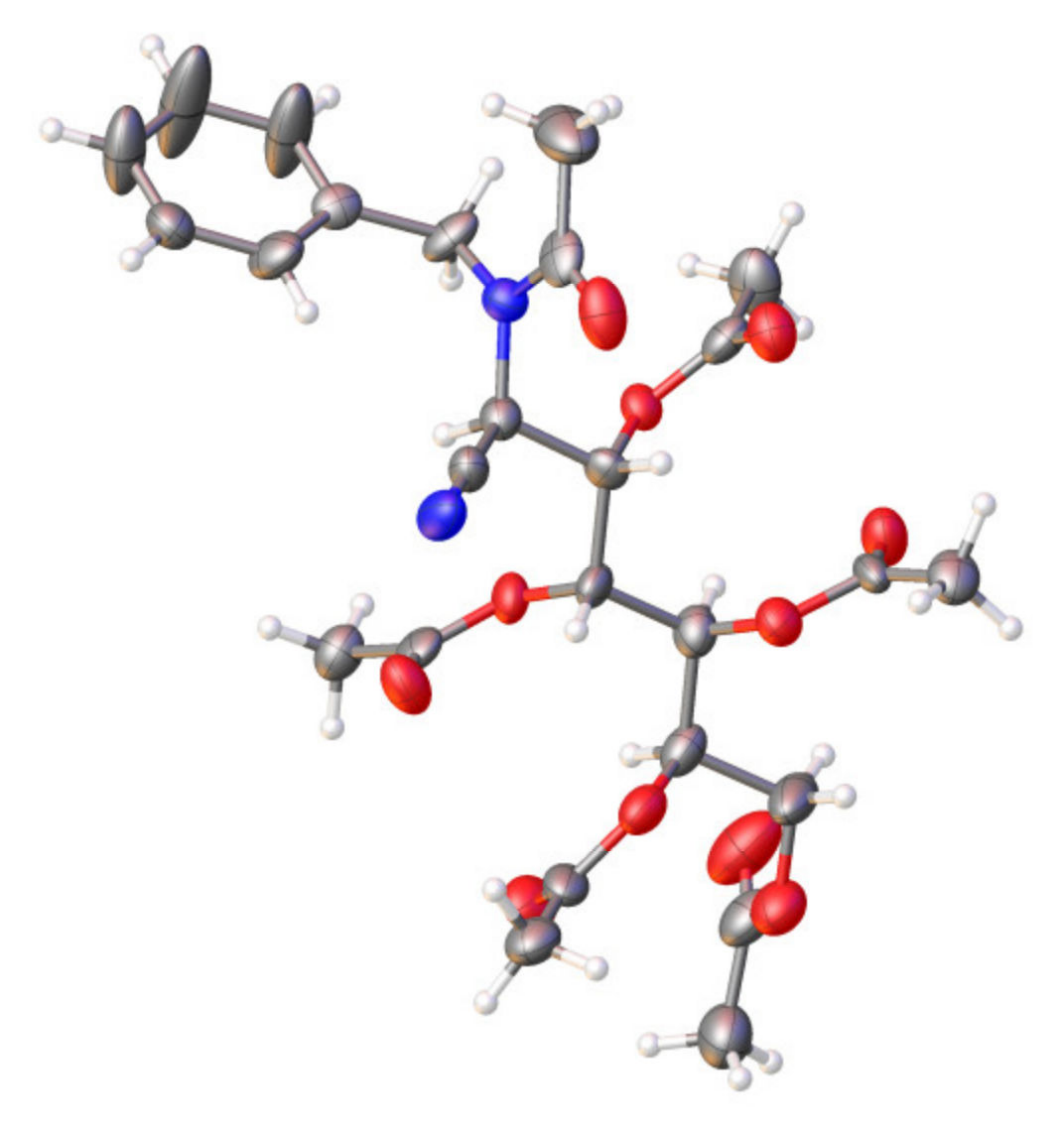
X-ray diffraction structure of **37** at 120 K. Thermal ellipsoids are drawn at 35% probability.

The structure of **37** determined with X-ray diffraction at 120 K is shown in Figure 3, and confirms definitively the acetylation at the nitrogen atom. In addition, the configuration at C-2 is *R*, which confirms in turn the stereochemistry at C-2 of the starting aminonitrile **32**, an assignment made based on the negative sign of optical rotation [49]. The stereochemistry of the amide group is *Z*-*syn-anti* (see Scheme 6): that is, in the conformation adopted in the unit cell, the C=O amide group is not arranged parallel to the C2-H bond, as it is in the case of **13**, but instead adopts an antiparallel arrangement.

Table 3 shows some experimental and calculated geometric parameters of **37**, both in a vacuum and in chloroform, as well as in the solid state using the experimental data from the single-crystal X-ray analyses.

**Figure 4:**
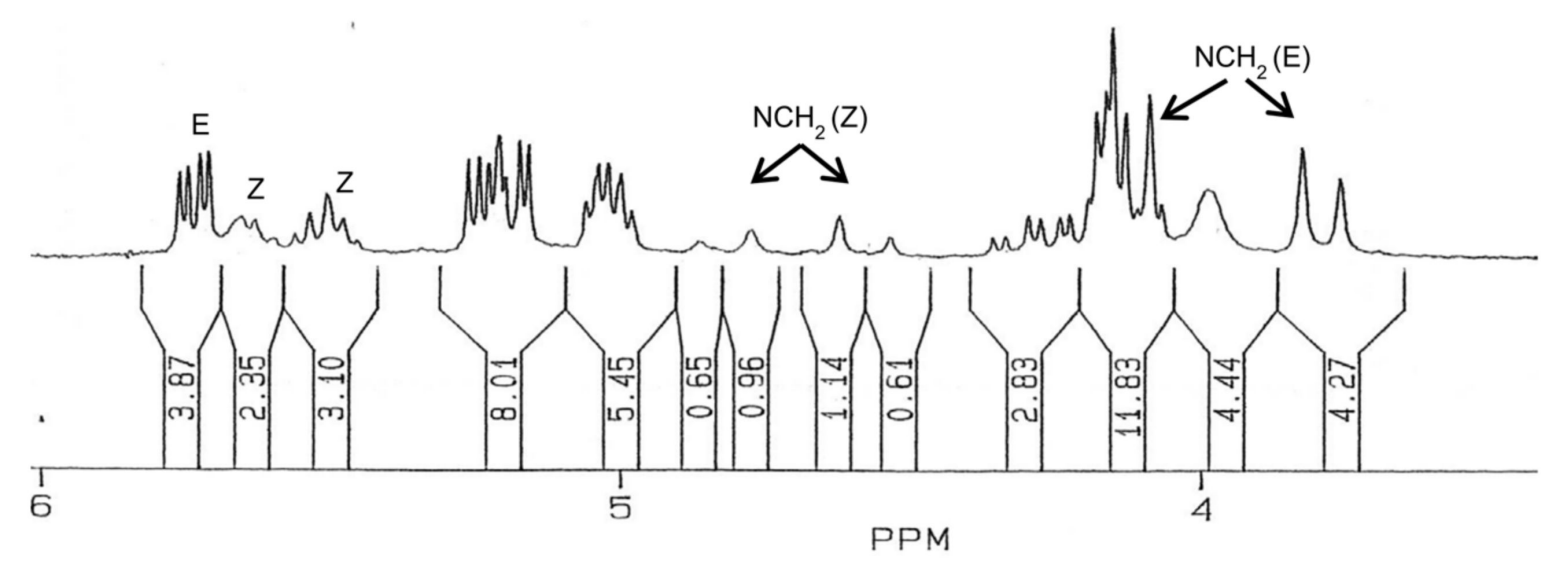
^1^H NMR spectrum (magnified zone between ca. 4 and 6 ppm) of compound **38**.

The ^1^H NMR spectrum of **38** is more complex than that of **37**, and shows a duplicity of signals (Figure 4). This duplicity does not result from an epimeric nitrile at C-2, having a d-*glycero*-l-*manno* configuration, since its synthesis took place from pure **33**. Initially, we thought of a mixture of two acetylated nitriles, i.e., both acylated and not acylated at the nitrogen atom. However, after successive trials and conducting the acetylation procedure at different temperatures or in the presence of sodium acetate or using 4-dimethylaminopyridine, the latter being known as an efficient nucleophilic catalyst in the acetylation of sterically hindered positions [104,105,106], no changes occurred. In addition, chromatographic analysis showed a single compound. We reasoned that the signal duplication should most likely be ascribed to a mixture of rotamers around the amide bond. Overall, the spectral patterns are similar to those observed for **37**, nevertheless.

**Scheme 7:**
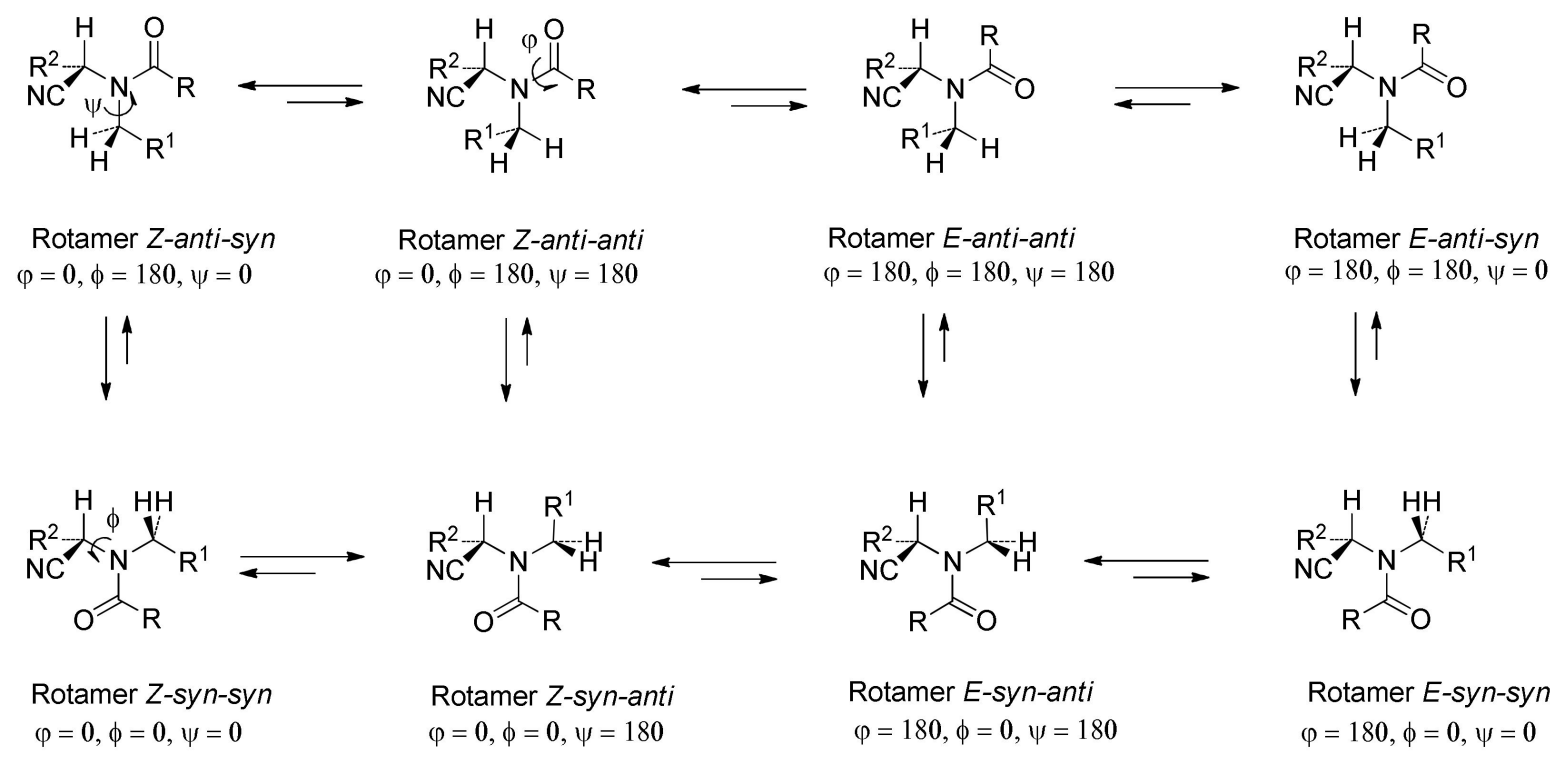
*E*/*Z* rotamers around *N*-alkyl and acyl amido moieties.

Scheme 7 (R = Me, R^1^ = Ph) shows the possible rotamers for the benzyl and tertiary acetamido groups [107], along with a visual description of the φ, ϕ, and ψ angles for conformational analyses. The amide-group stereochemistry of **37** in the solid state (Figure 3) is *Z-syn-anti* (φ = 0, ϕ = 0, ψ = 180), but the chemical shift of H-2 indicates that the most stable rotamer in the solution must be the *Z*-*anti*-*syn* (φ = 0, ϕ = 180, ψ = 0) conformer [107], since this is the disposition that generates less steric hindrance, being the only one actually detected in both the ^1^H and ^13^C NMR spectra. 

In stark contrast, compound **38** in the solution not only shows signals similar to those presented for **37**, identifiable with the *Z*-*anti*-*syn* conformation (**38Z**, Table 3), but also another signal set emerges, which must correspond to the *E*-*syn* conformer (**38E**). In this conformation, the H-2 proton undergoes a strong shielding with respect to the chemical shift shown in the *Z*-*anti*-*syn* conformation (Δδ_H-2_ = δ_H-2_^Z^ − δ_H-2_^E^ ~ 1.6 ppm). This effect stems from two factors: namely the deshielding caused by the amide C=O group disappearing, and the H-2 proton residing in the shielding zone of the aromatic ring of the benzyl group (*E*-*syn*-*anti*). This behavior has already been described in tertiary amides, in which the CH proton adjacent to the amide group in the *Z*-*anti* orientation is shifted downfield, by ca. 1.4 ppm, relative to an *E* conformation [107,108]. Also, the existence of restricted rotation is evident through the signal shape, small and broad, of the C-2 and CH_2_ signals in the benzyl group of the *Z*-*anti* conformer.

### 2.5. Perbenzoyl Alkylaminonitriles 

It is also interesting to ascertain whether an increase in size of the acylating agent, such as in the case of benzoylation, could prevent the acylation at the nitrogen atom in alkyl aminonitriles due to steric effects. Consequently, we have reassessed the structures of **18** and **20**, to confirm whether, as described in [49], benzoylation at the nitrogen atom does actually occur. 

Again, the determination of molecular mass peaks using HRMS shows that benzoylation has taken place; thus, for example, compound **18** shows [M + H]^+^ at *m*/*z* 859.2833 in agreement with the MW of 858.2789 for C_51_H_42_N_2_O_11_. The FT-IR spectra of **18** and **20** show benzamido group absorptions at ~1660 cm^−1^, which, together with the absence of absorptions above 3100 cm^−1^ (attributable to NH groups), demonstrate unequivocally the benzoylation reaction at the nitrogen atom. The ^1^H NMR spectrum of **18**, recorded at 27 °C in Cl_3_CD, acetone-*d*_6_, or benzene-*d*_5_, shows broadened and unresolved signals corresponding to the chain protons, thus indicating restricted rotation around the amide bond. The same is observed when viewing the ^1^H NMR spectrum of its isomer **20**, of a d-*glycero*-d-*galacto* configuration. This behavior also appears in the ^13^C NMR spectra, where the corresponding carbon signals of the ethyl group could not be identified. Improved results were obtained through longer acquisition pulses that allow for greater relaxation of the nuclei, yet poorly resolved signals remained. 

**Figure 5:**
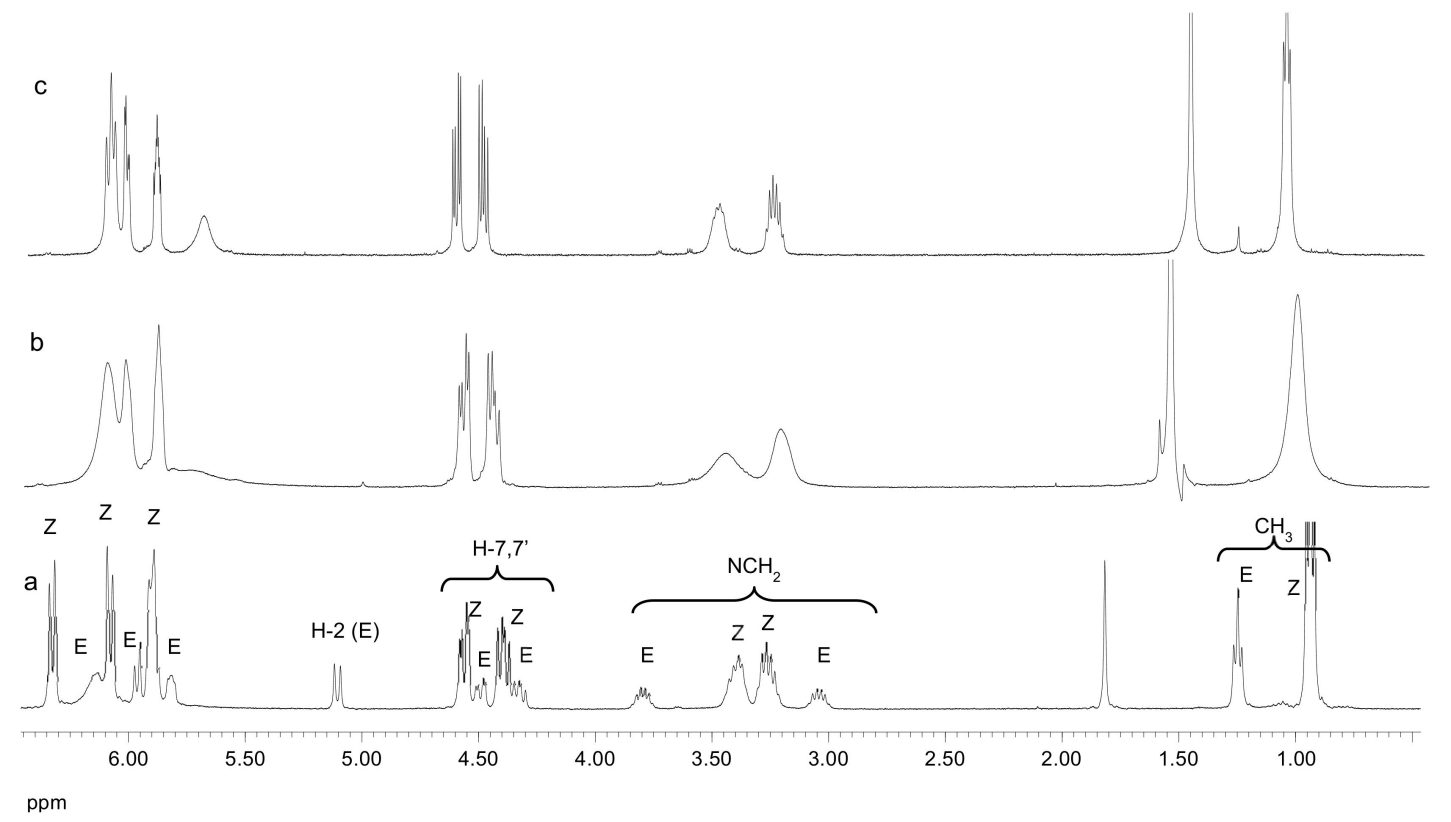
^1^H NMR spectra of **18** in Cl_3_CD: (**a**) 240 K, (**b**) 300 K, and (**c**) 327 K.

^1^H NMR spectra recorded above ambient temperature lead to sharper signals with improved resolution, which split into two signal sets by lowering the temperature at −50 °C (Figure 5). At this temperature, the minor rotamer in Cl_3_CD, estimated from signal integration, amounts to 23%. 

The methylene protons of the ethyl group are diastereotopic. Interestingly, the minor rotamer shows a significant difference between the chemical shifts of both protons (0.76 ppm versus 0.11 ppm). This large difference and the appearance of a doublet signal at 5.11 ppm, attributable to H-2, represent diagnostic features used to assign the stereochemistry of each rotamer.

**Figure 6:**
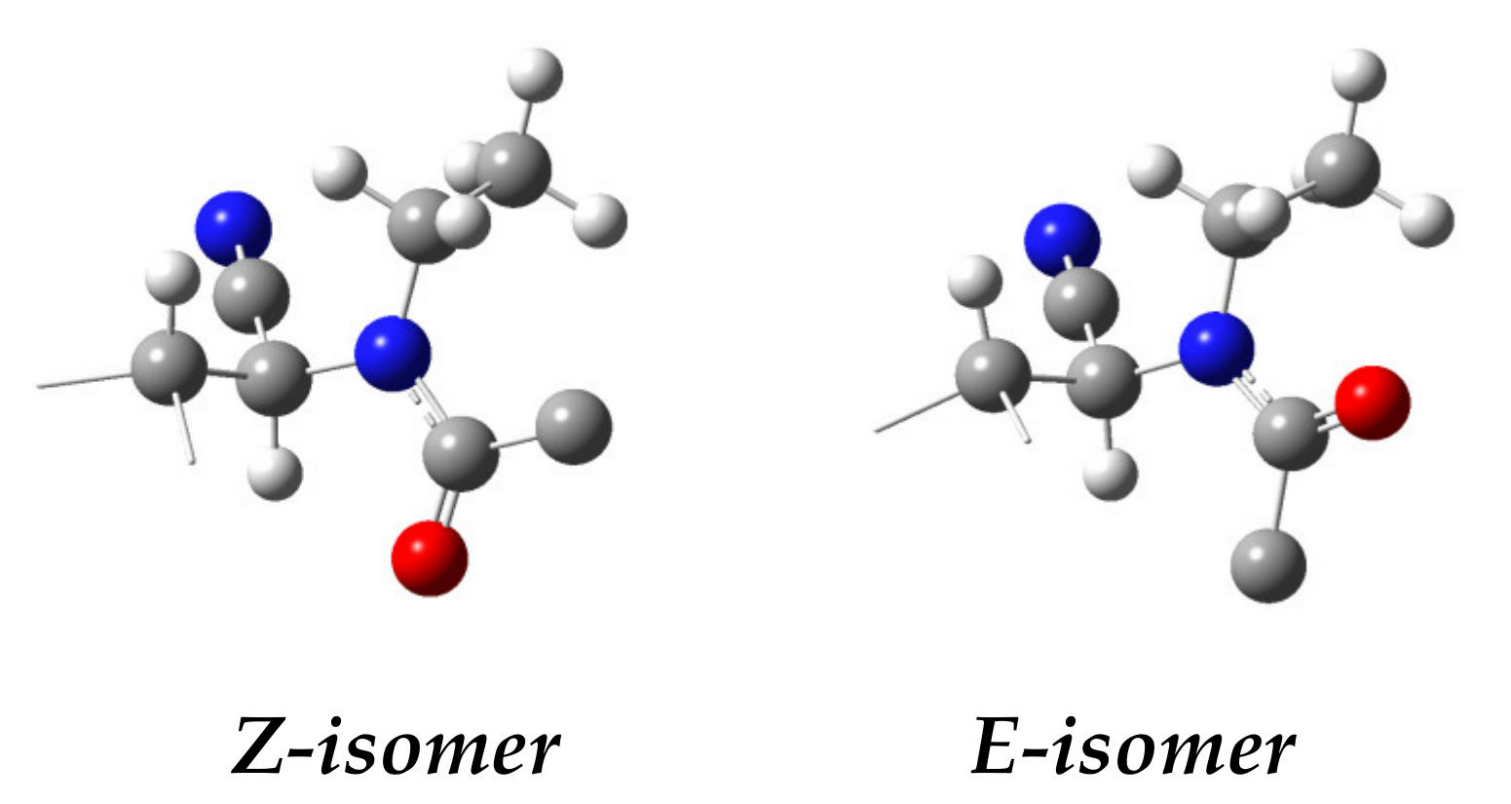
Ball-and-stick models for the spatial arrangements of the *Z* and *E* isomers.

Scheme 7 (R = Ph, R^1^ = Me) depicts the geometric arrangement of the possible rotamers of **18**. The more stable rotamer in the solution shows a *Z*-*anti* conformation, as occurs in the solid state for **13**. In this disposition, the amide carbonyl lies parallel to the C2-H2 bond, whereas an *E*-*anti*-*anti* stereochemistry (φ = 180, ϕ = 180, ψ = 180) places the C=O parallel to one of the methylene hydrogens of the ethyl group, which causes different chemical shifts for the two methylene protons. At the same time, the aromatic ring is close to the H-2, which results in a strong shielding of ~0.77 ppm and shifts the signal at 5.11 ppm (Figure 6).

This structural feature has also been highlighted in secondary amides, -CONHCHR-, where the chemical shift of the N-CHR proton located in a Z-*anti* orientation to the NH proton consistently resonates ~0.8 ppm further downfield than the proton located in a gauche disposition [107], as well as in tertiary amides and amide bond rotamers of 5-acyl-6,7-dihydrothieno [3,2-*c*]pyridine derivatives [108].

**Figure 7:**
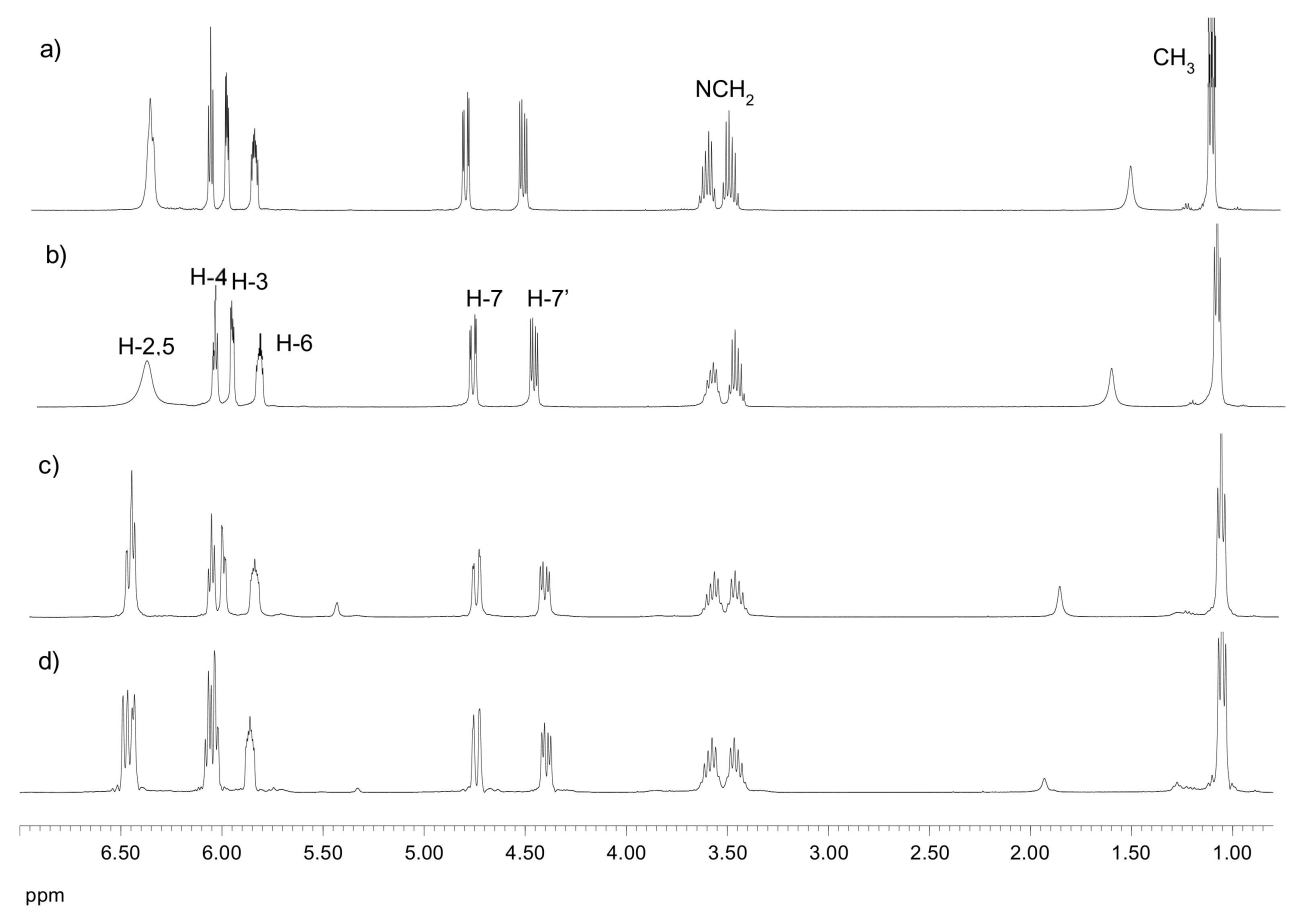
^1^H NMR spectra of **20** recorded in Cl_3_CD: (**a**) 330 K, (**b**) 300 K, (**c**) 256 K, and (**d**) 235 K.

In variable temperature experiments, the benzoylated derivative **20**, having a d-*glycero*-d-*galacto* configuration, shows apparently a simpler thermal behavior with well-resolved NMR signals. At low temperatures, the appearance of multiple signals corresponding to a minor rotamer is observed, like in the case of **18**. At 256 K, a small signal at ~5.35 ppm is detected, which could be assigned to the H-2 proton of that rotamer. However, no splitting is observed for the signals of the methylene protons of the ethyl group (Figure 7).

### 2.6. Peracetyl Phenylaminonitriles

Both the steric and the electronic parameters of the phenyl group (Table 2) explain why this group inhibits the acetylation at the nitrogen atom and enables the preparation of compounds **17** [56] and **42**. To achieve the acetylation of the phenylamino group leading to **43**, more drastic conditions must be used.

The presence of a sharp absorption peak at ~3350 cm^−1^, attributable to the stretching vibration of the NH bond, and the absence of the amide band at ~1660 cm^−1^, both in **17** and in **42**, confirm that the phenylamino group cannot be acetylated under the aforementioned conditions. In contrast, in the spectrum of **43**, the absorption of the NH disappears and the tertiary amide carbonyl appears at 1674 cm^−1^. The negligible absorption of the C≡N bond at 2200 cm^−1^ is noticeable as well. The absence or presence of acetylation at the nitrogen atom was evident anew through the determination of their molecular weights using mass spectrometry. Thus, **42** showed a [M + H]^+^ peak at *m*/*z* 421.1600, which corresponds to a molecular mass of 420.1533 for C_20_H_24_N_2_O_8_. However, compound **43** (462.1638 for C_22_H_26_N_2_O_9_) showed a [M + H]^+^ peak at *m*/*z* 463.1709, with a difference of 42 units with respect to that of **42**, clearly corresponding to the presence of the acetyl group at the nitrogen atom.

The ^1^H NMR spectrum of **42** shows only four acetates and the NH signal resonates as a doublet at 4.59 ppm, which disappears upon exchanging with D_2_O. The large coupling constant with the H-2 signal (*J*_NH,2_ 11.0 Hz) indicates that both protons adopt a relative antiperiplanar disposition. For compound **43**, however, the NH signal has disappeared and five acetyl groups are observed. The acetylation at the nitrogen atom shifts the H-2 signal from 4.71 ppm to 5.72 ppm. This deshielding of ~1 ppm arises from the *Z* arrangement adopted by the acetamido group, whose carbonyl is placed parallel to the C2-H bond. In the ^13^C NMR spectra of **17**, **42**, and **43**, the signals of nitrile functionality are clearly observed at ~115 ppm, whereas for **43**, the signal of the methyl carbon of the acetamido group resonates at ~22.55 ppm.

**Figure 8:**
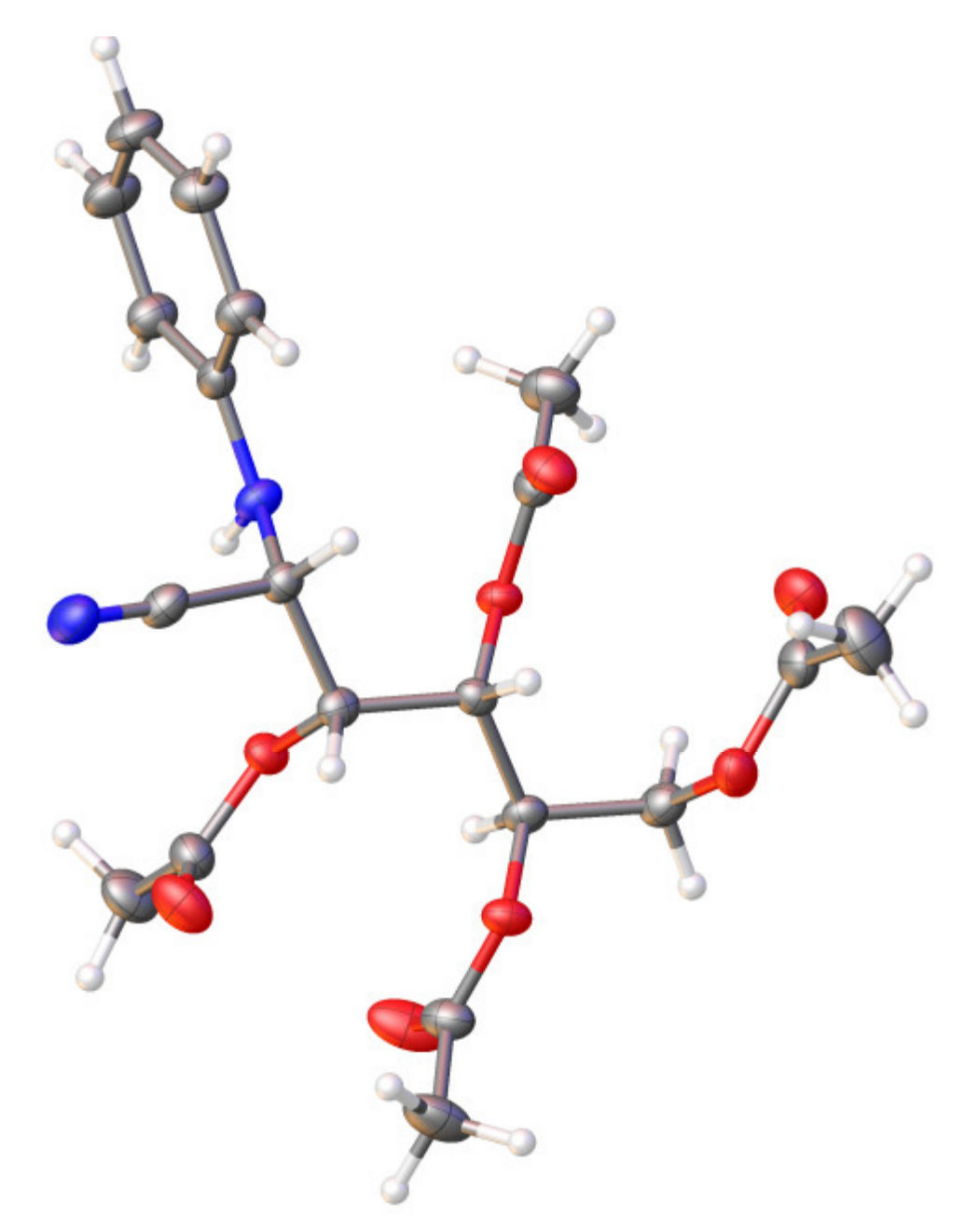
Single-crystal X-ray structure for **42** at 120 K. Thermal ellipsoids are drawn at 35% probability.

Figure 8 shows the structure of **42**, determined with X-ray diffraction at 120 K [83]. The crystal data confirm that there is no acetylation at the phenylamino group, probably due to both the steric effect created by the bulky phenyl group (Es ~ 2.6) and the decrease in nucleophilicity of the nitrogen atom (σ* = 0.60) when delocalizing its lone pair through the aromatic ring (see Table 2). In addition, the (*S*)-configuration at C-2 unequivocally allows us to assign the same stereochemistry to the C-2 atom of the starting nitrile **41**. Table 4 collects the geometric data of **42**, both experimental and calculated, which largely match each other.

### 2.7. Peracetyl Aminonitriles

**Figure 9:**
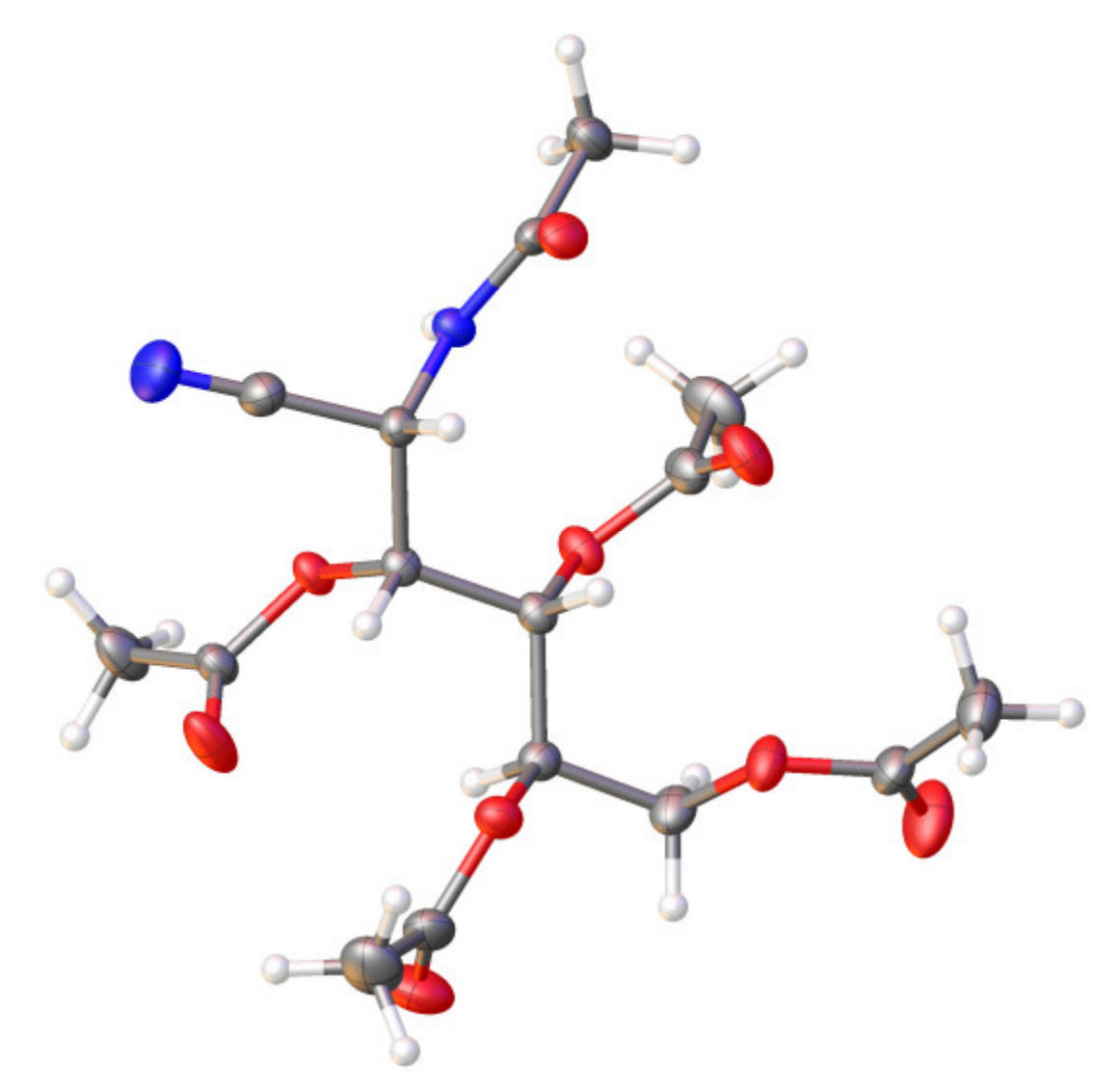
Single-crystal structure for compound **47** at 120 K. Thermal ellipsoids are drawn at 35% probability.

Compound **47** shows a [M + H]^+^ peak at *m*/*z* 387.1406, in agreement with the molecular mass of 386.1325 for C_16_H_22_N_2_O_9_. For compounds **47** and **51**, the NH absorption band of the secondary amide group appears at 3237 cm^−1^, with the first and second amide bands occurring at 1645 and 1541 cm^−1^, respectively. The stretching vibration of the C≡N group, generated by oxime dehydration, is hardly visible at ~2243 cm^−1^; however, the band is moderately intense in the Raman spectrum (also at 2243 cm^−1^). The ^1^H NMR spectra confirm likewise the structures of **47** and **51**, and thus the NH signal appears as a doublet at ~6.7 ppm. In the ^13^C NMR spectra, the carbon signals of the acetamido group are clearly distinguishable from those of the acetate groups: for example, in the case of **47**, such resonances appear at ~172 ppm and at ~22.9 ppm. As expected, the C≡N group resonates at ~116 ppm. Lastly, the unequivocal structure of compound **47** could be established with X-ray diffractometry (Figure 9) and shows the presence of the C≡N and acetamido groups at C-2.

Obviously, the stereochemistry at C-2 is *S*, the same configuration as that possessed by the parent oxime of d-glucosamine (**45**), since it is not affected during the elimination reaction from per-*O*-acetyl oxime **46**, which leads in turn to the formation of the C≡N group (Scheme 4) [109]. On the other hand, the acetamido group adopts a *Z*-*anti* arrangement. Table 5 shows some experimental and calculated geometric parameters of **47**.

### 2.8. Structural Assessment via Mass Spectrometry

**Scheme 8:**
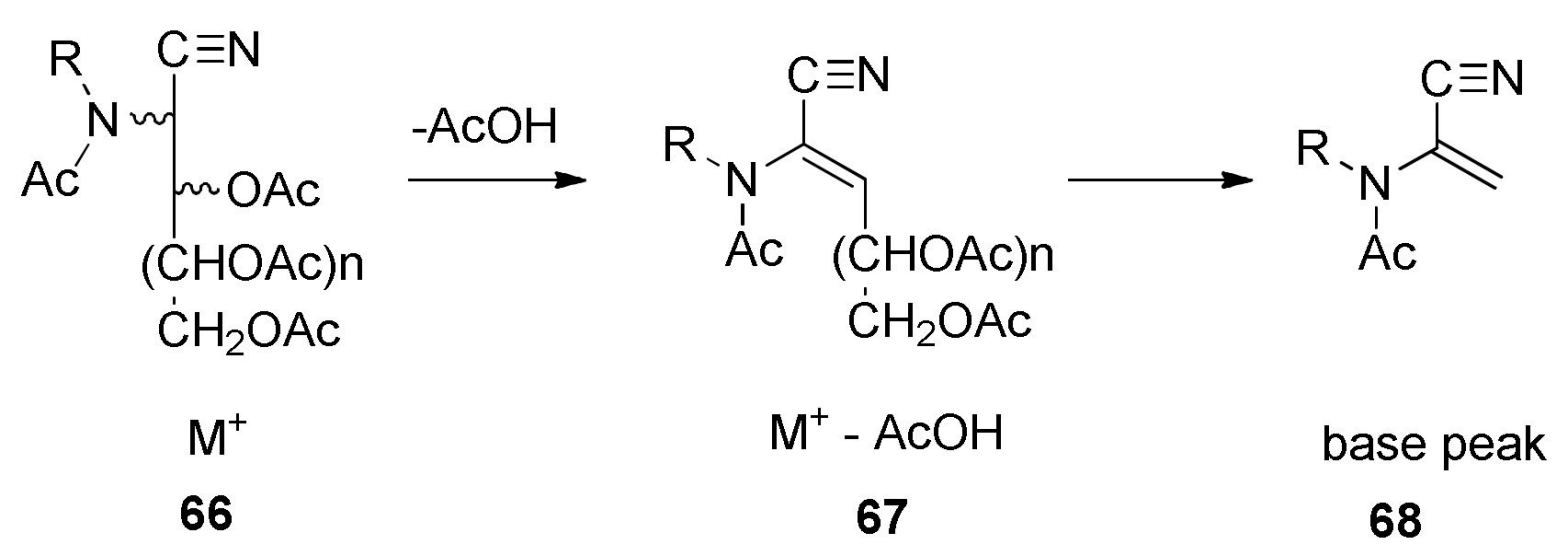
Typical EI-MS fragmentation patterns of per-*O*-acetyl-2-alkylaminonitriles.

Electron impact mass spectrometry provides valuable structural information on peracyl aminonitriles, which is usually lost in chemical ionization experiments. Accordingly, fragmentation patterns of some compounds were interrogated through EI-MS. In general, the [M^+^] peak is weak or does not appear at all for the per-*O*-acetyl-2-alkylacetamido-2-deoxyaldononitrile ion (**66**), while the second peak, appearing always, corresponds to the loss of acetic acid, [M^+^ − AcOH] (**67**), and then the fragmentation into the corresponding α,β-unsaturated 2-*N*-alkylacetamidonitrile (**68**), which is usually the basis peak (Scheme 8).

**Scheme 9:**
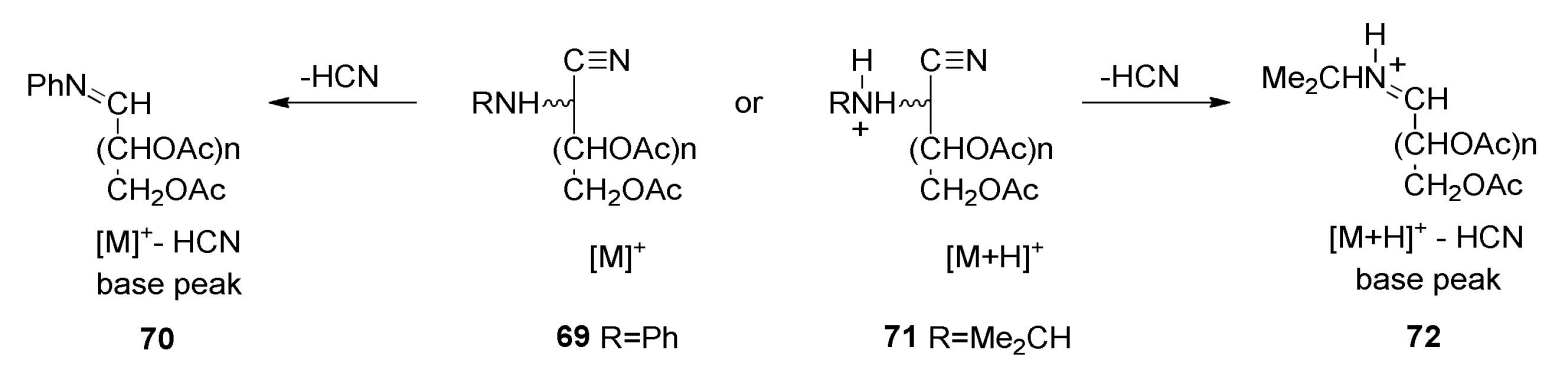
EI-MS fragmentation patterns of per-*O*-acetylated-2-alkylaminonitriles due to the loss of HCN.

When the nitrogen atom is not acylated (such as in **17** and **42**), a molecular [M]^+^ peak is observed and the loss of hydrogen cyanide (*m*/*z* 27) usually leads to the base peak (**70**). For more basic nitrogen atoms (like in **58**), a [M + H]^+^ peak (**71**) was recorded instead of [M]^+^. In this case, the base peak is [M + H-HCN]^+^ (**72**) (Scheme 9). Furthermore, the successive loss of acetic acid (*m*/*z* 60), radical acetate (*m*/*z* 59), ketene (*m*/*z* 42), and acetic anhydride (*m*/*z* 102) can also be typically detected in per-*O*-acetyl aldononitriles [110].

### 2.9. Conformational Analyses

Theoretical analyses have been conducted to determine the most stable conformational dispositions in both the gas phase and in a solution, which have also been compared with those obtained in the solid state. To this end, DFT calculations at the M06-2X/6-311G(d,p) level [111] were employed and we simulated the bulk solvent effects (in CHCl_3_) with the SMD method [112].

**Scheme 10:**
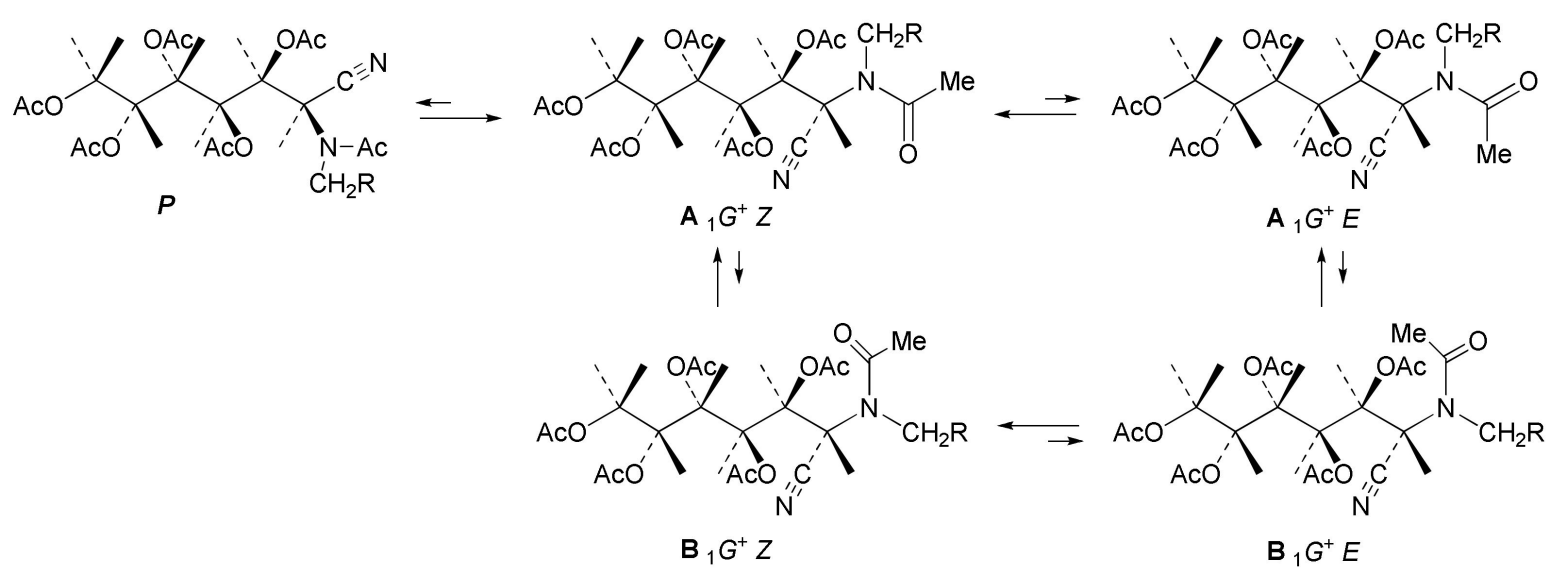
Conformational landscape for per-*O*-acylated aminonitriles.

As already mentioned, for the solid-state structures of **13**, **37**, **42**, and **47**, all of the acetamido and acetate groups adopt a *Z* stereochemistry (Scheme 6). Therefore, such a disposition was chosen as the starting point to obtain the computational estimations for the aforementioned compounds. Due to the small size and linear geometry of the C≡N group, these compounds do not adopt a *P* conformation with the chain fully extended in a zig-zag; they are more inclined towards the sickle conformation ^1^*G*_+_, which is reached through a 120^°^ clockwise turn of C-1 (Scheme 10) [113,114]. In this way, the 1,3-diaxial interaction between the *N*-ethylacetamido group and the acetate at C-4 is eliminated. Table 6 shows the results obtained when calculating the relative stabilities of the _1_*G*^+^ and _1_*G*^−^ conformations and the *Z*/*E* dispositions of the *N*-ethylacetamido group (letters A and B refer to different orientations of the substituted nitrogen with respect to the carbon chain, while stereochemical descriptors *E* and *Z* refer to the configuration adopted by the amide group).

**Figure 10:**
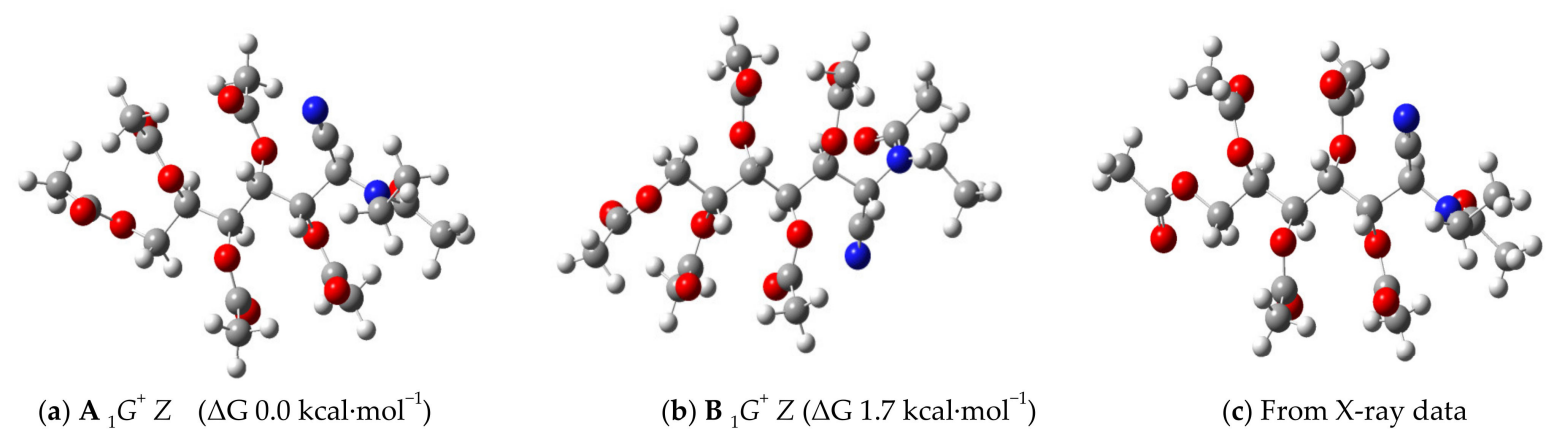
Most stable conformers in solution (**a**,**b**) and in the solid state (**c**) for aminonitrile **13**.

Figure 10 shows the calculated structures of the two most stable conformers. The most stable, both in the gas phase and in the presence of a solvent, is **A**_1_*G*^+^ Z. The _1_*G*^+^ chain conformation is supported by the coupling constants measured in the CDCl_3_ solution (Table 6). The high values of *J*_2,3_ and *J*_4.5_ (10.0 Hz) indicate an antiperiplanar arrangement between H-2 and H-3, and the same occurs with H-4 and H-5. In contrast, the low values of *J*_3,4_ and *J*_5,6_ (<2 Hz) confirm a gauche arrangement between such hydrogen atoms.

The geometric data of the **A**_1_*G*^+^ Z conformation are included in Table 4 and are quite similar to those found in the solid state. For comparative purposes, Figure 10c shows the structure of the conformer determined with X-ray diffraction; it can be seen that the only appreciable difference with the most stable **A**_1_*G*^+^ Z conformation is the arrangement adopted by the acetate group at the end of the chain (C-7). The conformational analysis performed for **37** is similar to that discussed for **13** (Scheme 10, R = Ph, Table 7).

**Figure 11:**
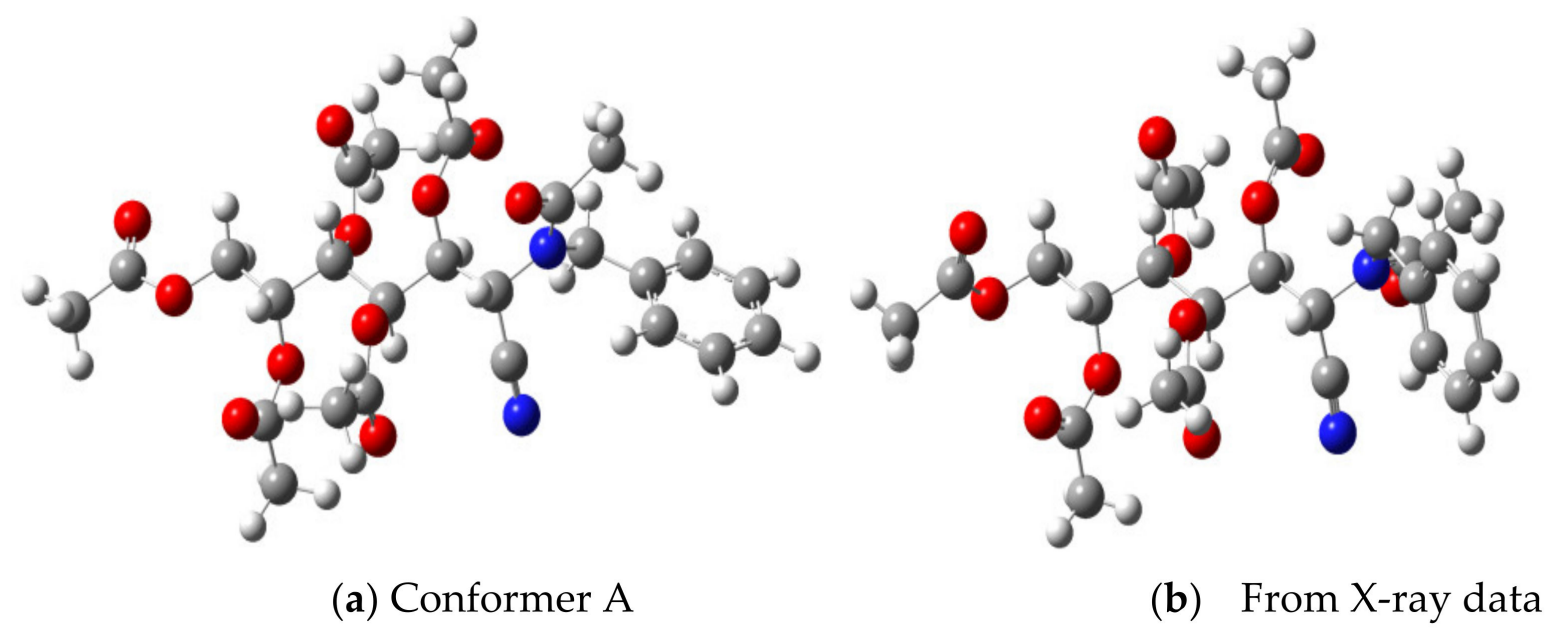
Structure of conformer **A** _1_*G*^+^*Z* (**a**) and X-ray structure (**b**) of **37**.

The data gathered in Table 7 indicate that, like in the case with **13**, the most stable conformer in the CDCl_3_ solution is **A**_1_*G*^+^ *Z*, followed by **B**_1_*G*^+^ *Z*. The former is practically identical with the conformer present in the crystalline unit cell (see the geometric data in Table 6): only the arrangement of the aromatic ring changes (Figure 11).

When the conformational study was conducted on aminonitrile **42**, similar results were obtained again (Table 8), not surprisingly as the absolute configurations of all carbon atoms (d-*gluco* chain) are mirror images of the first six stereogenic carbons of **13** and **37** (l-*gluco* chain).

**Figure 12:**
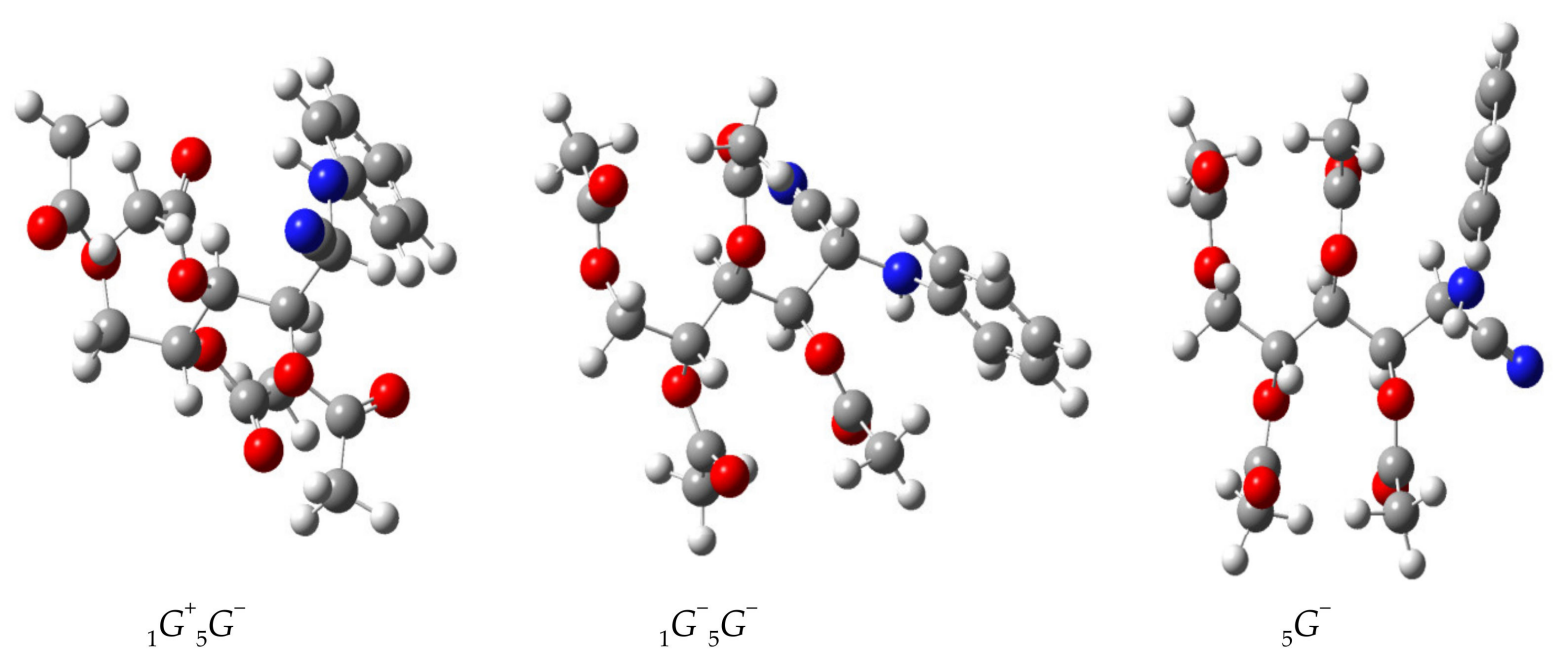
Calculated structures of the most stable conformers of **42**.

The most stable conformer in solution is _1_*G*^+^_5_*G*^−^, followed by _1_*G*^−^_5_*G*^−^. Then, the conformer _5_*G*^−^ follows in stability, which is almost coincidental with the arrangement present in the solid-state unit cell (see the geometric data in Table 7 and Figure 12).

**Figure 13:**
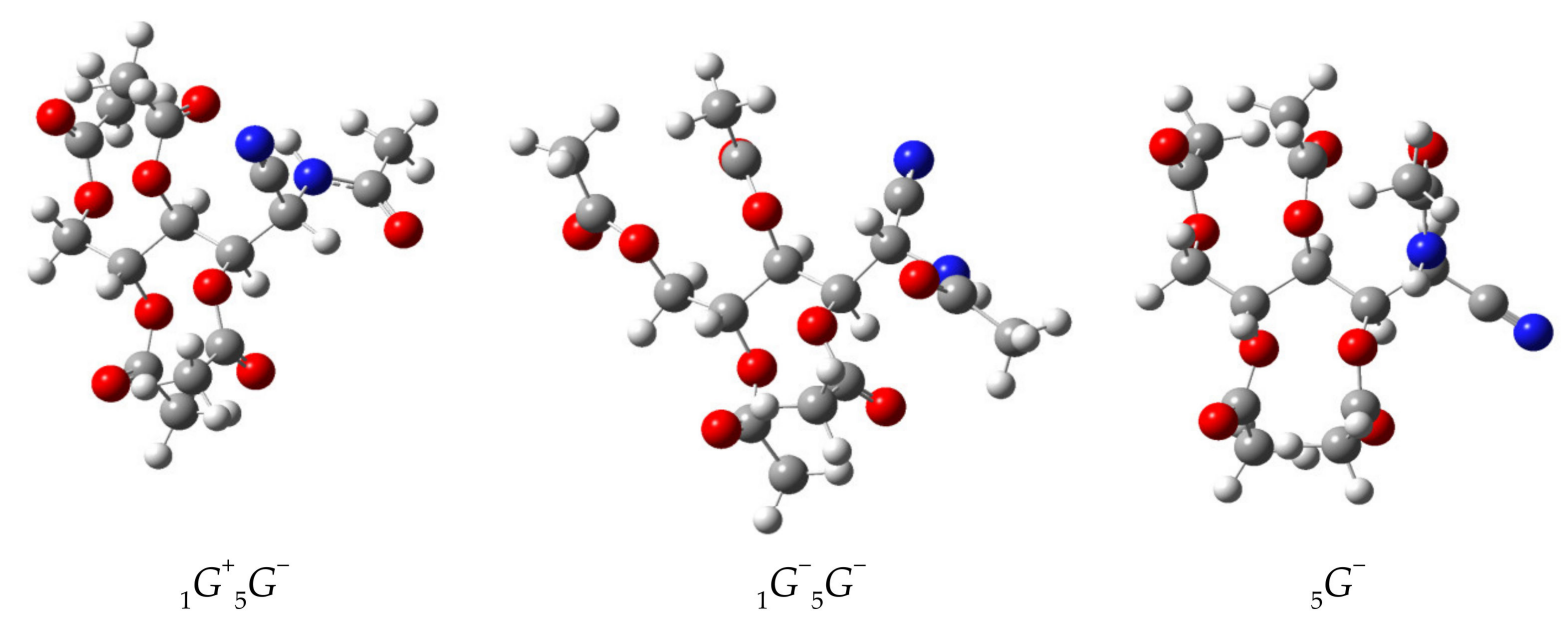
Calculated structures of the most stable conformers of **47**.

Finally, the results obtained in the conformational analysis of **47** agree well with those found for **42**, since both share an identical stereochemical pattern (Table 9). Again, the most stable conformer in chloroform is _1_*G*^+^_5_*G*^−^, while conformers _1_*G*^−^_5_*G*^−^ and _5_*G*^−^ exhibit practically identical stability. The third one represents the conformational disposition obtained in the unit cell (Table 8, Figure 13).

The experimental data confirm the conclusions reached through the above calculations. The high values of *J*_2,NH_ (11.0 Hz) in both **42** and **47** are indicative of an antiperiplanar arrangement between the NH and H-2 atoms. In addition, the intermediate values of *J*_2,3_ (~6–7 Hz) being between those expected for *anti* (10 Hz) and *syn* (<2 Hz) arrangements evidence that there must be an equilibrium involving the _1_*G*^+^_5_*G*^−^ and _1_*G*^−^_5_*G*^−^ conformers in solution.

## 3. Conclusions

To summarize, this structural reinvestigation evidences that 2-amino-2-deoxyaldononitriles bearing unbranched alkyl groups at the α-carbon (methyl, ethyl, *n*-propyl, or benzyl groups) are acylated under standard conditions. However, the presence of an isopropyl group at the nitrogen atom impedes acetylation, most likely owing to a steric hindrance to the reagents being brought together. These steric effects are also disclosed in the addition of anhydrous hydrogen cyanide to *N*-isopropyl mannosylamine, leading to a nitrile derivative instead of the corresponding α-aminonitrile.

Acylation of the NH_2_ group prevents further acylation of the amidic nitrogen. In addition, when the nitrogen atom carries a phenyl group, lone-pair delocalization decreases the nucleophilicity of the former and also prevents the acylation reaction, although the reaction can be achieved under more drastic conditions.

Unambiguous structural elucidation via spectroscopic methods and X-ray diffraction has allowed us to assign the configuration at the C-2 atom of the starting 2-amino-2-deoxy-aldononitriles. This is the chiral center generated during cyanohydrin synthesis, whose chirality has previously only been assigned empirically through optical rotation correlations. Therefore, by justifying the empirical rule accounting for the major epimer of α-aminonitriles, the new chiral center generated here bears a 2,3-*threo* stereochemical relationship with the adjacent chiral center.

The tertiary amide group showed restricted rotation, and theoretical calculations predicted the most stable conformers observed. These results were compared with those obtained through coupling constants (^3^*J*) for the protons observed in solution.

## 4. Experimental Methodology

### 4.1. General Methods

All solvents and chemicals were purchased from commercial suppliers and used without further purification. Solvents were evaporated below 40 °C at pressures between 15 and 30 mm Hg. Melting points were measured on an Electrothermal 9100 apparatus in capillary tubes and were uncorrected. Optical rotations were determined on a Perkin-Elmer 141 polarimeter at different wavelengths (Na and Hg lamps) at 18 ± 2 °C, as specified. Microanalyses for C, H, and N were performed using a Leco CHNS-932 analyzer (Leco Corporation, St. Joseph, MI, USA). IR spectra were recorded on Perkin-Elmer 399 or FT-IR THERMO spectrophotometers in the range of 4000–600 cm^−1^ using KBr pellets (Merck, Darmstadt, Germany). Raman spectra were recorded in a Nicolet Almega XR apparatus (Thermo Fisher Scientific, Waltham, MA, USA). Analytical thin-layer chromatography (TLC) was performed on Aldrich Polygram Sil G/UV254 plates employing the following eluent systems: (a) ethyl ether–petroleum ether (3:1) and (b) ethyl ether–petroleum ether (1:1), with subsequent monitoring with UV light at 254 and 360 nm, and iodine vapors. ^1^H and ^13^C{^1^H} NMR spectra were recorded on Bruker spectrometers operating at 200/400/500 and 50/100/125 MHz, respectively, in different solvent systems. Chemical shifts are reported in parts per million (δ) downfield from tetramethylsilane (Me_4_Si, TMS) as an internal reference. Coupling constants (*J* values) are given in Hz and standard abbreviations are used to indicate spin multiplicities, namely s = singlet, bs = broad singlet, d = doublet, t = triplet, q = quartet, dd = doublet of doublet, ddd = doublet of doublet of doublet, sep = septuplet, and m = multiplet. Carbon chemical shifts in the decoupled 13C{1H} NMR spectra are reported relative to CHCl_3_ (δ_C_ 77.00 ppm, central line of triplet). Assignments were confirmed using homo- and hetero-nuclear double-resonance, DEPT (distortionless enhancement by polarization transfer), and 2D proton–proton (COSY) and proton–carbon (HMQC) correlations. EI–mass spectra were recorded with a Kratos MS-SORFA, using a direct insertion probe and heating between 30 °C and the melting point of the samples. High-resolution mass spectra, HRMS (ESI-TOF), were measured using ESI ionization techniques with the 6520 Accurate-Mass Q-TOF LC/MS equipment from Agilent Technologies (Santa Clara, CA, USA) at the Servicio de Apoyo a la Investigación (SAIUEX) of the University of Extremadura.

### 4.2. X-ray Data Collection and Structural Refinement

Air-stable, single crystals of **13**, **37**, **42**, and **63** suitable for X-ray diffraction were mounted on a Nonius Kappa CCD diffractometer equipped with monochromated Mo-K_α_ radiation (λ = 0.71073 Å). Crystal data were collected using the software COLLECT Nonius [115] at low temperatures, usually 100.0 or 120.0 K, as specified in every case. Data reduction and cell refinements were carried out using DENZO [116]. Each structure was solved using SHELXS-97 [117] and refined with SHELXL-97 [118]. Crystallographic illustrations were prepared using the CAMERON software (X-LIB version) [119]. For compound **47**, crystal data were acquired with a Bruker Kappa APEX-II diffractometer using Mo-K_α_ radiation (λ = 0.71073 Å) at 170.0 K, and refined with the above-mentioned protocols.

Crystal data have been deposited within the Cambridge Structural Database with the following registry numbers: CCDC: 2332391 (for **13**), CCDC: 2332392 (for **37**), CCDC: 2332393 (for **42**), CCDC: 2334875 (for **47**), and CCDC: 2332394 (for **63**), which contain the supplementary crystallographic data for this paper. These data can be obtained free of charge from the Cambridge Crystallographic Data Centre via www.ccdc.cam.ac.uk/data_request/cif (accessed on 15 March 2024).

### 4.3. Computational Details

DFT calculations were carried out using the Gaussian09 suite of programs [120]. The M06-2X [111] hybrid functional was chosen for performing the geometry optimizations and frequency analyses, in conjunction with the 6-311G(d,p) basis set for all atoms [121]. Stationary points were confirmed via frequency analysis at the above-mentioned level of theory at 298.15 K. Solvent effects were modeled according to the geometry optimization of the corresponding gas-phase structures using the solvation model density (SMD) method [112].

### 4.4. Synthetic Procedures

All products described and fully characterized herein were obtained using methods reported in the literature or according to the procedure outlined in each case (**11** [53], **12** [53], **13** [51], **17** [56], **18** [51], **20** [51], **26** [52], **32** [49], **33** [49], **45** [61], **47** [61], **48** [67], **54** [50], **55** [50], **57** [99], **63** [99], and **64** [99]). Caution: The preparation of carbohydrate nitriles involving cyanohydrin synthesis requires the in situ generation of liquid hydrogen cyanide. This is very poisonous, highly volatile, and should be handled with extreme caution and ensuring effective ventilation.

3,4,5,6-Tetra-*O*-acetyl-2-deoxy-2-(*N*-ethylacetamido)-l-glucononitrile *(***11***)* [53]. Colorless needles, m.p. 103–105 °C, [α]_D_^20^ −49.0°; [α]_578_^20^ −51.4°; [α]_546_^20^ −58.5°, [α]_436_^20^ −100.6°, [α]_365_^20^ −160.5° (*c* 1.0, chloroform); IR (KBr) ṽ_max_/cm^−1^ 2239 (C≡N), 1755, 1649 (C=O), 1274, 1262, 1206 (C-O-C), 1057, 1029 (C-O); Raman ṽ_max_/cm^−1^ 2239 (C≡N), 1736 (C=O); ^1^H NMR (500 MHz, CDCl_3_) δ 5.57 (m, 2H, H-3, H-4), 5.48 (d, *J*_2,3_ = 9.0 Hz, 1H, H-2), 5.11 (ddd, *J*_5,6_ = 3.0 Hz, *J*_5,6′_ = 5.0 Hz, *J*_4,5_ = 8.5 Hz, H-5), 4.27 (dd, *J*_5,6_ = 3.0 Hz, *J*_6,6′_ = 12.5 Hz, 1H, H-6), 4.07 (dd, *J*_5,6′_ = 5.0 Hz, *J*_6,6′_ = 12.5 Hz, 1H, H-6′), 3.41 (m, 2H, CH_2_), 2.20, 2.12, 2.08, 2.07, 2.06, 2.05 (s, 15H, CH_3_), 1.35 (t, *J* = 7.0 Hz, 3H, CH_3_); ^13^C{^1^H} NMR (125 MHz, CDCl_3_) δ 170.88, 170.53, 169.69, 169.46, 169.36 (C=O), 114.78 (C≡N), 68.20, 68.04, 67.85 (C-3, C-4, C-5), 61.56 (C-6), 45.34 (C-2), 42.86 (CH_2_), 21.09, 20.73, 20.65, 20.52, 20.33, 14.48 (CH_3_). HRMS-(ESI-TOF) *m*/*z* [M + H]^+^ calcd. for C_18_H_27_N_2_O_9_: 415.1717. Found: 415.1706. *m*/*z* [M + NH_4_]^+^ calcd. for C_18_H_30_N_3_O_9_: 432.1982. Found: 432.1969. *m*/*z* [M_2_ + Na]^+^ calcd. for C_36_H_52_N_4_O_18_Na: 851.3174. Found: 851.3133.
3,4,5,6-Tetra-*O*-acetyl-2-deoxy-2-(*N*-*n*-propylacetamido)-l-glucononitrile (**12**) [53]. Colorless needles, m.p. 115–116 °C, [α]_D_^20^ −48.0°; [α]_578_^20^ −48.9°; [α]_546_^20^ −55.5°, [α]_436_^20^ −95.6°, [α]_365_^20^ −154.2° (*c* 0.4, chloroform); IR (KBr) ṽ_max_/cm^−1^ 2233 (C≡N), 1750 (C=O ester), 1655 (C=O amide), 1415, 1360, 1258 and 1210 (C-O-C), 1057, 1030 (C-O); ^1^H NMR (400 MHz, CDCl_3_) δ 5.60 (t, 2H, H-3, H-4), 5.34 (d, *J*_2,3_ = 8.8 Hz, 1H, H-2), 5.14 (m, *J*_5,6_ = 3.2 Hz, *J*_5,6′_ = 4.8 Hz, H-5), 4.27 (dd, *J*_5,6_ = 3.0 Hz, *J*_6,6′_ = 12.0 Hz, 1H, H-6), 4.07 (dd, *J*_5,6′_ = 3.0 Hz, *J*_6,6′_ = 12.0 Hz, 1H, H-6′), 3.25 (m, 2H, CH_2_), 2.21, 2.22, 2.08, 2.07, 2.05 (s, 15H, CH_3_), 0.97 (t, *J* = 7.2 Hz, 3H, CH_3_); ^13^C{^1^H} NMR (100 MHz, CDCl_3_) δ 170.94, 170.54, 169.72, 169.44, 169.32 (C=O), 114.70 (C≡N), 68.15, 68.08, 67.87 (C-3, C-4, C-5), 61.57 (C-6), 45.94 (C-2), 50.21, 22.54 (CH_2_), 21.11, 20.74, 20.65, 20.52, 20.35, 11.19 (CH_3_).
3,4,5,6,7-Penta-*O*-acetyl-2-deoxy-2-(*N*-ethylacetamido)-d-*glycero*-l-*gluco*-heptononitrile (**13**) [51]. Colorless needles, m.p. 120–122 °C, [α]_D_^20^ −11.8°; [α]_578_^20^ −11.8°; [α]_546_^20^ −15.0°, [α]_436_^20^ −26.0° (*c* 0.5, chloroform); IR (KBr) ṽ_max_/cm^−1^ 2255 (C≡N), 1749, 1662 (C=O), 1275, 1203 (C-O-C), 1067, 1036 (C-O); Raman ṽ_max_/cm^−1^ 2254 (C≡N), 1743, 1661 (C=O); ^1^H-NMR (500 MHz, CDCl_3_) δ 5.57 (dd, *J*_3,4_ = 1.5 Hz, *J*_2,3_ = 10.0 Hz, 1H, H-3), 5.44 (dd, *J*_3,4_ = 1.5 Hz, *J*_4,5_ = 10.0 Hz, 1H, H-4), 5.38 (d, *J*_2,3_ = 10.0 Hz, 1H, H-2), 5.30 (dd, *J*_5,6_ = 2.0 Hz, *J*_4,5_ = 7.5 Hz, 1H, H-5), 5.23 (ddd, *J*_5,6_ = 1.5 Hz, *J*_6,7_ = 5.0 Hz, *J*_6,7′_ = 7.0 Hz, H-6), 4.28 (dd, *J*_6,7_ = 5.5 Hz, *J*_7,7′_ = 12.0 Hz, 1H, H-7), 3.85 (dd, *J*_6,7′_ = 7.0 Hz, *J*_7,7′_ = 11.5 Hz, 1H, H-7′), 3.39 (m, 2H, CH_2_), 2.21, 2.15, 2.14, 2.12, 2.06, 2.04 (s, 18H, CH_3_), 1.35 (t, *J* = 7.0 Hz, 3H, CH_3_); ^13^C{^1^H} NMR (100 MHz, CDCl_3_) δ 170.70, 170.40, 170.32, 169.74, 169.61, 169.44 (C=O), 114.81 (C≡N), 67.76, 67.40, 67.36, 66.84 (C-3, C-4, C-5, C-6), 61.85 (C-7), 45.16 (C-2), 42.94 (CH_2_), 21.06, 20.73, 20.61, 20.57, 20.49, 20.38, 14.51 (CH_3_). HRMS-(ESI-TOF) *m*/*z* [M + H]^+^ calcd. for C_21_H_31_N_2_O_11_: 487.1928. Found: 487.1904. *m*/*z* [M + NH_4_]^+^ calcd. for C_21_H_34_N_3_O_11_: 504.2193. Found: 504.2158.
3,4,5,6,7-Penta-*O*-acetyl-2-deoxy-2-(*N*-phenylamino)-d-*glycero*-d-*altro*-heptononitrile (**17**) [56]. White solid, m.p. 100–102 °C, [α]_D_^20^ −43.6°; [α]_578_^20^ −45.9°; [α]_546_^20^ −55.2°, [α]_436_^20^ −103.2°, [α]_365_^20^ −183.6° (*c* 0.5, chloroform); IR (KBr) ṽ_max_/cm^−1^ 3349 (NH), 2229 (C≡N), 1771, 1747 (C=O), 1509 (NH), 1252, 1231, 1213 (C-O-C), 1061 (C-O); ^1^H NMR (500 MHz, CDCl_3_) δ 7.26 (m, 2H, H-arom), 6.91 (t, 1H, H-arom), 6.70 (d, 2H, H-arom), 5.62 (dd, *J*_2,3_ = 4.0 Hz, *J*_3,4_ = 7.0 Hz, 1H, H-3), 5.46 (dd, *J*_4,5_ = 3.5 Hz, *J*_3,4_ = 7.0 Hz, 1H, H-4), 5.40 (dd, *J*_4,5_ = 3.5 Hz, *J*_5,6_ = 7.0 Hz, 1H, H-5), 5.30 (ddd, *J*_6,7_ = 3.0 Hz, *J*_6,7′_ = 6.0 Hz, *J*_5,6_ = 7.0 Hz, 1H, H-6), 4.52 (dd, *J*_2,3_ = 4.0 Hz, *J*_2,*NH*_ = 11.0 Hz, 1H, H-2), 4.37 (dd, *J*_6,7_ = 3.0 Hz, *J*_7,7′_ = 12.5 Hz, 1H, H-7), 4.11 (d, *J*_2,*NH*_ = 11.0 Hz, 1H, NH), 4.10 (dd, *J*_6,7′_ = 6.0 Hz, *J*_7,7′_ = 12.5 Hz, 1H, H-7′), 2.23, 2.12, 2.07, 2.06, 2.05 (s, 15H, CH_3_); ^13^C{^1^H} NMR (100 MHz, CDCl_3_) δ 170.60, 170.01, 169.83, 169.53, 169.27, 169.11 (C=O), 143.96, 129.70, 120.91 (C-arom), 116.70 (C≡N), 114.46 (C-arom), 69.84, 69.65, 69.21 (C-3, C-4, C-5, C-6), 61.75 (C-7), 47.26 (C-2), 20.86, 20.67, 20.62 (CH_3_).
3,4,5,6,7-Penta-*O*-benzoyl-2-deoxy-2-(*N*-ethylbenzamido)-d-*glycero*-l-*gluco*-heptononitrile (**18**) [51]. White solid, m.p. 79–81 °C, [α]_D_^20^ −9.4°; [α]_578_^20^ −10.0°; [α]_546_^20^ −11.4°, [α]_436_^20^ −12.6° (*c* 0.5, chloroform) [Lit. [51] m. p. 80 °C, [α]_D_^20^ −23.9° (*c* 0.3, pyridine)]; IR (KBr) ṽ_max_/cm^−1^ 1730, 1655 (C=O), 1259 (C-O-C), 1090, 1068, 1024 (C-O); Raman ṽ_max_/cm^−1^ 2250 (C≡N), 1732, 1658 (C=O); ^1^H NMR (500 MHz, CDCl_3_) δ 8.10–7.00 (m, 30H, H-arom), 6.12 (bs, 2H, H-3, H-4), 6.04 (bs, 1H, H-5), 5.91 (s, 1H, H-6), 5.76 (bs, 1H, H-2), 4.60 (dd, *J*_6,7_ = 4.5 Hz, *J*_7,7′_ = 11.5 Hz, 1H, H-7), 4.48 (dd, *J*_6,7′_ = 7.0 Hz, *J*_7,7′_ = 11.5 Hz, 1H, H-7), 3.47 (bs, 1H, CH_2_), 3.26 (bs, 1H, CH_2_), 1.05 (bs, 3H, CH_3_); ^13^C{^1^H} NMR (100 MHz, CDCl_3_) δ 171.90, 171.85, 165.85, 165.37, 165.23, 165.01(C=O),134.45, 133.98, 133.88, 133.77, 133.36, 133.01, 130.13, 130.08, 130.03, 1298.88, 129.69, 129.27, 128.72, 128.66, 128.47, 128.44, 128.24, 126.41 (C-arom), 114.87 (C≡N), 69.70, 69.17, 68.68 (C-3, C-4, C-5, C-6), 62.86 (C-7), 47.52 (C-2), 43.44 (CH_2_), 14.01 (CH_3_). HRMS-(ESI-TOF) *m*/*z* [M + H]^+^ calcd. for C_51_H_43_N_2_O_11_: 859.2867. Found: 859.2833. *m*/*z* [M + NH_4_]^+^ calcd. for C_51_H_46_N_3_O_11_: 876.3132. Found: 876.3097.
3,4,5,6,7-Penta-*O*-benzoyl-2-deoxy-2-(*N*-ethylbenzamido)-d-*glycero*-d-*galacto*-heptononitrile (**20**) [51]. White solid, m. p. 166–168 °C, [α]_D_^20^ +6.9° (*c* 0.5, pyridine) [Lit. [51] m. p. 170 °C, [α]_D_^24^ +5.8° (*c* 0.37, pyridine)]; IR (KBr) ṽ_max_/cm^−1^ 2243 (C≡N), 1742, 1720, 1639 (C=O), 1264 (C-O-C), 1093, 1069, 1026 (C-O); Raman ṽ_max_/cm^−1^ 2250 (C≡N), 1746, 1639 (C=O); [α]_D_^22^ +4.2°; [α]_578_^22^ +4.2°; [α]_546_^22^ +4.2°; [α]_436_^22^ +4.6° (*c* 0.5, chloroform); ^1^H-NMR (500 MHz, CDCl_3_) δ 8.06–7.88 (m, 10H, H-arom), 7.60–7.24 (m, 20H, H-arom), 6.40 (bs, 2H, H-2, H-5), 6.07 (t, *J*_3,4_ = 5.5 Hz, 1H, H-4), 5.98 (dd, *J*_2,3_ = 2.5 Hz, *J*_3,4_ = 5.5 Hz,1H, H-3), 5.85 (ddd, *J*_6,7_ = 3.0 Hz, *J*_6,7′_ = 5.0 Hz, *J*_5,6_ = 8.0 Hz, 1H, H-6), 4.79 (dd, *J*_6,7_ = 3.0 Hz, *J*_7,7′_ = 12.5 Hz, 1H, H-7), 4.48 (dd, *J*_6,7′_ = 5.0 Hz, *J*_7,7′_ = 12.5 Hz, 1H, H-7′), 3.61 (m, 1H, CH_2_), 3.48 (m, 1H, CH_2_), 1.10 (t, *J* = 7.5 Hz, 3H, CH_3_); ^13^C{^1^H} NMR (125 MHz, CDCl_3_) 171.95, 166.03, 165.52, 165.33, 165.27, 165.09 (C=O),134.95, 133.69, 133.62, 133.58, 133.23, 133.02, 130.11, 129.98, 129.90, 129.84, 129.42, 129.11, 128.97, 128.76, 128.63, 128.58, 128.52, 128.43, 128.30, 126.49 (C-arom), 114.80 (C≡N), 70.69, 69.43, 69.02, 68.37 (C-3, C-4, C-5, C-6), 62.61 (C-7), 47.38 (C-2), 43.50 (CH_2_), 15.05 (CH_3_). HRMS-(ESI-TOF) *m*/*z* [M + H]^+^ calcd. for C_51_H_43_N_2_O_11_: 859.2822. Found: 859.2826. *m*/*z* [M + NH_4_]^+^ calcd. for C_51_H_46_N_3_O_11_: 876.3132. Found: 876.3081.
2-(Benzyloxycarbonyl)amino-2-deoxy-d-*glycero*-d-*ido*-heptononitrile (**26**) [52]. M. p. 200–202 °C (dec.), [α]_D_^22^ −25.4° (*c* 0.5, pyridine); IR (KBr) ṽ_max_/cm^−1^ 3440, 3400–3200 (OH), 3300 (NH), 2250 (C≡N), 1695 (C=O amide I), 1535 (C=O amide II), 1285 (C-O-C), 1085, 1050, 1040 (C-O); ^13^C{^1^H} NMR (50 MHz, DMSO-*d*_6_) δ 155.93 (C=O), 136.62, 128.52 (2C), 128.07, 127.94 (2C) (aromatics), 118.71 (C≡N), 72.13, 71.98, 71.38, 68.87, 66.17, 63.26 (C-7), 45.85 (C-2).
2-(*N*-Benzylamino)-2-deoxy-d-*glycero*-l-*gluco*-heptononitrile (**32**) [49]. M. p. 130–132 °C (dec.), [α]_D_^22^ −25.4° (*c* 0.5, pyridine); IR (KBr) ṽ_max_/cm^−1^ 3500–3100, 3020, 2230 (C≡N), 1610, 1500, 1430, 1090, 1050, 760, 700 (C-O); ^13^C{^1^H} NMR (50 MHz, DMSO-*d*_6_) δ 138.95, 128.62, 128.49, 127.27 (C-arom), 119.76 (C≡N), 72.56, 71.30, 70.78, 69.52 (C-3, C-4, C-5, C-6), 63.71 (C-7), 51.50 (CH_2_), 50.87 (C-2).
2-(*N*-Benzylamino)-2-deoxy-d-*glycero*-d-*ido*-heptononitrile (**33**) [49]. M. p. 200–202 °C (dec.), [α]_D_^22^ −81.6° (*c* 0.5, pyridine); IR (KBr) ṽ_max_/cm^−1^ 3400–3200, 2230 (C≡N), 1610, 1500, 740, 690; ^13^C{^1^H} NMR (50 MHz, DMSO-*d*_6_) δ 138.95, 128.62, 128.49, 127.27 (C-arom), 119.76 (C≡N), 72.56, 71.30, 70.78, 69.52 (C-3, C-4, C-5, C-6), 63.71 (C-7), 51.50 (CH_2_), 50.87 (C-2).
3,4,5,6,7-Penta-*O*-acetyl-2-(*N*-benzylacetamido)-2-deoxy-d-*glycero*-l-*gluco*-heptononitrile (**37**). A suspension of 2-benzylamino-2-deoxy-d-*glycero*-l-*gluco*-heptononitrile (**32**) [49] (2.1 g, 7.1 mmol) in pyridine (10 mL) was treated with acetic anhydride (10 mL) and the mixture was kept for 24 h at room temperature. After this time, it was poured into ice–water, and a white solid was separated by stirring and scraping. It was filtered, washed with cold water, and dried under a vacuum on silica gel. Compound **37** was obtained as a colorless solid (3.6 g, 92%). Crystallized from ethanol, it formed colorless needles. M. p. 135–137 °C, [α]_D_^20^ −11.2° (*c* 2.0, pyridine) (Lit. [49] for **14**, m. p. 83–85 °C); IR (KBr) ṽ_max_/cm^−1^ 2247 (C≡N), 1754, 1672 (C=O), 1250, 1215 (C-O-C), 1033 (C-O); ^1^H NMR (500 MHz, CDCl_3_) δ 7.41 (m, 2H, H-arom), 7.34 (m, 1H, H-arom), 7.24 (d, 2H, H-arom), 5.58 (d, *J*_3,4_ = *J*_4,5_ = 9.5 Hz, 1H, H-4), 5.53 (d, *J*_2,3_ = *J*_3,4_ = 9.5 Hz, 1H, H-3), 5.38 (d, *J*_2,3_ = 9.5 Hz, 1H, H-2), 5.32 (dd, *J*_5,6_ = 2.0 Hz, *J*_4,5_ = 9.5 Hz, 1H, H-5), 5.23 (t, *J*_6,7_ = 5.5 Hz, 1H, H-6), 4.67 (d, *J* = 7.5 Hz, 1H, CH_2_), 4.44 (d, *J =* 8.0 Hz, 1H, CH_2_), 4.29 (dd, *J*_6,7_ = 5.5 Hz, *J*_7,7′_ = 12.0 Hz, 1H, H-7), 3.86 (dd, *J*_6,7′_ = 7.5 Hz, *J*_7,7′_ = 12.0 Hz, 1H, H-7′), 2.19, 2.17, 2.12, 2.11, 2.05, 1.99 (s, 18H, CH_3_); ^13^C{^1^H} NMR (125 MHz, CDCl_3_) δ 171.62, 170.43, 170.37, 169.83, 169.79, 169.42 (C=O), 135.36, 129.18, 128.10, 126.00 (C-arom), 114.32 (C≡N), 67.89, 67.83, 67.45, 66.92 (C-3, C-4, C-5, C-6), 61.89 (C-7), 51.78 (CH_2_), 46.81 (C-2), 21.81, 20.75, 20.63, 20.50 (CH_3_). Anal. Calcd. for C_26_H_32_N_2_O_11_: C, 56.93; H, 5.88; N, 5.11. Found: C, 56.84; H, 5.90; N, 5.23. HRMS-(ESI-TOF) *m*/*z* [M + H]^+^ calcd. for C_26_H_33_N_2_O_11_: 549.2084. Found: 549.2069. *m*/*z* [M + NH_4_]^+^ calcd. for C_26_H_36_N_3_O_11_: 566.2350. Found: 566.2330.
3,4,5,6,7-Penta-*O*-acetyl-2-(*N*-benzylacetamido)-2-deoxy-d-*glycero*-d-*ido*-heptononitrile (**38**). A suspension of 2-benzylamino-2-deoxy-d-*glycero*-d-*ido*-heptononitrile (**33**) [49] (0.50 g, 1.7 mmol) in pyridine (3.0 mL) was treated with acetic anhydride (2.5 mL) and the mixture was kept for 24 h at room temperature. After this time, it was poured into ice–water, and a white solid was separated by stirring and scraping. It was filtered, washed with cold water, and dried under a vacuum on silica gel. Compound **38** was obtained as an amorphous, colorless, partially melted solid (0.81 g, 87%). M. p. 42–46 °C, [α]_D_^20^ −10.0° (*c* 1.0, pyridine) (Lit. [49] for **15**, m. p. room temperature ~25 °C; [α]_D_^20^ −9.6° (*c* 1.27, pyridine)); IR (KBr) ṽ_max_/cm^−1^ 2240 (C≡N), 1750 (C=O acetate),1670 (C=O amide), 1375, 1210 (C-O-C), 1050 (C-O); ^1^H NMR (200 MHz, CDCl_3_) δ 7.42–7.23 (m, 5H, H-arom), 5.73 (dd, *J*_3,4_ = 7.0 Hz, *J*_4,5_ = 3.1 Hz, 1H, H-4 *E*-*syn*), 5.68 (m, 1H, H-3 *Z-syn*), 5.62 (d, *J*_2,3_ = 5.0 Hz, 1H, H-2 *Z-syn*), 5.50 (m, 2H, H-4, H-5 *Z-syn*), 5.23 (dd, *J*_2,3_ = 3.9 Hz, *J*_3,4_ = 7.3 Hz, 1H, H-3 *E*-*syn*), 5.19 (dd, *J*_4,5_ = 3.1 Hz, *J*_5,6_ = 7.8 Hz, 1H, H-5 *E*-*syn*), 5.02 (m, 2H, H-6 *E*-*syn* and *Z-syn*), 4.82 (d, *J* = 17.6 Hz, 1H, PhCH_2_ *Z-syn*), 4.58 (d, *J =* 17.6 Hz, 1H, PhCH_2_ *Z-syn*), 4.32 (dd, *J*_6,7_ = 4.2 Hz, *J*_7,7_ = 12.3 Hz, 1H, H-7 *E*-*syn*), 4.17 (m, 2H, H-7, H-7′ *Z-syn*), 4.16 (dd, *J*_6,7′_ = 2.1 Hz, *J*_7,7′_ = 12.3 Hz, 1H, H-7′ *E*-*syn*), 4.08 (d, *J* = 12.8 Hz, 1H, PhCH_2_ *E*-*syn*), 3.99 (d, *J*_2,3_ = 3.9 Hz, 1H, H-2 *E*-*syn*), 3.80 (d, *J =* 13.0 Hz, 1H, PhCH_2_ *E*-*syn*), 2.15, 2.13, 2.11, 2.10, 2.08, 2.06, 2.04, 1.99 (s, 36H, CH_3_); ^13^C{^1^H} NMR (50 MHz, CDCl_3_) δ 172.02 (C=O *Z-syn*), 170.63 (*Z-syn*), 170.33 (*E*-*syn*), 170.14 (*E*-*syn*), 169.66 (*Z-syn*), 169.61 (*E*-*syn*), 169.42 (*Z-syn*), 169.20 (*E*-*syn*) (C=O), 137.52 (*E*-*syn*), 135.49 (*Z-syn*), 129.03 (*Z-syn*), 128.60 (*E*-*syn*), 128.30 (*E*-*syn*), 127.91 (*Z-syn*), 127.76 (*Z-syn*), 126.03 (*Z-syn*) (C-arom), 116.96 (C≡N *E*-*syn*), 114.41 (C≡N *Z-syn*), 70.15 (*E*-*syn*), 69.27 (*Z-syn*), 68.91 (*Z-syn*), 68.63 (*Z-syn*), 68.08 (*E*-*syn*), 68.00 (*Z-syn*), 67.93 (*Z-syn*), 67.76 (*E*-*syn*) (C-3, C-4, C-5, C-6), 61.22 (C-7 *Z-syn*), 61.11 (C-7 *E*-*syn*), 52.08 (CH_2_ *Z-syn*), 51.43 (CH_2_ *E*-*syn*), 49.35 (C-2 *E*-*syn*), 46.94 (C-2 *Z-syn*), 21.76 (CH_3_ *Z-syn*), 20.62, 20.54, 20.46, 20.37, 20.31 (CH_3_). Anal. Calcd. for C_26_H_32_N_2_O_11_: C, 56.93; H, 5.88; N, 5.11. Found: C, 56.72; H, 5.67; N, 5.33.
2-Deoxy-2-phenylamino-d-glucononitrile (**41**). To a suspension of d-arabinose (10.0 g, 66.6 mmol) in ethanol (30.0 mL) and water (3.0 mL), aniline (8.0 mL, 87.6 mmol) was added and the mixture was refluxed until dissolution. The reddish solution was concentrated in vacuo to a thick oil, which was treated with ethyl ether. The ethereal layer was decanted and the treatment was repeated twice. The residue was dissolved in hot ethanol and crystallized into *N*-phenyl-d-arabinopyranosylamine. The solid was dissolved by heating and then cooled rapidly to room temperature, and then anhydrous hydrogen cyanide (7–8 mL) was added. After one hour at room temperature, it was stored in a refrigerator overnight. The resulting solid was filtered, washed with cold ethanol and diethyl ether, and dried under a vacuum over silica gel (9.75 g, 58%). An additional yield of crystals was obtained from the mother liquors (2.17 g, 13%) (total yield: 11.92 g, 71%). White solid, m. p. 150–152 °C; [α]_D_^18^ +129.2°; [α]_578_^18^ +133.9°; [α]_546_^18^ +156.0° (*c* 0.5, pyridine); IR (KBr) ṽ_max_/cm^−1^ 3500–3100 (OH, NH), 2229 (C≡N), 1603, 1499, 754, 693 (phenyl), 1142, 1103, 1053, 1034 (C-O, C-N); ^13^C{^1^H} NMR (125 MHz, DMSO-*d*_6_) δ 144.64 (C≡N), 70.78, 70.24, 68.97 (C-3, C-4, C-5), 63.36 (C-6), 52.86 (C-2).
3,4,5,6-Tetra-*O*-acetil-2-deoxy-2-phenylamino-d-glucononitrile (**42**). A suspension of 2-deoxy-2-phenylamino-d-glucononitrile (**41**) (0.50 g, 2.0 mmol) in pyridine (3.5 mL) was treated with acetic anhydride (2.8 mL) and the mixture was kept under agitation for 24 h at 0 °C. After this time, it was poured into ice–water and, by stirring and scraping, a white solid was separated. It was filtered, washed with cold water, and dried under a vacuum over silica gel (0.74 g, 89%). Crystallization from 96% ethanol gave rise to a white microcrystalline powder. M. p. 135–136 °C; [α]_D_^22^ +415.4°; [α]_578_^22^ +416.4°; [α]_546_^22^ +423.0°; [α]_436_^22^ +461.4° (*c* 0.5, chloroform); IR (KBr) ṽ_max_/cm^−1^ 3347 (NH), 2239 (C≡N), 1763, 1743 (C=O), 1215 (C-O-C), 1081, 1050 (C-O); Raman ṽ_max_/cm^−1^ 2236 (C≡N), 1737 (C=O); ^1^H NMR (500 MHz, CDCl_3_) δ 7.26 (m, 2H, H-arom), 6.91 (t, 1H, H-arom), 6.78 (d, 2H, H-arom), 5.57 (dd, *J*_3,4_ = 1.5 Hz, *J*_4,5_ = 9.0 Hz, 1H, H-4), 5.28 (ddd, *J*_5,6_ = 3.0 Hz, *J*_5,6′_ = 4.0 Hz, *J*_4,5_ = 9.0 Hz, 1H, H-5), 5.25 (dd, *J*_3,4_ = 2.0 Hz, *J*_2,3_ = 6.0 Hz, 1H, H-3), 4.72 (dd, *J*_2,3_ = 6.0 Hz, *J*_*NH*,2_ = 11.0 Hz, 1H, H-2), 4.59 (d, *J*_*NH*,2_ = 11.0 Hz, 1H, NH), 4.28 (dd, *J*_5,6_ = 2.5 Hz, *J*_6,6′_ = 12.5 Hz, 1H, H-6), 4.23 (dd, *J*_5,6′_ = 3.5 Hz, *J*_6,6′_ = 12.5 Hz, 1H, H-6′), 2.23, 2.19, 2.08, 2.05 (s, 12H, CH_3_); ^13^C{^1^H} NMR (125 MHz, CDCl_3_) δ 171.79, 170.52, 170.22, 169.63 (C=O), 144.08, 129.72, 121.02 (C-arom), 116.67 (C≡N), 114.87 (C-arom), 68.15, 67.67, 66.95 (C-3, C-4, C-5), 61.50 (C-6), 46.00 (C-2), 21.03, 20.70, 20.67, 20.58 (CH_3_). Anal. Calcd. for C_20_H_24_N_2_O_8_: C, 57.14; H, 5.75; N, 6.66. Found: C, 56.97; H, 6.06; N, 6.63. HRMS-(ESI-TOF) *m*/*z* [M + H]^+^ calcd. for C_20_H_25_N_2_O_8_: 421.1611. Found: 421.1600.
3,4,5,6-Tetra-*O*-acetyl-2-deoxy-2-(*N*-phenylacetamido)-d-glucononitrile (**43**). To a suspension of 3,4,5,6-tetra-*O*-acetyl-2-deoxy-2-phenylamino-d-glucononitrile (**42**) (0.2 g, 0.5 mmol) in isopropenyl acetate (5.0 mL), a catalytic amount of *p*-toluenesulfonic acid was added and the mixture was refluxed for 8 h. Upon cooling, a white solid was separated, which was filtered and dried under a vacuum over silica gel (0.18 g, 81%), and had an m. p. of 195–196 °C. [α]_D_^22^ −3.1°; [α]_578_^22^ −3.6°; [α]_546_^22^ +1.0°; [α]_436_^22^ +23.4° (*c* 0.5, chloroform); IR (KBr) ṽ_max_/cm^−1^ 2237 (C≡N), 1765, 1744, 1673 (C=O), 1280, 1262, 1224 (C-O-C), 1065, 1036 (C-O); Raman ṽ_max_/cm^−1^ 2243 (C≡N), 1751, 1671 (C=O); ^1^H NMR (500 MHz, CDCl_3_) δ 7.48 (m, 3H, H-arom), 7.33 (d, 2H, H-arom), 5.72 (d, *J*_2,3_ = 8.5 Hz, 1H, H-2), 5.67 (dd, *J*_3,4_ = 3.0 Hz, *J*_2,3_ = 8.5 Hz, 1H, H-4), 5.51 (dd, *J*_3,4_ = 3.0 Hz, *J*_4,5_ = 8.5 Hz, 1H, H-4), 5.09 (ddd, *J*_5,6_ = 3.5 Hz, *J*_5,6′_ = 5.5 Hz, *J*_4,5_ = 8.5 Hz, 1H, H-5), 4.26 (dd, *J*_5,6_ = 3.5 Hz, *J*_6,6′_ = 12.5 Hz, 1H, H-6), 4.04 (dd, *J*_5,6′_ = 5.5 Hz, *J*_6,6′_ = 12.5 Hz, 1H, H-6′), 2.15, 2.05, 1.84 (s, 15H, CH_3_); ^13^C{^1^H} NMR (125 MHz, CDCl_3_) δ 171.04, 170.53, 169.82, 169.63, 169.23 (C=O), 139.64, 130.20, 129.61, 128.93 (C-arom), 114.40 (C≡N), 68.90, 68.80, 68.48 (C-3, C-4, C-5), 61.55 (C-6), 49.14 (C-2), 22.54, 20.79, 20.67, 20.57, 20.54 (CH_3_). Anal. Calcd. for C_22_H_27_N_2_O_9_: C, 57.14; H, 5.62; N, 6.06. Found: C, 57.09; H, 5.76; N, 5.73. HRMS-(ESI-TOF) *m*/*z* [M + H]^+^ calcd. for C_22_H_27_N_2_O_9_: 463.1717. Found: 463.1709. *m*/*z* [M + NH_4_]^+^ calcd, for C_22_H_30_N_3_O_9_: 480.1982. Found: 480.1974.
2-Amino-2-deoxy-d-glucose Oxime Hydrochloride (**45**). This compound was synthesized following the procedure described by Restelli and Deulofeu [61]. IR (KBr) ṽ_max_/cm^−1^ 3500–2400 (OH, NH^+^), 1610 (C=N), 1530, 1410, 1100, 1080, 1040, 1010 (C-O), 750; ^1^H NMR (200 MHz, DMSO-*d*_6_) δ 11.38 (s, 1H, NOH), 8.00 (bs, 2H), 5.31 (bs, 1H), 4.68 (bs, 1H), 4.52 (bs, 1H), (OH, NH), 7.35 (d, *J*_2,3_ = 5.6 Hz, 1H, H-1), 3.86 (m, 2H), 3.47 (m, 5H), 3.25 (d, 1H); ^13^C{^1^H} NMR (50 MHz, DMSO-*d*_6_) δ 144.64 (C=N), 70.78, 70.24, 68.97 (C-3, C-4, C-5), 63.36 (C-6), 52.86 (C-2).
2-Acetamido-3,4,5,6-tetra-*O*-acetyl-2-deoxy-d-glucononitrile (**47**). This compound was synthesized by modifying the Restelli and Deulofeu procedure [61]. A suspension of **45** (1.0 g, 4.2 mmol) in pyridine (6.0 mL) was treated with acetic anhydride (6.0 mL). The mixture was stirred for 24 h at room temperature. After this time, it was poured into ice–water and extracted with dichloromethane (2 × 50 mL), and the organic phase was washed successively with 1*M* hydrochloric acid (2 × 50 mL), saturated sodium bicarbonate solution (2 × 50 mL), and distilled water, and dried with anhydrous MgSO_4_. The solvent was removed in vacuo to dryness and the resulting residue was crystallized from ethanol. After being stored in the refrigerator for several hours, it was filtered, washed with cold ethanol, and dried under a vacuum over silica gel (0.77 g, 43%). Colorless prisms from ethanol, m. p. 126–129 °C; [α]_D_ +18.0°; [α]_578_ +18.2°; [α]_546_ +21.4°; [α]_436_ +40.2° (*c* 0.5, chloroform) [Lit. [61] m. p. 126 °C; [α]_D_^20^ +20.5° (*c* 4.7, chloroform)]; IR (KBr) ṽ_max_/cm^−1^ 3237 (NH), 2243 (C≡N), 1753, 1730, 1675, 1645 (C=O), 1541 (NH), 1252, 1211 (C-O-C), 1079, 1065, 1051 (C-O); Raman ṽ_max_/cm^−1^ 2243 (C≡N), 1753, 1728, 1643 (C=O); ^1^H NMR (400 MHz, CDCl_3_) δ 6.69 (d, *J*_*NH*,2_ = 9.8 Hz, 1H, NH), 5.45 (dd, *J*_2,3_ = 6.8 Hz, *J*_*NH*,2_ = 9.6 Hz, 1H, H-2), 5.36 (dd, *J*_3,4_ = 1.8 Hz, *J*_4,5_ = 9.2 Hz, 1H, H-4), 5.19 (dt, *J*_5,6_ = 4.0 Hz, *J*_5,6′_ = 6.4 Hz, *J*_4,5_ = 9.2 Hz, 1H, H-5), 5.10 (dd, *J*_3,4_ = 1.6 Hz, *J*_2,3_ = 6.8 Hz, 1H, H-3), 4.24 (m, 1H, H-6), 4.23 (m, 1H, H-6′), 2.23, 2.15, 2.08, 2.07, 2.05 (s, 15H, CH_3_); ^13^C{^1^H} NMR (100 MHz, CDCl_3_) δ 172.31, 170.37, 169.54, 169.39, 169.20 (C=O), 115.77 (C≡N), 67.51, 67.30, 67.00 (C-3, C-4, C-5), 61.42 (C-6), 39.22 (C-2), 22.92, 21.03, 20.68, 20.61, 20.37 (CH_3_). HRMS-(ESI-TOF) *m*/*z* [M + H]^+^ calcd. for C_16_H_23_N_2_O_9_: 387.1404. Found: 387.1406. *m*/*z* [M + NH_4_]^+^ calcd. for C_16_H_26_N_3_O_9_: 404.1669. Found: 404.1675.
2-Amino-2-deoxy-d-*glycero*-l-*gluco*-heptose Oxime Hydrochloride (**49**). This compound was obtained in a similar way to that described for the preparation of **45**: through the treatment of **48** [67] with hydroxylamine. Crystallized from aqueous ethanol, it formed colorless prisms, m. p. 157–160 °C (dec.). The product was characterized through its NMR spectra, which were almost coincidental with those of **45**. ^1^H NMR (200 MHz, DMSO-*d*_6_) δ 11.44 (s, 1H, NOH), 8.16 (bs, 2H), 5.27 (bs, 1H), 4.56 (bs, 1H), 4.35 (bs, 1H), 3.72 (m, 1H) (OH, NH), 7.40 (d, *J*_2,3_ = 5.2 Hz, 1H, H-1), 3.92 (m, 2H), 3.50 (m, 6H); ^13^C{^1^H} NMR (50 MHz, DMSO-*d*_6_) δ 144.55 (C=N), 69.74, 69.28, 69.14, 68.97 (C-3, C-4, C-5, C-6), 63.20 (C-7), 52.98 (C-2).
2-Acetamido-3,4,5,6,7-penta-*O*-acetyl-2-deoxy-d-*glycero*-l-*gluco*-heptononitrile (**51**). 2-Amino-2-deoxy-d-*glycero*-l-*gluco*-heptose oxime hydrochloride (**49**) (0.15 g, 0.6 mmol) in pyridine (1.0 mL) was treated with acetic anhydride (1.1 mL). The mixture was stirred for 24 h at room temperature. After this time, it was poured into ice–water and a white solid was separated by stirring and scraping. It was filtered, washed with cold water, and dried under a vacuum over silica gel (11.6%). The aqueous phase was extracted with dichloromethane (3 × 10 mL) and the organic phase was washed successively with 1*M* hydrochloric acid (2 × 10 mL), saturated sodium bicarbonate solution (2 × 10 mL), and distilled water, and dried with anhydrous MgSO_4_. The solvent was removed in vacuo to dryness and the resulting residue was crystallized from ethanol. After being stored in the refrigerator for several hours, it was filtered, washed with cold ethanol, and dried under a vacuum over silica gel (8.1%) (total yield 19.7%). Crystallized from ethanol, it formed colorless prisms, m. p. 154–156 °C; [α]_D_ +22.8°; [α]_578_ +24.0°; [α]_546_ +25.5°; [α]_436_ +40.6° (*c* 0.5, chloroform); IR (KBr) ṽ_max_/cm^−1^ 3278 (NH), 2251 (C≡N), 1743, 1690, 1674 (C=O), 1274, 1222 (C-O-C), 1077, 1046, (C-O); Raman ṽ_max_/cm^−1^ 2245 (C≡N), 1734, 1679 (C=O); ^1^H NMR (500 MHz, CDCl_3_) δ 6.67 (d, *J*_*NH*,2_ = 9.5 Hz, 1H, NH), 5.47 (dd, *J*_2,3_ = 6.5 Hz, *J*_*NH*,2_ = 10.0 Hz, 1H, H-2), 5.44 (dd, *J*_3,4_ = 2.0 Hz, *J*_4,5_ = 10.0 Hz, 1H, H-4), 5.39 (ddd, *J*_5,6_ = 2.0 Hz, *J*_6,7_ = 5.0 Hz, *J*_6,7′_ = 7.5 Hz, 1H, H-6), 5.26 (dd, *J*_5,6_ = 1.5 Hz, *J*_4,5_ = 10.0 Hz, 1H, H-5), 4.91 (dd, *J*_3,4_ = 1.5 Hz, *J*_2,3_ = 6.5 Hz, 1H, H-3), 4.26 (dd, *J*_6,7_ = 5.0 Hz, *J*_7,7′_ = 12.0 Hz, 1H, H-7), 3.85 (dd, *J*_6,7′_ = 7.5 Hz, *J*_7,7′_ = 11.5 Hz, 1H, H-7′), 2.23, 2.17, 2.10, 2.09, 2.07, 2.02 (s, 18H, CH_3_); ^13^C{^1^H} NMR (125 MHz, CDCl_3_) δ 173.01, 170.31, 170.06, 169.61, 169.49, 169.07 (C=O), 115.75 (C≡N), 67.50, 67.09, 66.71, 66.50 (C-3, C-4, C-5, C-6), 61.90 (C-7), 38.97 (C-2), 22.95, 21.13, 20.62, 20.57, 20.45 (CH_3_). HRMS-(ESI-TOF) *m*/*z* [M + H]^+^ calcd. for C_19_H_27_N_2_O_11_: 459.1615. Found: 459.1629.
2-Deoxy-2-(*N*-isopropylamino)-d-*glycero*-l-*gluco*-heptononitrile (**54**) [50]. White solid crystallized from ethanol, m. p. 130–132 °C (dec.); [α]_D_ −13.0° (*c* 0.5, pyridine); IR (KBr) ṽ_max_/cm^−1^ 3500–3200 (OH, NH), 2210 (C≡N), 1280, 1090, 1040, 1020 (C-O); ^13^C{^1^H} NMR (50 MHz, DMSO-*d*_6_) δ 120.99 (C≡N), 71.69, 70.73 (2C), 69.83 (C-3, C-4, C-5, C-6), 63.95 (C-7), 52.16 (CH, isopropyl), 47.82 (C-2), 23.90, 22.47 (CH_3_).
2-Deoxy-2-(*N*-isopropylamino)-d-*glycero*-d-*ido*-heptononitrile (**55**) and 2-deoxy-2- (*N*-isopropylamino)-d-*glycero*-d-*gulo*-heptononitrile (**60**) [50]. White solid crystallized from methanol, m. p. 132–134 °C (dec.). Spectroscopic data of **55**: ^13^C{^1^H} NMR (50 MHz, DMSO-*d*_6_) δ 120.29 (C≡N), 72.75, 71.20, 70.04, 69.63 (C-3, C-4, C-5, C-6), 63.67 (C-7), 49.16 (C-2), 46.56 (CH, isopropyl), 23.40, 21.11 (CH_3_, isopropyl). Spectroscopic data of **60**: ^13^C{^1^H} NMR (50 MHz, DMSO-*d*_6_) 119.98 (C≡N), 71.81, 71.33, 69.63, 69.23 (C-3, C-4, C-5, C-6), 63.45 (C-7), 50.90 (C-2), 46.25 (CH isopr.), 23.57, 21.49 (CH_3_, isopropyl).
*N*-Isopropyl-β-d-mannopyranosylamine (**56**). To a suspension of d-mannose (5.0 g, 27.7 mmol) in methanol (50 mL), isopropylamine (5.2 mL, 60.0 mmol) was added and the mixture was stirred and heated until dissolution. The solvent was partially evaporated, which often results in crystal formation. When the latter did not occur, the mixture was concentrated and the residue was solidified by triturating it with diethyl ether. The white solid was filtered, washed with ether, and dried in vacuo (5.96 g, 97%). Crystallized from absolute ethanol–diethyl ether, it showed an m. p. of 108–109 °C (dec.); [α]_D_^20^ −21° (*c* 0.5, NH_4_OH-H_2_O 1:9); [α]_D_^20^ −17° (*c* 0.5, HCl 2.5 *M*). IR (KBr) ṽ_max_/cm^−1^ 3500–3000 (OH), 3280 (NH), 1075, 1045, 1025 (C-O). Anal. Calcd. for C_9_H_19_NO_5_: C, 48.86; H, 8.64; N, 6.33. Found: C, 48.66; H, 8.50; N, 6.11.
d-*glycero*-d-*galacto*-Heptononitrile (**57**) [99]. Method (a): To a suspension of d-mannose (5.4 g, 30.0 mmol) in ethanol (50 mL), isopropylamine (5.2 mL, 60.0 mmol) was added and the mixture was heated until dissolution. After cooling to room temperature, anhydrous hydrogen cyanide (3.0 mL, 80 mmol) was added. A white crystalline solid separated, which was filtered, washed with cold ethanol and diethyl ether, and dried in vacuo (1.1 g, 17%). Crystallized from methanol, it showed an m. p. of 112–113 °C (dec.). Method (b): To a solution of the *N*-isopropyl-β-*d*-mannopyranosylamine (8.0 g, 36.0 mmol) in ethanol (32 mL), anhydrous hydrogen cyanide (2.8 mL, 74.6 mmol) was added. The reaction mixture was kept overnight in the refrigerator. The resulting white crystalline solid was filtered, washed with cold ethanol and diethyl ether, dried in vacuo (3.4 g, 45%), and recrystallized from methanol. The white solid had an m. p. of 113–115 °C (dec.); [α]_D_^20^ +10.4° (*c*. 0.5, pyridine) [Lit. [99] m. p. 122 °C; [α]_D_^20^ +10.5° (*c*. 0.5, pyridine)]. IR (KBr) ṽ_max_/cm^−1^ 3500–3100 (OH), 2220 (C≡N), 1400, 1070, 1000 (C-O); ^13^C{^1^H} NMR (125 MHz, DMSO-*d*_6_) δ 171.72, 170.26, 170.19, 170.04, 169.47 (C=O), 117.87 (C≡N), 68.45, 67.23, 67.18, 65.93 (C-3, C-4, C-5, C-6), 61.79 (C-7), 47.17 (C-2).
3,4,5,6,7-Penta-*O*-acetyl-2-deoxy-2-(*N*-isopropylamino)-d-*glycero*-l-*gluco*-heptononitrile (**58**). To a suspension of 2-deoxy-2-(*N*-isopropylamino)-d-*glycero*-l-*gluco*-heptononitrile (**54**) (0.25 g, 1.0 mmol) in pyridine (1.7 mL), acetic anhydride (1.4 mL) was added and the mixture was kept under stirring until dissolution, and then at 0 °C for 24 h. After this time, the solution was poured onto ice–water and a white solid was separated, which was filtered and washed with cold water (0.38 g, 64%). Crystallized from 96% ethanol, the white solid presented an m. p. of 125–127 °C; [α]_D_^22^ +17.2° (*c* 1.5, pyridine). IR (KBr) ṽ_max_/cm^−1^ 3330 (NH), 2980, 1740 (C=O), 1365, 1210 (C-O-C), 1040 (C-O); ^1^H NMR (200 MHz, CDCl_3_) δ 5.42–5.34 (m, 3H, H-4, H-5, H-6), 4.90 (d, *J*_2,3_ = 6.0 Hz, *J*_3,4_ = 0 Hz, 1H, H-3), 4.27 (dd, *J*_6,7_ = 5.0 Hz, *J*_7,7′_ = 11.7 Hz, 1H, H-7), 3.90 (d, *J*_2,3_ = 6.0 Hz, 1H, H-2), 3.86 (dd, *J*_6,7′_ = 7.2 Hz, *J*_7,7′_ = 11.7 Hz, 1H, H-7′), 2.97 (sep, *J* = 6.2 Hz, 1H, CH isopropyl), 2.18, 2.17, 2.11, 2.09, 2.02 (s, 15H, CH_3_), 1.13 (d, *J* = 6.2 Hz, 3H, CH_3_ isopropyl), 1.01 (d, *J* = 6.2 Hz, 3H, CH_3_ isopropyl); ^13^C{^1^H} NMR (50 MHz, CDCl_3_) δ 171.72, 170.26, 170.19, 170.04, 169.47 (C=O), 117.87 (C≡N), 68.45, 67.23, 67.18, 65.93 (C-3, C-4, C-5, C-6), 61.79 (C-7), 47.17 (C-2), 23.24, 21.15, 20.95, 20.53, 20.45, 20.36, 20.28 (CH_3_). Anal. Calcd. for C_20_H_30_N_2_O_10_: C, 52.40; H, 6.60; N, 6.11. Found: C, 52.21; H, 6.45; N, 5.90.
3,4,5,6,7-Penta-*O*-acetyl-2-deoxy-2-(*N*-isopropylamino)-d-*glycero*-d-*ido*-heptononitrile (**59***)* and 3,4,5,6,7-penta-*O*-acetyl-2-deoxy-2-(*N*-isopropylamino)-d-*glycero*-d-*gulo*- heptononitrile (**61**). White solid obtained from **55**/**60**, using the procedure described for **58**, as a mixture of **59** and **61** in a proportion of ~80:20. IR (KBr) ṽ_max_/cm^−1^ 3330 (NH), 2980, 1740 (C=O), 1365, 1210 (C-O-C), 1040 (C-O). Spectroscopic data of **59**: ^1^H NMR (200 MHz, CDCl_3_) δ 5.66 (dd, *J*_3,4_ = 6.7 Hz, *J*_4,5_ = 3.0 Hz, 1H, H-4), 5.37 (dd, *J*_4,5_ = 3.0 Hz, *J*_5,6_ = 7.7 Hz, 1H, H-5), 5.23 (dd, *J*_2,3_ = 3.8 Hz, *J*_3,4_ = 6.7 Hz, 1H, H-3), 4.99 (ddd, *J*_5,6_ = 7.7 Hz, *J*_6,7_ = 3.2 Hz, *J*_6,7′_ = 4.0 Hz, 1H, H-6), 4.23 (dd, *J*_6,7_ = 3.2 Hz, *J*_7,7″_ = 12.2 Hz, H-7), 4.13 (dd, *J*_6,7′_ = 4.0 Hz, *J*_7,7′_ = 12.2 Hz, 1H, H-7′), 4.10 (d, *J*_2,3_ = 3.8 Hz, 1H, H-2), 3.04 (sep, 1H, CH, isopropyl), 2.17, 2.14, 2.08, 2.05 (s, 15H, CH_3_, acetate), 1.17 (d, *J* = 6.2 Hz, 3H, CH_3_, isopropyl), 1.10 (d, *J* = 6.2 Hz, 3H, CH_3_, isopropyl); ^13^C{^1^H} NMR (50 MHz, CDCl_3_) δ 170.00, 169.93, 169.78, 169.24, 168.95 (C=O), 117.26 (C≡N), 70.30, 67.73 (3C) (C-3, C-4, C-5, C-6), 60.66 (C-7), 47.56 (C-2), 46.53 (CH, isopropyl), 23.15, 20.79 (CH_3_, isopropyl), 20.39, 20.22, 20.13, 20.03, 19.94 (CH_3_, acetate). Spectroscopic data of **61**: ^13^C{^1^H} NMR (50 MHz, CDCl_3_) δ 170.00, 169.93, 169.88, 169.61, 168.87 (C=O), 117.32 (C≡N), 69.21, 69.12, 68.45, 68.34 (C-3, C-4, C-5, C-6), 60.98 (C-7), 49.14 (C-2), 48.73 (CH, isopropyl), 22.97, 20.71 (CH_3_, isopropyl), 20.79, 20.71, 20.57, 20.22, 20.03 (CH_3_, acetate).
2,3,4,5,6,7-Hexa-*O*-acetyl-d-*glycero*-d-*galacto*-heptononitrile (**63**) [99]. To a stirred solution of d-*glycero*-d-*galacto*-heptononitrile (**57**) (1.0 g, 4.1 mmol) in pyridine (5 mL), cooled externally with an ice bath, acetic anhydride (4.0 mL, 27.1 mmol) was added gradually. The reaction mixture was kept overnight in the refrigerator and, subsequently, it was poured onto a water–ice mixture. The resulting solid was filtered, washed with water, and dried under a vacuum over silica gel (1.65 g, 53%). Crystallized from acetone–water, it showed an m. p. of 163–165 °C; [α]_D_^22^ +28.7°; [α]_578_ +29.3°; [α]_546_ +32.7°; [α]_436_ +55.3° (*c* 0.3, chloroform) (Lit. [99] m. p. 124–125 °C). ^1^H NMR (500 MHz, CDCl_3_) δ 5.52 (dd, *J*_3,4_ = 10.0 Hz, *J*_4,5_ = 2.0 Hz, 1H, H-4), 5.50 (dd, *J*_2,3_ = 3.0 Hz, *J*_3,4_ = 10.0 Hz, 1H, H-3), 5.38 (d, *J*_2,3_ = 3.0 Hz, 1H, H-2), 5.38 (dd, *J*_4,5_ = 2.0 Hz, *J*_5,6_ = 9.0 Hz, 1H, H-5), 5.02 (ddd, *J*_5,6_ = 9.0 Hz, *J*_6,7_ = 3.0 Hz, *J*_6,7′_ = 5.0 Hz, 1H, H-6), 4.20 (dd, *J*_6,7_ = 3.0 Hz, *J*_7,7″_ = 12.5 Hz, H-7), 4.00 (dd, *J*_6,7′_ = 5.0 Hz, *J*_7,7′_ = 12.5 Hz, 1H, H-7′), 2.17 (s, 6H, CH_3_ acetate), 2.10, 2.07, 2.05, 2.04 (s, 12H, CH_3_, acetate); ^13^C{^1^H} NMR (50 MHz, CDCl_3_) δ 170.48, 169.80, 169.56, 169.02, 168.76 (C=O), 113.92 (C≡N), 67.81 (C-6), 66.99, 66.89 (C-3,C-5), 66.48 (C-4), 61.75 (C-7), 59.40 (C-2), 20.80, 20.62, 20.48, 20.45, 20.11 (CH_3_, acetate). HRMS-(ESI-TOF) *m*/*z* [M + NH_4_]^+^ calcd. for C_19_H_29_N_2_O_12_: 477.1720. Found: 477.1730.
2,3,4,5,6,7-Hexa-*O*-benzoyl-d-*glycero*-d-*galacto*-heptononitrile (**64**) [99]. To a stirred solution of d-*glycero*-d-*galacto*-heptononitrile (1.0 g, 4.1 mmol) in pyridine (5 mL), cooled externally with an ice bath, benzoyl chloride (4.0 mL, 27.1 mmol) was added gradually. The reaction mixture was stored overnight in the refrigerator and, subsequently, it was poured onto a water–ice mixture and extracted with dichloromethane (3 × 20 mL). The organic layer was washed twice with 3*M* sulfuric acid; twice with saturated sodium bicarbonate solution; and finally with distilled water. The organic phase was dried with anhydrous sodium sulfate, filtered through activated charcoal, and evaporated to dryness. The residue was evaporated repeatedly with ethanol. The resulting crystalline solid was dried under a vacuum over calcium chloride (1.65 g, 41%). Crystallized from acetone–water, it showed an m. p. of 165 °C; [α]_D_^22^ +30.4° (c 2.0, chloroform) [Lit. [99] m. p. 161–162 °C; [α]_D_^22^ +30.3° (*c*. 2, chloroform)]. IR (KBr) ṽ_max_/cm^−1^ 1715, 1690 (C=O), 1590, 1570, 1435 (phenyl), 1240 (C-O-C), 1070, 1045 (C-O); ^1^H NMR (200 MHz, CDCl_3_) δ 8.04–7.87 (m, 12H, H-arom), 7.56–7.25 (m, 18H, H-arom), 6.31 (dd, *J*_3,4_ = 7.9 Hz, *J*_4,5_ = 2,1 Hz, 1H, H-4), 6.20 (d, *J*_4,5_ = 2.2 Hz, *J*_5,6_ = 7.8 Hz, 2H, H-5), 6.13 (dd, *J*_2,3_ = 4.2 Hz, *J*_3,4_= 7.8 Hz, 1H, H-3), 5.96 (d, *J*_2,3_ = 4.2 Hz, 1H, H-2), 5.79 (m, 1H, H-6), 4.85 (dd, *J*_6,7_ = 3.4 Hz, *J*_7,7′_ = 12.4 Hz, 1H, H-7), 4.45 (dd, *J*_6,7′_ = 5.4 Hz, *J*_7,7′_ = 12.4 Hz, 1H, H-7′); ^13^C{^1^H} NMR (50 MHz, CDCl_3_) δ 165.87, 165.21, 164.98, 164.81, 164.57, 164.18 (C=O),134.01, 133.79, 133.25, 133.05, 130.00, 129.91, 129.68, 129.27, 129.09, 128.73, 128.54, 128.49, 128.28, 127.42 (C-arom), 114.05 (C≡N), 69.39, 68.81, 68.22 (C-3,4,5), 62.35 (C-7), 60.67 (C-2).

## Figures and Tables

**Table 1:** Experimental and calculated geometrical parameters of **13**.

		Experimental ^a^	Gas Phase ^b^	CHCl_3_^b,c^	Solid ^b,d^	
Distances ^e^	C≡N	1.151	1.148	1.148	1.148	
Dihedral angle ^f^	H2 C2 C3 H3	184.78	171.87	174.93	176.89	
	H3 C3 C4 H4	47.57	47.046	49.46	51.47	
	H4 C4 C5 H5	181.08	179.62	177.10	177.92	
	H5 C5 C6 H6	302.17	308.77	306.44	312.33	
	H6 C6 C7 H7	56.22	77.60	74.47	69.85	
	H6 C6 C7 H7′	176.30	199.25	195.91	190.41	

^a^ X-ray data at 120 K; ^b^ M06-2X/6-311G(d,p); ^c^ SMD method; ^d^ calculated from X-ray data; ^e^ in Å; ^f^ in degrees.

**Table 2:** Steric, electronic, and conformational parameters for substituents at the nitrogen atom ^a^.

	H	Me	Et	^n^Pr	^i^Pr	^t^Bu	Bn	Ph
Es ^b^	1.24	0.00	−0.07	−0.36	−0.47	−1.54	−0.38	−2.55
υ ^c^	0.00	0.52	0.56	0.68	0.76	1.24	0.70	2.15
σ*	0.49	0.00	−0.10	−0.12	−0.19	−0.30	0.22	0.60
A	0.0	1.70	1.75		2.15	>4.5		3.0

^a^ Values taken from references [101,102,103,104]; ^b^ Taft steric parameter; ^c^ Charton steric parameter.

**Table 3:** Experimental and calculated geometrical parameters of **37**.

		Experimental ^a^	Gas Phase ^b^	CHCl_3_^b,c^	Solid ^b,d^
Distances ^e^	C≡N	1.16	1.15	1.15	1.15
	O-H3	2.64	2.34	2.39	2.35
Dihedral angle ^f^	H2 C2 C3 H3	188.88	172.08	174.36	170.29
	H3 C3 C4 H4	55.42	51.00	51.54	48.44
	H4 C4 C5 H5	180.71	180.49	180.62	180.69
	H5 C5 C6 H6	306.37	309.84	309.13	303.13
	H6 C6 C7 H7	62.97	69.22	67.18	64.70
	H6 C6 C7 H7′	181.68	189.80	188.28	186.57

^a^ X-ray data at 120 K; ^b^ M06-2X/6-311G(d,p); ^c^ SMD method; ^d^ calculated from X-ray data; ^e^ in Å; ^f^ in degrees.

**Table 4:** Experimental and calculated geometrical parameters of **42**.

		Experimental ^a^	Gas Phase ^b^	CHCl_3_^b,c^	Solid ^b,d^
Distances ^e^	C≡N	1.139	1.148	1.148	1.148
Dihedral angle ^f^	H2 C2 C3 H3	76.38	68.65	68.36	70.62
	H3 C3 C4 H4	309.63	301.92	302.275	308.06
	H4 C4 C5 H5	180.74	187.74	187.716	179.43
	H5 C5 C6 H6	295.71	291.05	290.322	286.47
	H5 C5 C6 H6′	54.48	171.24	170.367	47.67

^a^ X-ray data; ^b^ M06-2X/6-311G(d,p); ^c^ SMD method; ^d^ calculated from crystal data; ^e^ in Å; ^f^ in degrees.

**Table 5:** Experimental and calculated geometrical parameters of **47**.

		Experiment ^a^	Gas Phase ^b^	CHCl_3_^b,c^	Solid ^b,d^
Distances ^e^	C≡N	1.142	1.148	1.148	1.148
Dihedral angle ^f^	H2 C2 C3 H3	56.45	79.95	77.22	70.92
	H3 C3 C4 H4	287.50	310.82	308.16	300.03
	H4 C4 C5 H5	177.39	182.17	184.71	176.26
	H5 C5 C6 H6	293.47	291.08	290.60	289.48
	H5 C5 C6 H6′	53.04	170.54	170.21	50.72

^a^ X-ray data; ^b^ M06-2X/6-311G(d,p); ^c^ SMD method; ^d^ calculated from crystal data; ^e^ in Å; ^f^ in degrees.

**Table 6:** Relative electronic and Gibbs energies ^a,b^ for the conformers of compound **13**, along with their coupling constants determined in solution.

Rotamer	Gas Phase		Chloroform ^c^		Coupling Constants ^e^	
	ΔE	ΔG	ΔE	ΔG	*J*_a,b_	(Hz)
**A**_1_*G*^+^ *Z*	0.00	0.00	0.00	0.00	*J*_2,3_	10.0
**A**_1_*G*^+^ *E*	0.88	1.49	0.75	3.18	*J*_3,4_	1.5
**B**_1_*G*^+^ *Z*	2.88	3.45	0.97	1.69	*J*_4,5_	10.0
**B**_1_*G*^+^ *E*	3.79	5.12	3.71	5.21	*J*_5,6_	2.0
**A**_1_*G*^−^ *Z*	1.63	2.73	2.18	3.70	*J*_6,7_	5.5
**A**_1_*G*^−^ *E*	2.70	2.51	3.00	5.06	*J*_6,7′_	7.0
**B**_1_*G*^−^ *Z*	3.90	4.37	2.13	4.11	*J*_7,7′_	12.0
**B**_1_*G*^−^ *E*	7.18	7.13	7.28	9.90		
R-x ^d^	1.89	1.55				

^a^ In kcal·mol^−1^; ^b^ M06-2X/6-311G(d,p); ^c^ SMD method; ^d^ values calculated from experimental X-ray data; ^e^ calculated using ^1^H NMR spectroscopy (500 MHz, CDCl_3_).

**Table 7:** Relative electronic and Gibbs energies ^a,b^ for the conformers of **37** and their coupling constants in solution.

Conformer	Gas Phase		Chloroform ^c^		Coupling Constants ^e^	
	ΔE	ΔG	ΔE	ΔG	*J*_a,b_	(Hz)
**A**_1_*G*^+^ *Z*	0.00	0.00	0.00	0.00	*J*_2,3_	9.5
**A**_1_*G*^+^ *E*	1.31	0.91	1.85	2.40	*J*_3,4_	0.0
**B**_1_*G*^+^ *Z*	2.43	1.85	0.98	1.30	*J*_4,5_	9.5
**B**_1_*G*^+^ *E*	3.30	1.10	3.61	4.24	*J*_5,6_	2.0
**A**_1_*G*^−^ *Z*	−0.29	−0.16	1.02	1.50	*J*_6,7_	5.5
**A**_1_*G*^−^ *E*	1.40	2.66	2.46	3.14	*J*_6,7′_	7.5
**B**_1_*G*^−^ *Z*	1.34	−0.30	0.74	2.09	*J*_7,7′_	12.0
**B**_1_*G*^−^ *E*	3.66	4.21	5.47	8.68		
R-x ^d^	−1.19	−0.45				

^a^ In kcal.mol^−1^; ^b^ M06-2X/6-311G(d,p); ^c^ SMD method; ^d^ calculated from X-ray data; ^e^ calculated using ^1^H NMR spectroscopy (500 MHz, CDCl_3_).

**Table 8:** Relative electronic and Gibbs energies for the conformers of **42** ^a,b^.

Conformer	Gas Phase		Chloroform ^c^		Coupling Constants ^e^	
	ΔE	ΔG	ΔE	ΔG	*J*_a,b_	(Hz)
_1_ *G* ^+^_5_ *G* ^−^	0.00	0.00	0.00	0.00	*J*_2,3_	9.5
_1_ *G* ^−^_5_ *G* ^−^	3.64	2.93	2.38	2.40	*J*_3,4_	0.0
_5_ *G* ^−^	3.77	4.34	2.75	3.43	*J*_4,5_	9.5
R-x ^d^	2.74	2.46			*J*_5,6_	2.0

^a^ In kcal·mol^−1^; ^b^ M06-2X/6-311G(d,p); ^c^ SMD method; ^d^ calculated from X-ray data; ^e^ calculated using ^1^H NMR spectroscopy (500 MHz, CDCl_3_).

**Table 9:** Relative electronic and Gibbs energies for the conformers of **47** ^a,b^.

Conformer	Gas Phase		Chloroform ^c^		Coupling Constants ^e^	
	ΔE	ΔG	ΔE	ΔG	*J*_a,b_	(Hz)
_1_ *G* ^+^_5_ *G* ^−^	0.00	0.00	0.00	0.00	*J*_2,NH_	9.6
_1_ *G* ^−^_5_ *G* ^−^	5.78	6.71	3.18	3.37	*J*_2,3_	6.8
_5_ *G* ^−^	3.31	4.05	1.75	3.04	*J*_3,4_	1.8
R-x ^d^	0.79	0.17			*J*_4,5_	9.2

^a^ In kcal·mol^−1^; ^b^ M06-2X/6-311G(d,p); ^c^ SMD method; ^d^ calculated from X-ray data; ^e^ calculated using ^1^H NMR spectroscopy (500 MHz, CDCl_3_).

## Data Availability

Crystallographic data have been deposited in the Cambridge Structural Database (CSD) and can be obtained free of charge at www.ccdc.cam.ac.uk/data_request/cif (accessed on 15 March 2024), or via email at data_request@ccdc.cam.ac.uk, or by contacting the Cambridge Crystallographic Data Centre, 12 Union Road, Cambridge CB2 1EZ, UK. The corresponding registry numbers are indicated in the appropriate citations and correspond to the compounds mentioned throughout this manuscript.

## Supplementary Materials

The following supporting information can be downloaded at https://www.mdpi.com/article/10.3390/molecules29081796/s1: Crystal data, IR and NMR spectra, and computational data, as stated through the entire manuscript.
