# Supplementary material for: From Potential Prebiotic Synthons to Useful Chiral Scaffolds: A Synthetic and Structural Reinvestigation of 2-Amino-Aldononitriles
Source: Molecules. 2024 Apr 15;29(8):1796. doi: 10.3390/molecules29081796 (PMC11052109; doi:10.3390/molecules29081796)
Supplement: Supplementary file 1 [file molecules-29-01796-s001.zip › molecules-2942625-supplementary.pdf]

Supporting Information (SI)

2-Amino-aldononitriles: From Potential Prebiotic Synthons to Useful Chiral Scaffolds. A Synthetic and Structural Reinvestigation

Esther Matamoros[1,2,3,*], Mark E. Light[4], Pedro Cintas[1] and Juan C. Palacios[1,*]

[1] Departamento de Química Orgánica e Inorgánica, Facultad de Ciencias, and Instituto del Agua, Cambio Climático y Sostenibilidad (IACYS)-Unidad de Química Verde y Desarrollo Sostenible, Universidad de Extremadura, 06006 Badajoz, Spain
[2] Departamento de Química Orgánica, Universidad de Málaga, Campus Teatinos s/n, 29071 Málaga, Spain
[3] Instituto de Investigación Biomédica de Málaga y Plataforma en Nanomedicina – IBIMA, Plataforma Bionand, Parque Tecnológico de Andalucía, 29590 Málaga, Spain
[4] Department of Chemistry, Faculty of Engineering and Physical Sciences, University of Southampton, Southampton SO17 1BJ, UK
* Correspondence: esthermc@unex.es (E.M.); palacios@unex.es (J.C.P.)

ORCID

Esther Matamoros: 0000-0003-4460-2065

Pedro Cintas: 0000-0002-2608-3604

Mark E. Light: 0000-0002-0585-0843

Juan Carlos Palacios: 0000-0002-5004-4744

Summary

Pages	Supporting Data
2	Tables S1-S3
3	Crystallographic Data (Tables S4-S8)
9	IR Spectra. (Fig. S1-S18)
18	Raman Spectra (Fig. S19-S22)
19	NMR Spectra (Fig. S23-S92)
54	Mass Spectra (Fig. S93-S110)
63	Cartesian Coordinates and Calculated Energies at the M06-2X/6-311G(d,p) Level in Gas Phase and $CHCl_3$ (SMD Model).

Table S1. Selected IR and Raman data for some acyl aminonitriles in the solid state.[a,b]

Comp	$\bar{\nu}$ NH[b]	$\bar{\nu}$ C≡N[b]	$\bar{\nu}$ C≡N[c]	$\bar{\nu}$ C=O[b,d]	$\bar{\nu}$ C=O[c,d]	$\bar{\nu}$ C=O[b,e]	$\bar{\nu}$ C=O[c,e]	δNH[b,f]
**11**		2239	2239	1755	1736	1649	1645	
**13**		2255	2254	1749	1743	1662	1661	
**17**	3349	2229		1771, 1747				1509
**18**		g	2250	1730	1732	1655	1658	
**20**		g	2246	1742, 1720	1716	1639	1639	
**37**		2247		1754		1672		
**38**		2240[h]		1750		1670		
**42**	3347	2239	2236	1763, 1743	1737			
**43**		2237	2243	1765, 1744	1751	1673	1671	
**47**	3237	2243	2243	1753, 1730	1753, 1728	1675, 1645	1643	1541
**51**	3278	2251	2245	1743	1734	1690, 1674	1679	1503

[a]In cm^{-1}; [b]IR (KBr disc); [c]Raman; [d]Ester; [e]Amide band I; [f]Amide band II; [g]Not observed; [h]Imperceptible.

Table S2. ^{1}H NMR data (δ, ppm) for some acyl aminonitriles.[a]

Compo	H-2	H-3	H-4	H-5	H-6	H-6´	H-7	H-7´	NCH₂	NH
**11**	5.48 d	5.57 t	5.57 t	5.11 m[c]	4.27 dd	4.07dd	--	--	3.41 m	--
**12**[b]	5.34 d	5.60 t	5.60 t	5.14 m[c]	4.27 dd	4.07dd	--	--	3.25 m	--
**13**	5.38 d	5.57 d	5.44 dd	5.40 d	5.23 dd	--	4.28 dd	3.85 dd	3.39 m	--
**17**	4.52 dd	5.62 dd	5.46 dd	5.40 dd	5.30 m[c]	--	4.37 dd	4.10 dd	--	4.11 d
**18**	5.76 bs	6.12 m	6.12 m	6.04 m	5.91 bs	--	4.60 dd	4.48 dd	3.36 m[d]	--
**20**	5.98 dd	6.54 bs	6.07 t	6.54 bs	5.85 m[c]	--	4.79 dd	4.48 dd	3.55 m[e]	--
**37**	5.38 d	5.53 d	5.58 d	5.32 dd	5.23 m	--	4.29 dd	3.86 dd	4.58 m[f]	--
**38*Z***[i]	5.62 d	5.68 m	5.50 m	5.50 m	5.02 m		4.17 m	4.17 m	4.70 m[g]	
**38*E***[i]	3.99 d	5.23 dd	5.73 dd	5.19 dd	5.02 m		4.32 dd	4.16 dd	3.94 m[h]	
**42**	4.72 dd	5.25 dd	5.60 dd	5.28 m[c]	4.26 m	4.26 m				4.60 d
**43**	5.73 d	5.68 dd	5.53 dd	5.09 m[c]	4.27 dd	4.04dd				
**47**[b]	5.47 dd	5.14 dd	5.39 dd	5.19 dt	4.25 m	4.25 m				6.77 d
**51**	5.47 dd	4.91 dd	5.44 dd	5.26 dd	5.39 m[c]		4.26 dd	3.85 dd		6.67 d

[a]In $CDCl_3$ at 500 MHz; [b]In $CDCl_3$ at 400 MHz; [c]m = ddd; [d]m = 3.47 m + 3.25 m; [e]m = 3.60 m + 3.49 m; [f]m= 4.67 d + 4.44 d; [g]dd = 4.82 d + 4.58 d; [h]dd = 4.08 d + 3.80 d; [i]In $CDCl_3$ at 200 MHz.

Table S3. ^{13}C NMR data (δ, ppm) for some acyl aminonitriles.[a]

Compound	C≡N	C-2	C-6	C-7	NCH₂	NC=O	Me[d]
**11**	114.57	45.37	61.35		42.83		
**12**[b]	114.70	45.94	61.57		50.21		
**13**	114.70	45.18		61.74	42.91		
**17**	116.67	47.01		61.60			
**18**	114.62	47.16		62.56	42.73		
**20**	114.60	47.38		62.32	43.26		
**37**	114.32	46.81		61.89	51.78	171.62	21.81
**38*Z***[c]	116.94	49.35		61.26		172.06	21.76
**38*E***[c]	114.42	46.92		61.10			
**42**	116.54	45.78	61.27				
**43**	114.25	49.05	61.38			170.91	22.42
**47**[b]	115.73	39.23	61.38				
**51**	115.75	38.97		61.90		173.01	22.95

[a]In $CDCl_3$ at 125 MHz; [b]In $CDCl_3$ at 100 MHz; [c]In $CDCl_3$ at 50 MHz; [d]Acetamido group.

Table S4. Crystal data and structure refinement details of **13**

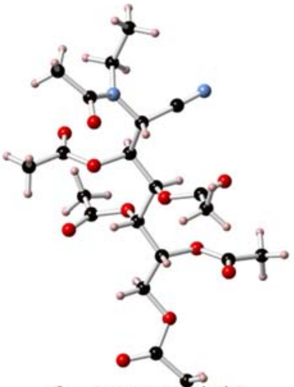

Identification code	**2011acc0771**
Empirical formula	$C_{21}H_{30}N_2O_{11}$
Formula weight	486.47
Temperature	100(2) K
Wavelength	0.71075 Å
Crystal system	Monoclinic
Space group	$P2_1$
Unit cell dimensions	a = 8.689(2) Å
	b = 12.613(3) Å β = 103.672(4)°
	c = 11.114(3) Å
Volume	1183.4(5) $Å^3$
Z	2
Density (calculated)	1.365 Mg / m^3
Absorption coefficient	0.111 mm^{-1}
$F(000)$	516
Crystal	Fragment; Colourless
Crystal size	0.20 × 0.10 × 0.03 mm^3
θ range for data collection	3.14 – 27.48°
Index ranges	$-11 \leq h \leq 6$, $-10 \leq k \leq 16$, $-14 \leq l \leq 14$
Reflections collected	6306
Independent reflections	2828 [R_{int} = 0.0245]
Completeness to θ = 27.48°	99.5 %
Absorption correction	Semi–empirical from equivalents
Max. and min. transmission	0.9967 and 0.9781
Refinement method	Full-matrix least-squares on F^2
Data / restraints / parameters	2828 / 1 / 314
Goodness-of-fit on F^2	1.038
Final R indices [$F^2 > 2\sigma(F^2)$]	$R1$ = 0.0379, $wR2$ = 0.0855
R indices (all data)	$R1$ = 0.0424, $wR2$ = 0.0877
Largest diff. peak and hole	0.278 and –0.179 e $Å^{-3}$

Table S5. Crystal data and structure refinement details of **37**

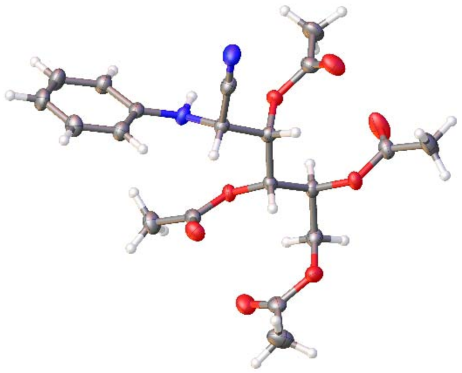

**Compound**	**2012acc0031**
Formula	$C_{26}H_{32}O_{11}N_2$
$D_{calc.}$/ g cm^{-3}	1.315
m/mm^{-1}	0.103
Formula Weight	548.53
Colour	clear colourless
Shape	needle-shaped
Size/mm^3	0.20×0.20×0.20
T/K	100(2)
Crystal System	monoclinic
Flack Parameter	-0.7(10)
Hooft Parameter	-1.8(10)
Space Group	*C*2
a/Å	19.892(11)
b/Å	6.009(3)
c/Å	23.888(12)
a/°	90
b/°	103.91(3)
g/°	90
V/Å^3	2772(3)
Z	4
Z'	1
Wavelength/Å	0.71073
Radiation type	Mo K_a
Q_{min}/°	2.954
Q_{max}/°	25.026
Measured Refl's.	10256
Indep't Refl's	4095
Refl's I≥2 *s*(I)	2395
R_{int}	0.0995
Parameters	377
Restraints	648
Largest Peak	0.307
Deepest Hole	-0.209
GooF	1.070
wR_2 (all data)	0.2155
wR_2	0.1730
R_1 (all data)	0.1520
R_1	0.0903

Table S6. Crystal data and structure refinement details of **42.**

Compound	2014acc0004
Formula	$C_{20}H_{24}N_2O_8$
$D_{calc.}$/ g cm^{-3}	1.292
μ/mm^{-1}	0.101
Formula Weight	420.41
Colour	clear colourless
Shape	prism
Max Size/mm	0.32
Mid Size/mm	0.28
Min Size/mm	0.20
T/K	120
Crystal System	monoclinic
Space Group	$P2_1$
a/Å	10.0041(8)
b/Å	10.3736(5)
c/Å	10.8552(7)
α/°	90
β/°	106.375(4)
γ/°	90
V/Å^3	1080.84(12)
Z	2
Z'	1.000
Θ_{min}/°	3.138
Θ_{max}/°	27.101
Measured Refl.	10823
Independent Refl.	4674
Reflections Used	4216
R_{int}	0.0315
Parameters	279
Restraints	1
Largest Peak	0.194
Deepest Hole	-0.245
GooF	1.167
wR_2 (all data)	0.1017
wR_2	0.0892
R_1 (all data)	0.0466
R_1	0.0391

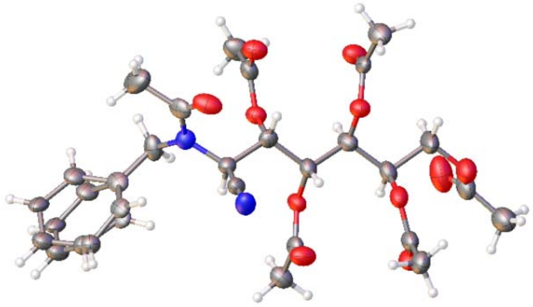

Table S7. Crystal data and structure refinement details of **47**

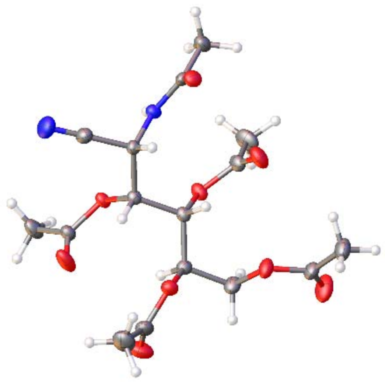

Identification code	adona_0m
Empirical formula	$C_{16}H_{22}N_2O_9$
Formula weight	386.36
Temperature/K	170(2)
Crystal system	Orthorhombic
Space group	$P2_12_12_1$
a/Å	8.3036(3)
b/Å	14.5478(5)
c/Å	15.9656(5)
α/°	90
β/°	90
γ/°	90
Volume/Å^3	1928.63(11)
Z	4
ρ_{calc}g/cm^3	1.331
μ/mm^{-1}	0.11
F(000)	816.0
Crystal size/mm^3	0.2 × 0.18 × 0.12
Radiation	MoKα (λ = 0.71073)
2Θ range for data collection/°	3.78 to 60.38
Index ranges	-11 ≤ h ≤ 11, -11 ≤ k ≤ 20, -22 ≤ l ≤ 18
Reflections collected	12536
Independent reflections	5693 [R_{int} = 0.0281, R_{sigma} = 0.0484]
Data/restraints/parameters	5693/0/250
Goodness-of-fit on F^2	0.804
Final R indexes [I>=2σ (I)]	R_1 = 0.0444, wR_2 = 0.1170
Final R indexes [all data]	R_1 = 0.0617, wR_2 = 0.1334
Largest diff. peak/hole / e Å^{-3}	0.25/-0.21

Table S8. Crystal data and structure refinement details of **63.**

**Compound**	**2015acc0013_K_100K**
Formula	$C_{19}H_{24}NO_{12}$
$D_{calc.}$/ g cm^{-3}	1.383
m/mm^{-1}	0.117
Formula Weight	458.39
Colour	clear colourless
Shape	block-shaped
Size/mm^3	0.13×0.05×0.02
T/K	100(2)
Crystal System	orthorhombic
Flack Parameter	-0.4(14)
Hooft Parameter	-0.4(14)
Space Group	$P2_12_12_1$
a/Å	8.6244(3)
b/Å	12.0277(4)
c/Å	21.2251(10)
a/°	90.00
b/°	90.00
g/°	90.00
V/Å^3	2201.73(15)
Z	4
Z'	1
Wavelength/Å	0.71073
Radiation type	Mo K_a
Q_{min}/°	3.04
Q_{max}/°	28.70
Measured Refl's.	12046
Indep't Refl's	5634
Refl's I≥2 s(I)	4355
R_{int}	0.0363
Parameters	313
Restraints	0
Largest Peak	0.445
Deepest Hole	-0.305
GooF	1.040
wR_2 (all data)	0.1707
wR_2	0.1575
R_1 (all data)	0.0883
R_1	0.0671

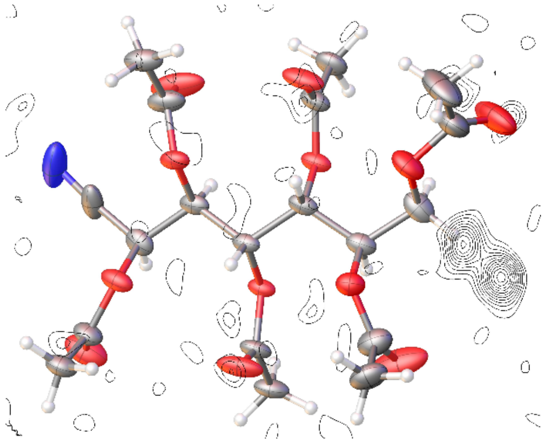

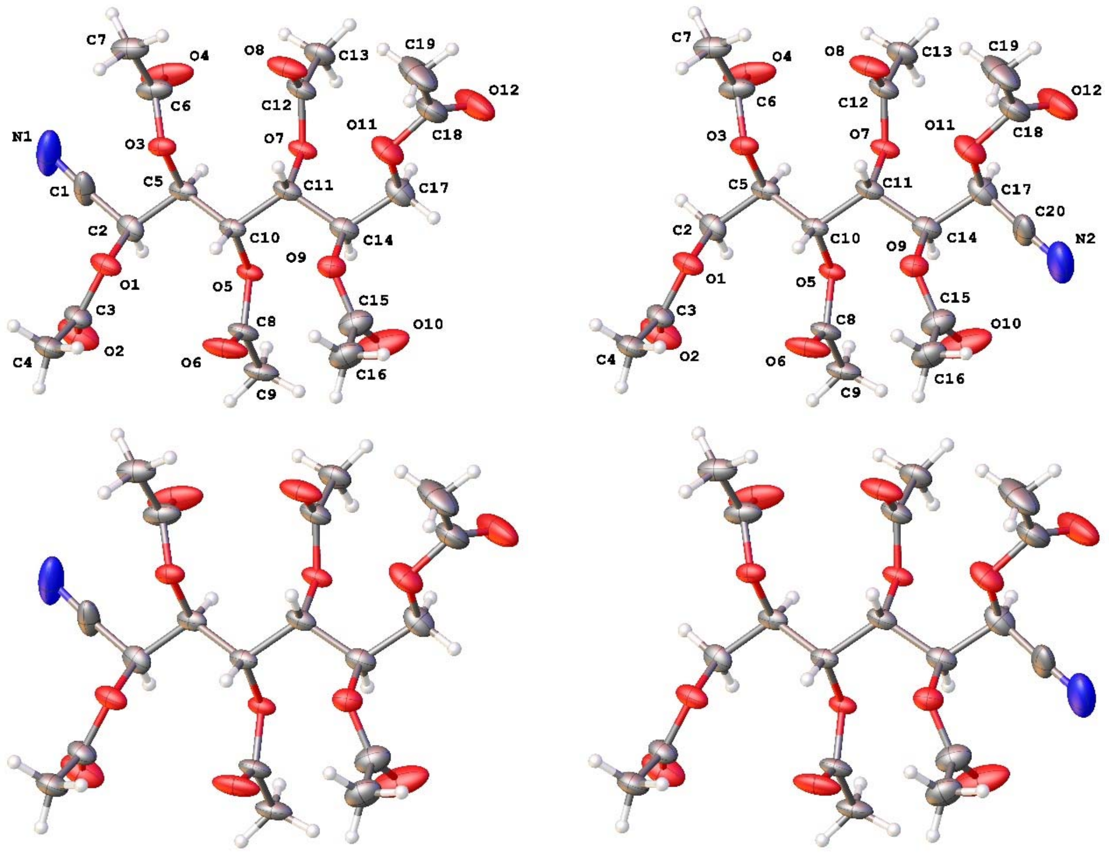

(a) (b)

Solid-state Orientations in the Crystal Structure for Compound 63: Major component (a) and minor component (b), thermal ellipsoids drawn at the 50% probability level.

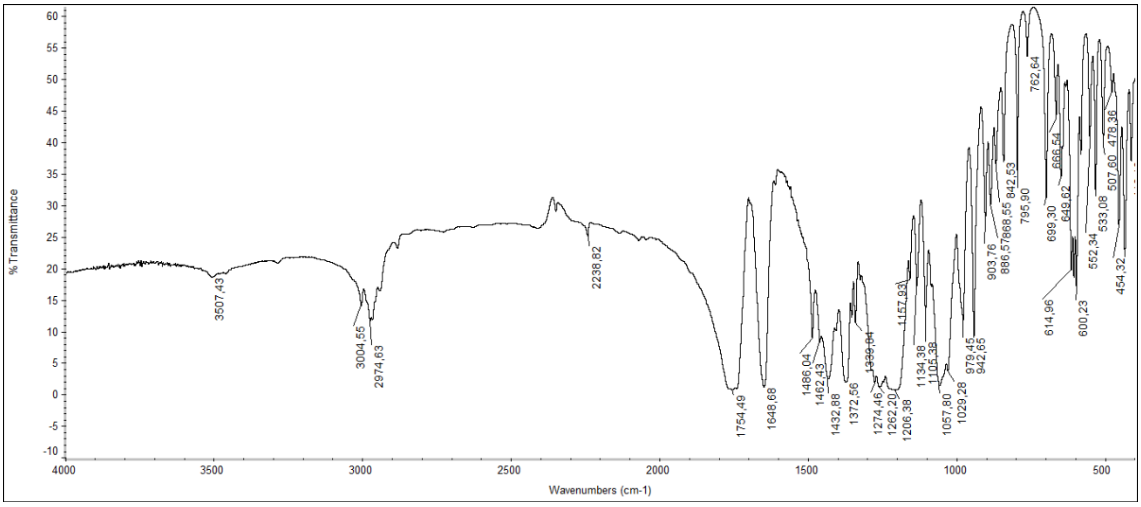

Figure S1. IR spectrum of **11**.

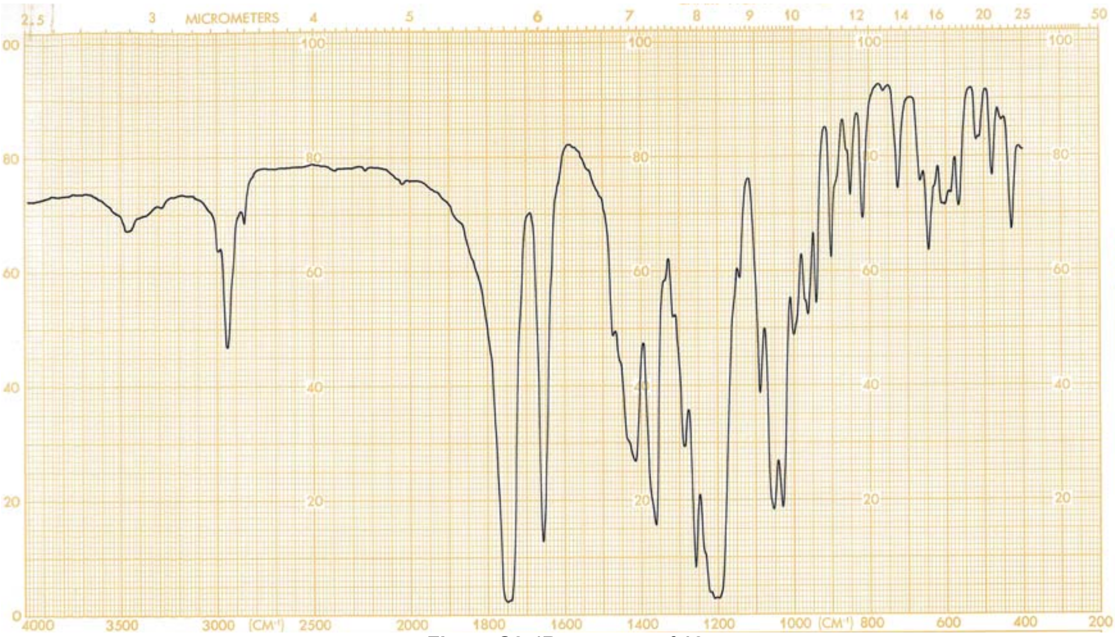

Figure S2. IR spectrum of **12**.

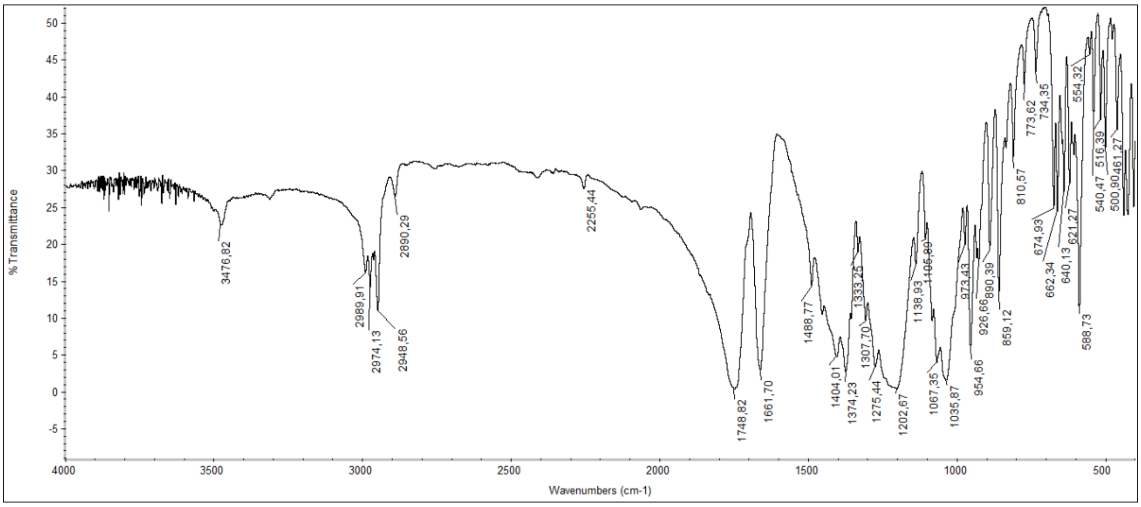

Figure S3. IR spectrum of **13**.

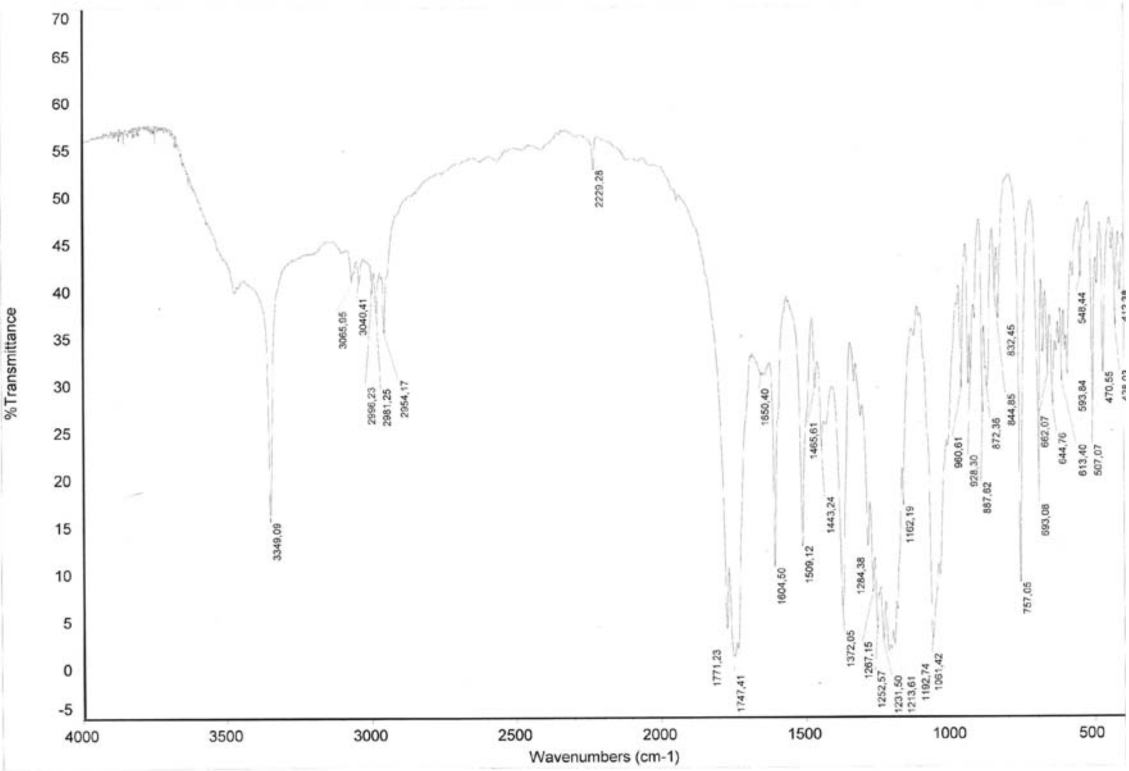

Figure S4. IR spectrum of **17**.

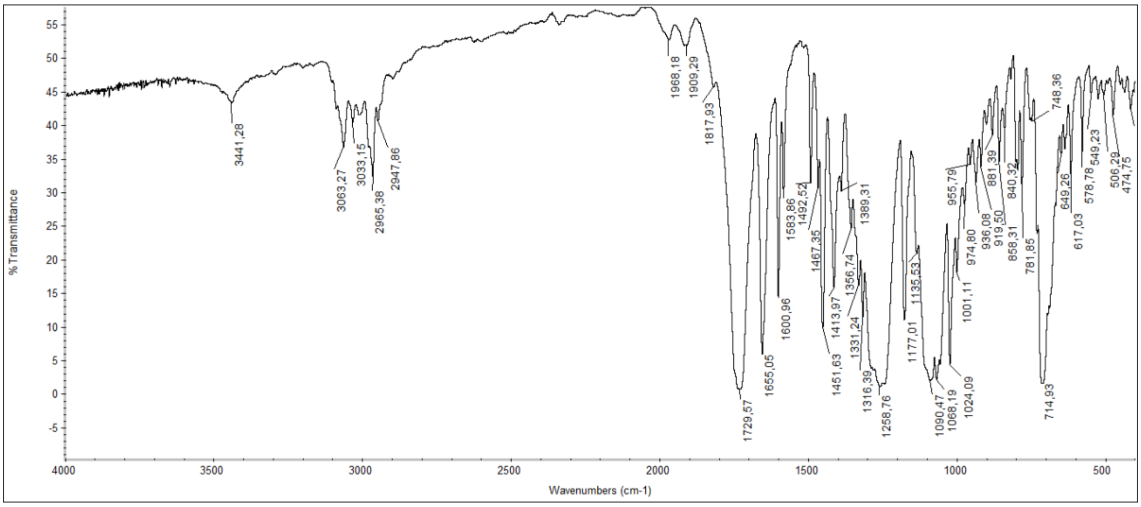

Figure S5. IR spectrum of **18**.

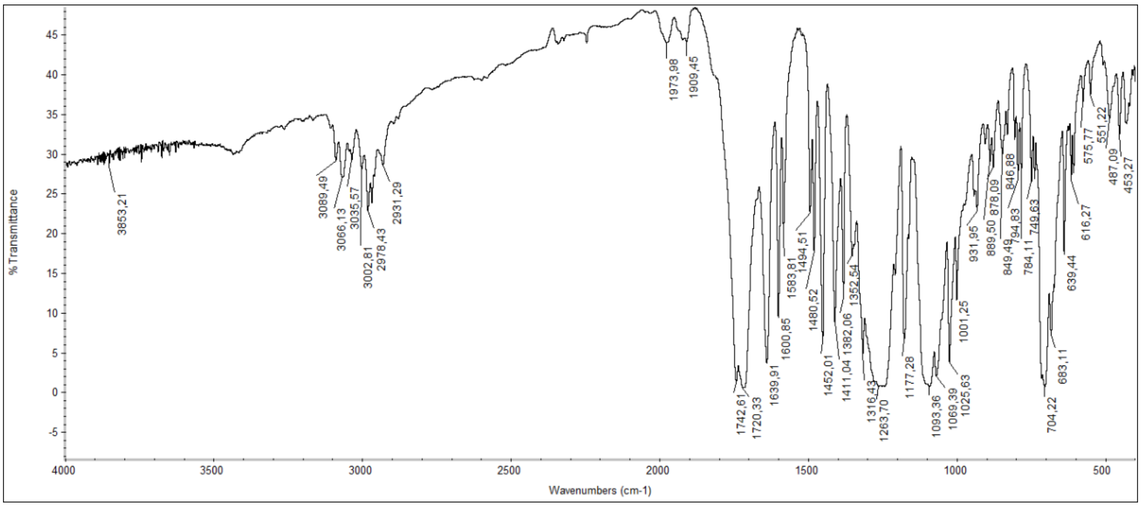

Figure S6. IR spectrum of **20**.

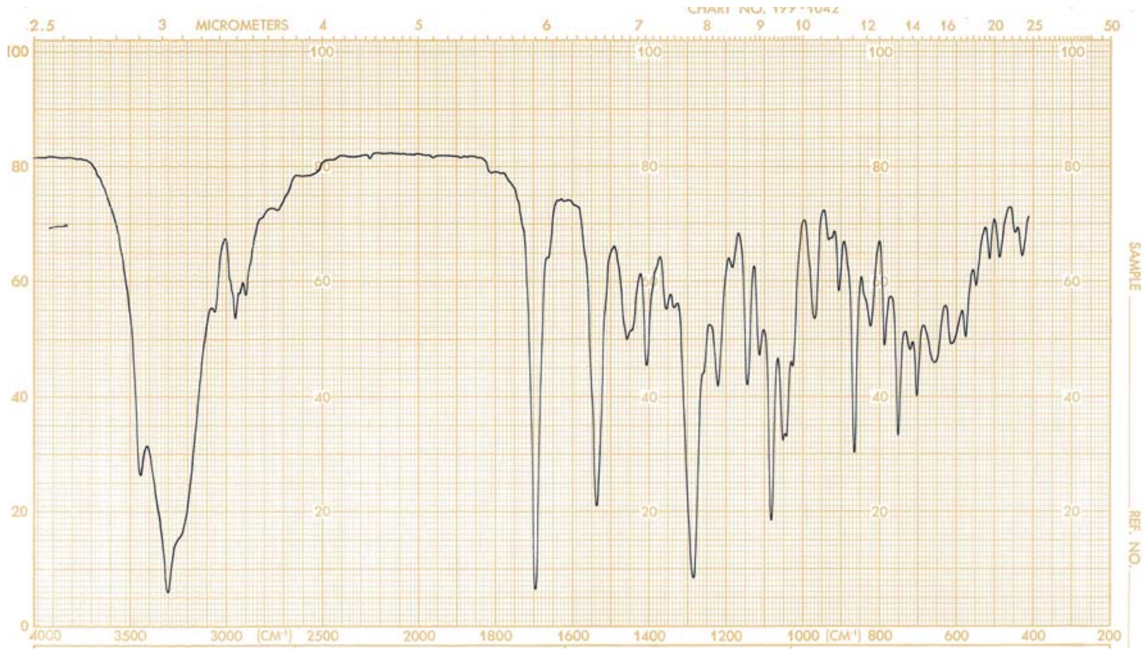

Figure S7. IR spectrum of **26.**

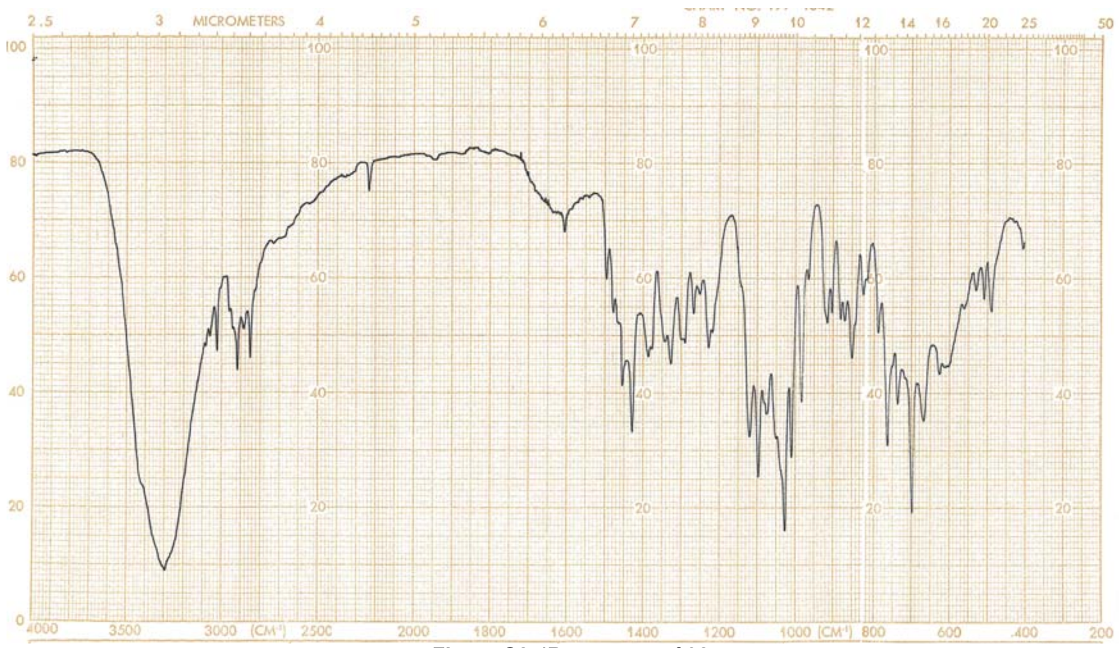

Figure S8. IR spectrum of **32.**

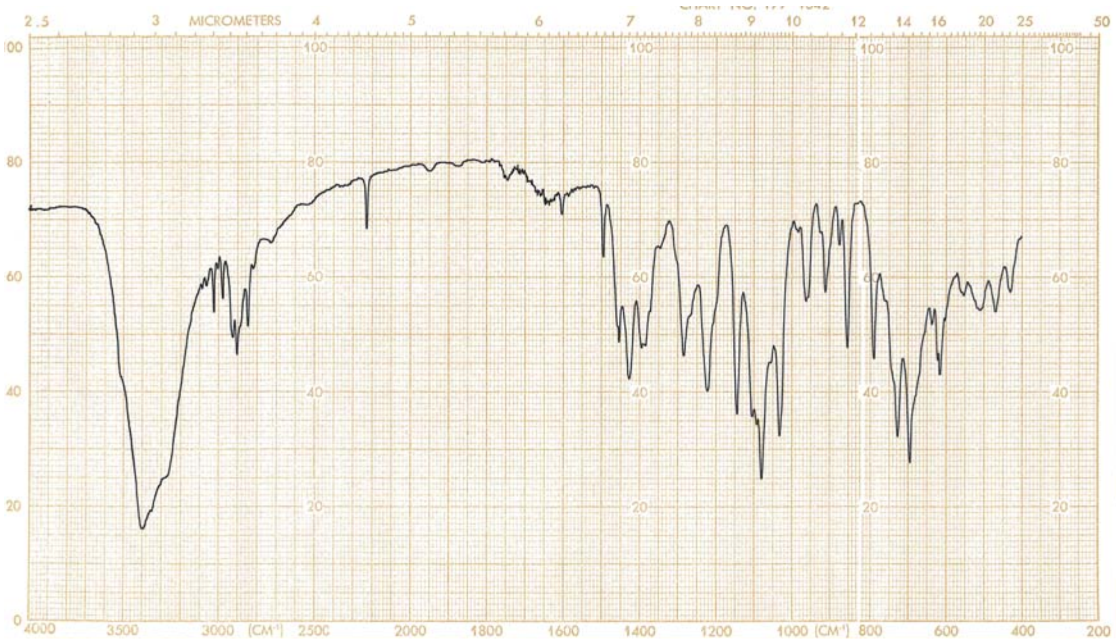

Figure S9. IR spectrum of **33**.

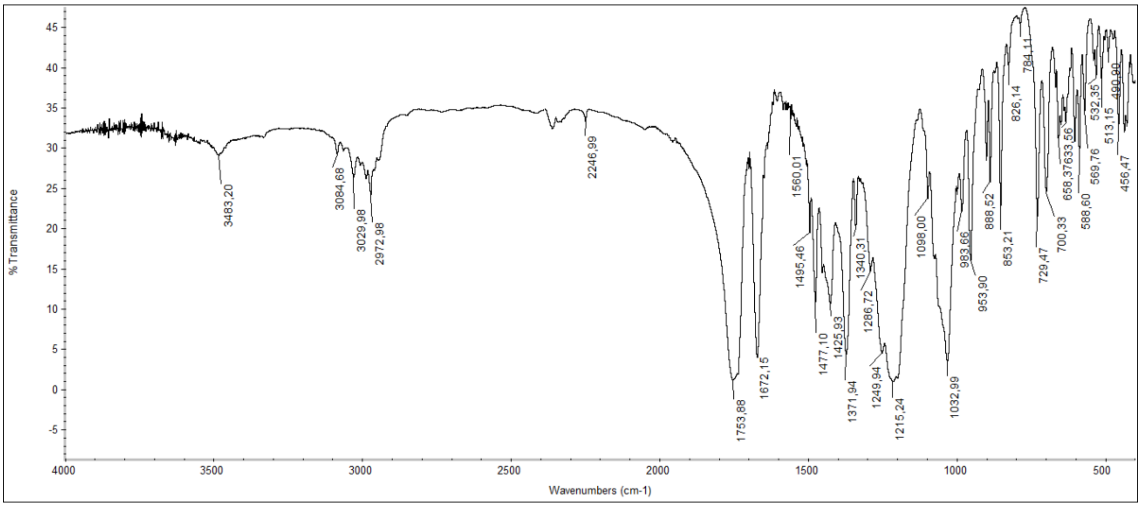

Figure S10. IR spectrum of **37**.

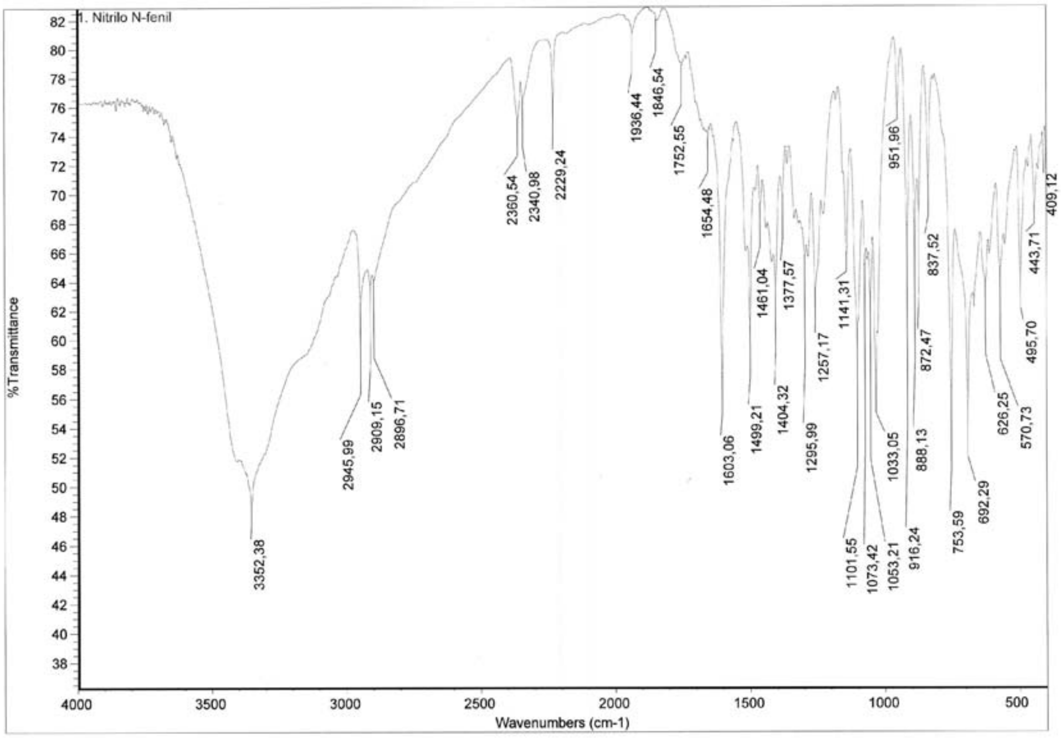

Figure S11. IR spectrum of **41**.

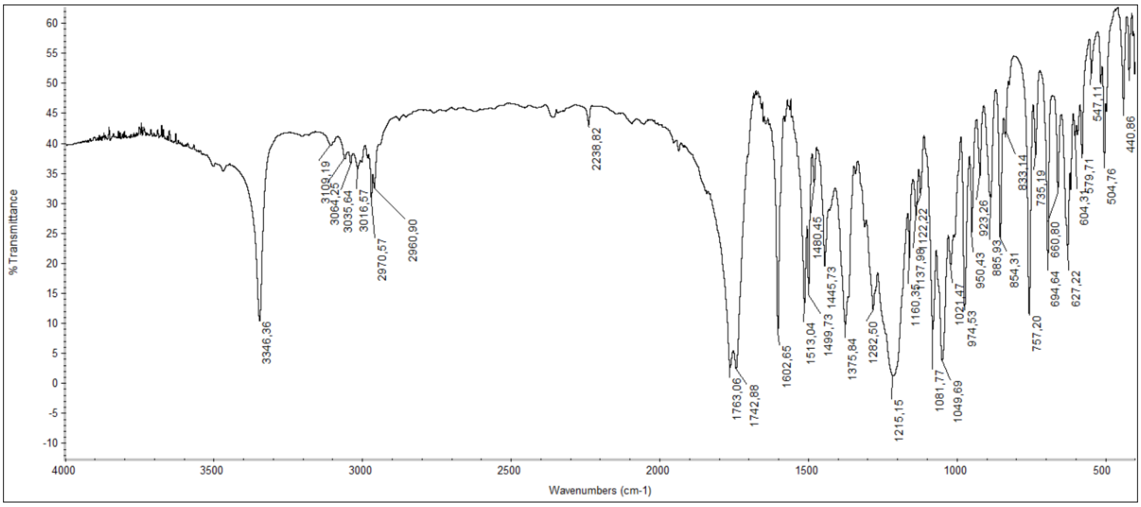

Figure S12. IR spectrum of **42**.

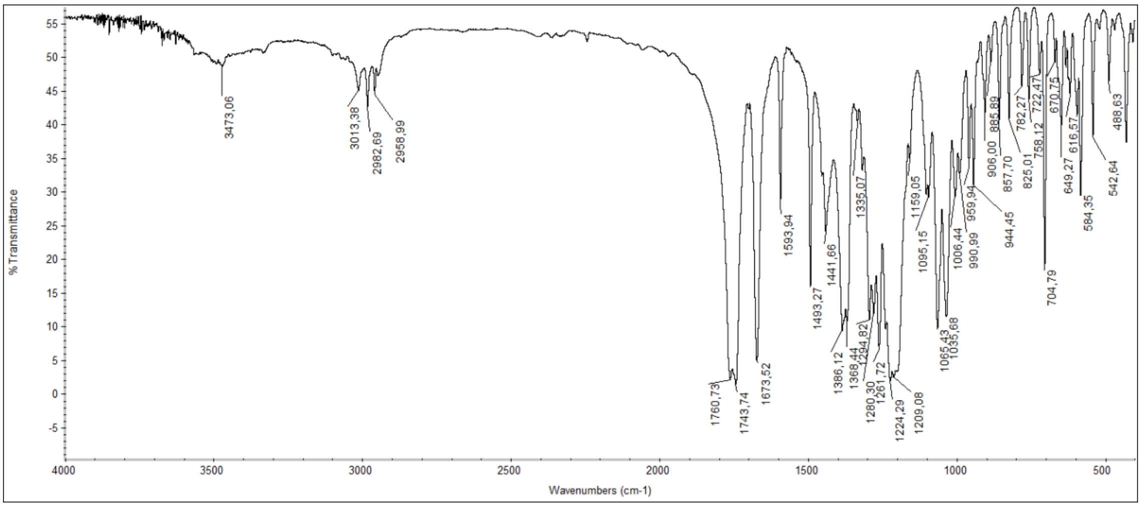

Figure S13. IR spectrum of **43.**

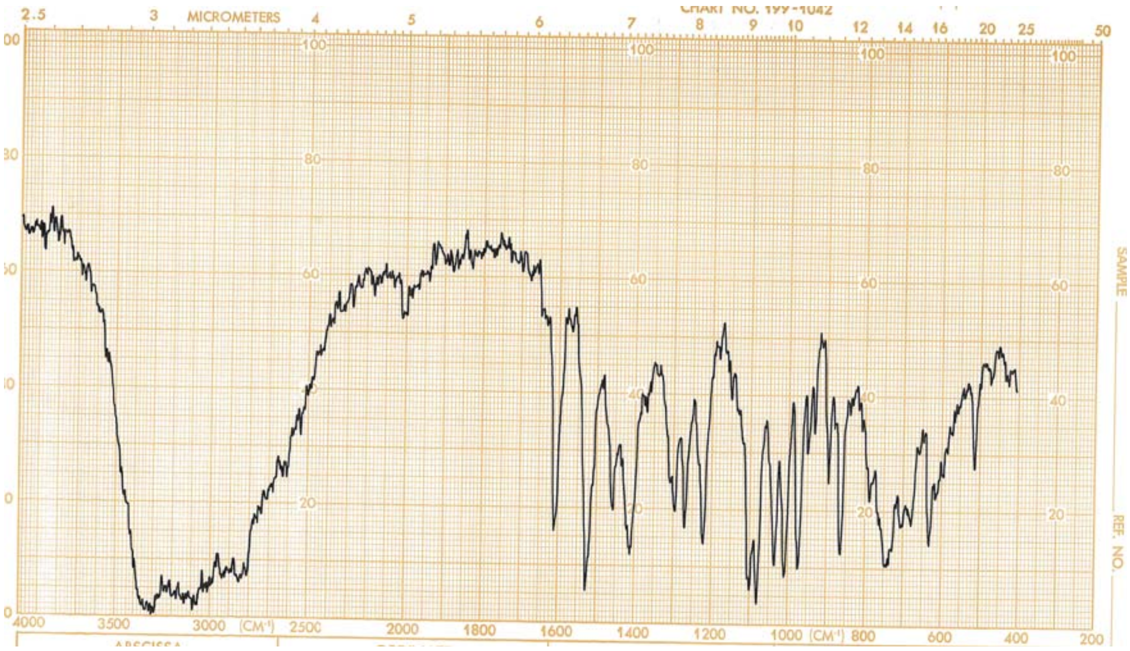

Figure S14. IR spectrum of **45.**

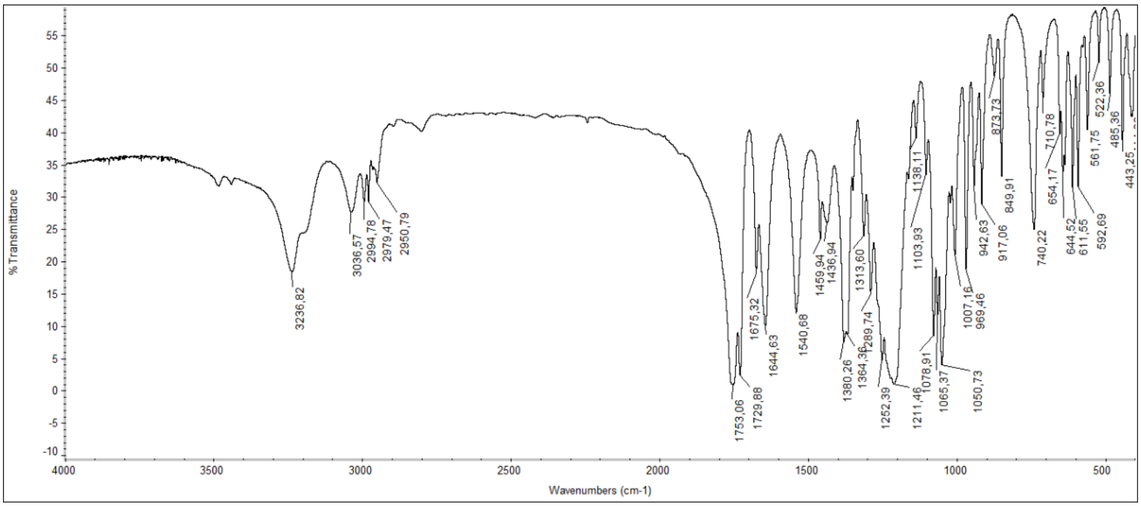

Figure S15. IR spectrum of **47**.

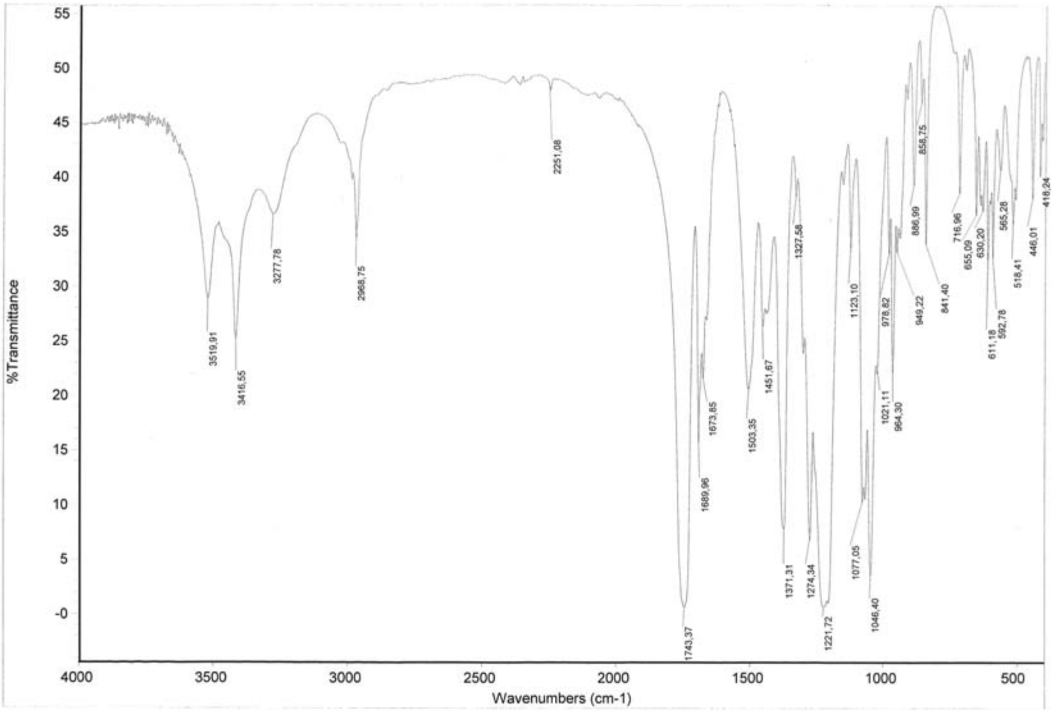

Figure S16. IR spectrum of **51**.

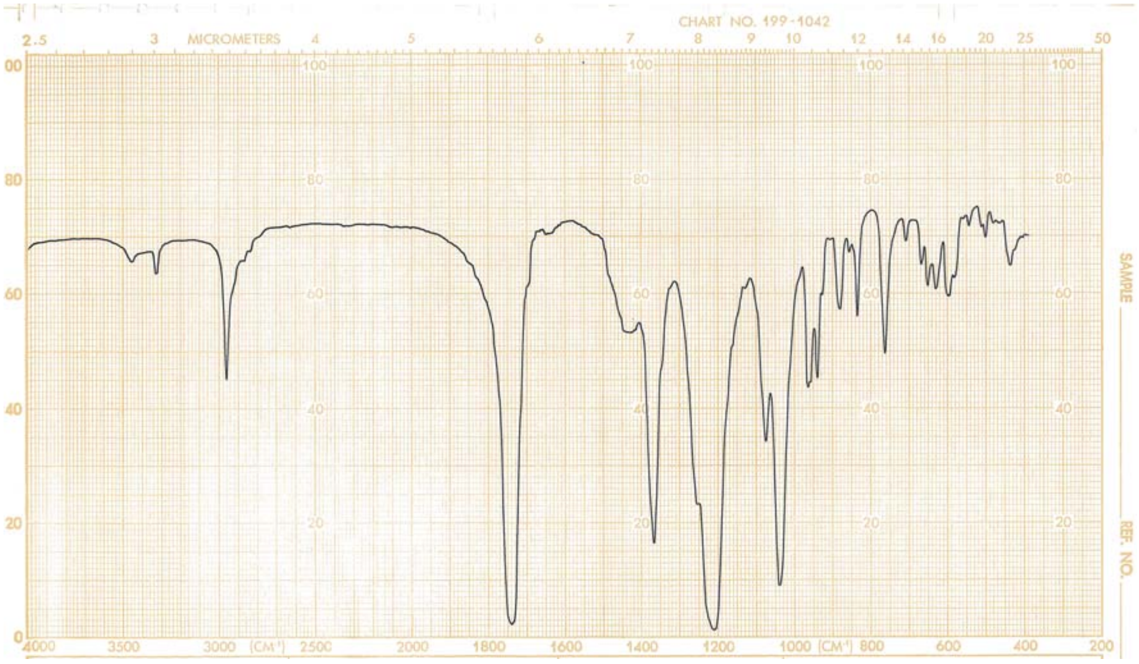

Figure S17. IR spectrum of **58.**

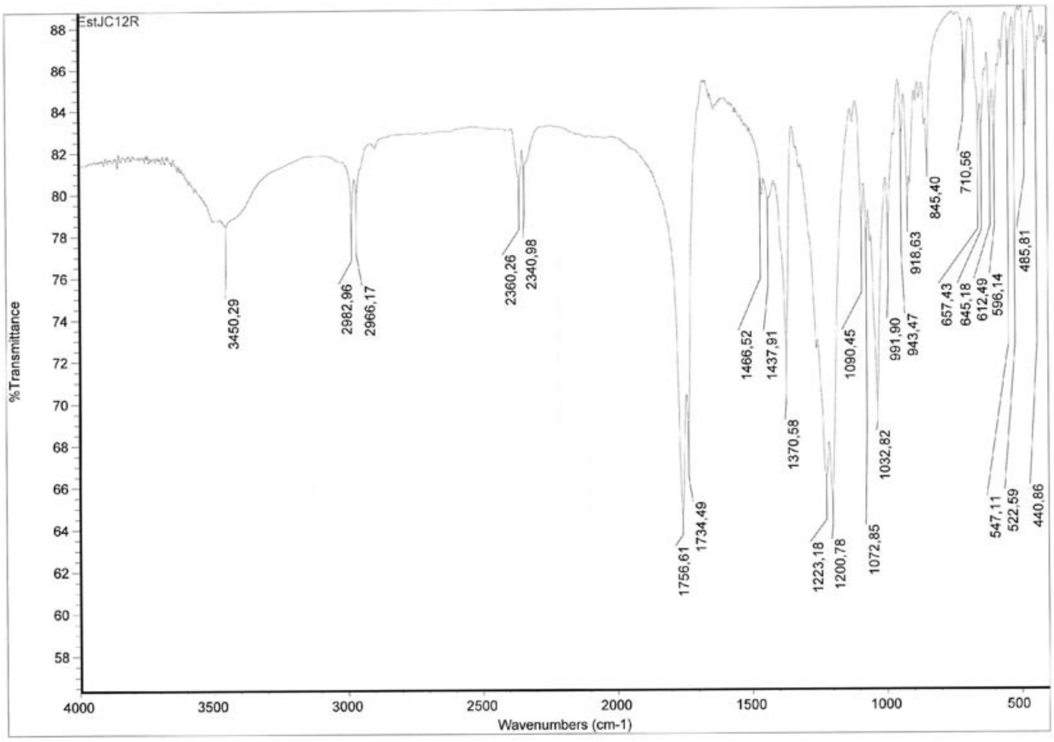

Figure S18. IR spectrum of **63.**

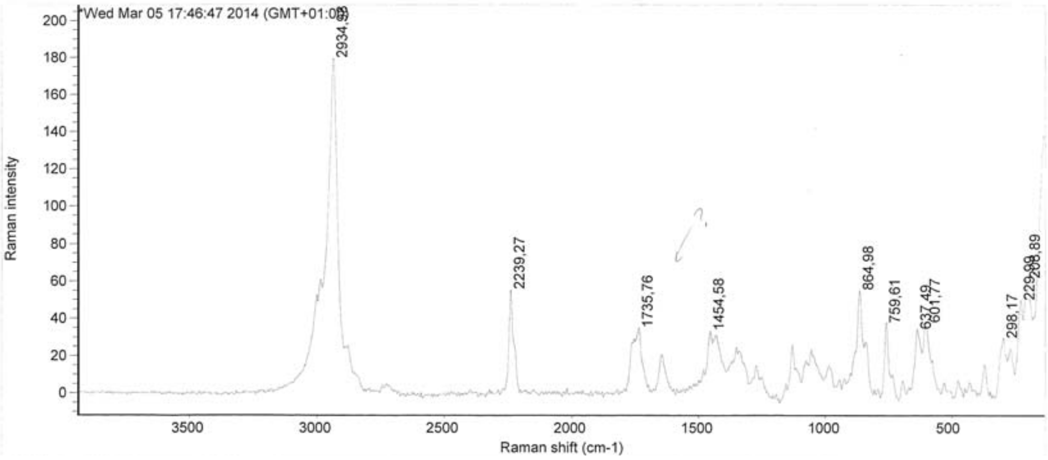

Figure S19. Raman spectrum of **11**.

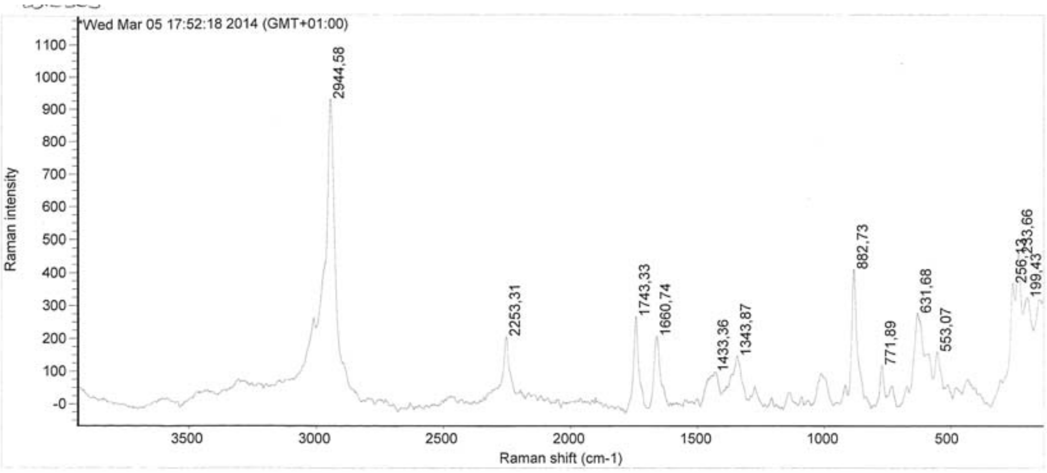

Figure S20. Raman spectrum of **13**.

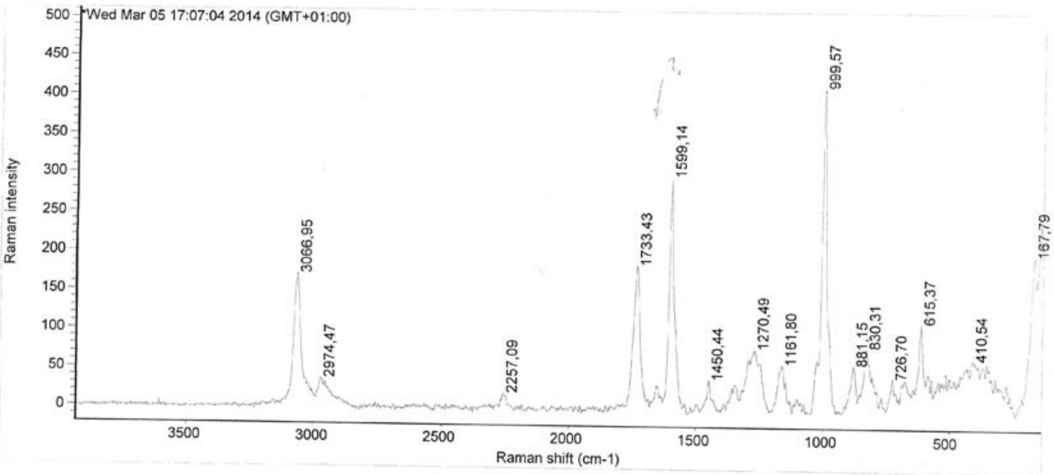

Figure S21. Raman spectrum of **18**.

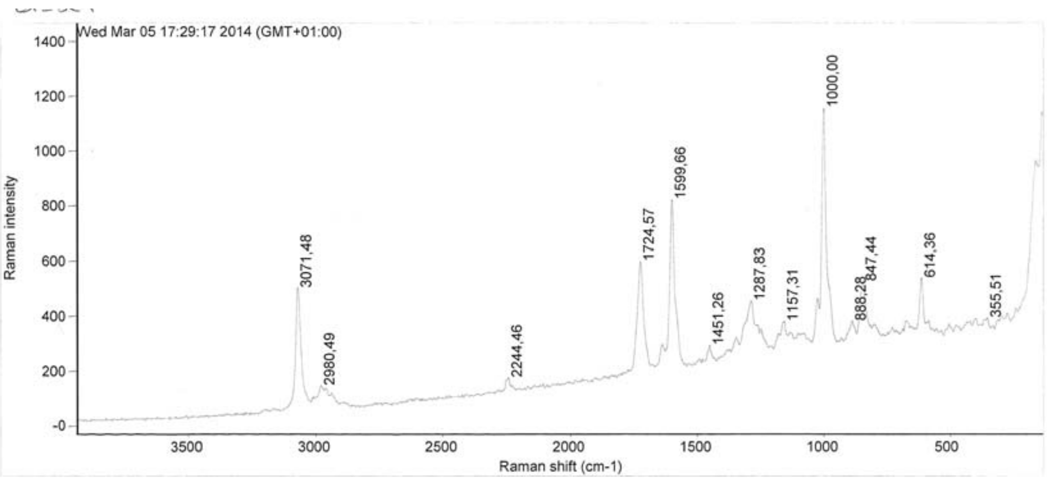

Figure S22. Raman spectrum of **20**.

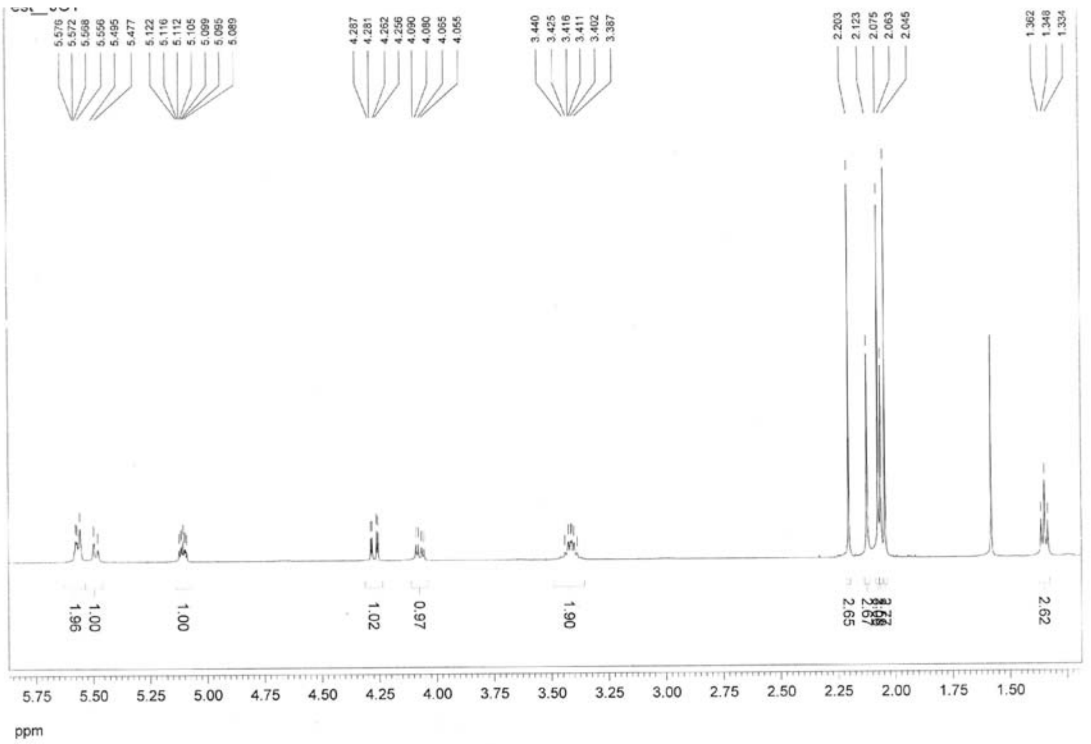

Figure S23. ^{1}H NMR spectrum of **11** in $CDCl_3$.

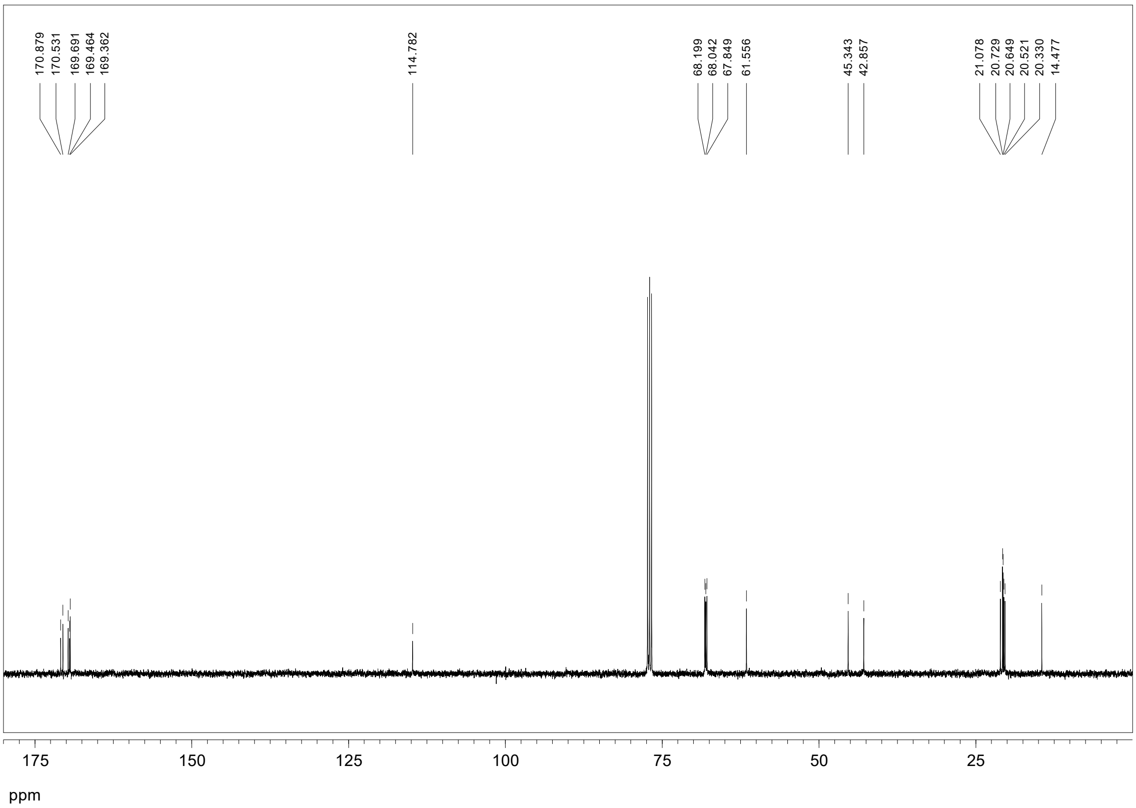

Figure S24. $^{13}C\{^1H\}$ NMR spectrum of **11** in $CDCl_3$.

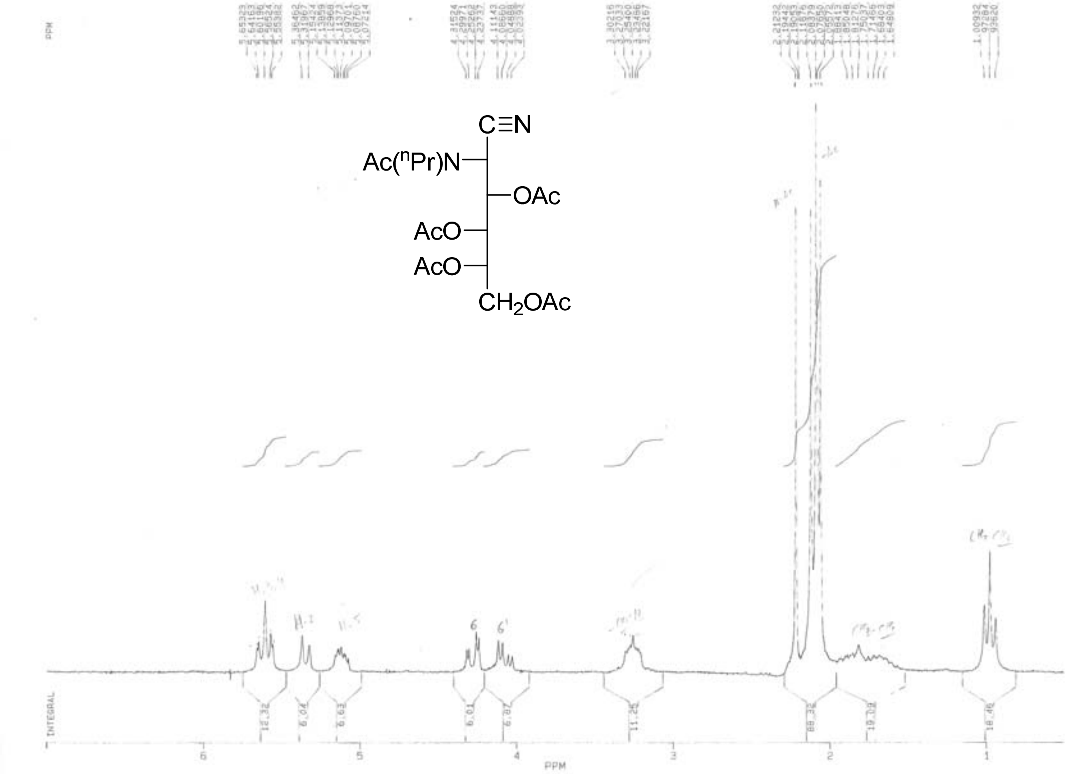

Figure S25. ^1H NMR spectrum of **12** in $CDCl_3$

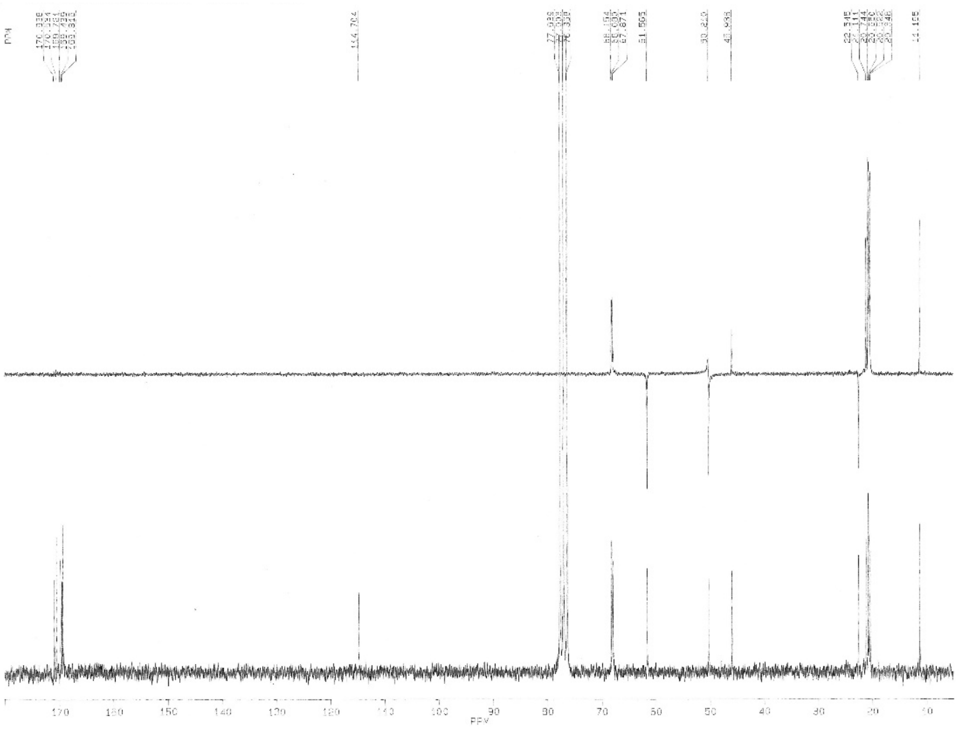

Figure S26. $^{13}C\{^1H\}$ NMR and DEPT spectra of **12** in $CDCl_3$

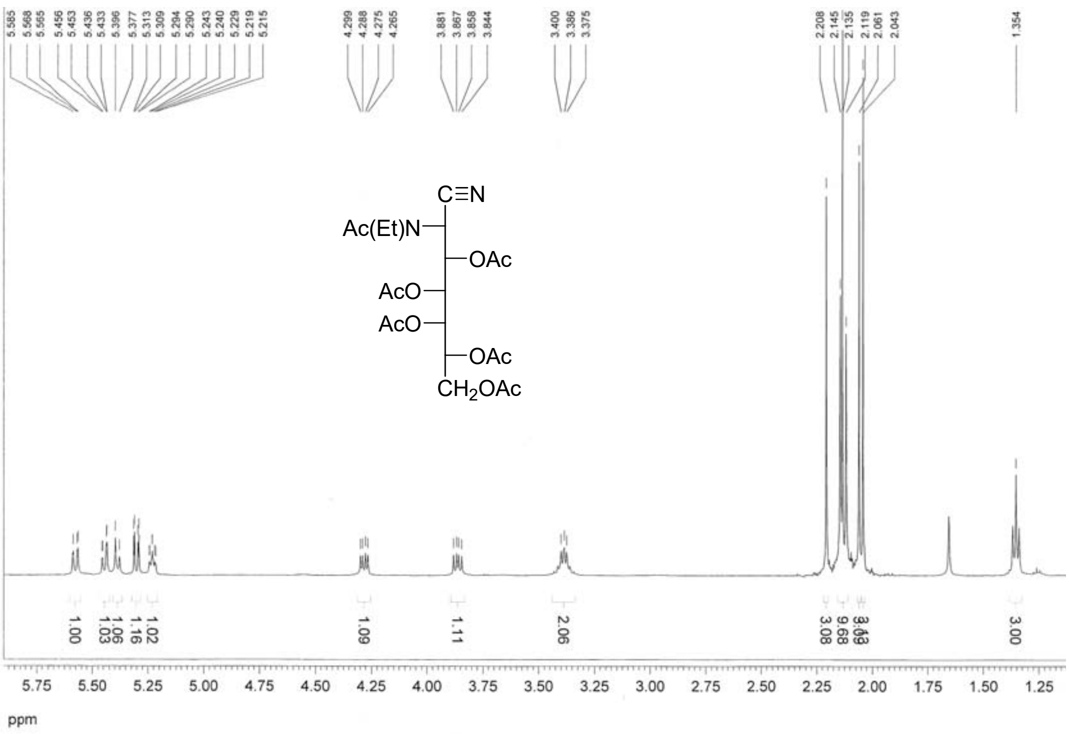

Figure S27. ^1H NMR spectrum of **13** in $CDCl_3$.

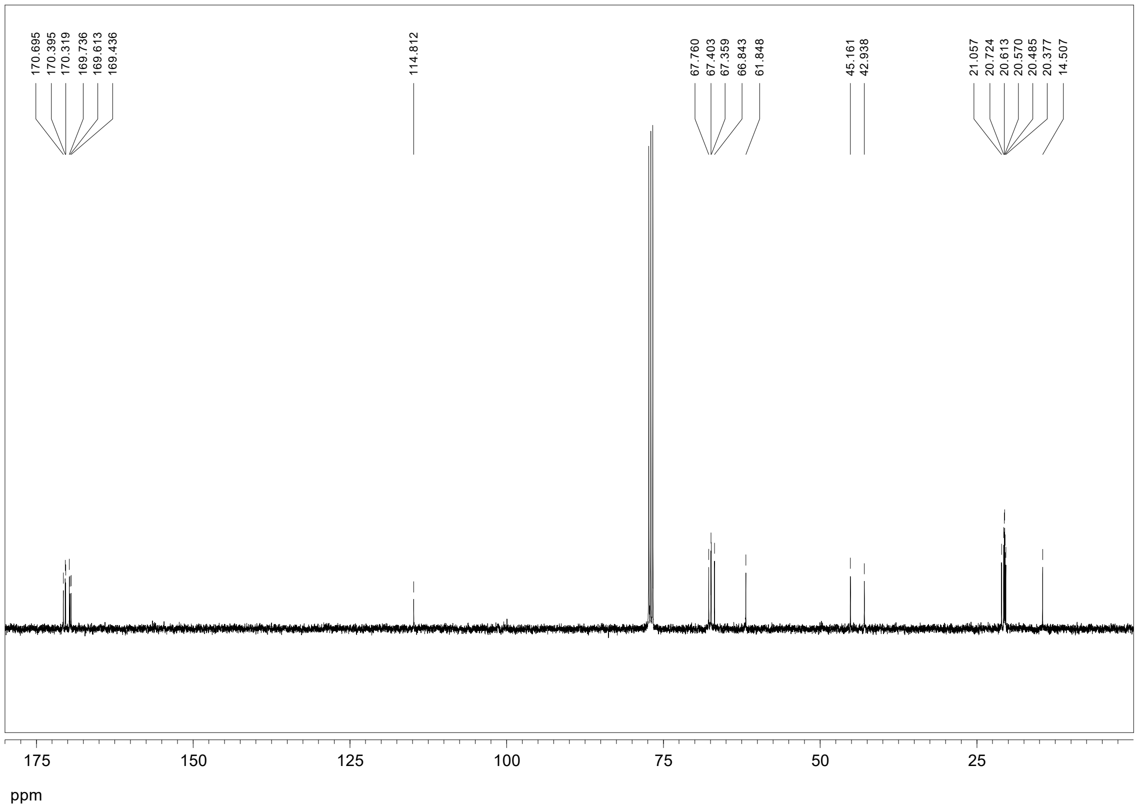

Figure S28. $^{13}C\{^1H\}$ NMR spectrum of **13** in $CDCl_3$.

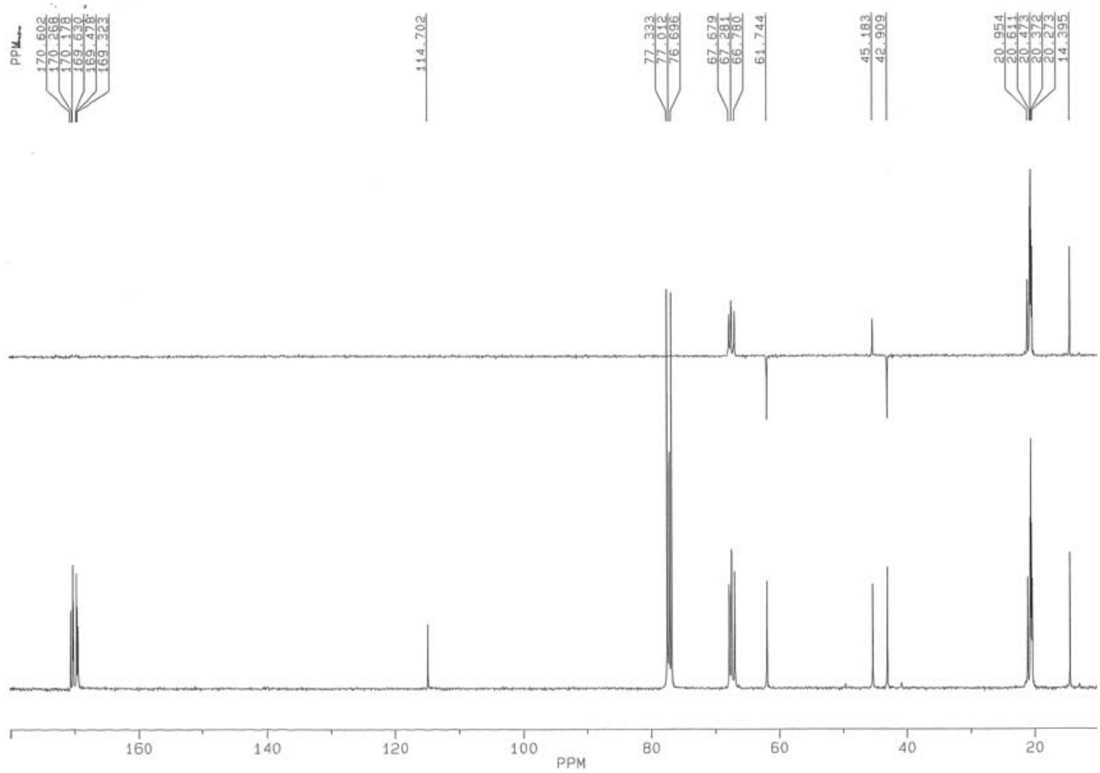

Figure S29. $^{13}C\{^1H\}$ NMR and DEPT spectra of **13** in $CDCl_3$.

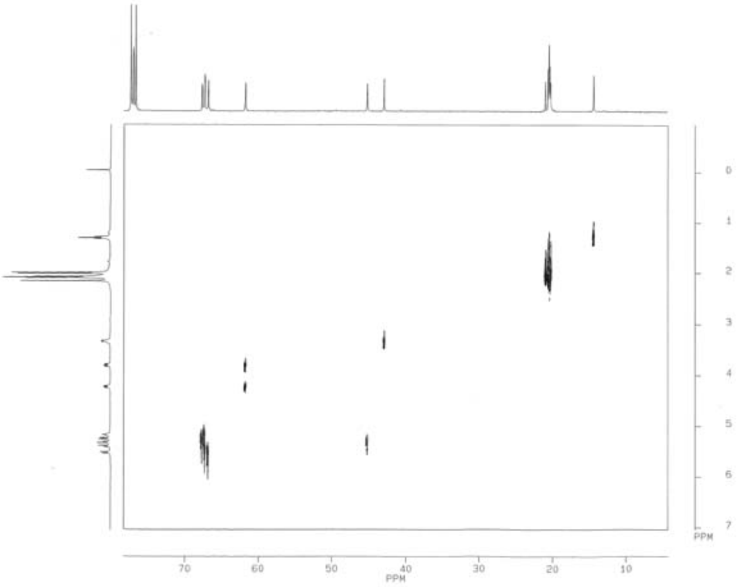

Figure S30. HMQC spectrum of **13** in $CDCl_3$.

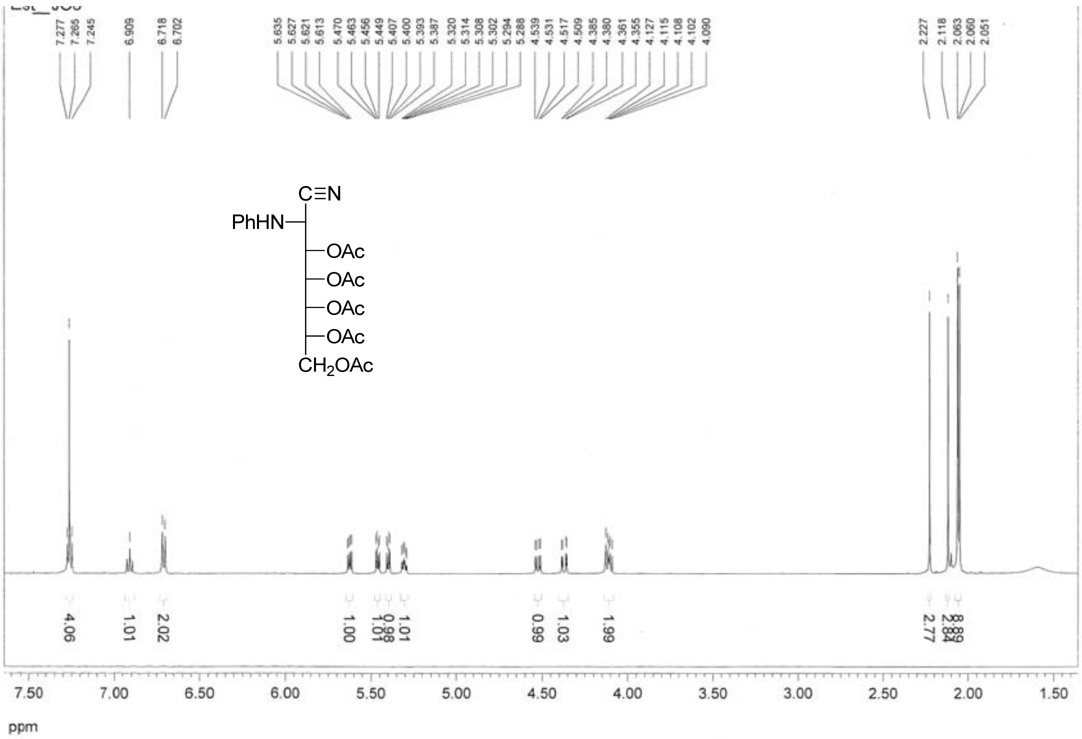

Figure S31. ^{1}H NMR spectrum of **17** in $CDCl_3$.

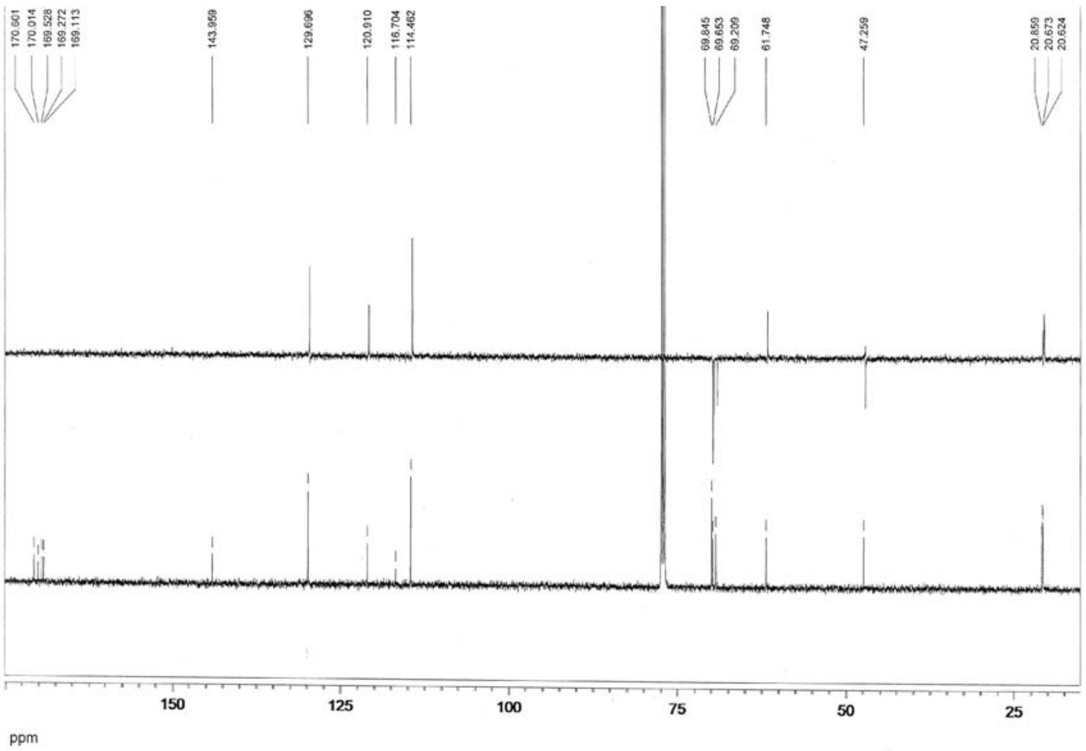

Figure S32. $^{13}C\{^1H\}$ NMR and DEPT spectra of **17** in $CDCl_3$.

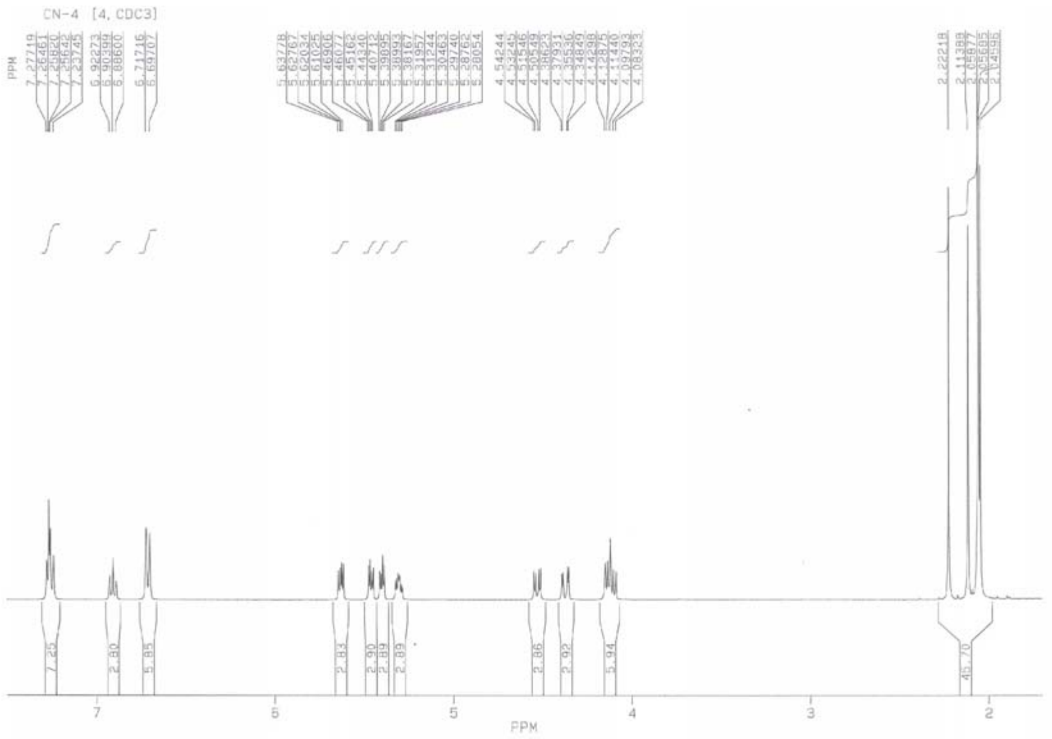

Figure S33. ^1H NMR spectrum of **17** in $CDCl_3$

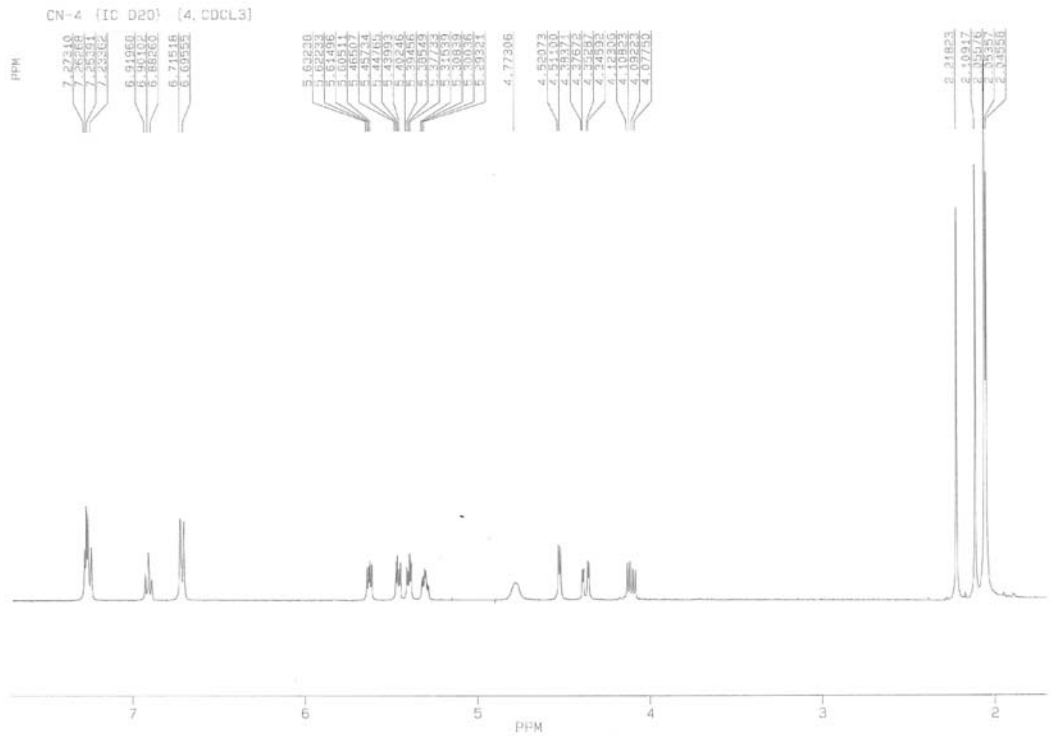

Figure S34. ^{1}H NMR spectrum of **17** in $CDCl_3$

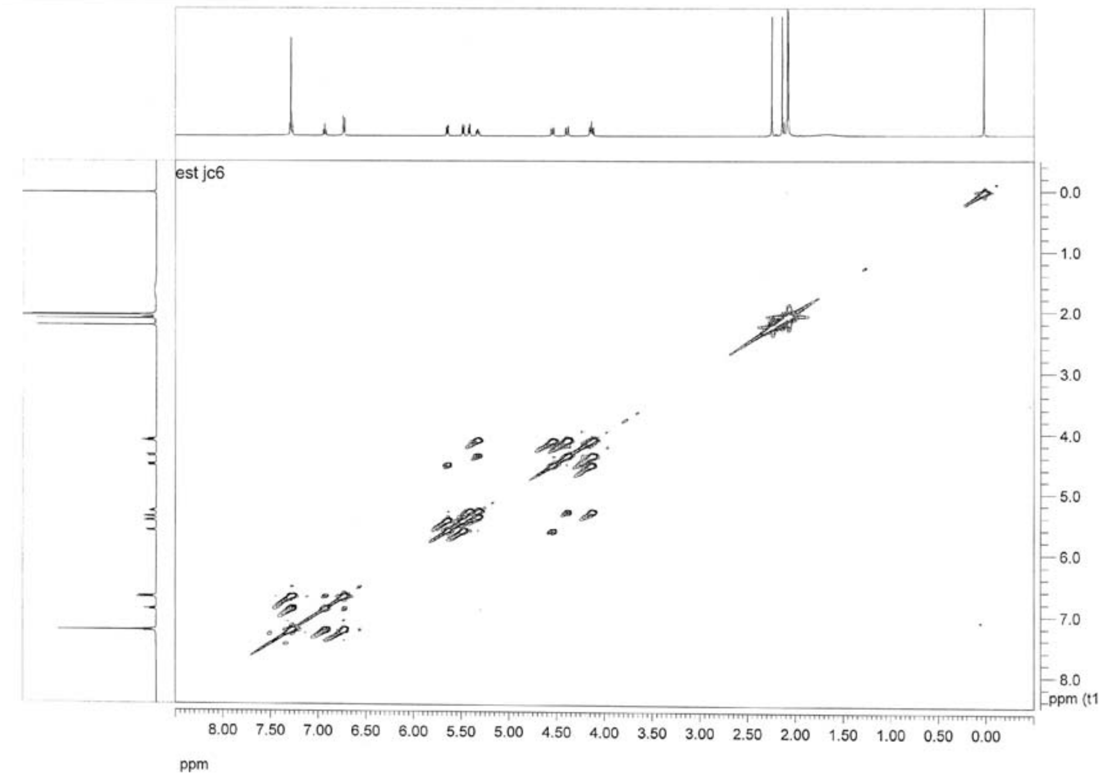

Figure S35. COSY spectrum of **17** in $CDCl_3$

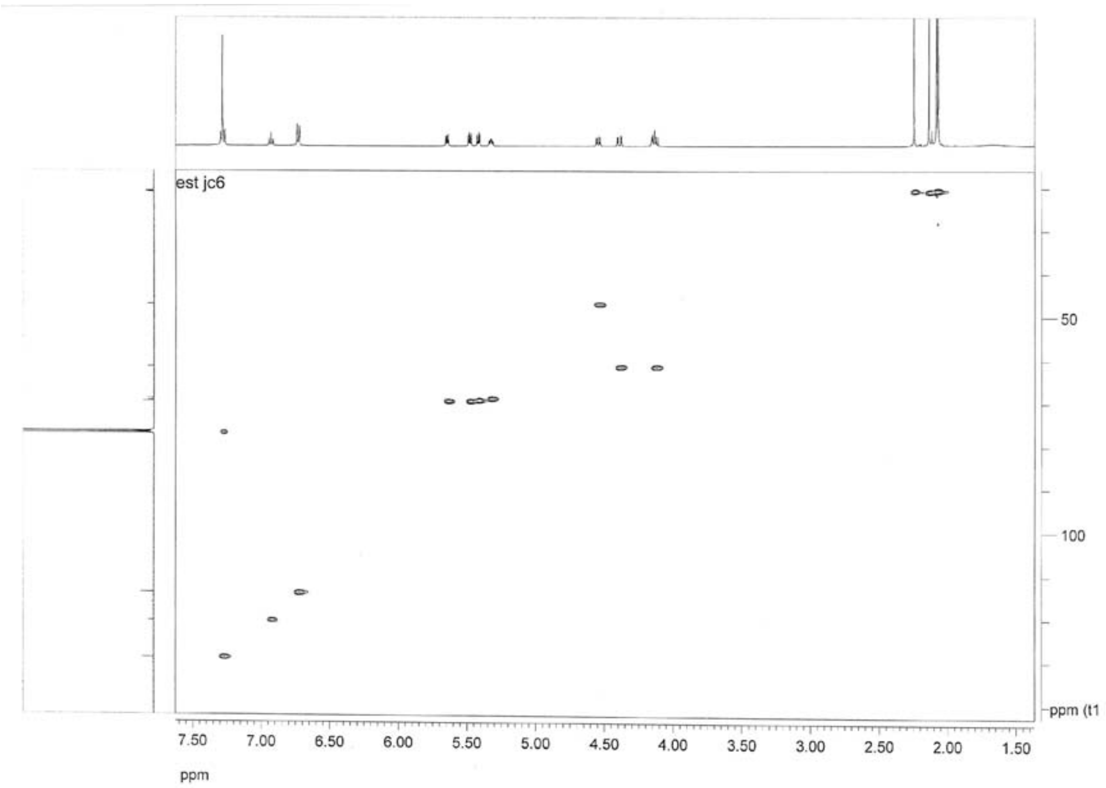

Figure S36. HMQC spectrum of **17** in $CDCl_3$

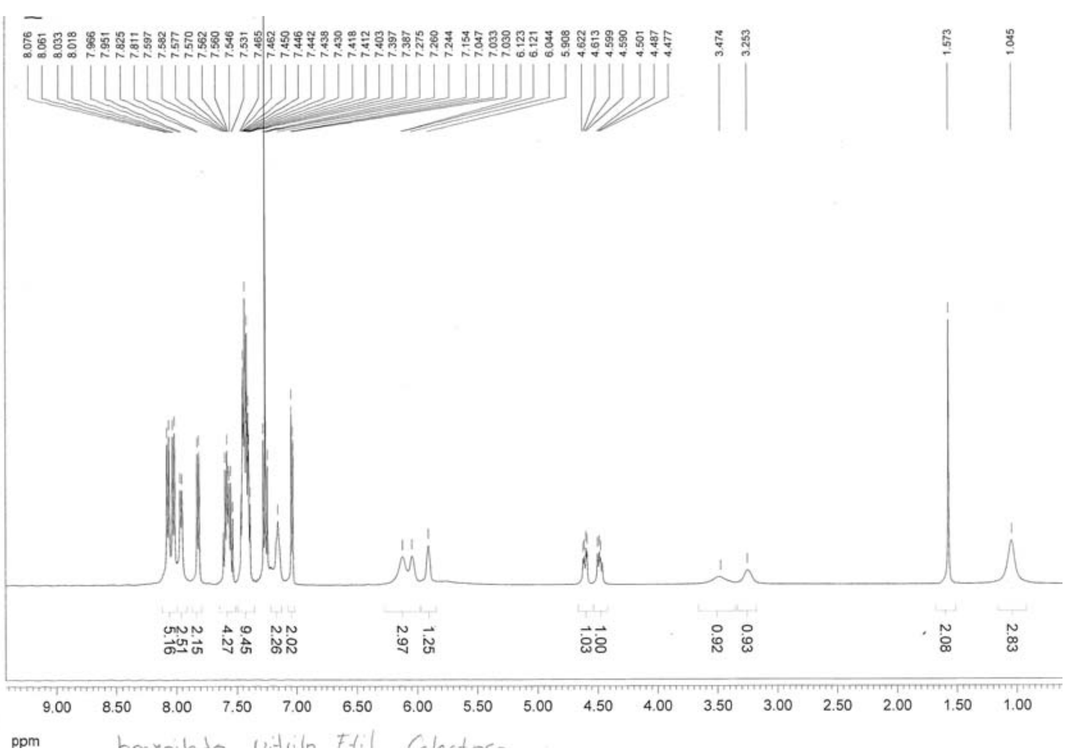

Figure S37. ^1H NMR spectrum of **18** in $CDCl_3$.

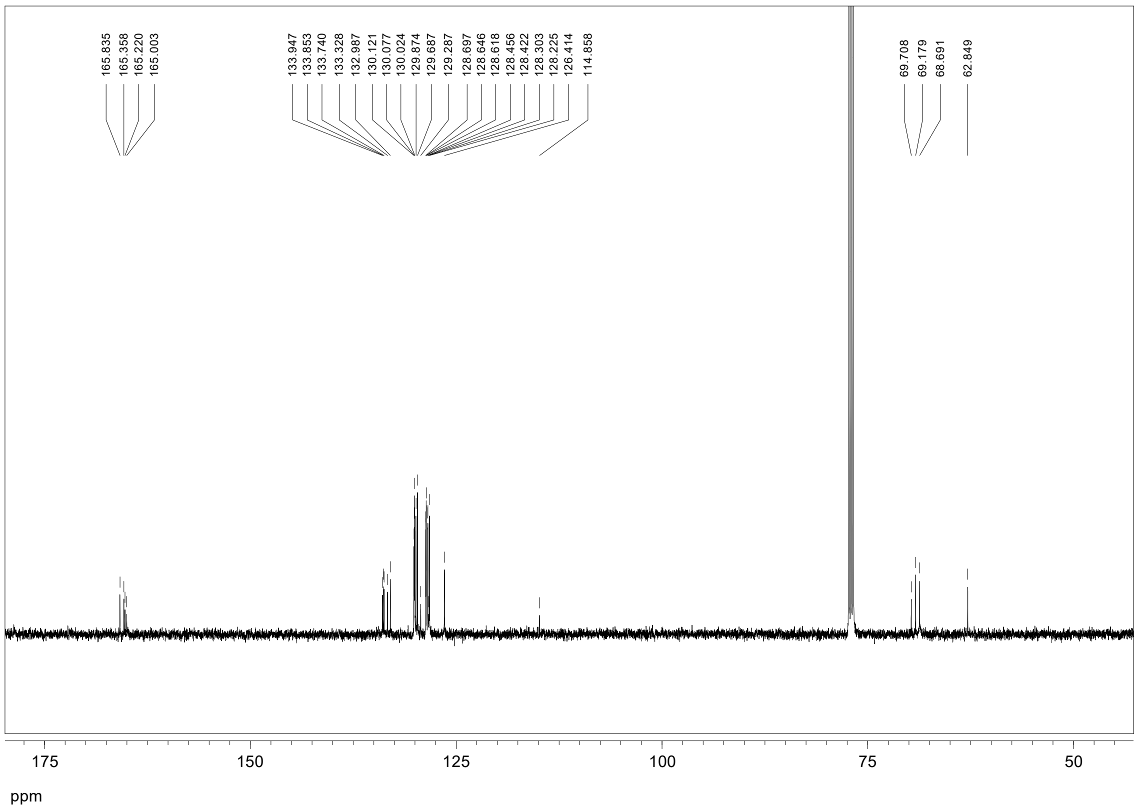

Figure S38. $^{13}C\{^1H\}$ NMR spectrum of **18** in $CDCl_3$.

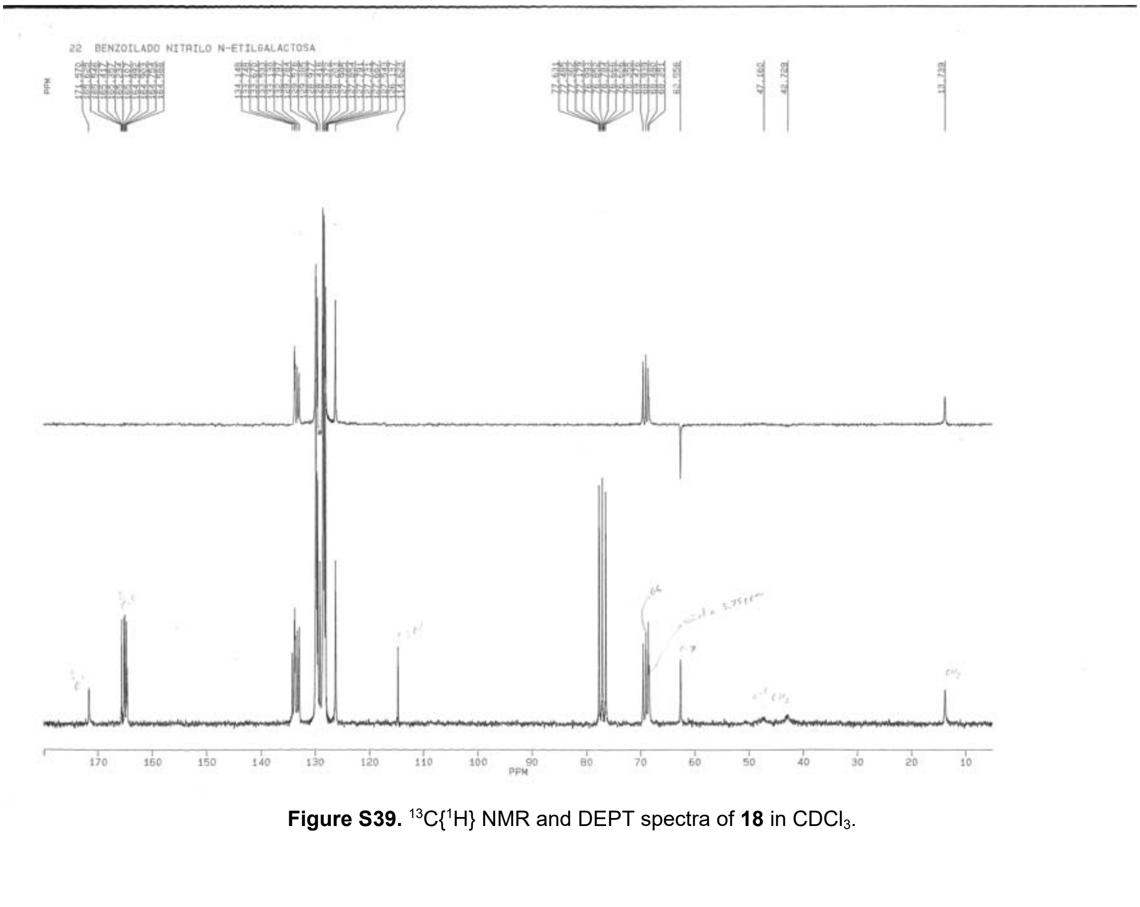

Figure S39. $^{13}C\{^1H\}$ NMR and DEPT spectra of **18** in $CDCl_3$.

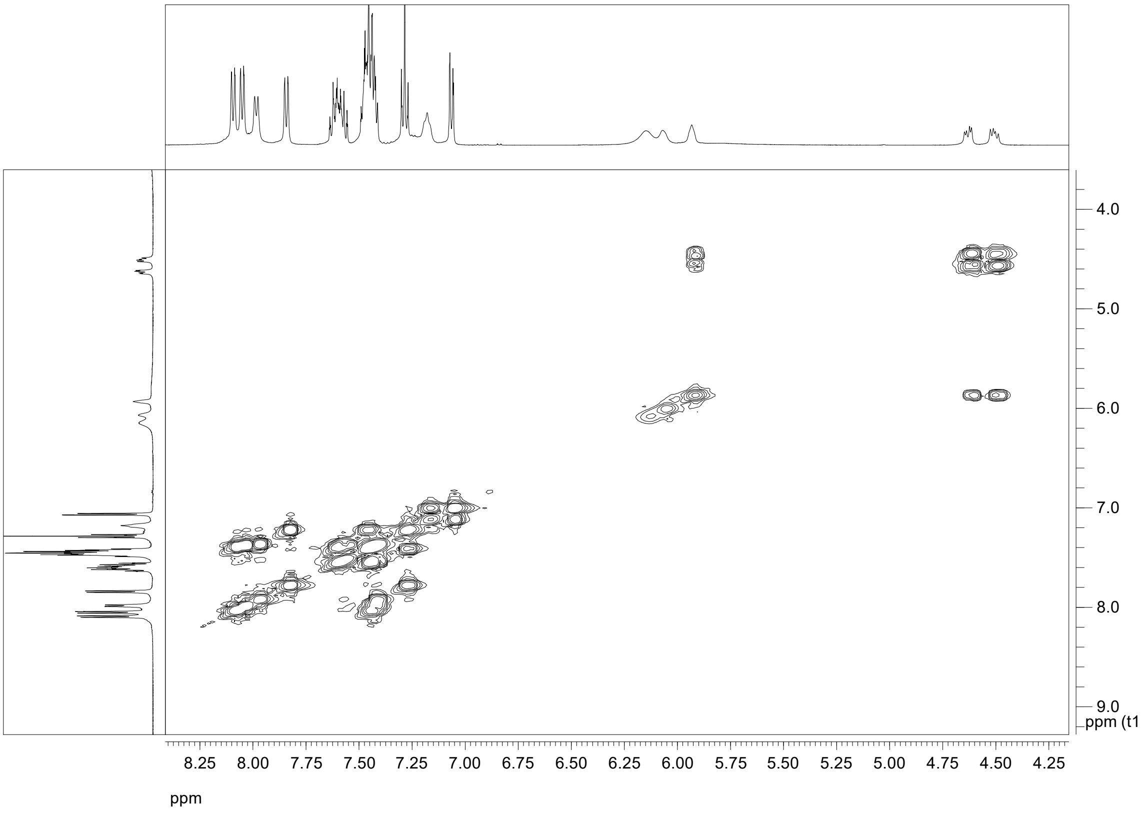

Figure S40. COSY spectrum of **18** in $CDCl_3$.

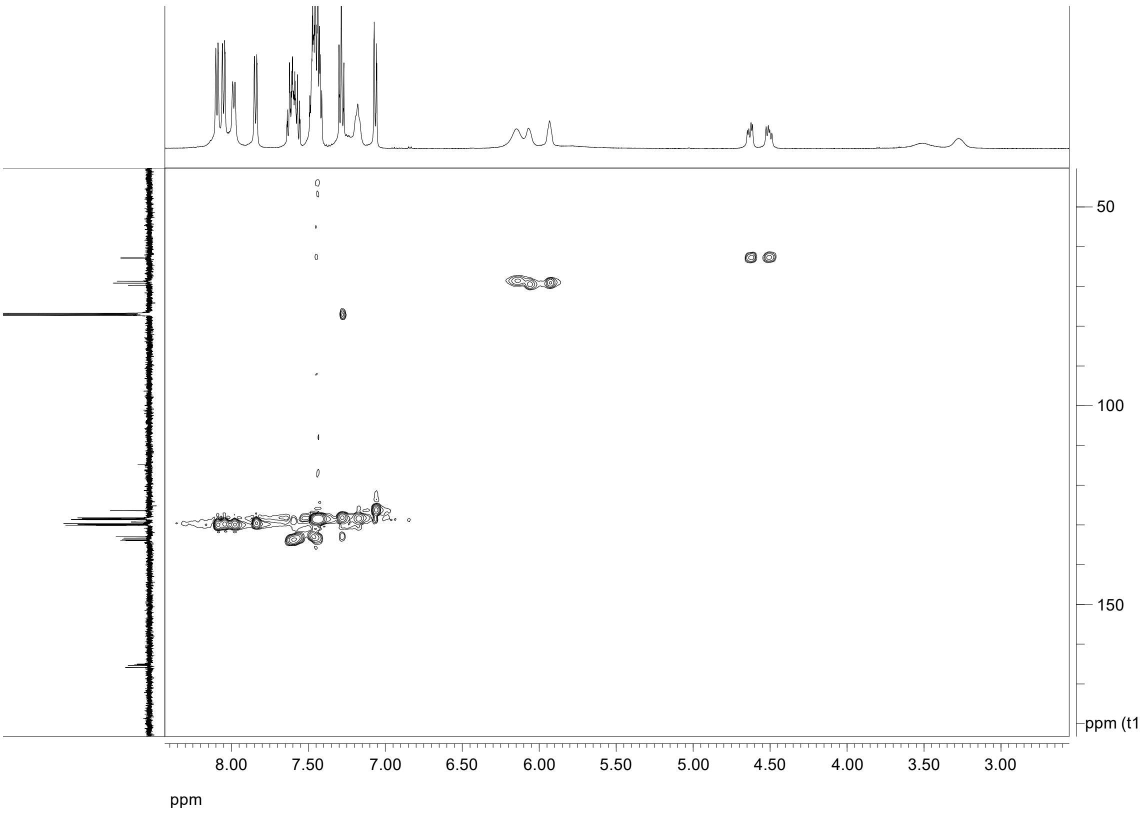

Figure S41. HMQC spectrum of **18** in $CDCl_3$.

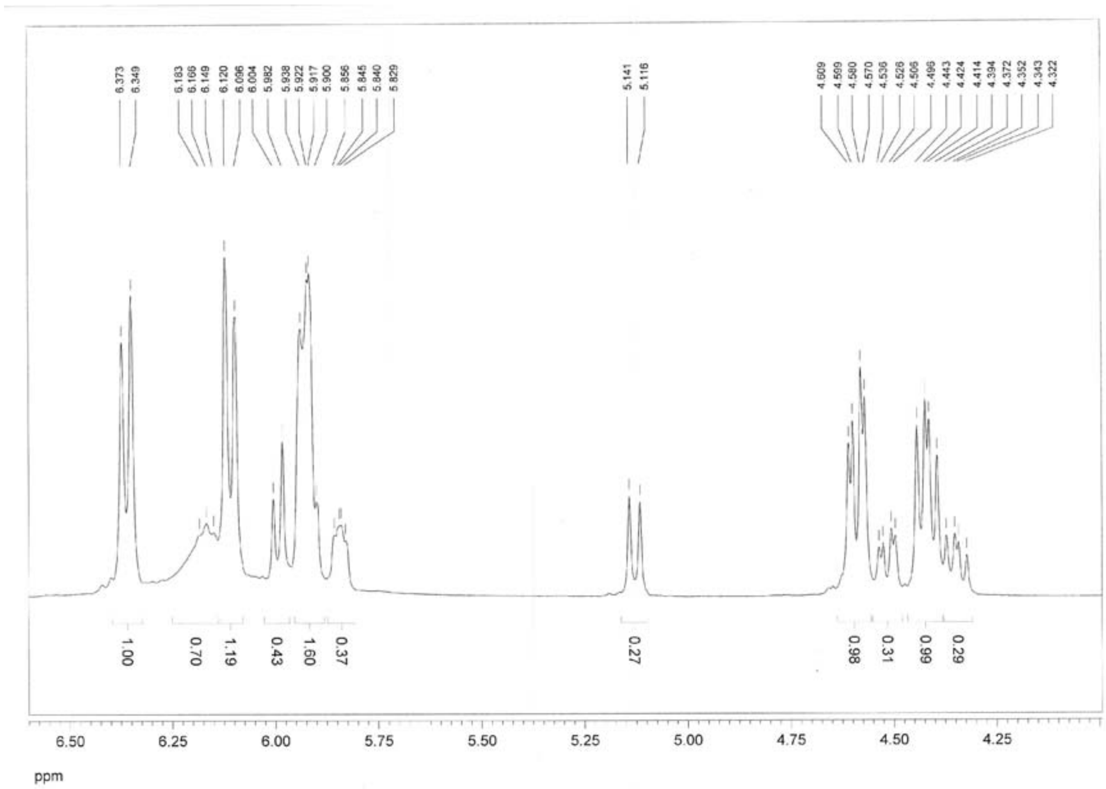

Figure S42. Magnified ^1H NMR espectral zone of **18** in $CDCl_3$ (240K).

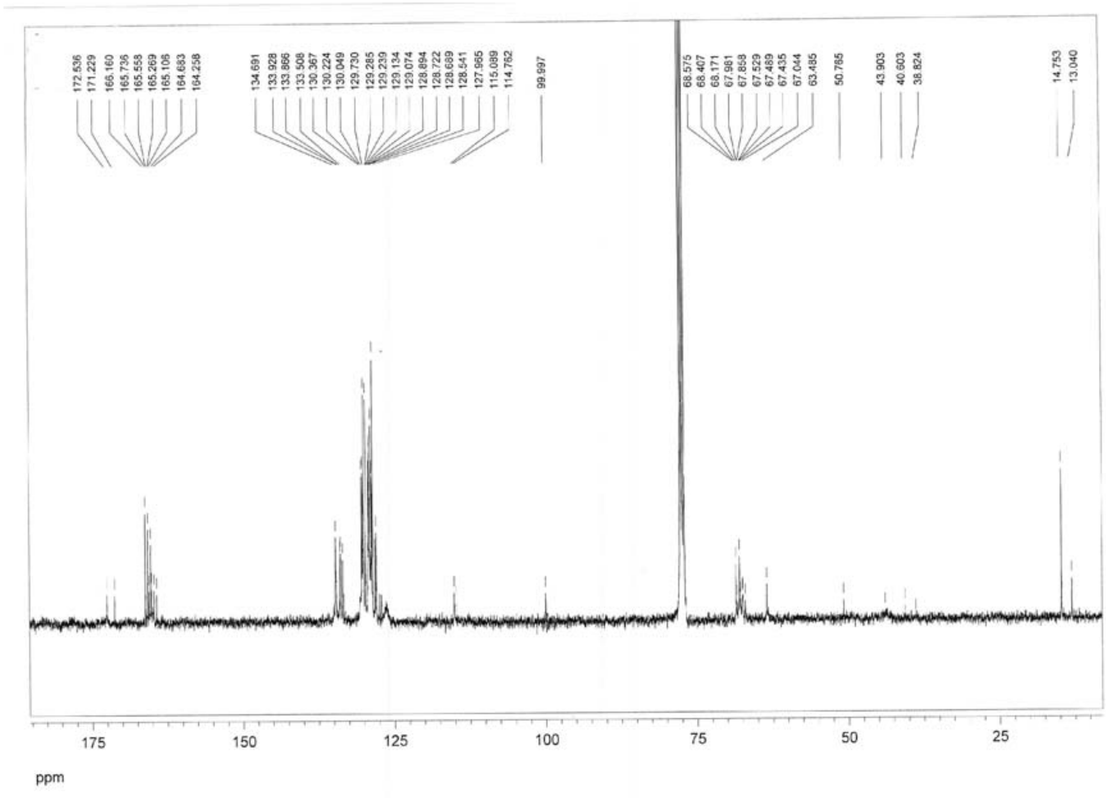

Figure S43. $^{13}C\{^1H\}$ NMR spectrum of **18** in $CDCl_3$ (240K).

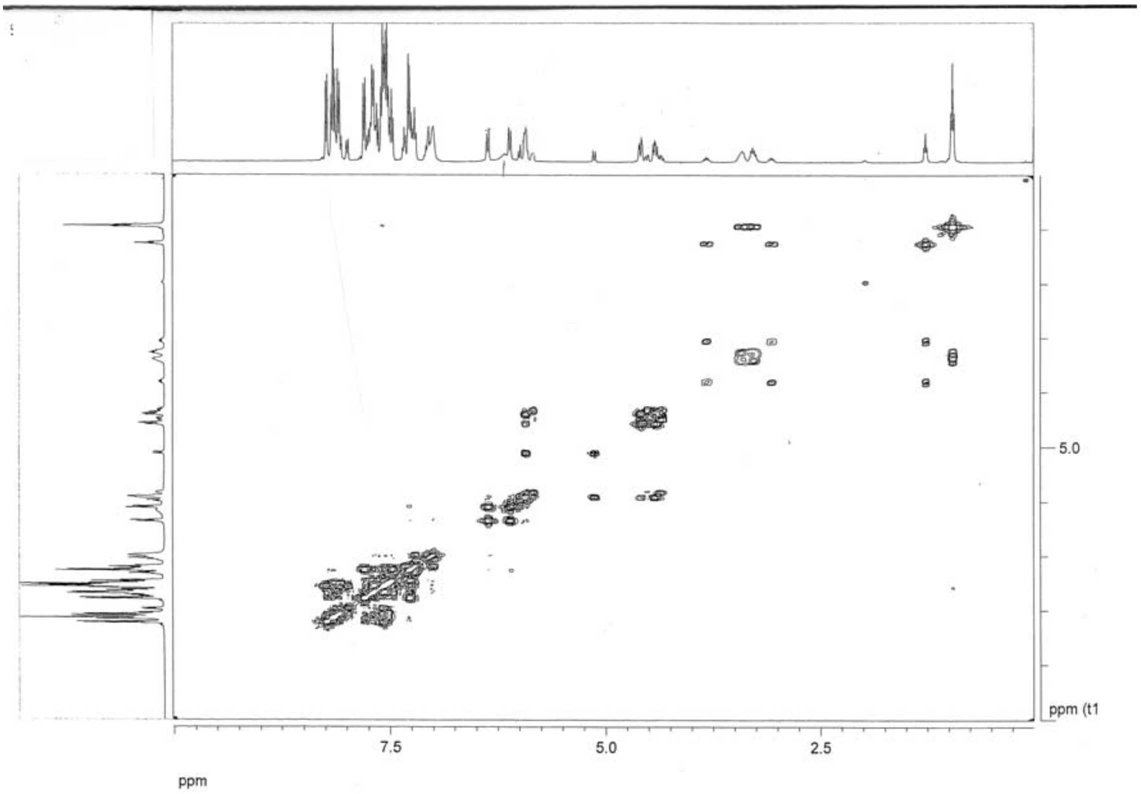

Figure S44. COSY spectrum of **18** in $CDCl_3$ (240K).

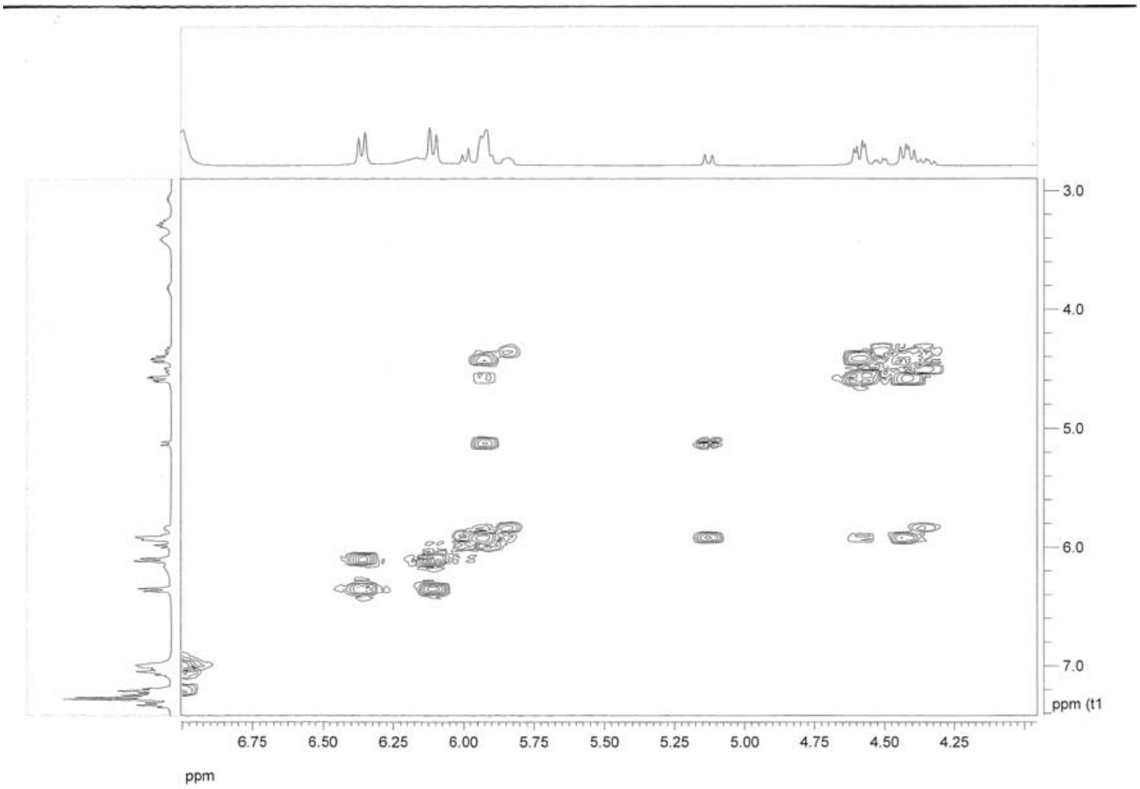

Figure S45. Magnified COSY espectral zone of **18** in $CDCl_3$ (240K).

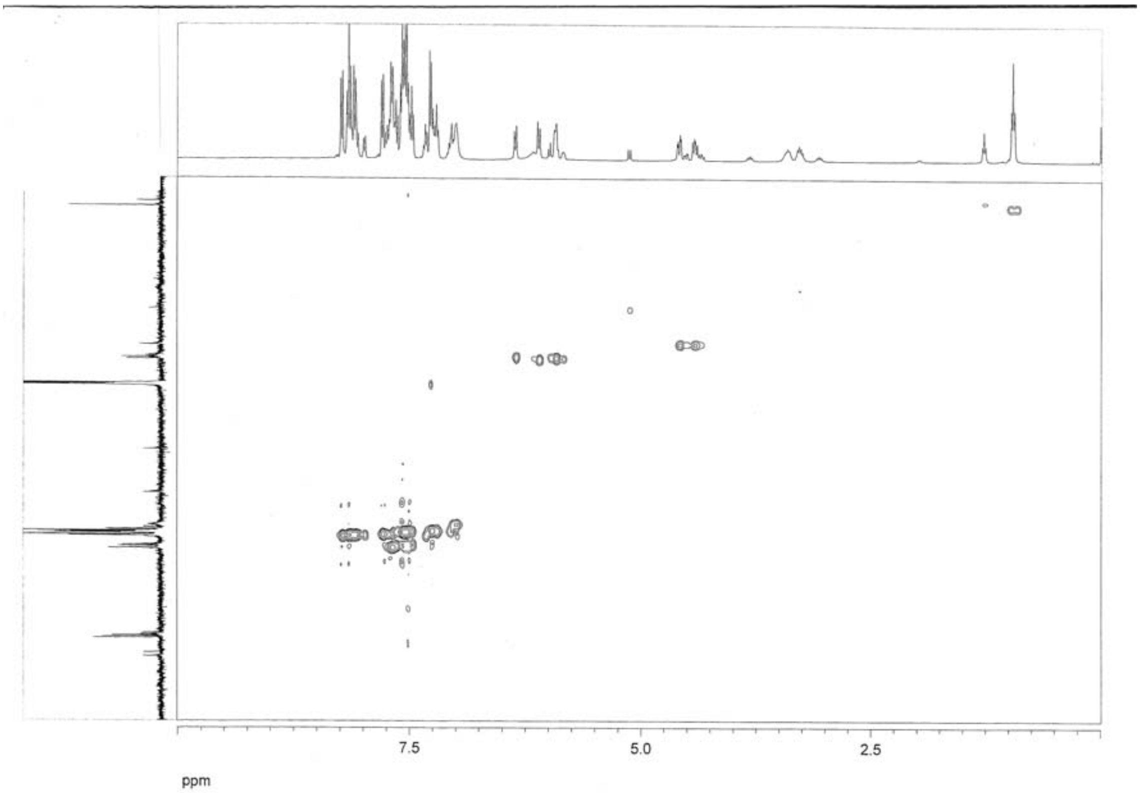

Figure S46. HMQC spectrum of **18** in $CDCl_3$ (240K).

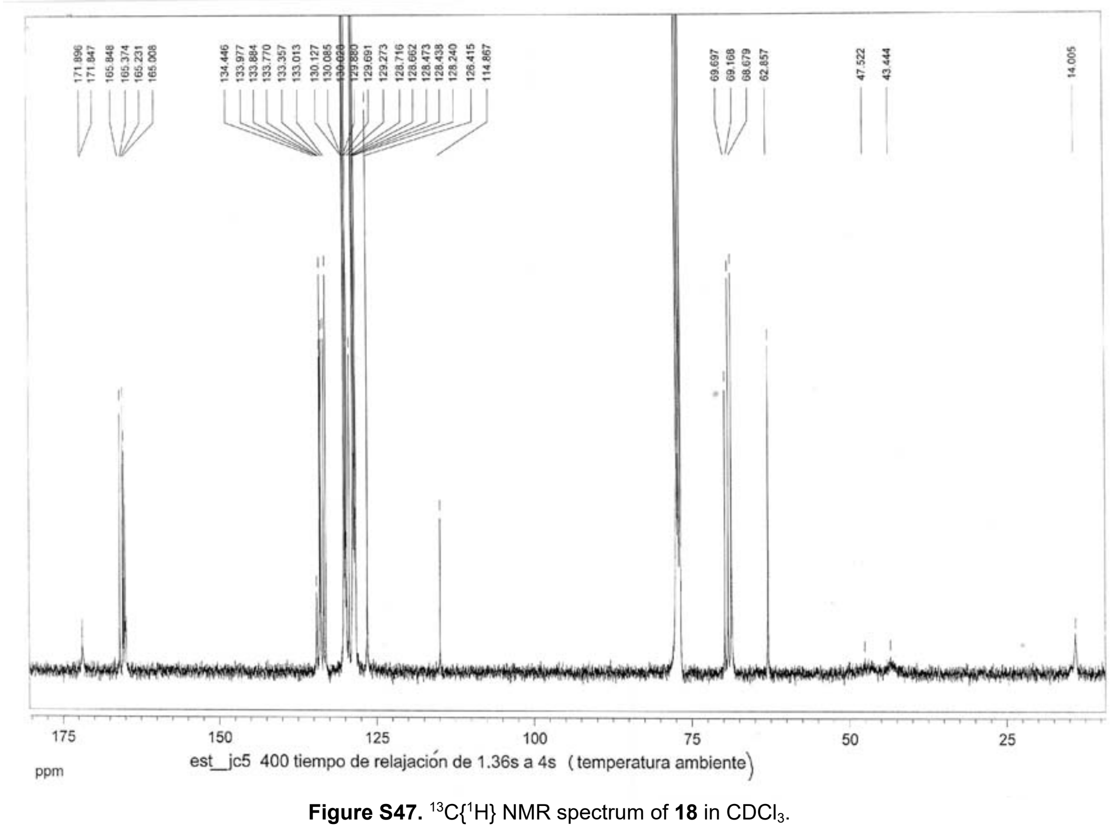

Figure S47. $^{13}C\{^1H\}$ NMR spectrum of **18** in $CDCl_3$.

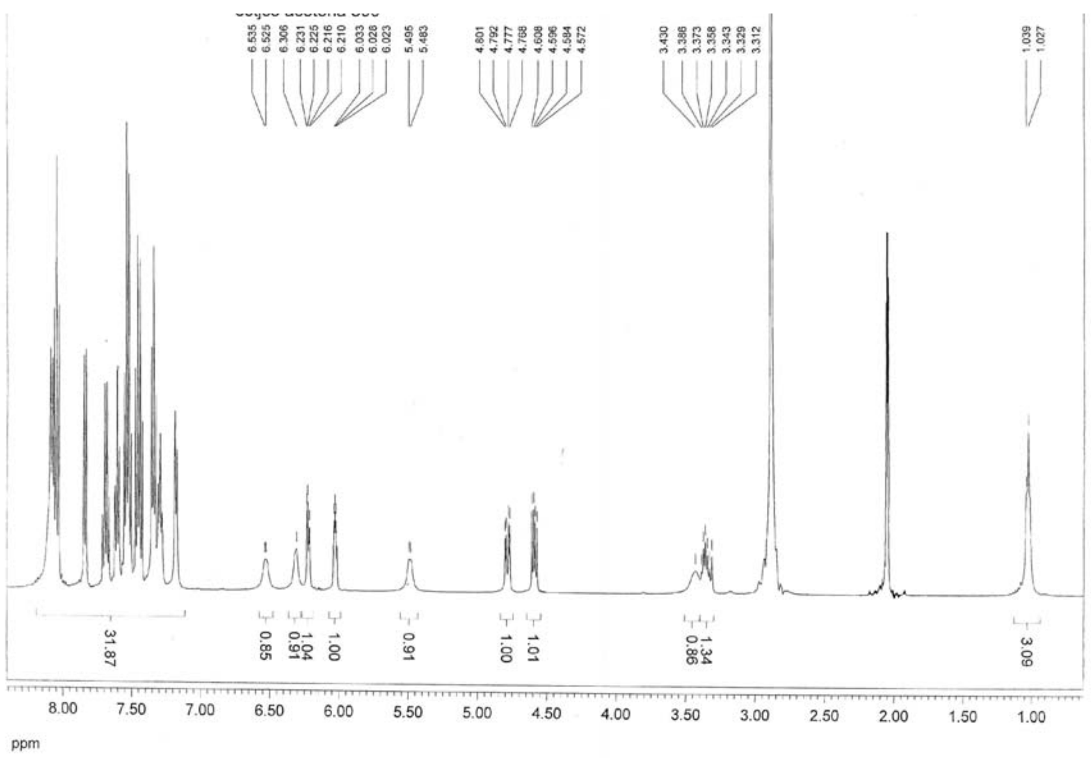

Figure S48. ^{1}H NMR spectrum of **18** in Acetone-d_6.

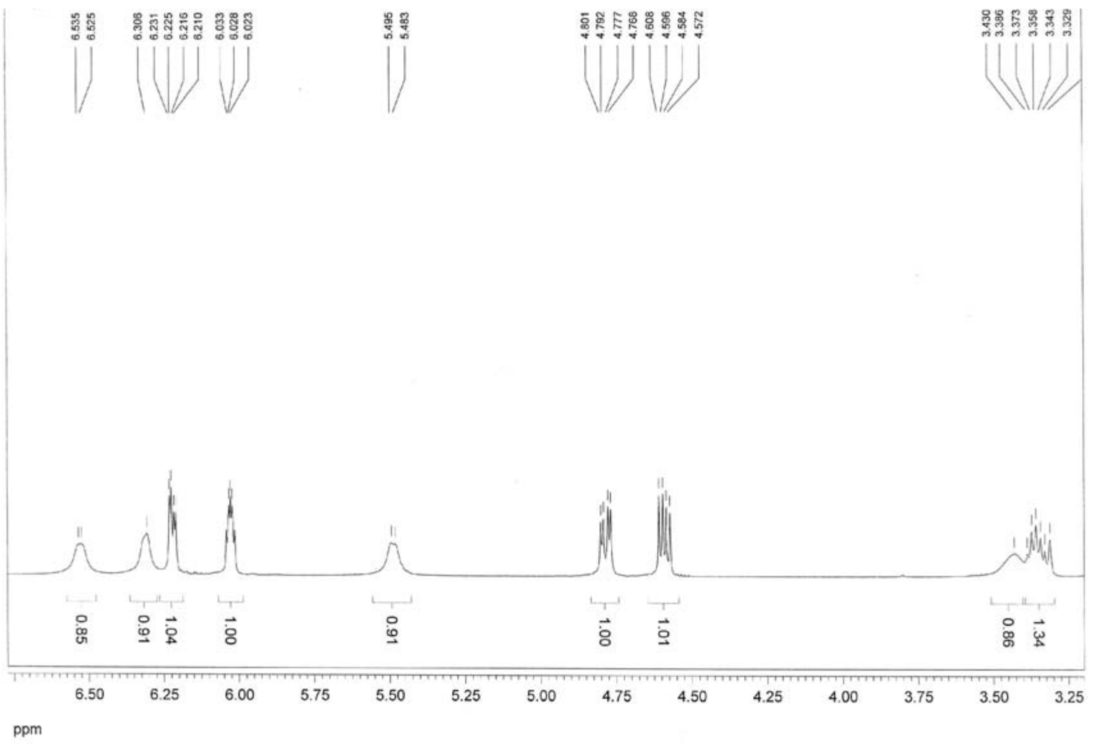

Figure S49. Magnified ^{1}H NMR espectral zone of **18** in Acetone-d_6.

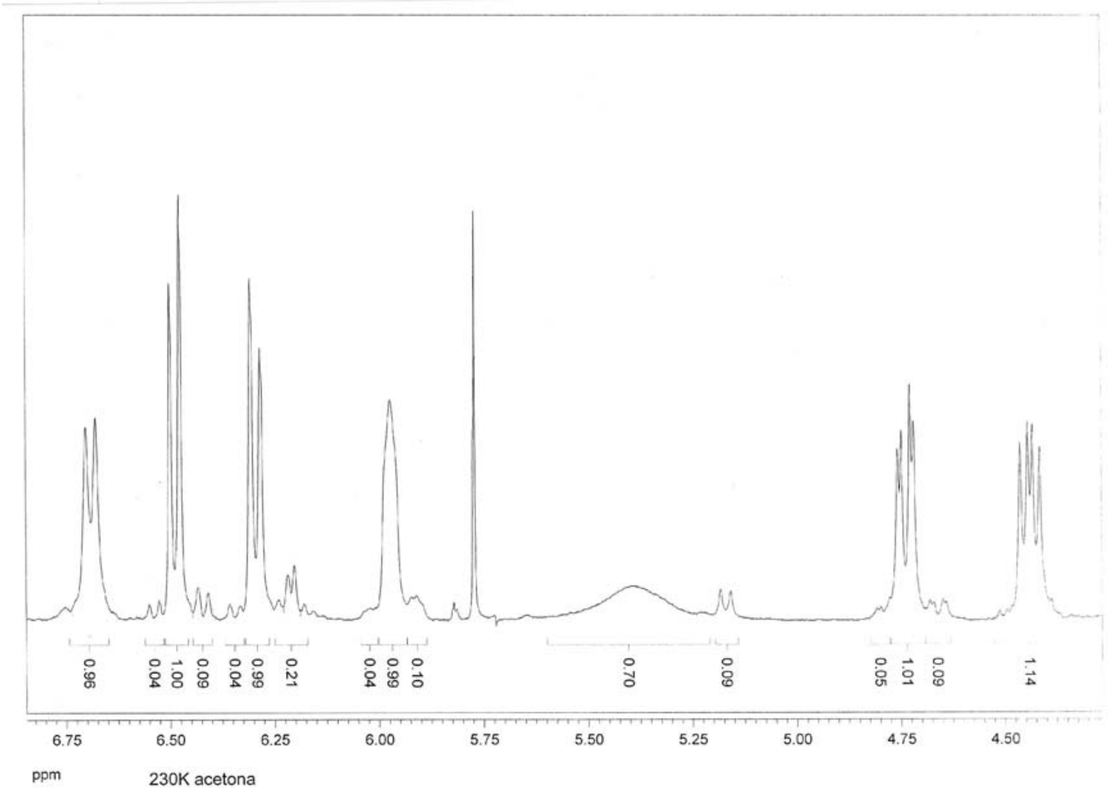

Figure S50. Magnified ^{1}H NMR espectral zone of **18** in Acetone-d_6 (230K).

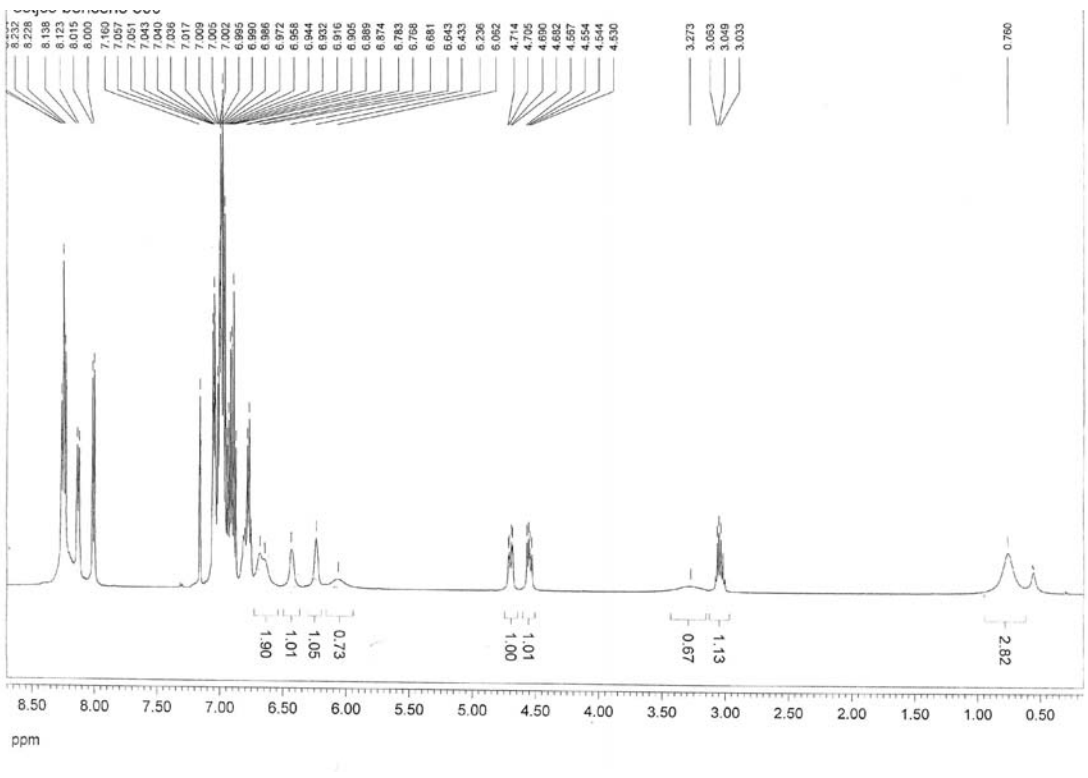

Figure S51. ^{1}H NMR spectrum of **18** in Benzene-d_6.

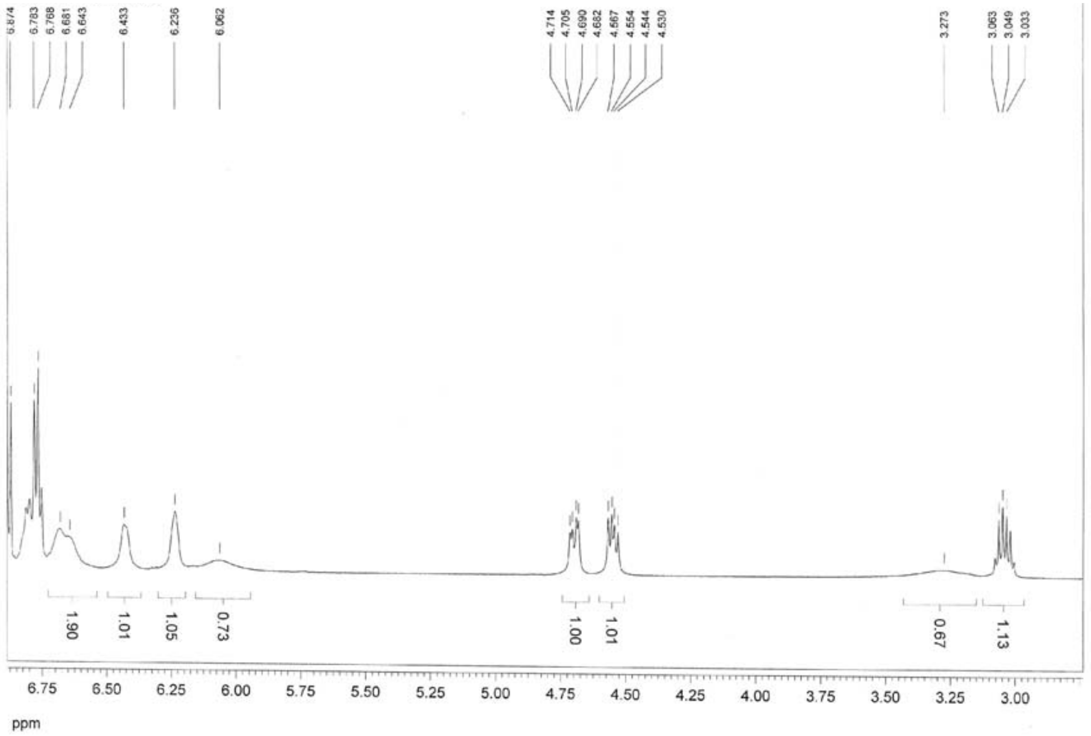

Figure S52. Magnified ^{1}H NMR espectral zone of **18** in Benzene-d_6.

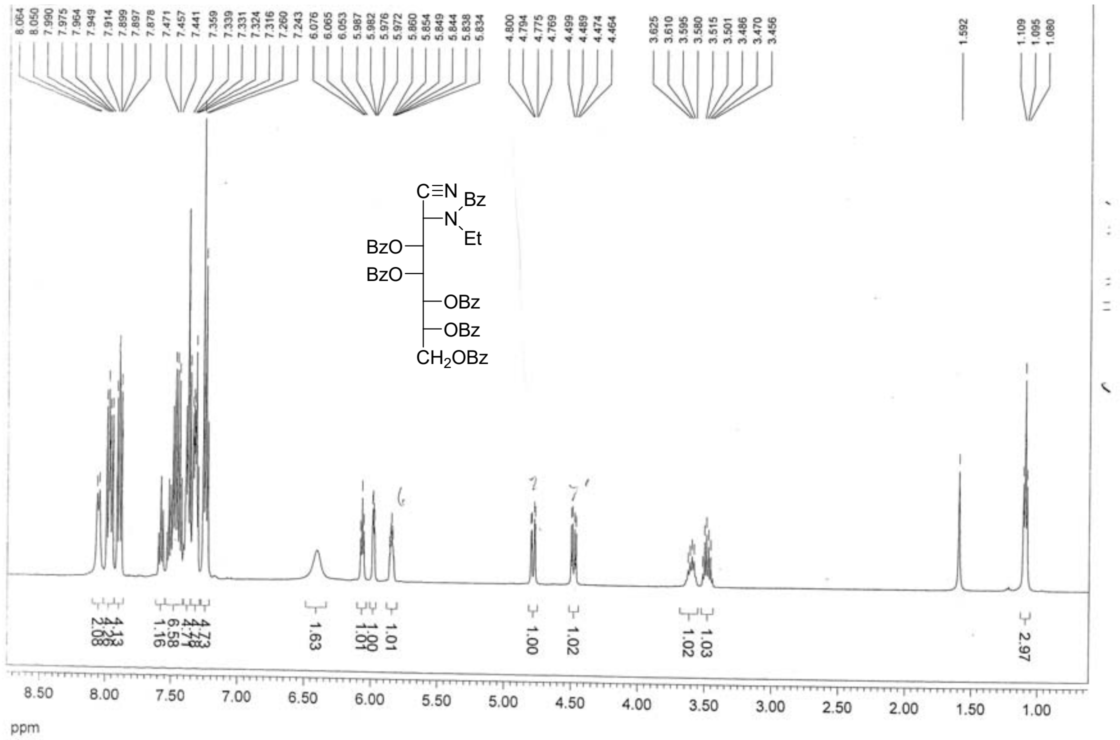

Figure S53. ^{1}H NMR spectrum of **20** in $CDCl_3$.

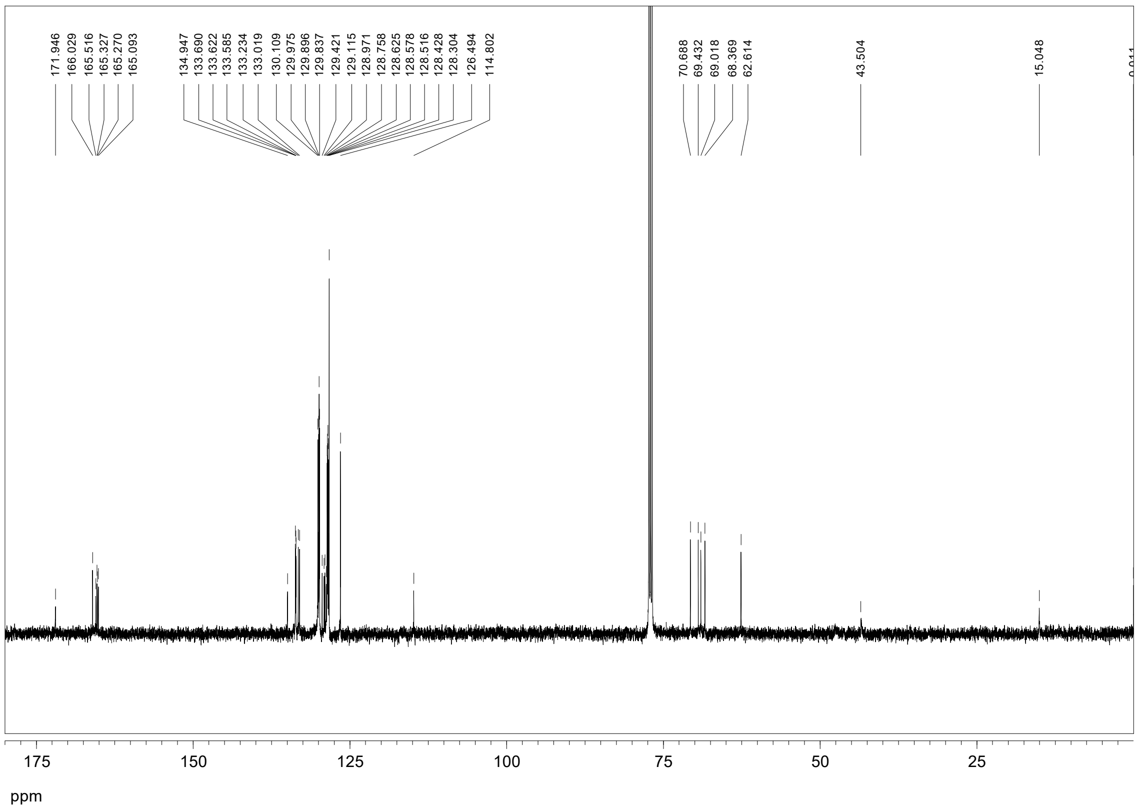

Figure S54. $^{13}C\{^1H\}$ NMR spectrum of **20** in $CDCl_3$.

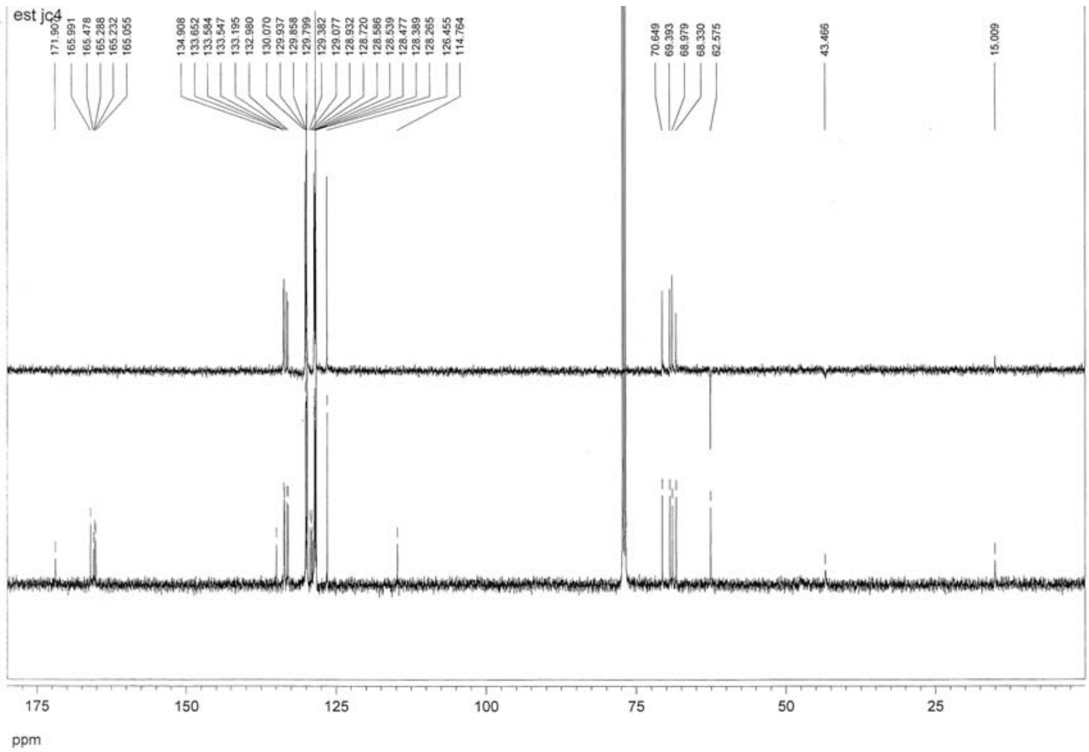

Figure S55. $^{13}C\{^1H\}$ NMR and DEPT spectra of **20** in $CDCl_3$.

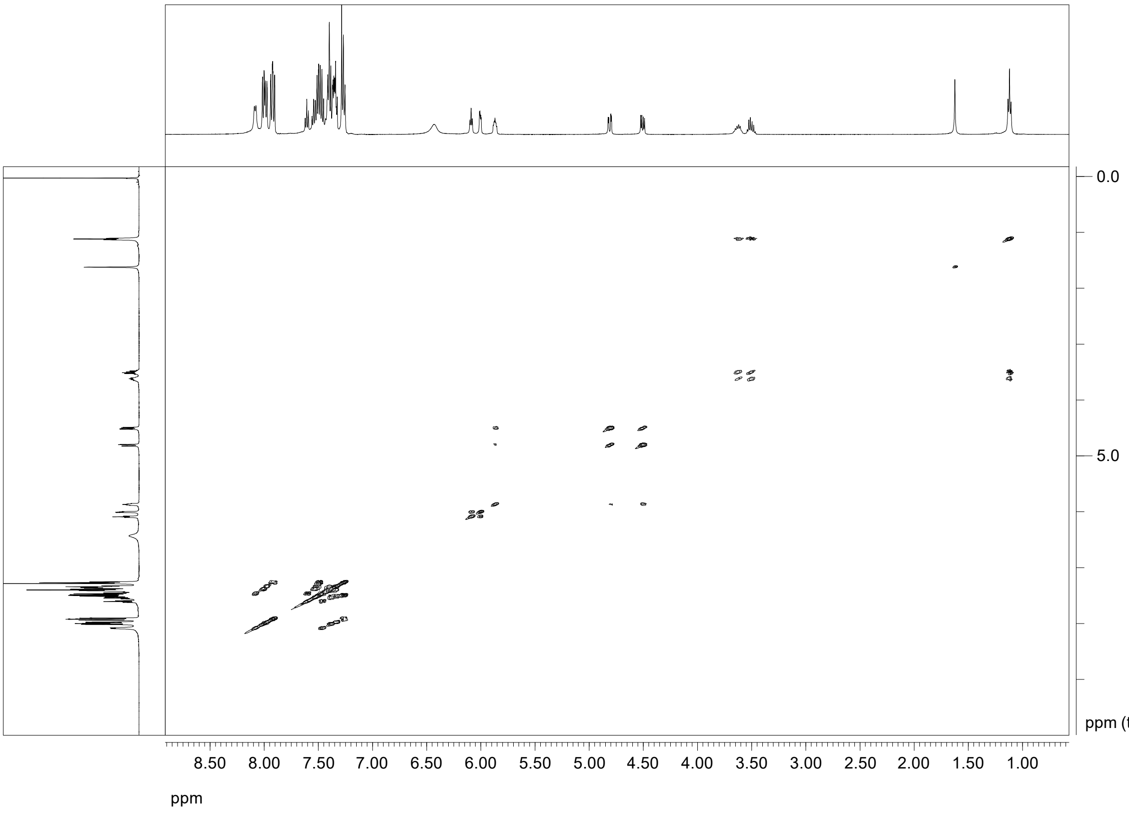

Figure S56. COSY spectrum of **20** in $CDCl_3$.

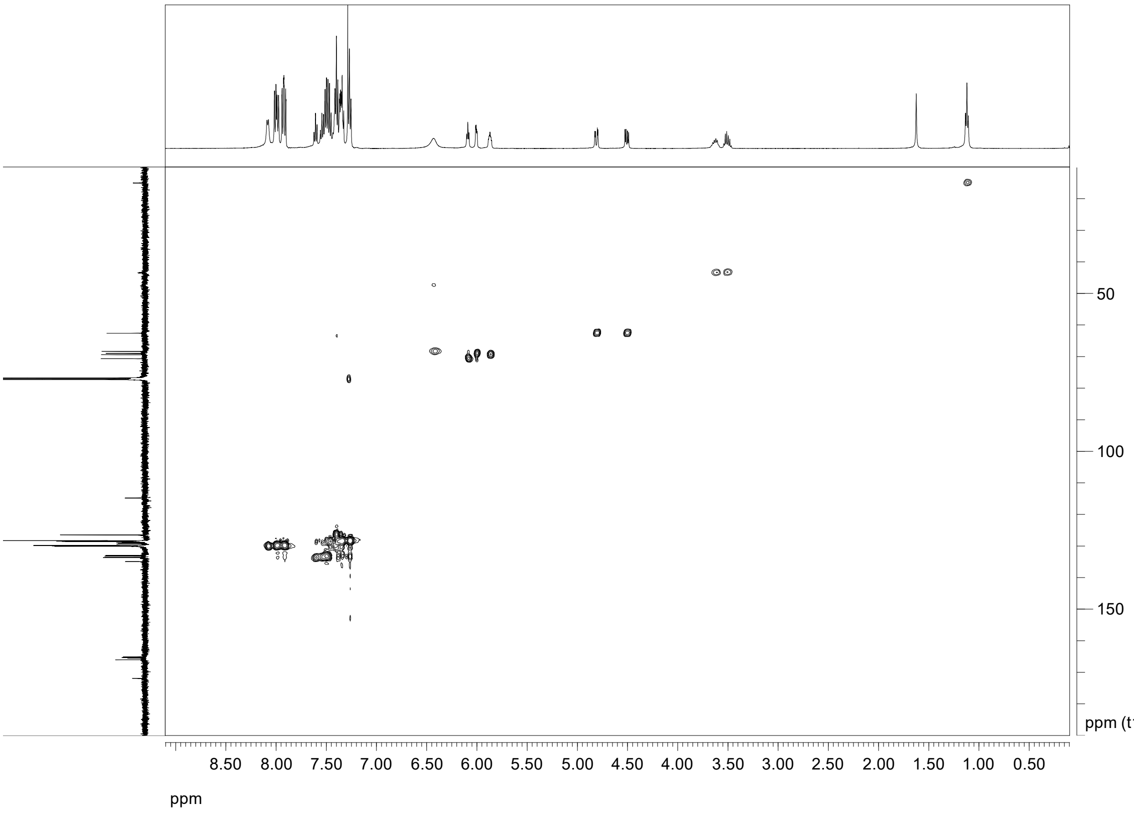

Figure S57. HMQC spectrum of **20** in $CDCl_3$.

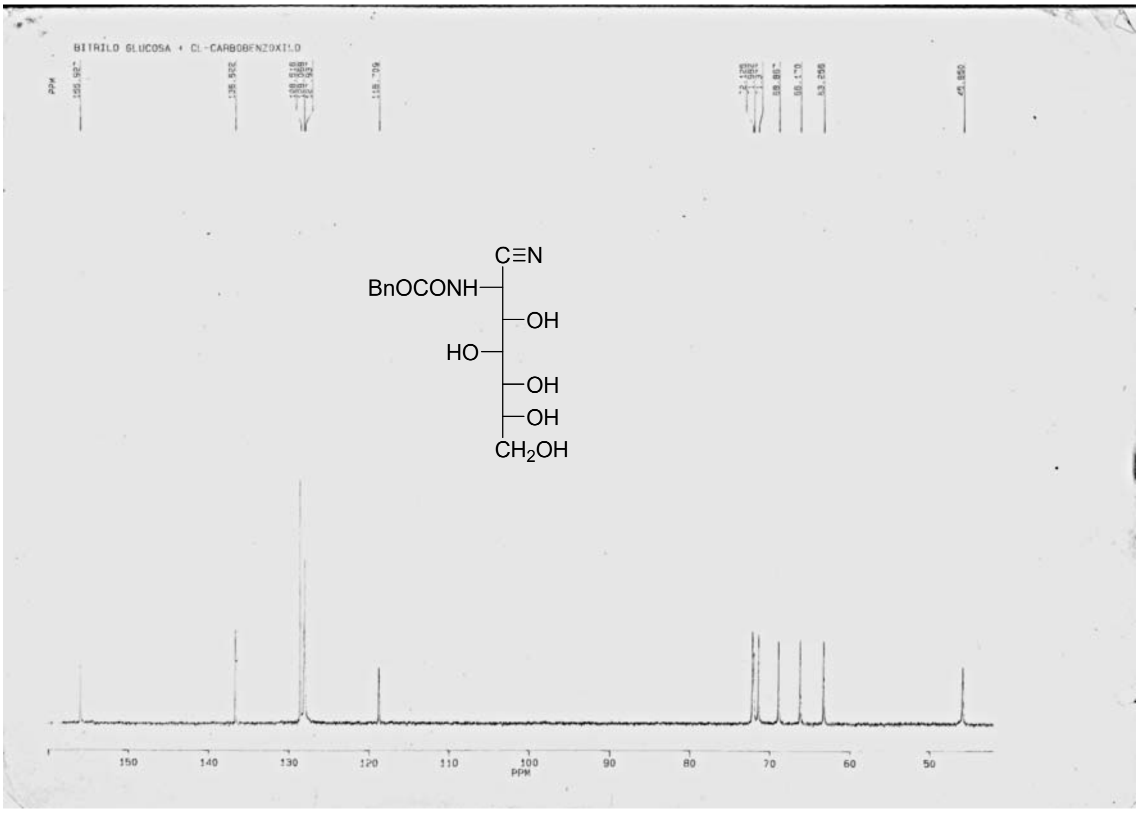

Figure S58. $^{13}C\{^1H\}$ NMR spectrum of **26** in DMSO-d_6.

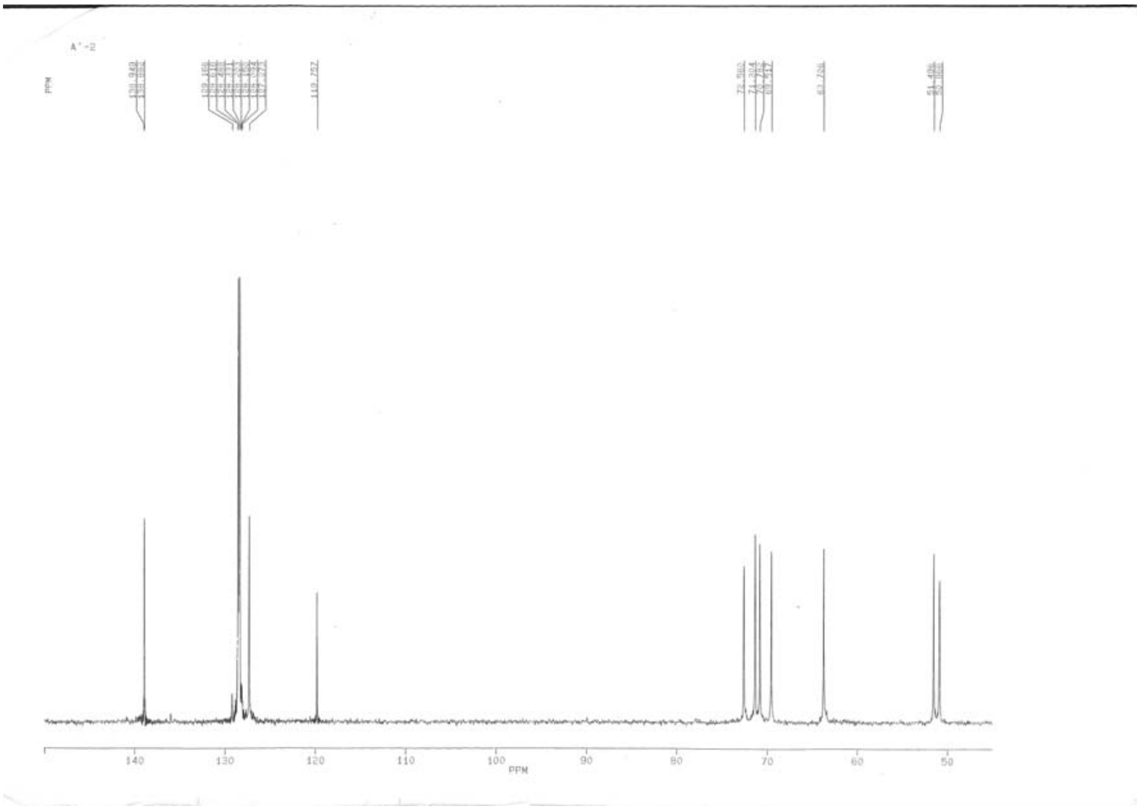

Figure S59. $^{13}C\{^1H\}$ NMR spectrum of **33** in $CDCl_3$.

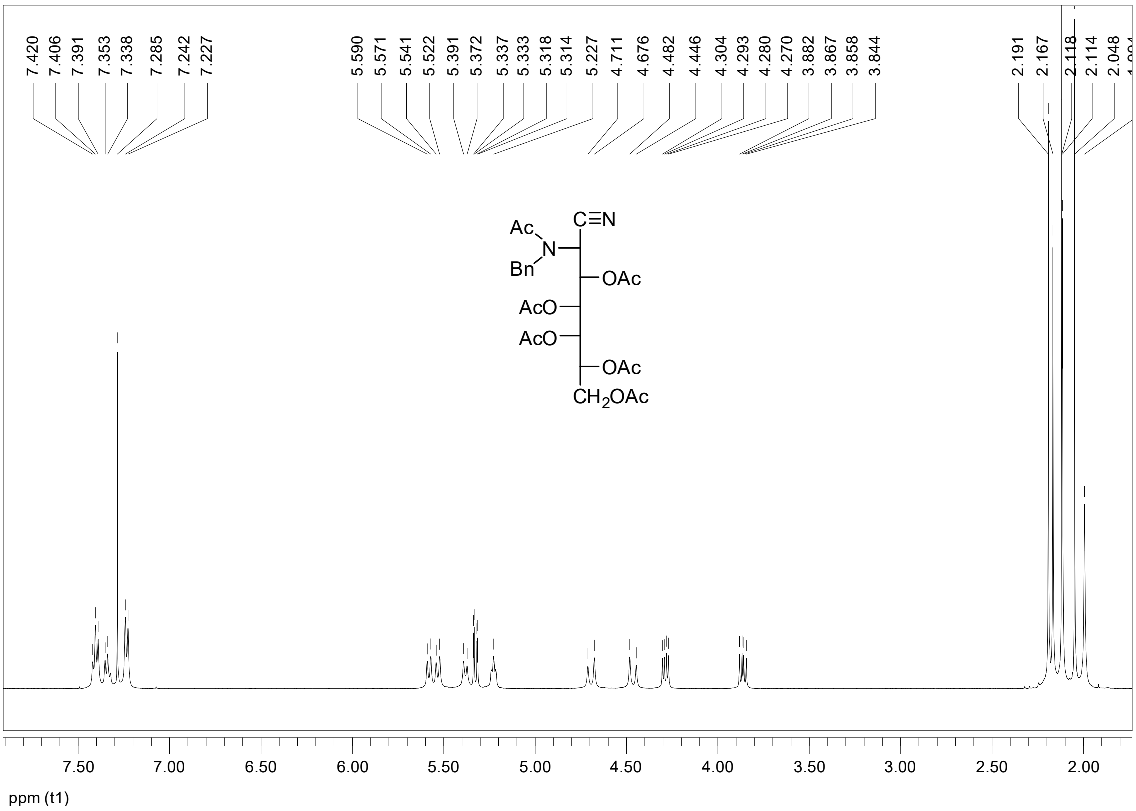

Figure S60. ^{1}H NMR spectrum of **37** in $CDCl_3$.

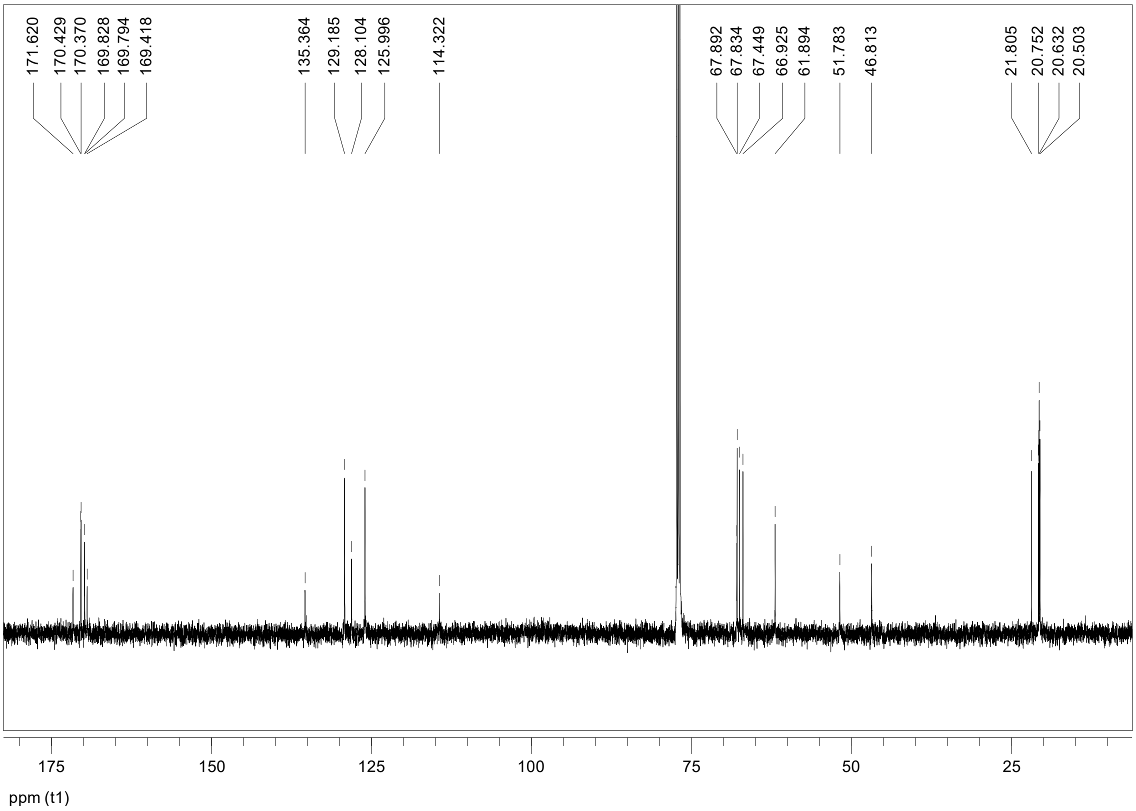

Figure S61. $^{13}C\{^{1}H\}$ NMR spectrum of **37** in $CDCl_3$.

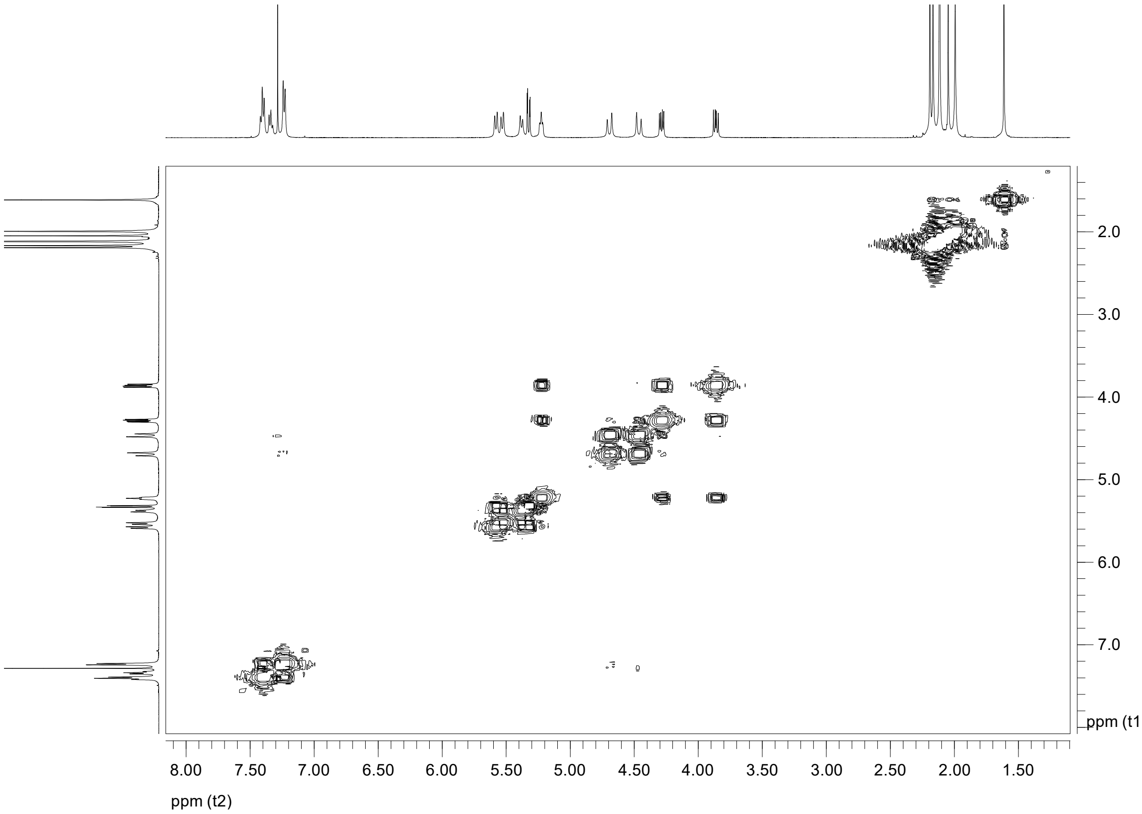

Figure S62. COSY spectrum of **37** in $CDCl_3$.

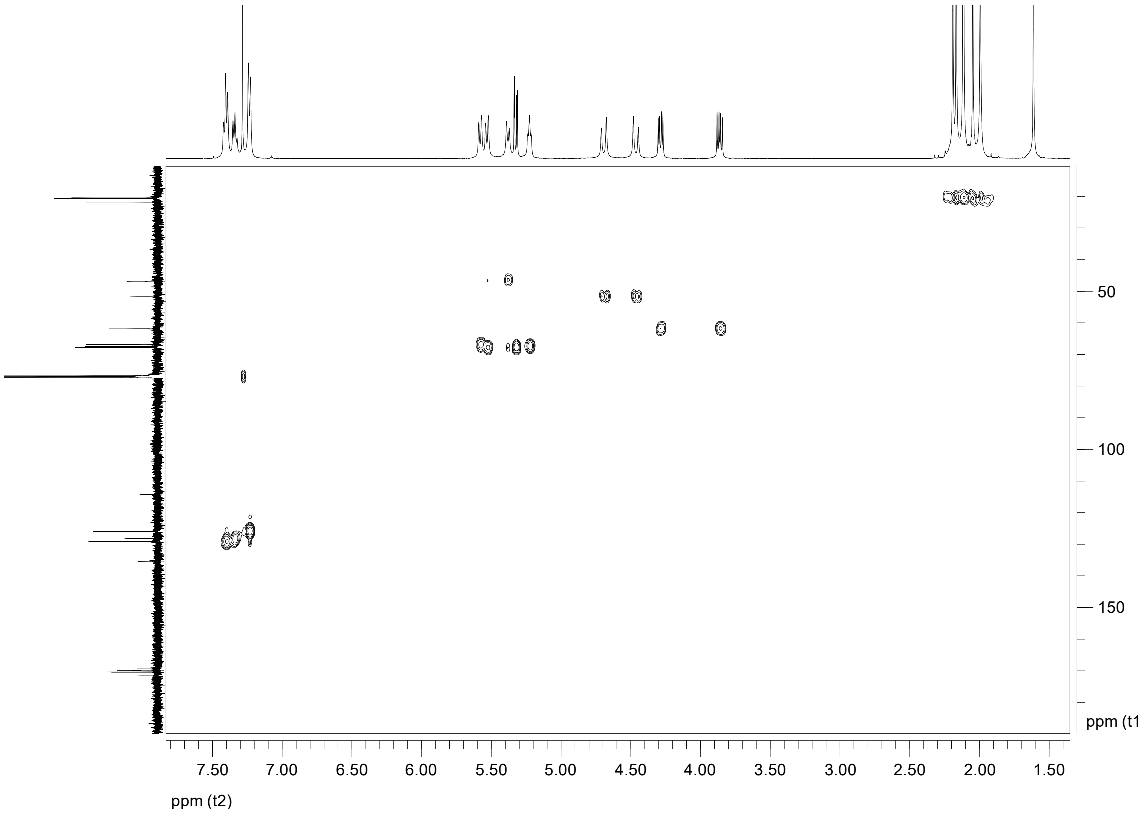

Figure S63. HMQC spectrum of **37** in $CDCl_3$.

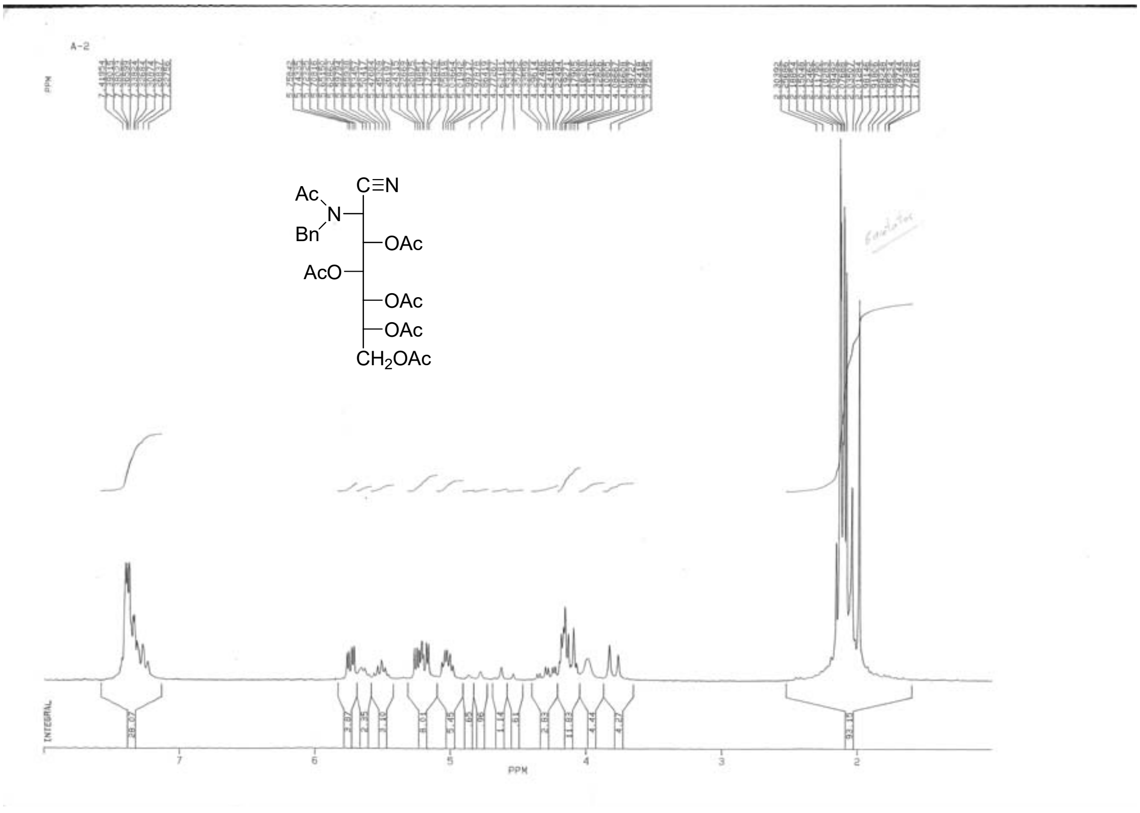

Figure S64. ^{1}H NMR spectrum of **38** in $CDCl_3$.

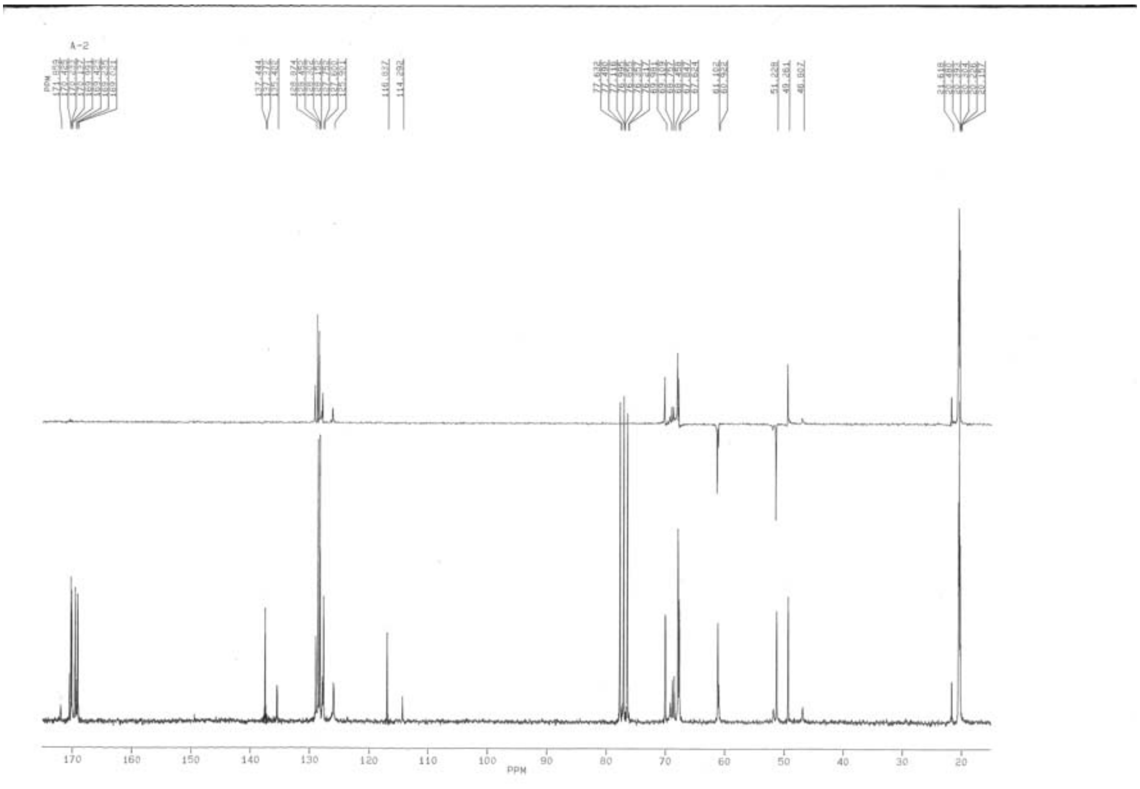

Figure S65. $^{13}C\{^1H\}$ NMR and DEPT spectra of **38** in $CDCl_3$

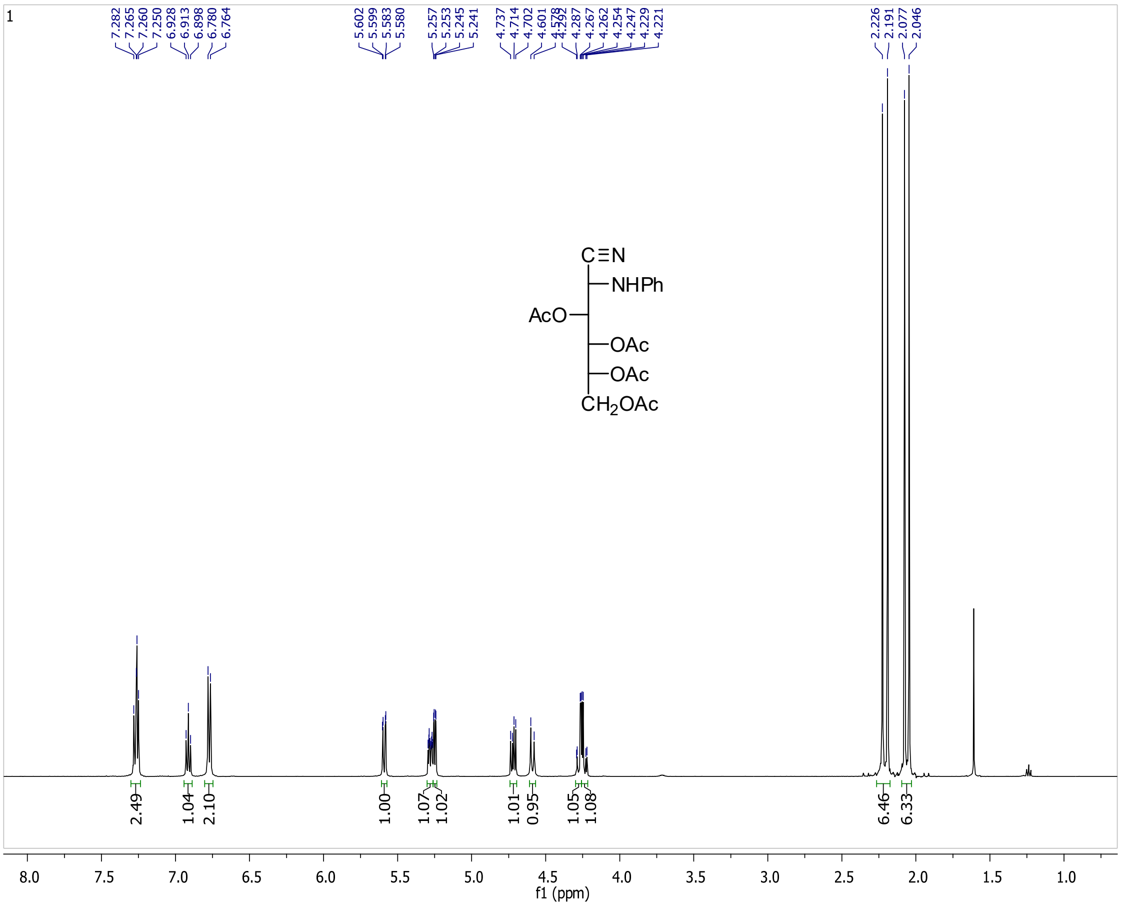

Figure S66. ^{1}H NMR spectrum of **42** in $CDCl_3$.

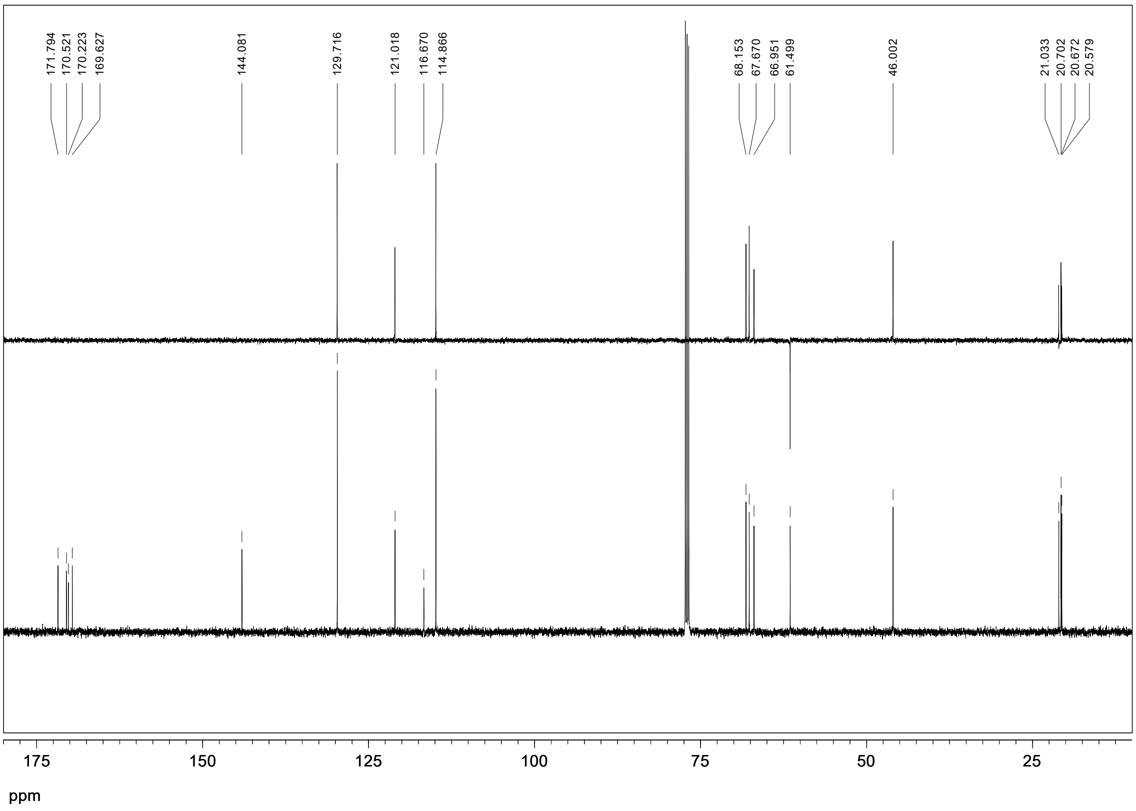

Figure S67. $^{13}C\{^1H\}$ NMR and DEPT spectra of **42** in $CDCl_3$.

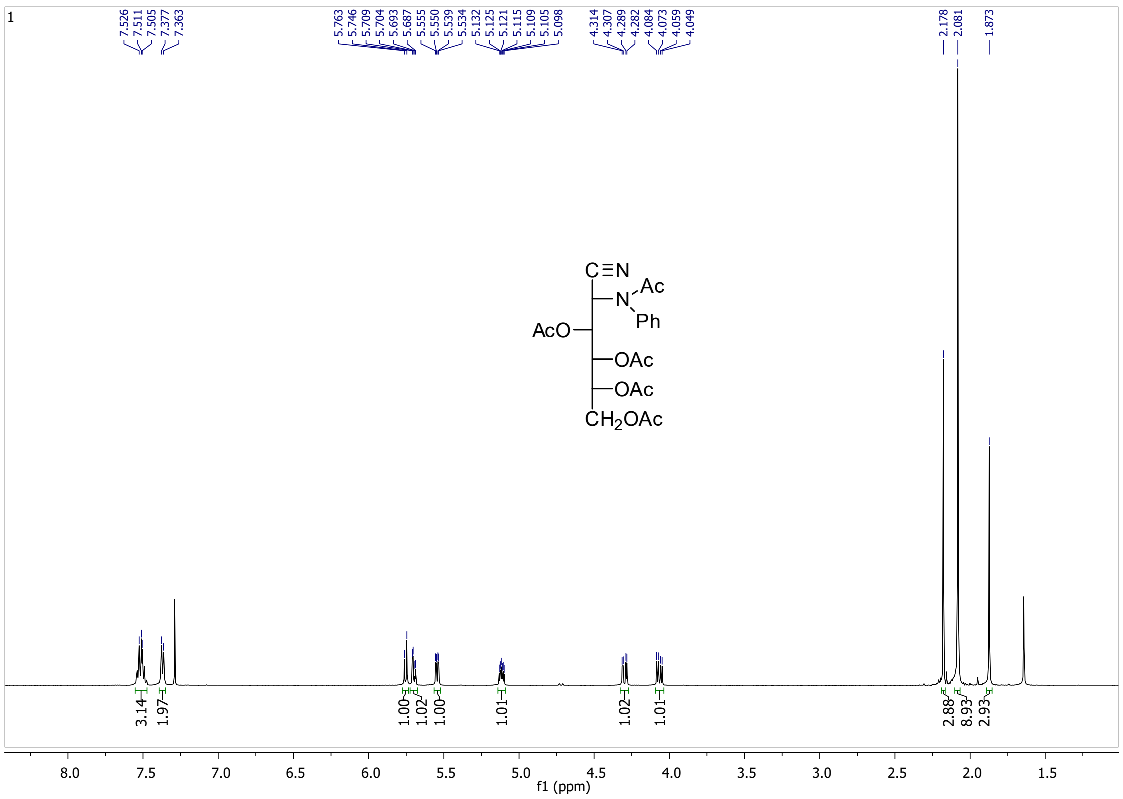

Figure S68. ^{1}H NMR spectrum of **43** in $CDCl_3$.

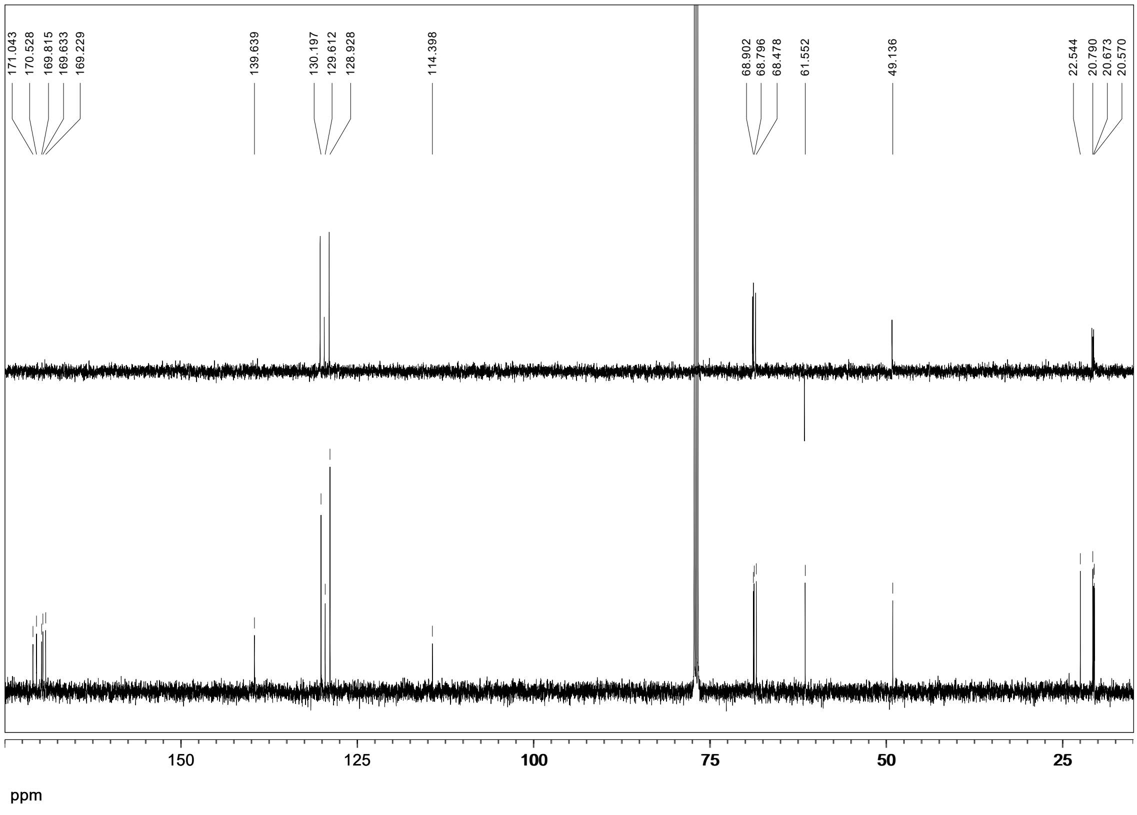

Figure S69. $^{13}C\{^{1}H\}$ NMR and DEPT spectra of **43** in $CDCl_3$.

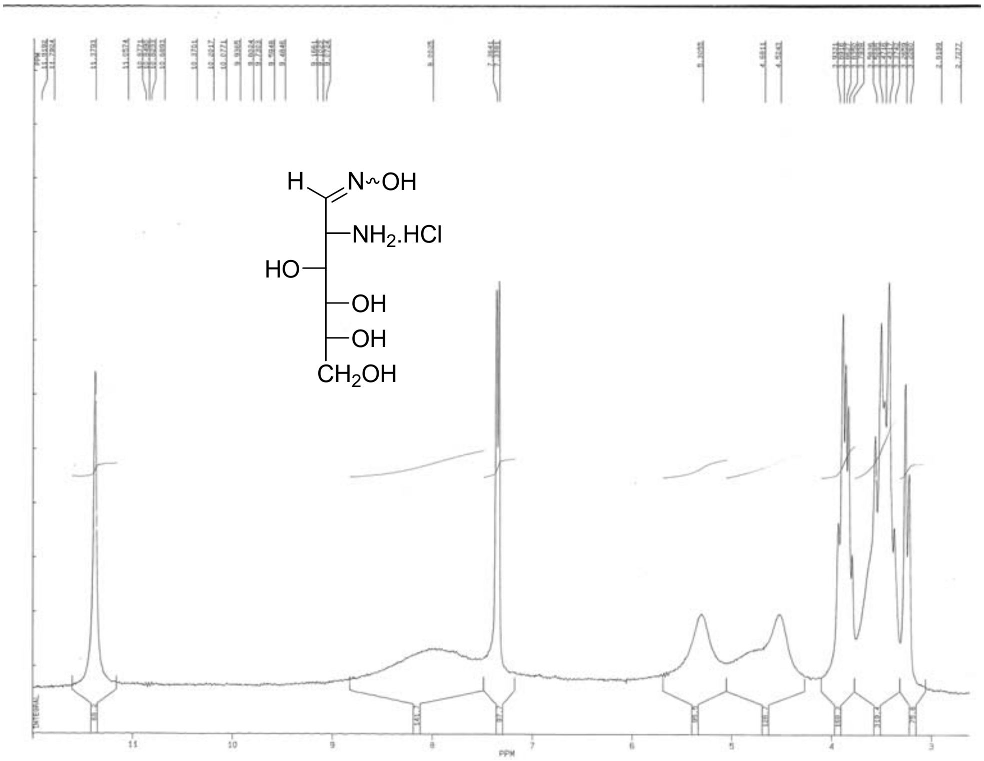

Figure S70. ^{1}H NMR spectrum of **45** in DMSO-d_6.

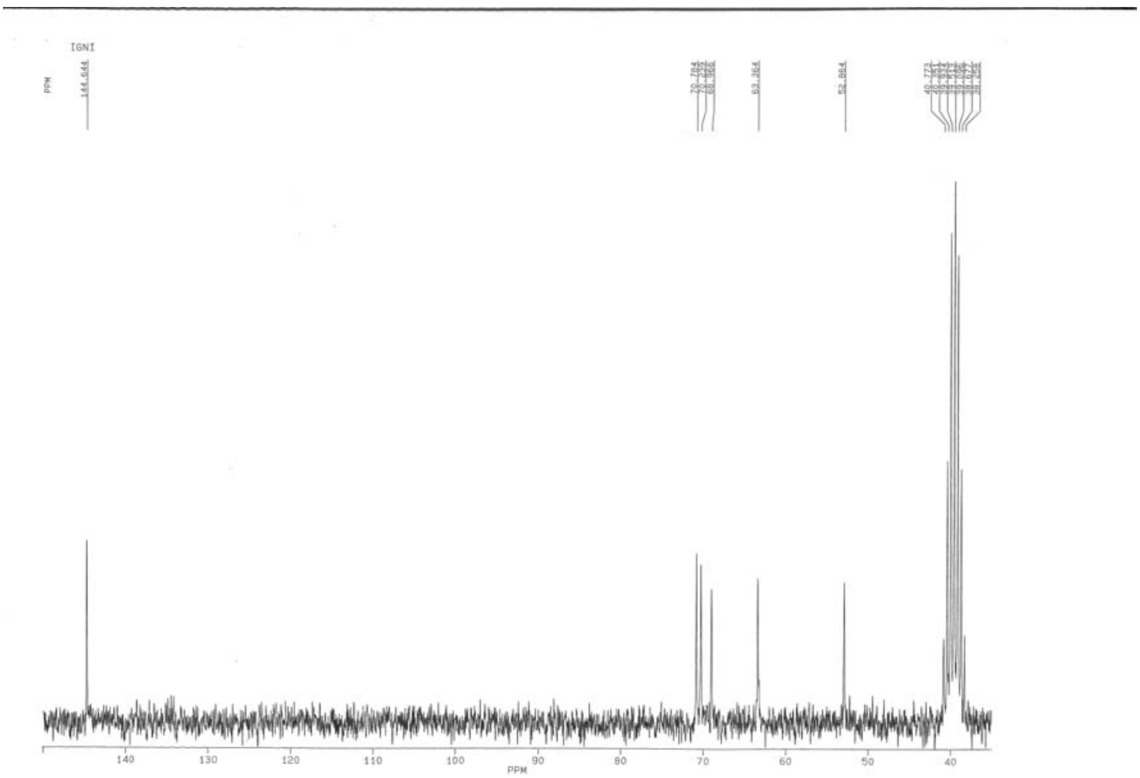

Figure S71. $^{13}C\{^{1}H\}$ NMR spectrum of **45** in DMSO-d_6..

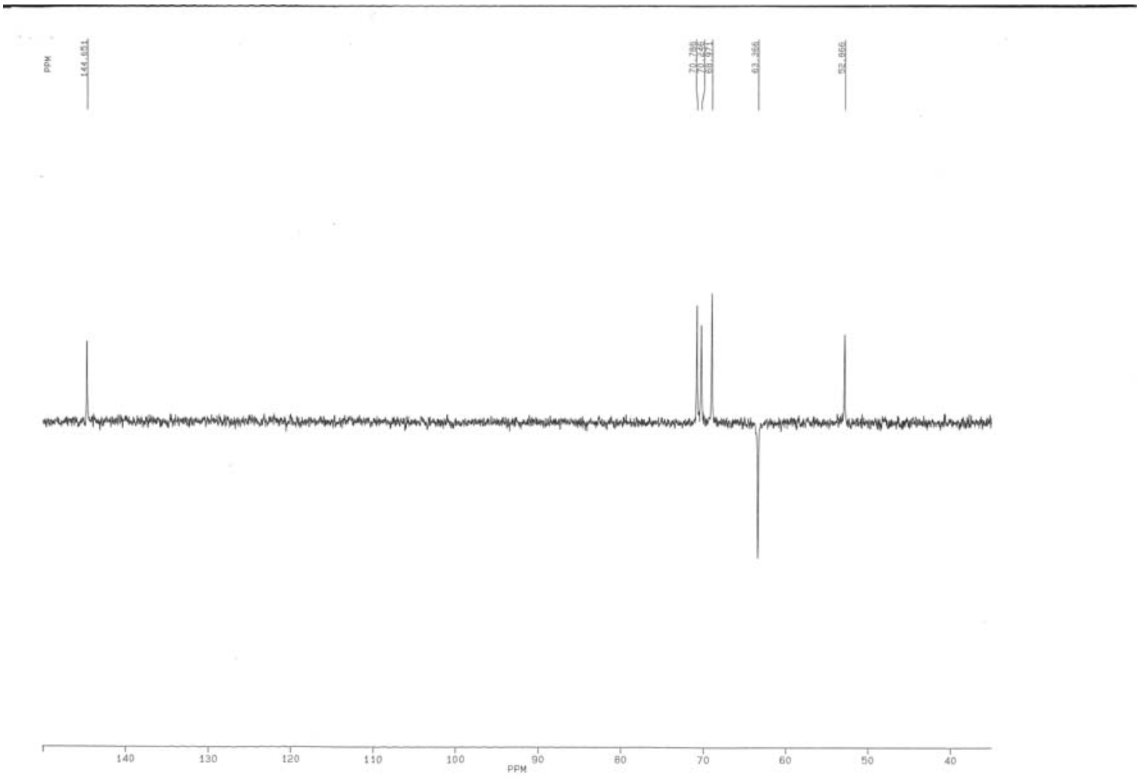

Figure S72. DEPT spectrum of **45** in DMSO-d_6.

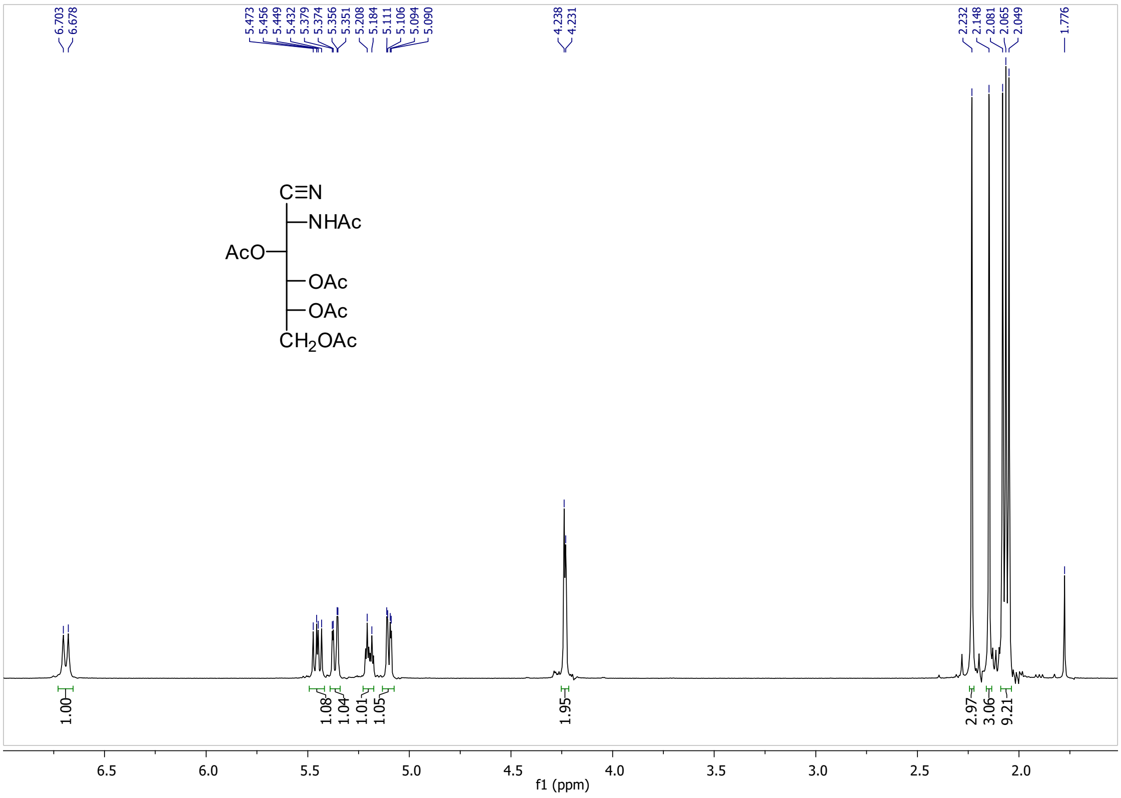

Figure S73. ^{1}H NMR spectrum of **47** in $CDCl_3$.

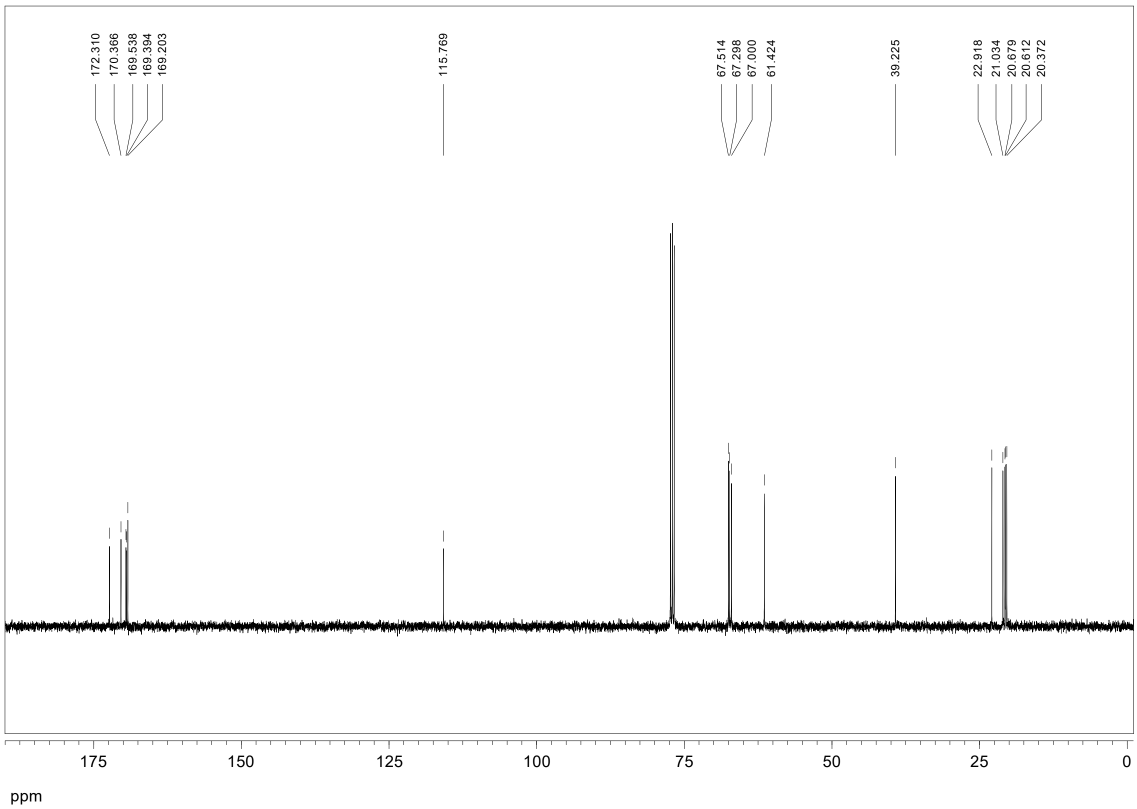

Figure S74. $^{13}C\{^1H\}$ NMR spectrum of **47** in $CDCl_3$.

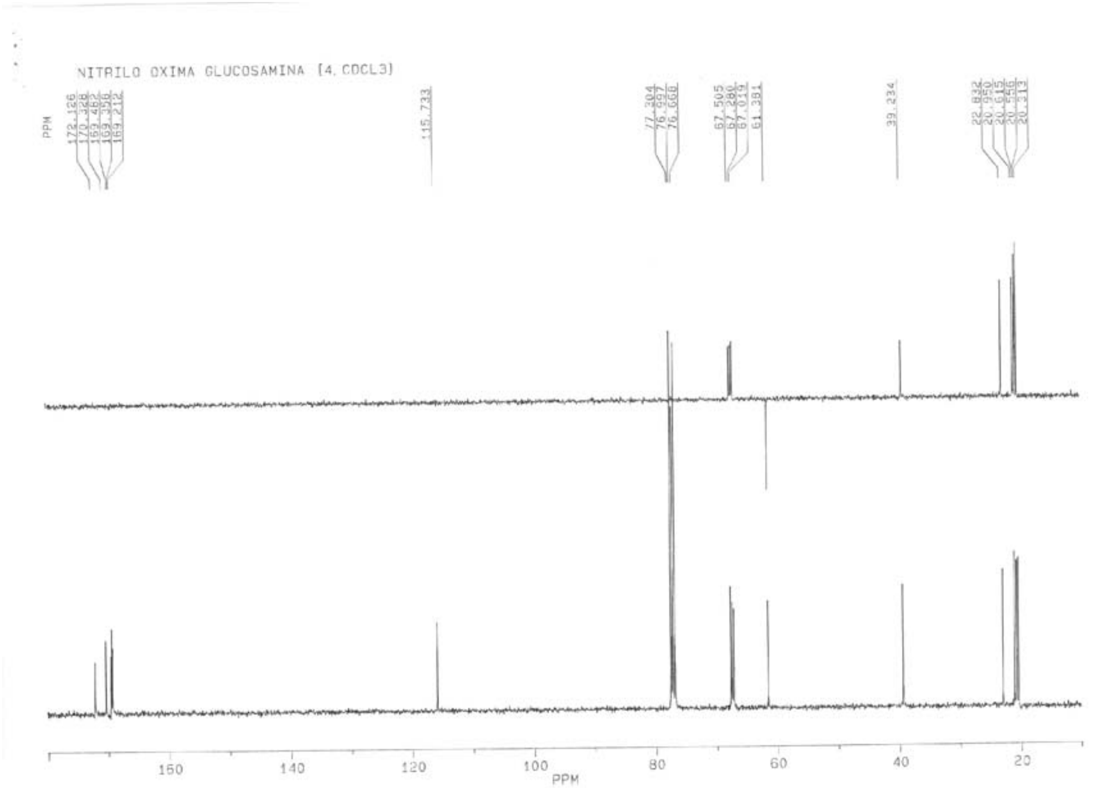

Figure S75. $^{13}C\{^1H\}$ NMR and DEPT spectra of **47** in $CDCl_3$.

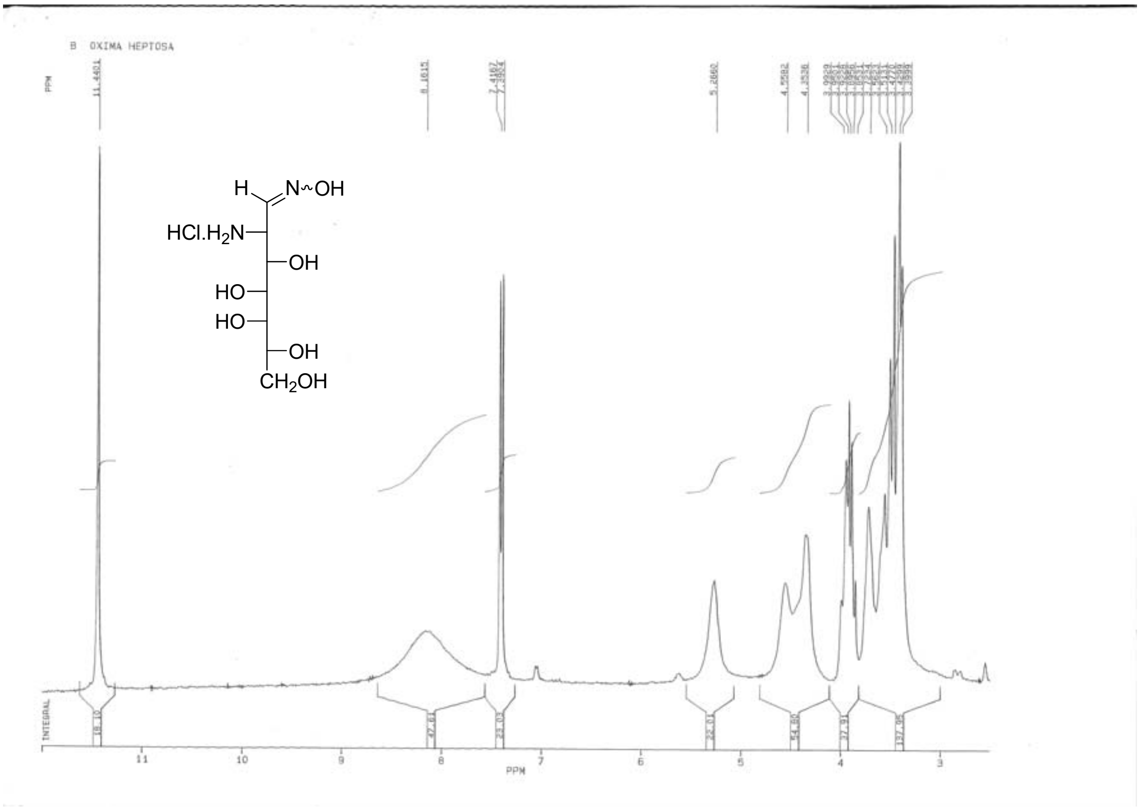

Figure S76. ^{1}H NMR spectrum of **49** in DMSO-*d*6.

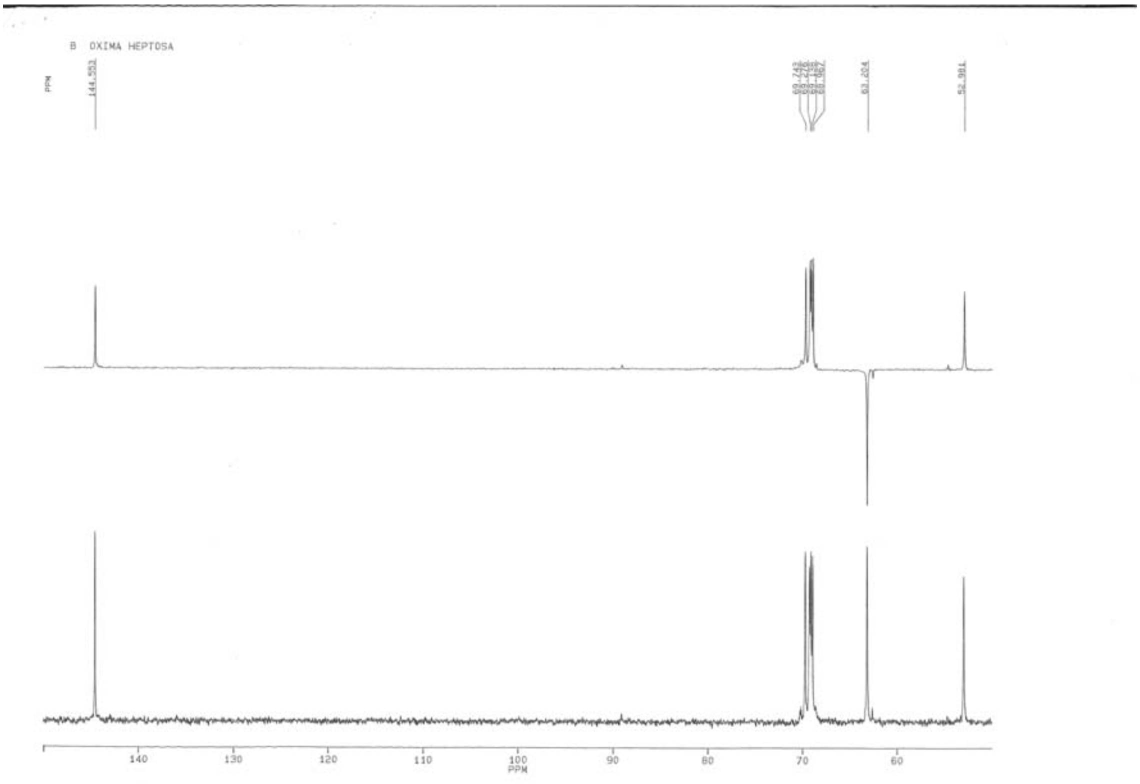

Figure S77. $^{13}C\{^{1}H\}$ NMR and DEPT spectra of **49** in DMSO-*d*6..

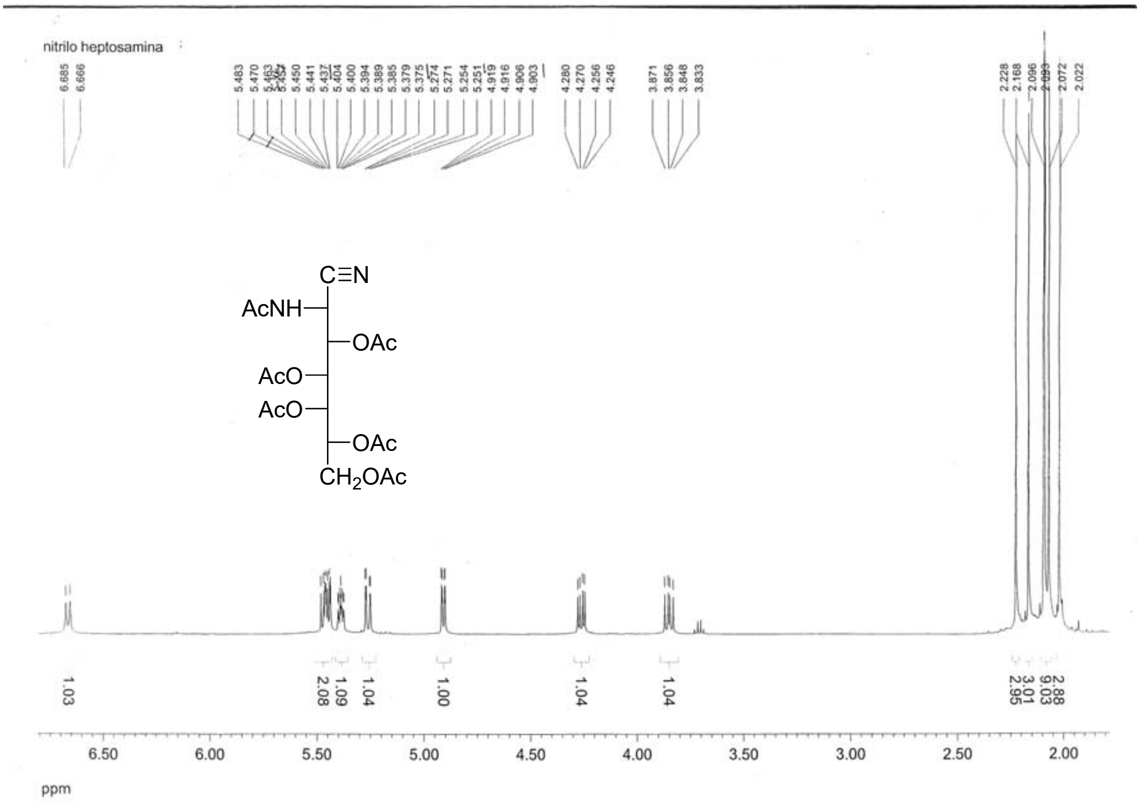

Figure S78. ^{1}H NMR spectrum of **51** in $CDCl_3$.

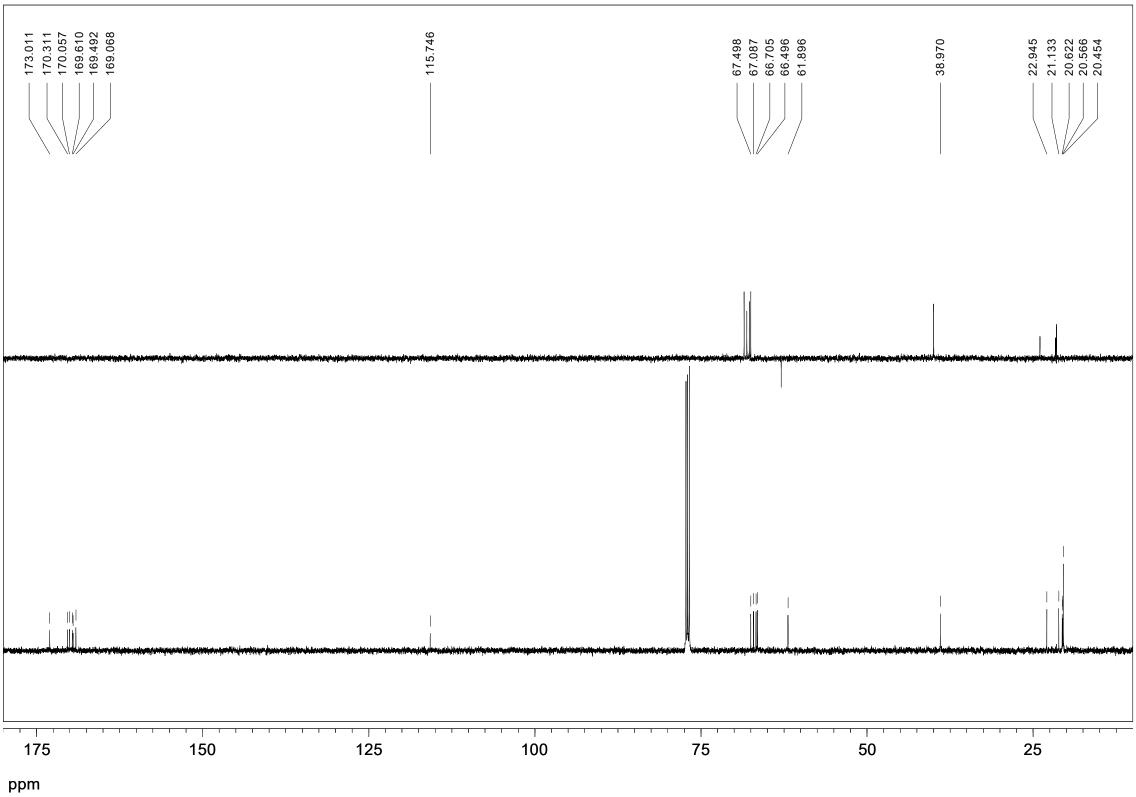

Figure S79. $^{13}C\{^{1}H\}$ NMR and DEPT spectra of **51** in $CDCl_3$.

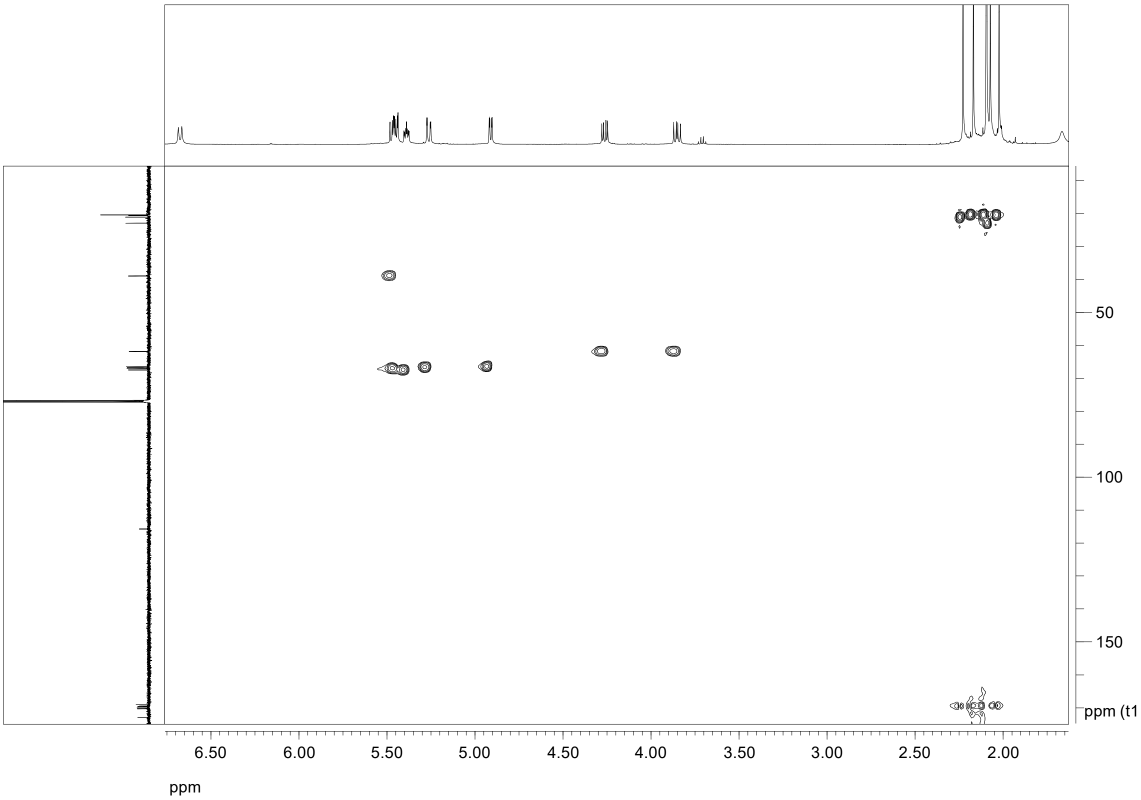

Figure S80. HMQC spectrum of **51** in $CDCl_3$.

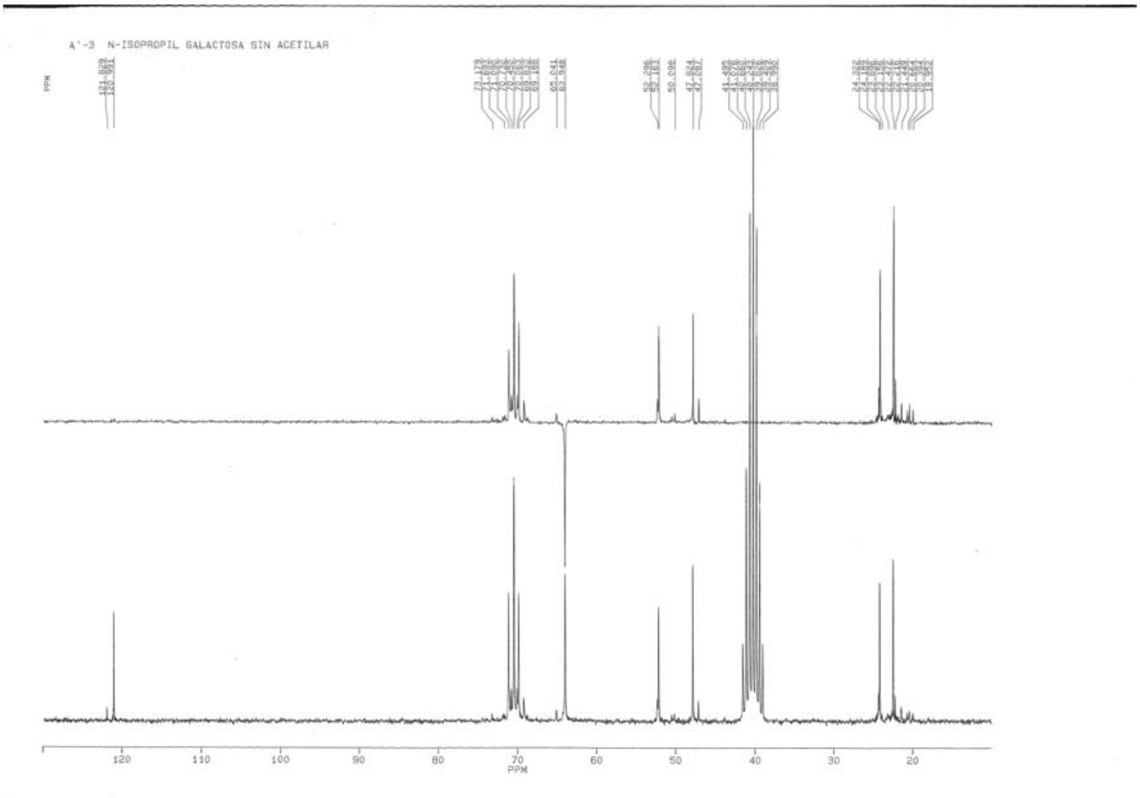

Figure S81. $^{13}C\{^{1}H\}$ NMR and DEPT spectra of **54** in DMSO-d_6.

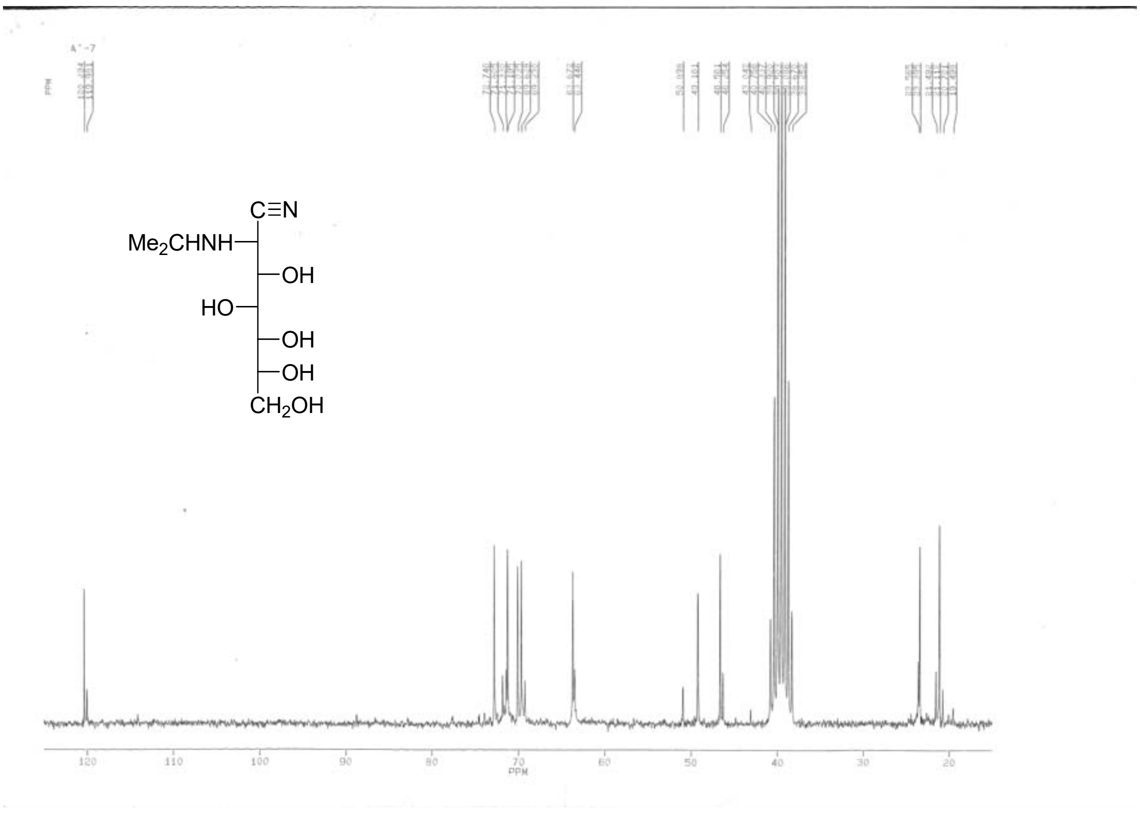

Figure S82. $^{13}C\{^{1}H\}$ NMR spectrum of **55** in DMSO-d_6.

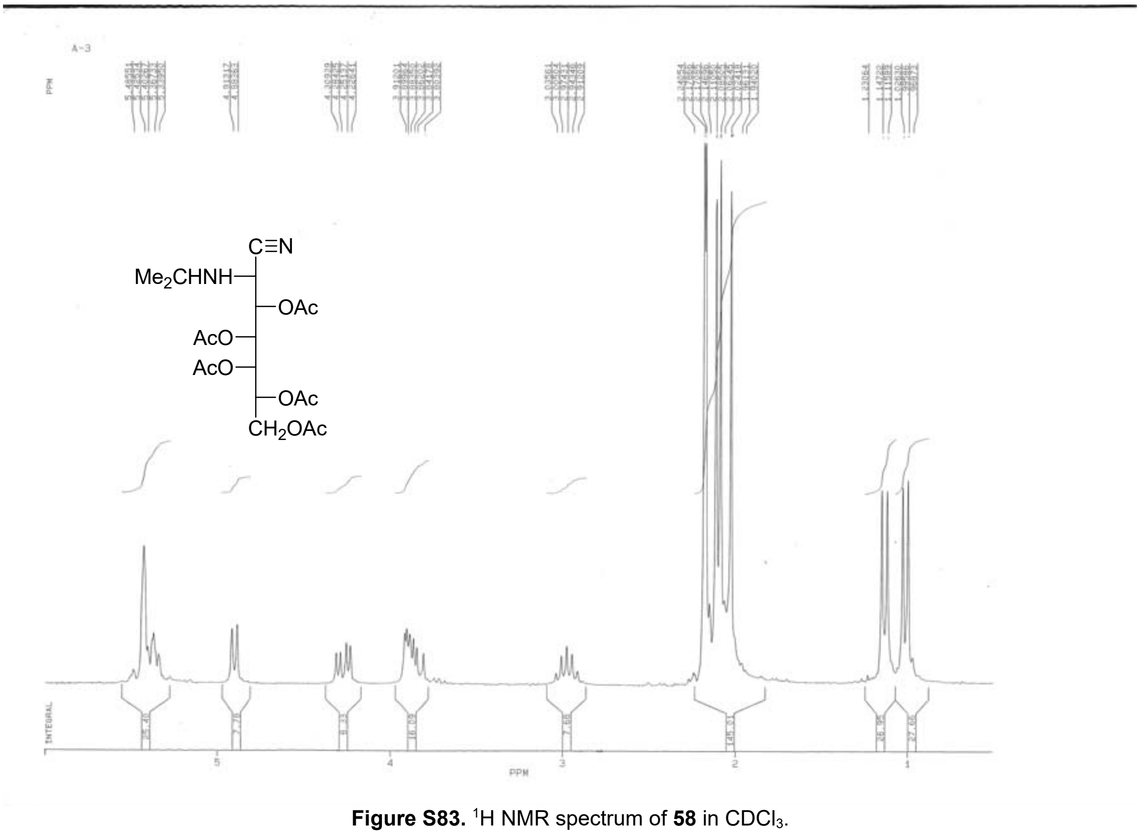

Figure S83. ^{1}H NMR spectrum of **58** in $CDCl_3$.

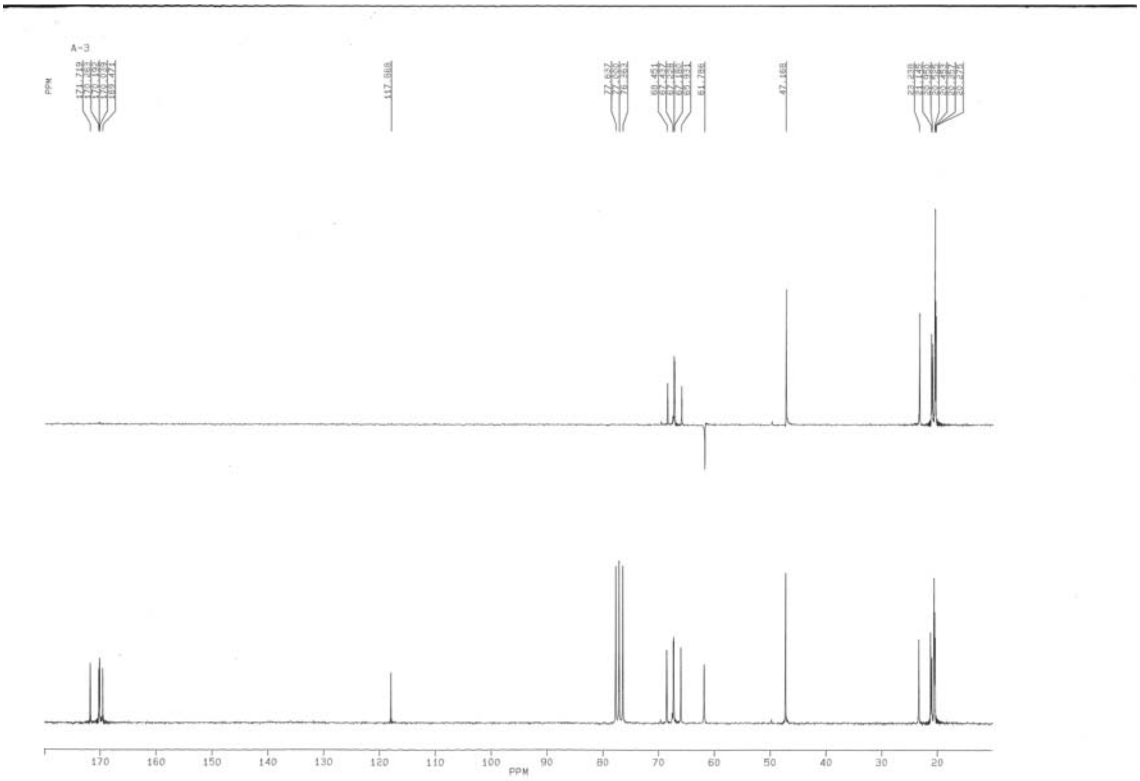

Figure S84. $^{13}C\{^1H\}$ NMR and DEPT spectra of **58** in $CDCl_3$.

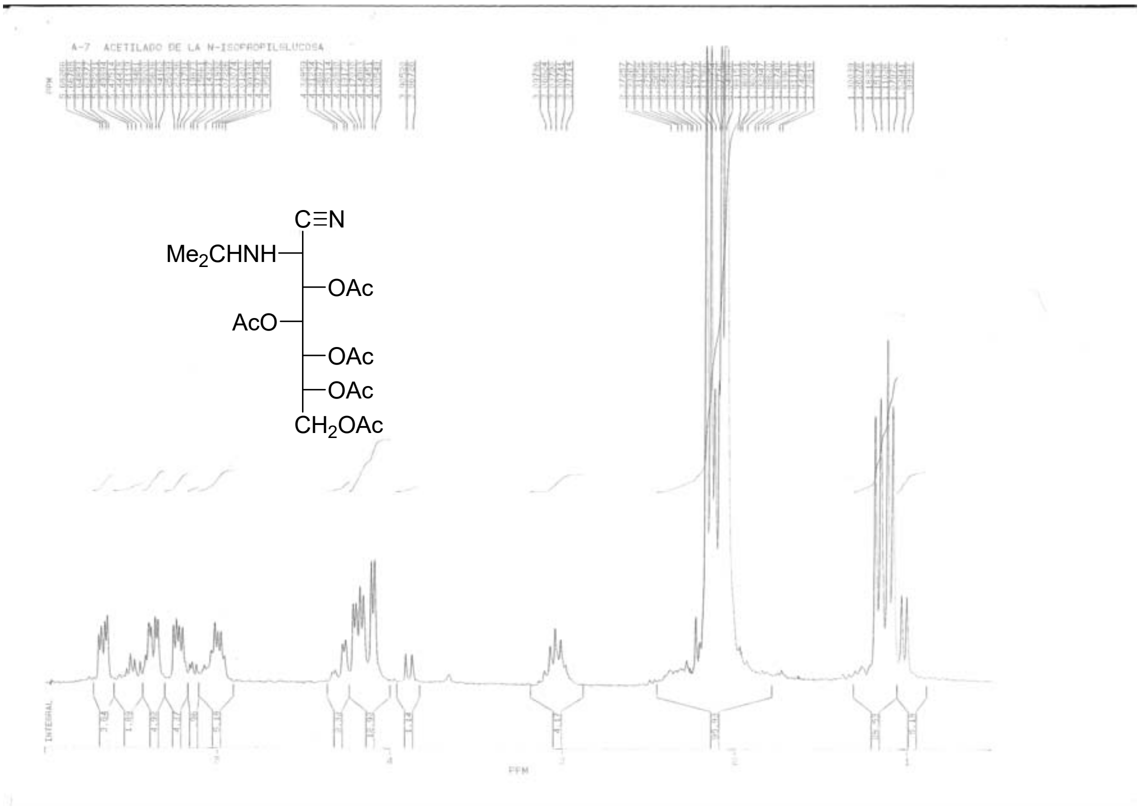

Figure S85. ^1H NMR spectrum of **59** and **61** in $CDCl_3$

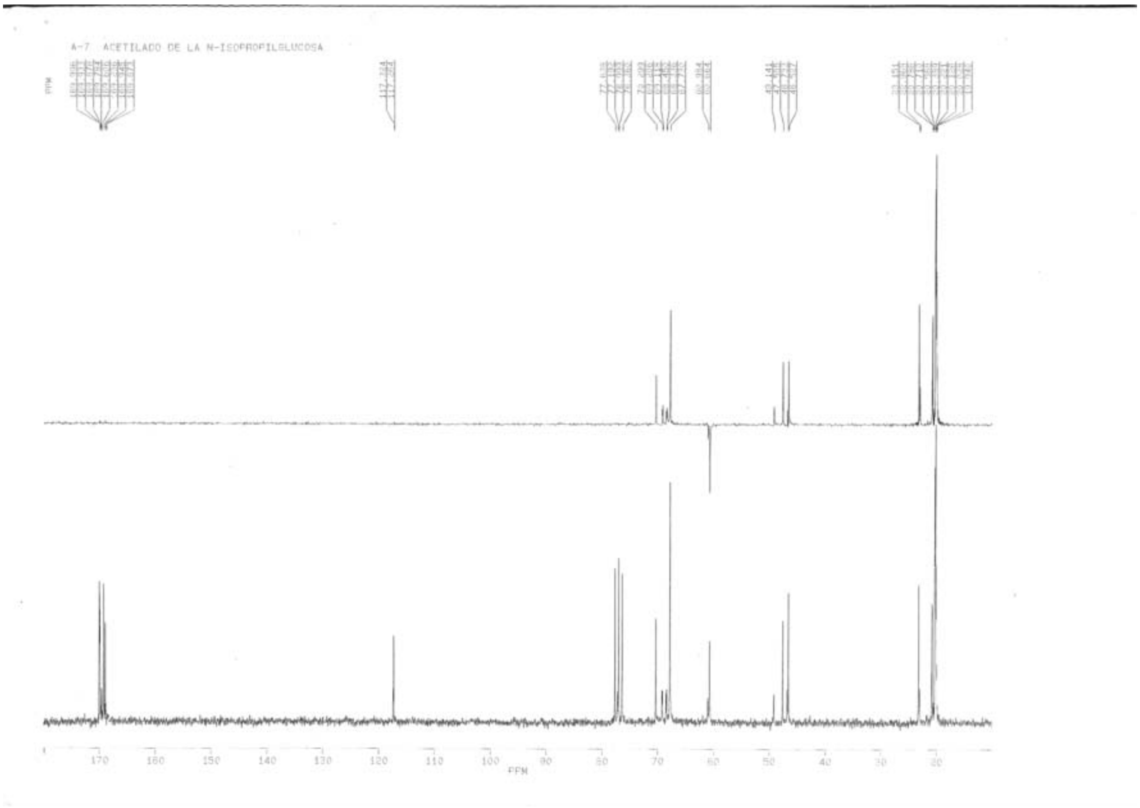

Figure S86. $^{13}C\{^1H\}$ NMR and DEPT spectra of **59** and **61** in $CDCl_3$

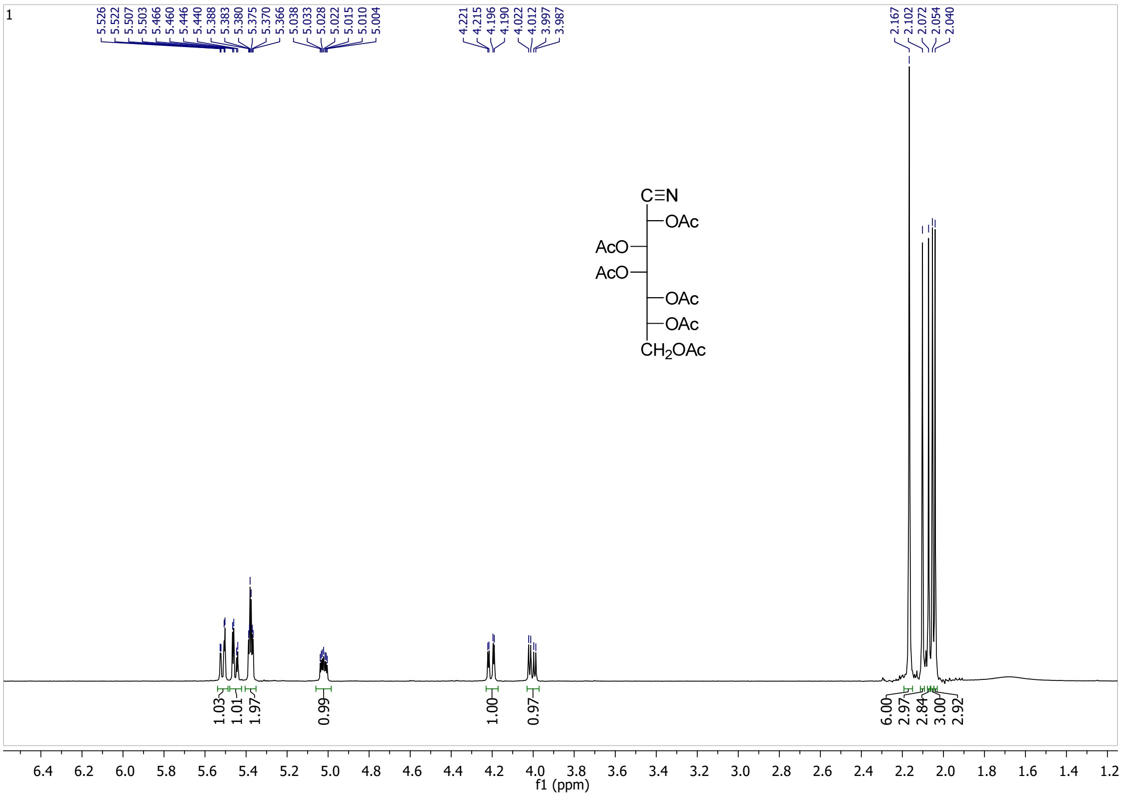

Figure S87. ^1H NMR spectrum of **63** in $CDCl_3$

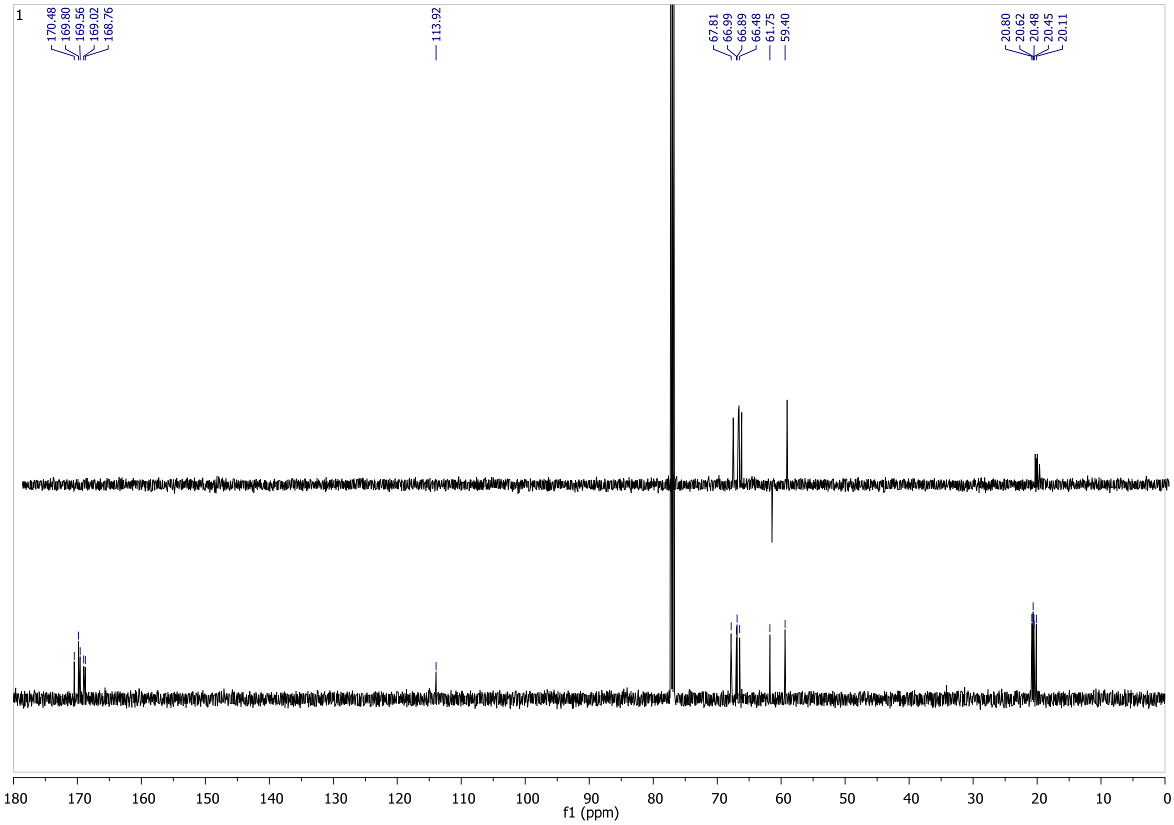

Figure S88. $^{13}C\{^{1}H\}$ NMR and DEPT spectra of **63** in $CDCl_3$

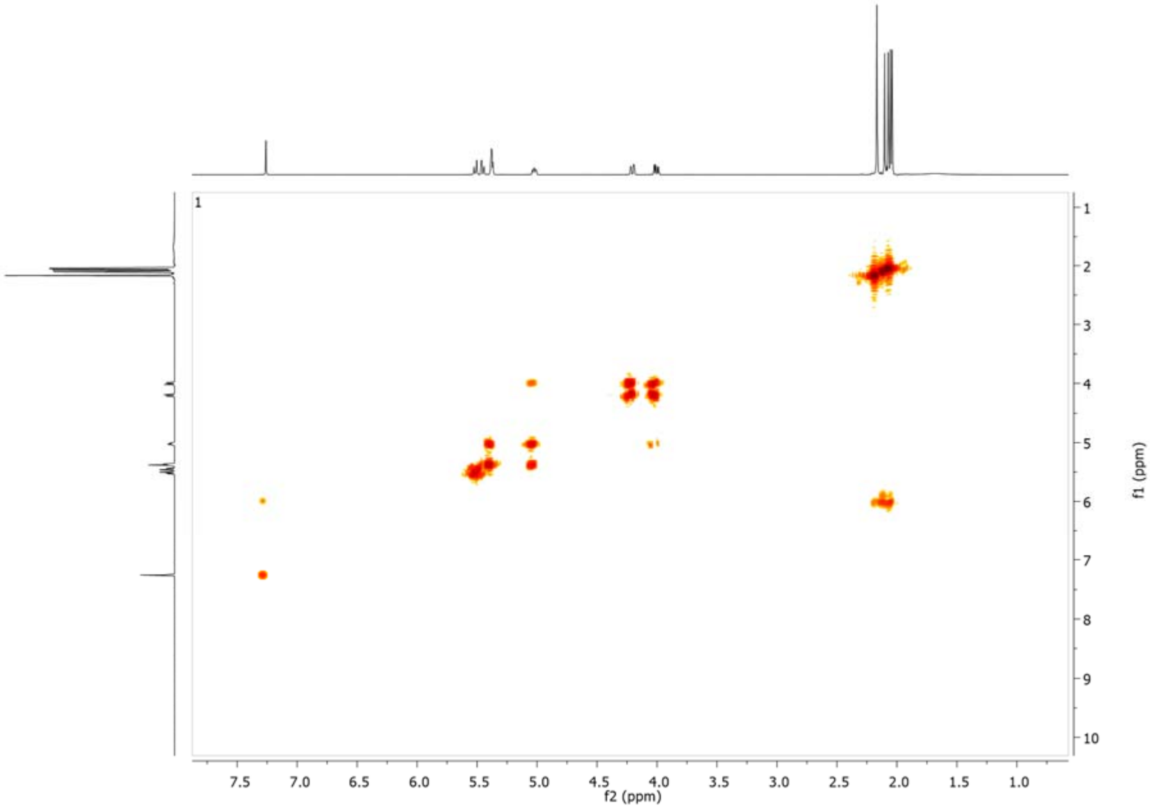

Figure S89. COSY spectrum of **63** in $CDCl_3$

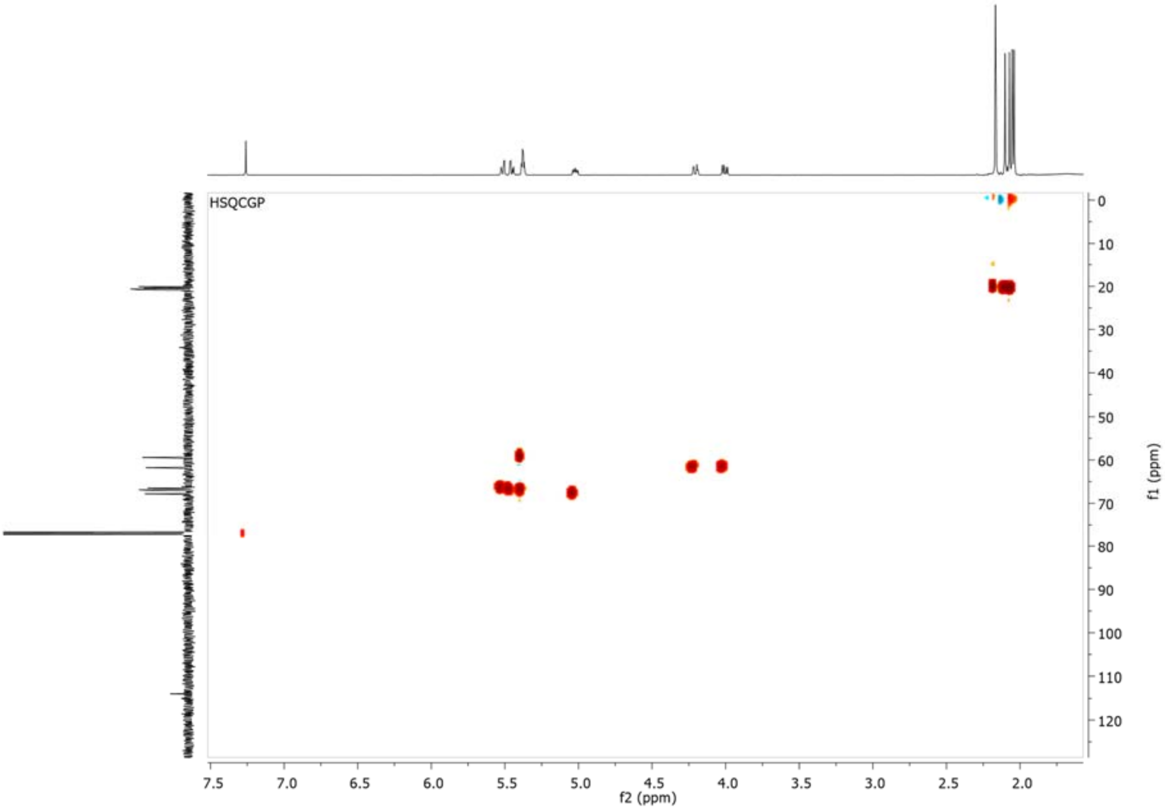

Figure S90. HMQC spectrum of **63** in $CDCl_3$

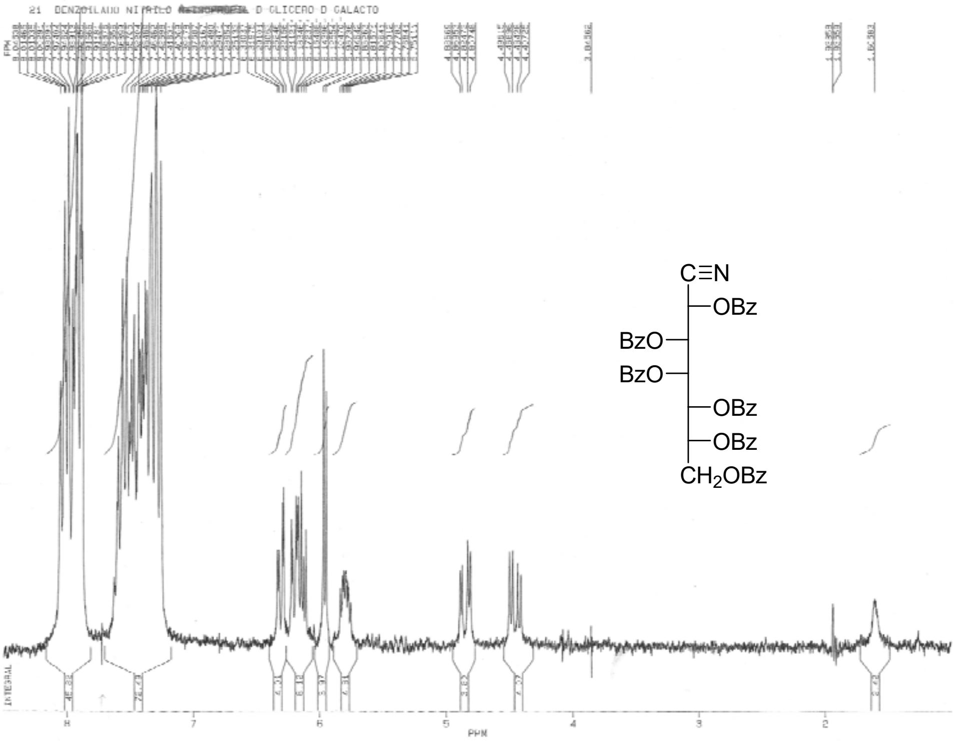

Figure S91. ^1H NMR spectrum of **64** in $CDCl_3$

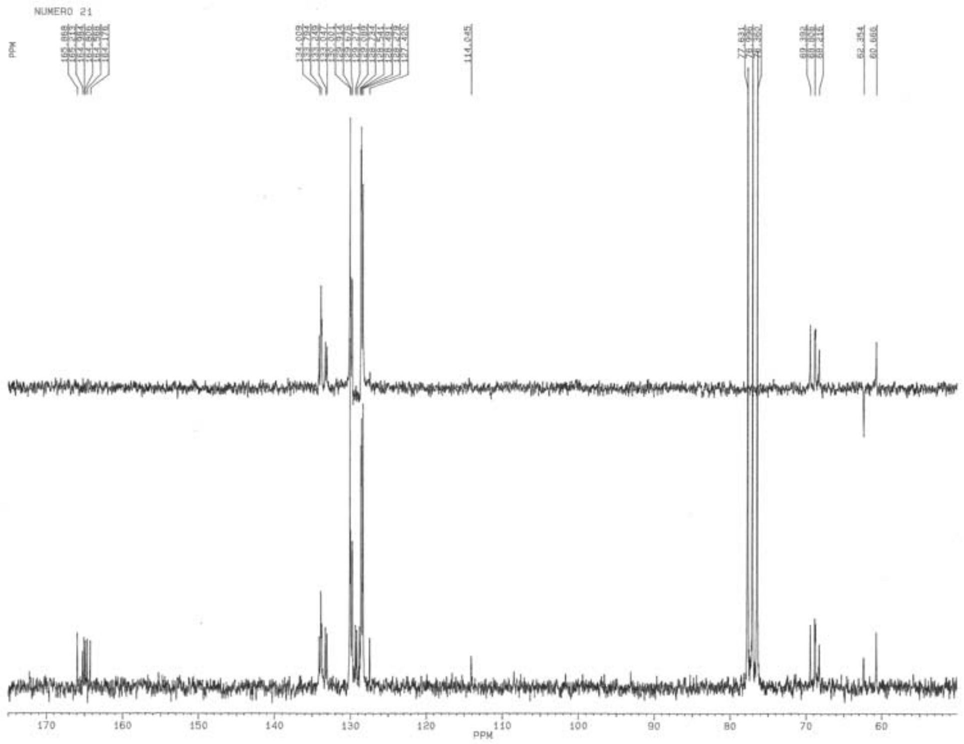

Figure S92. $^{13}C\{^{1}H\}$ NMR and DEPT spectra of **64** in $CDCl_3$

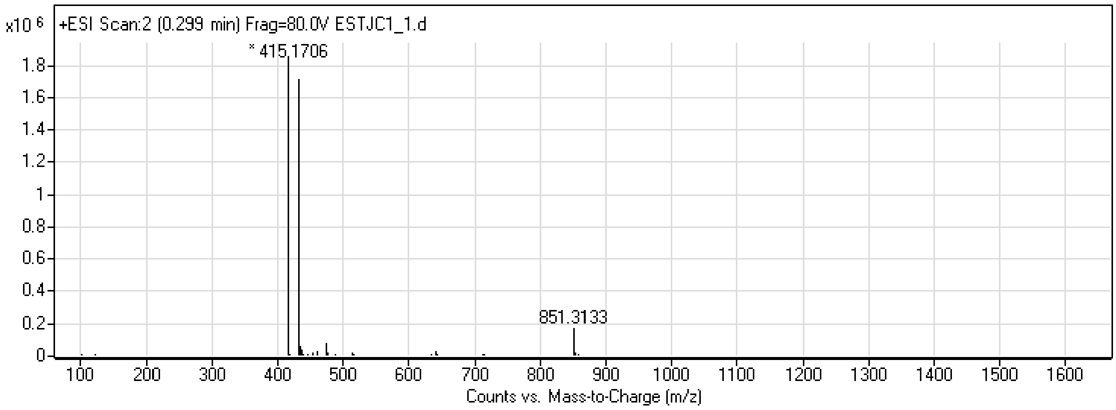

Figure S93. Mass spectrum of **11**.

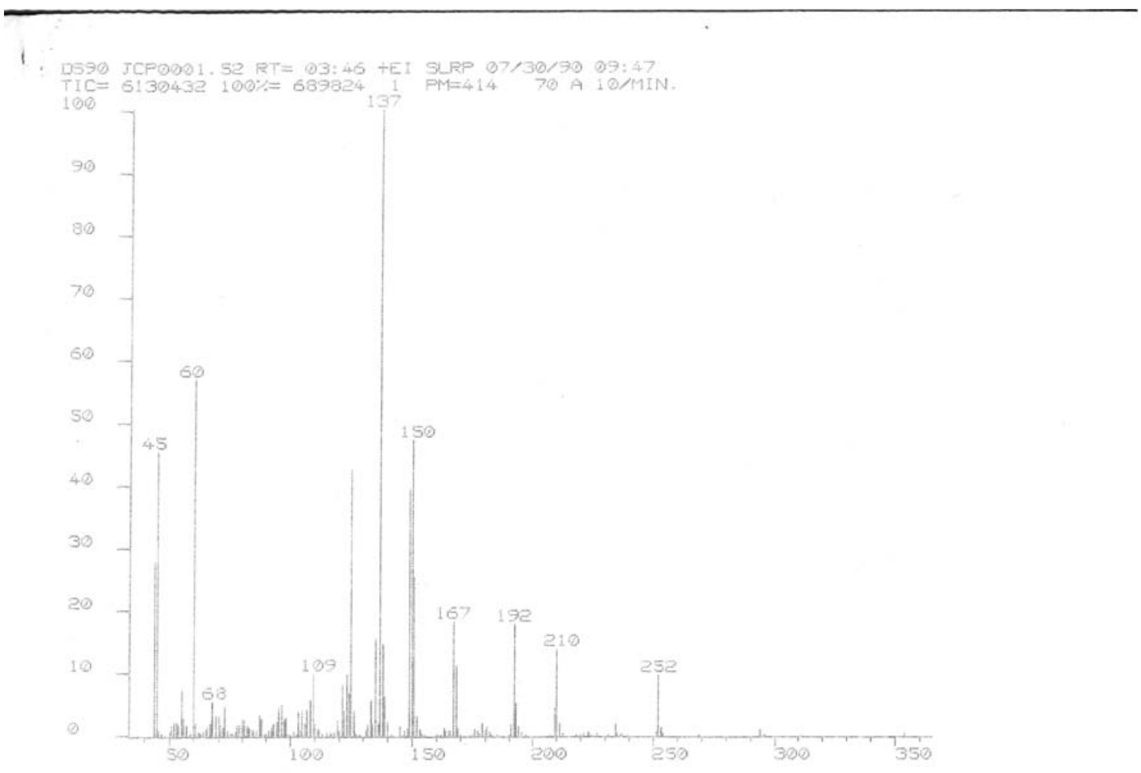

Figure S94. Mass spectrum (EI) of **11**.

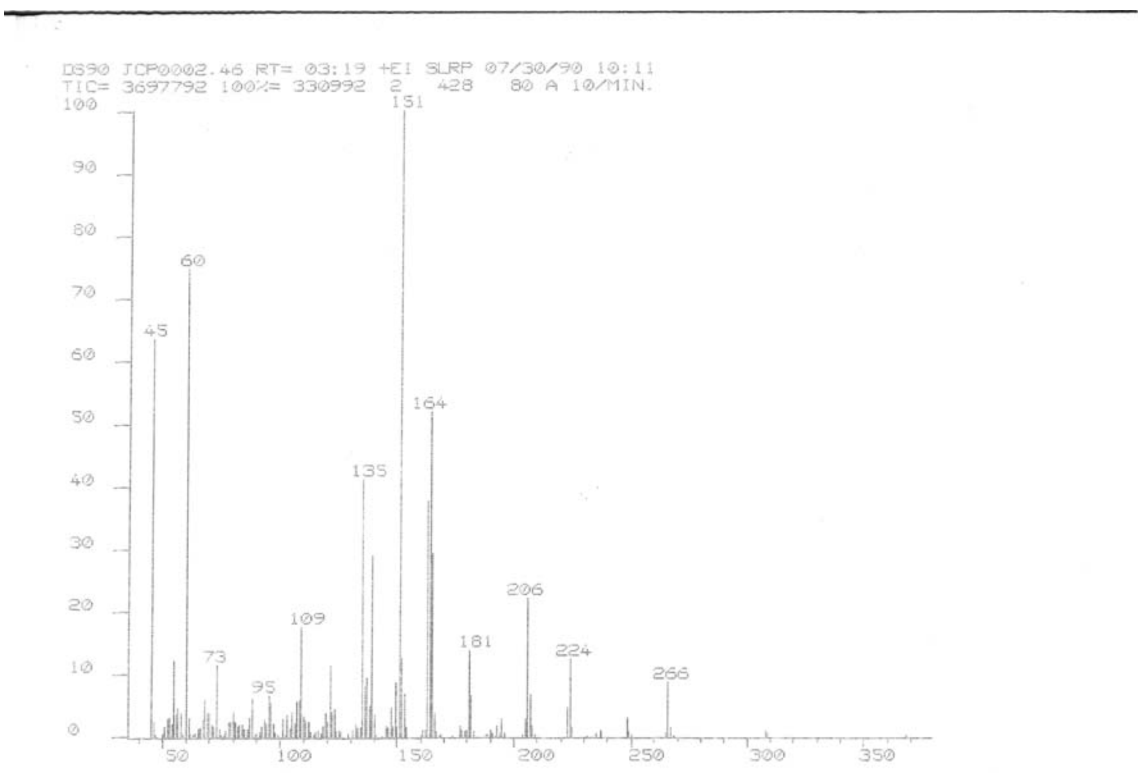

Figure S95. Mass spectrum (EI) of **12**.

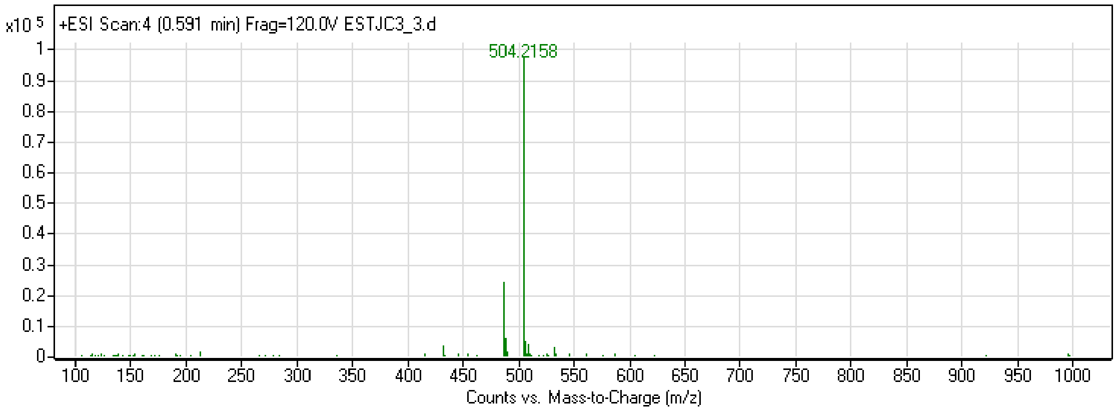

Figure S96. Mass spectrum of **13**.

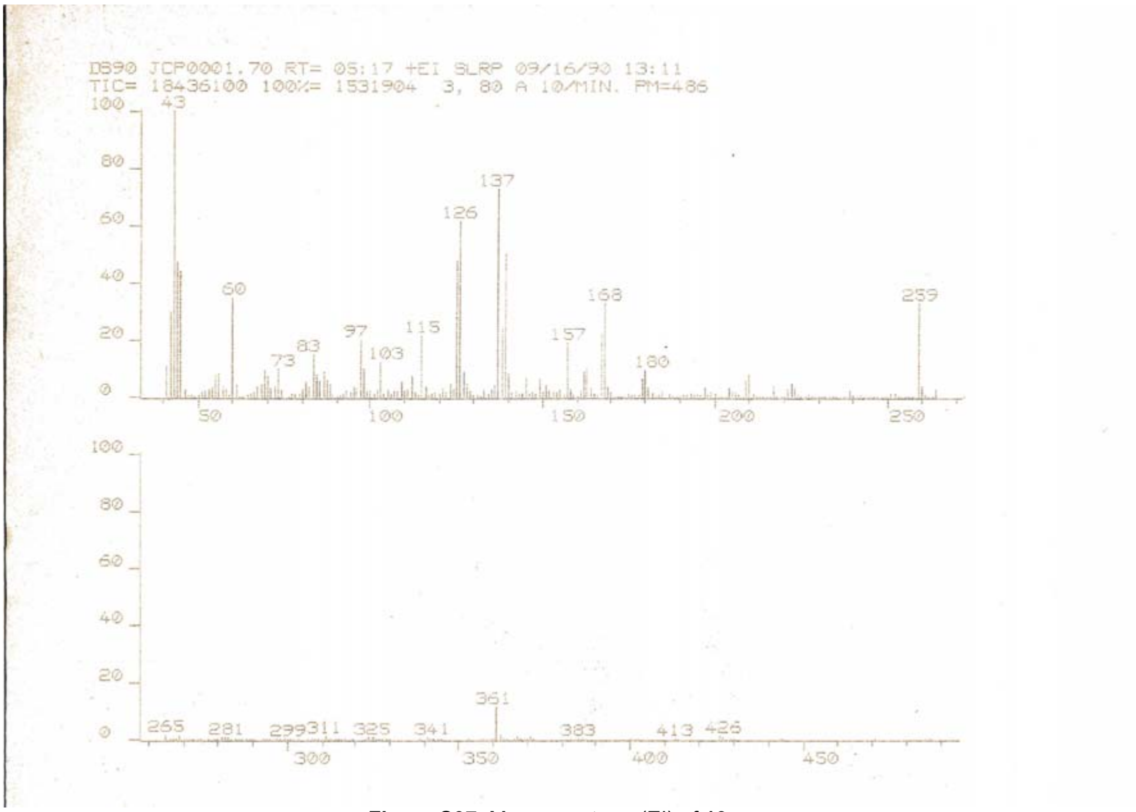

Figure S97. Mass spectrum (EI) of **13**.

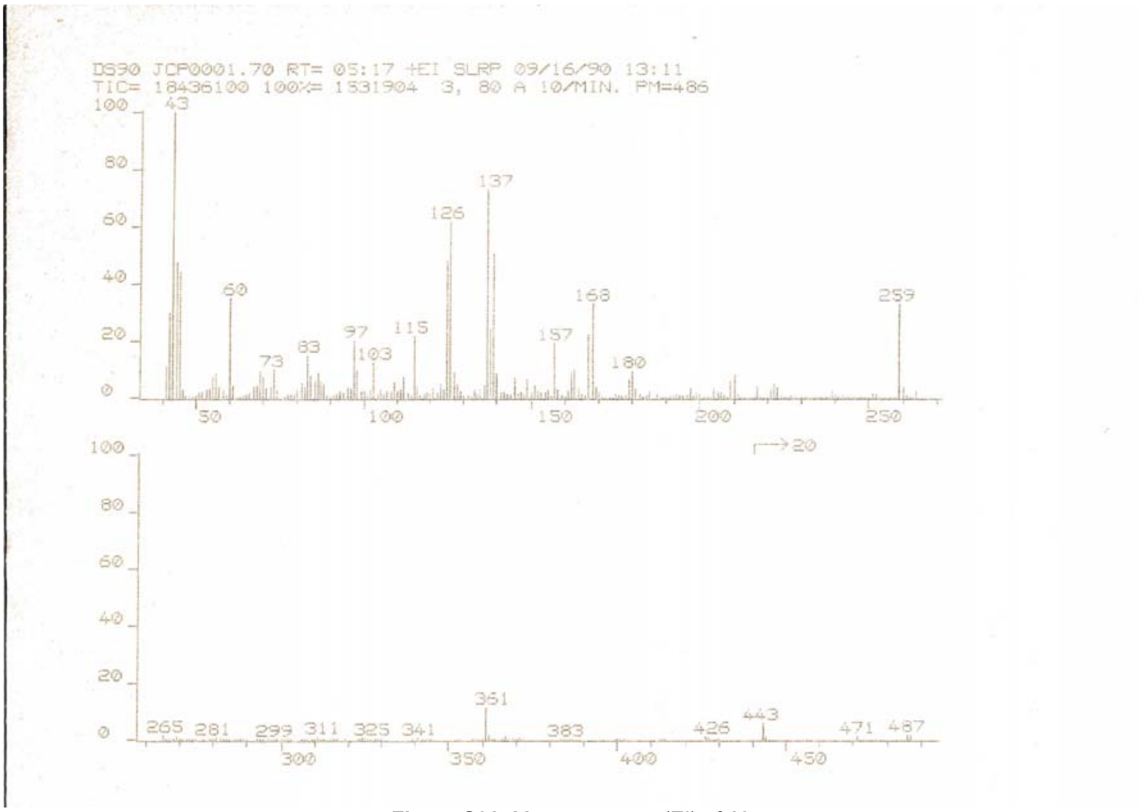

Figure S98. Mass spectrum (EI) of **13**.

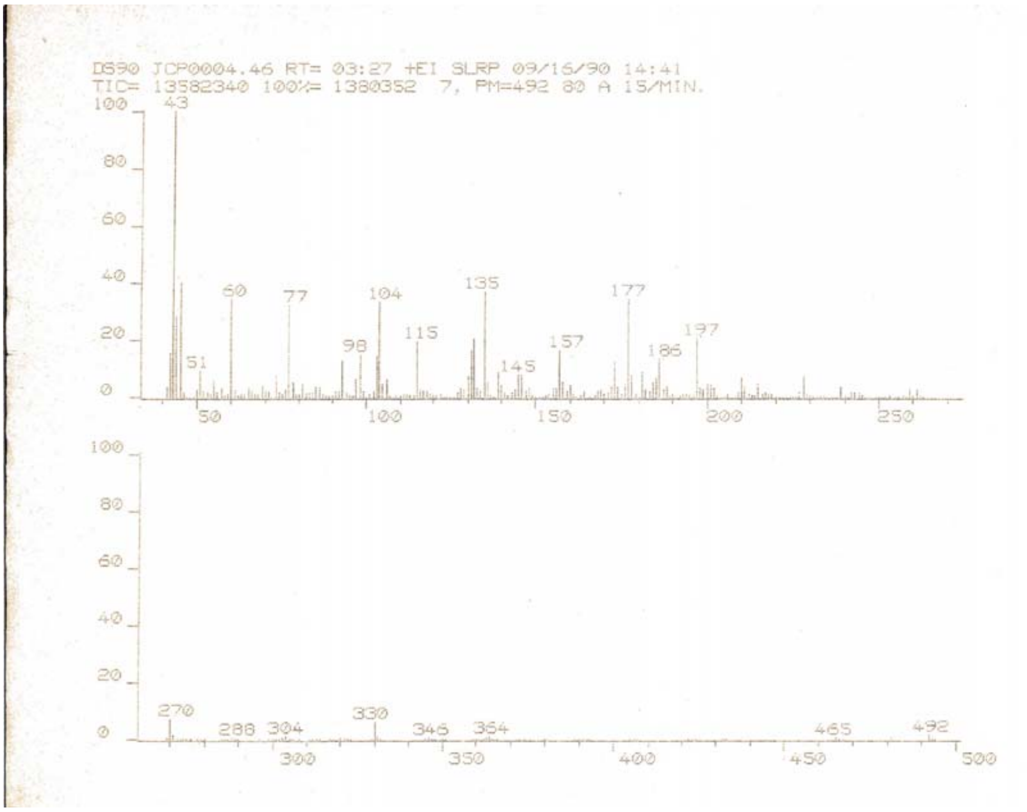

Figure S99. Mass spectrum (EI) of **17.**

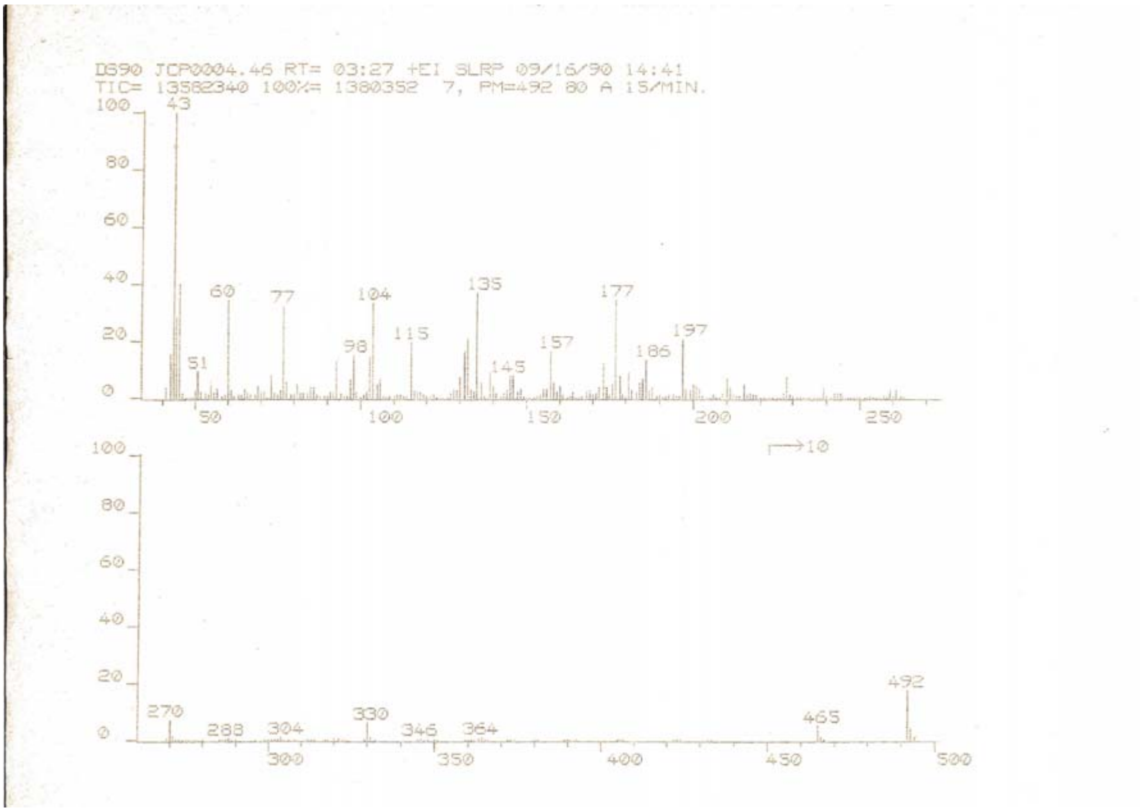

Figure S100. Mass spectrum (EI) of **17.**

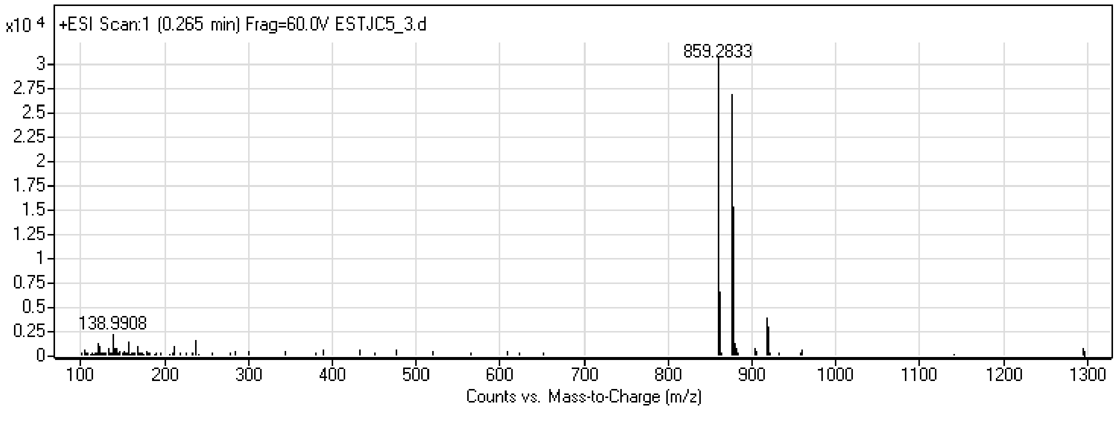

Figure S101. Mass spectrum of **18**.

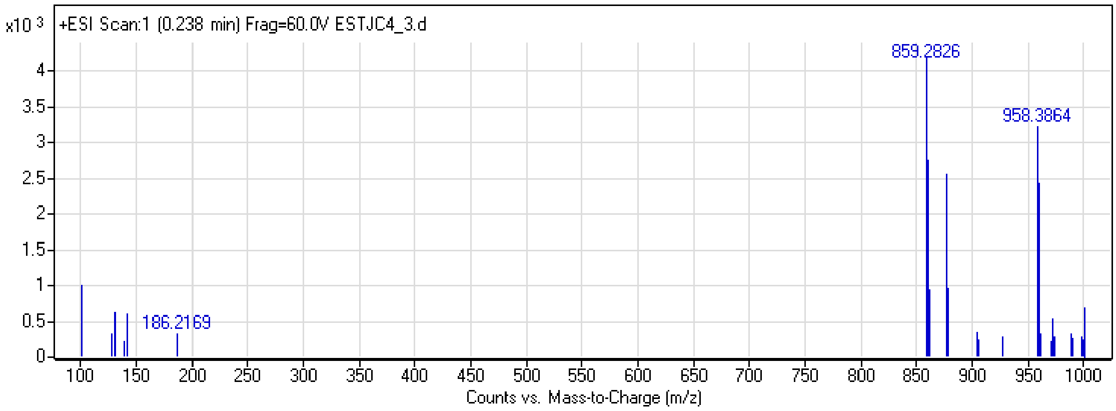

Figure S102. Mass spectrum of **20**.

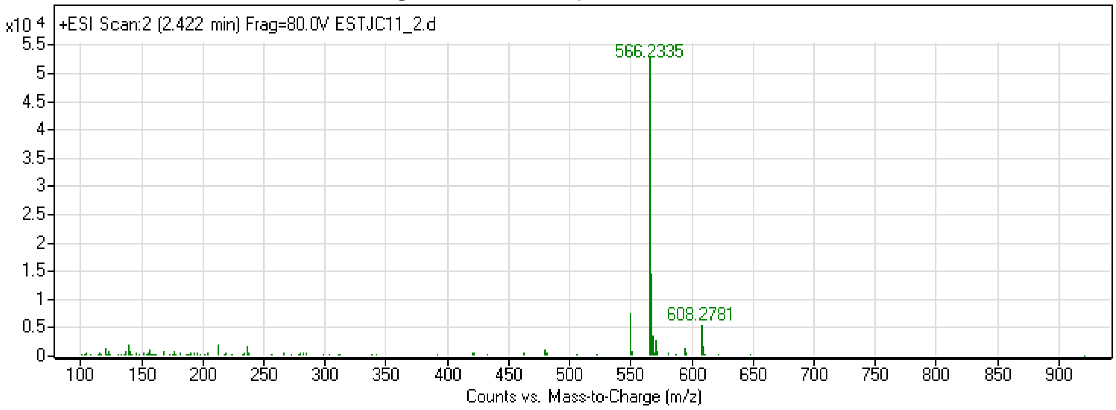

Figure S103. Mass spectrum of **37**

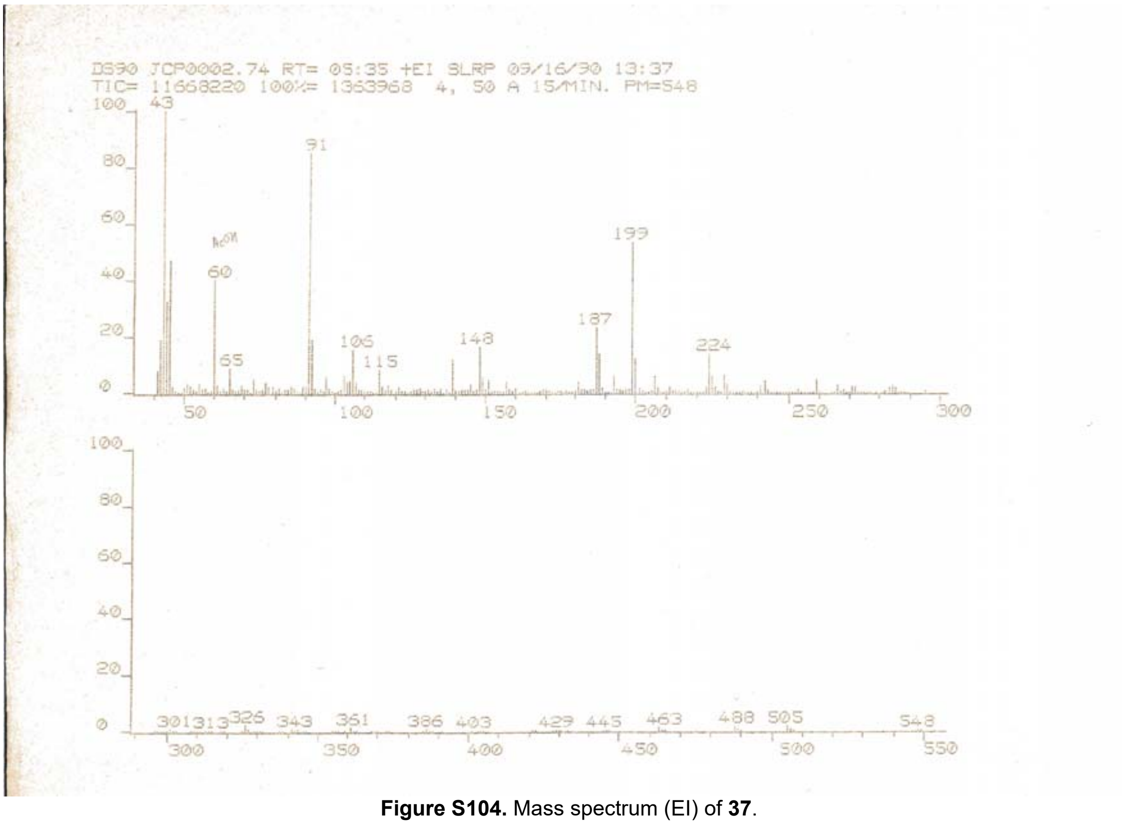

Figure S104. Mass spectrum (EI) of **37**.

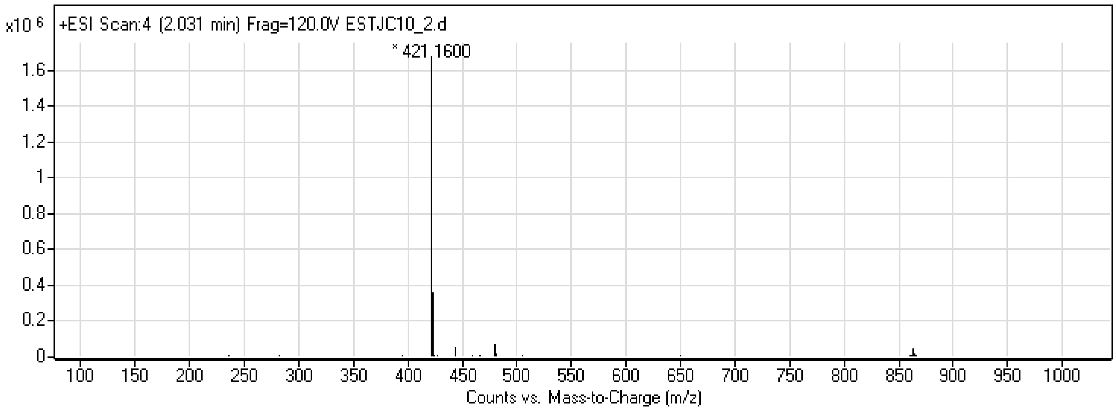

Figure S105. Mass spectrum of **42**.

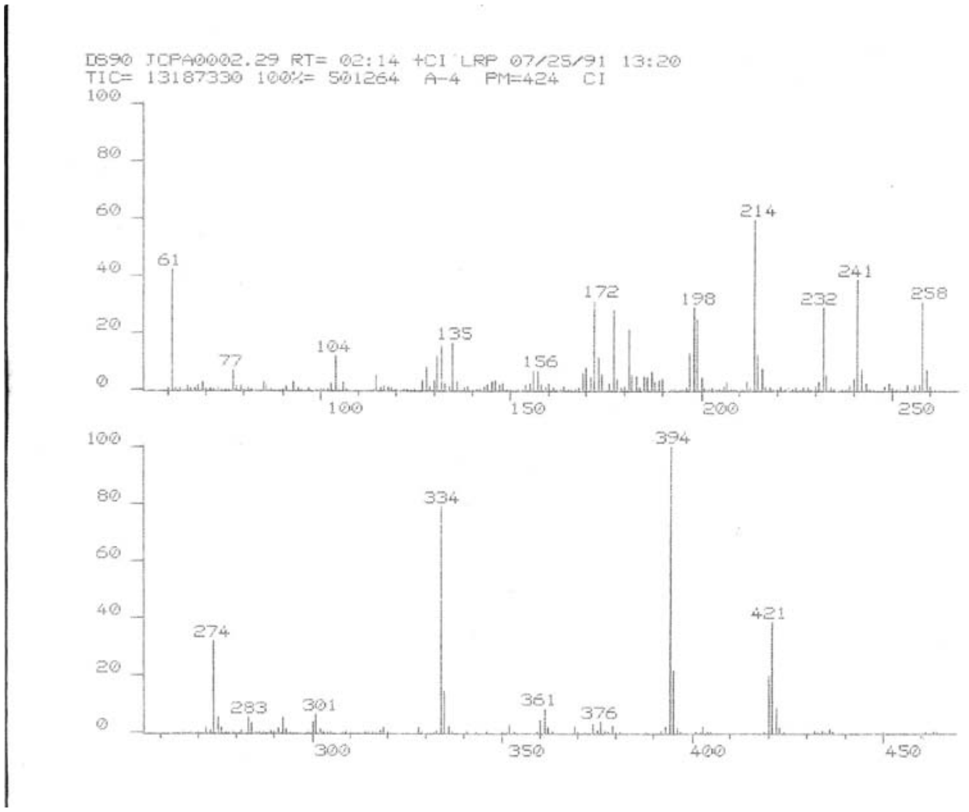

Figure S106. Mass spectrum (EI) of **42**.

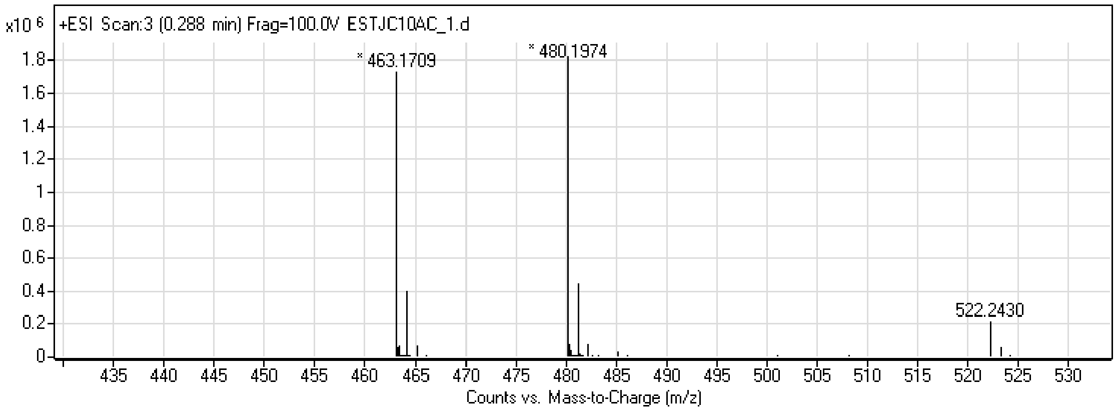

Figure S107. Mass spectrum of **43**.

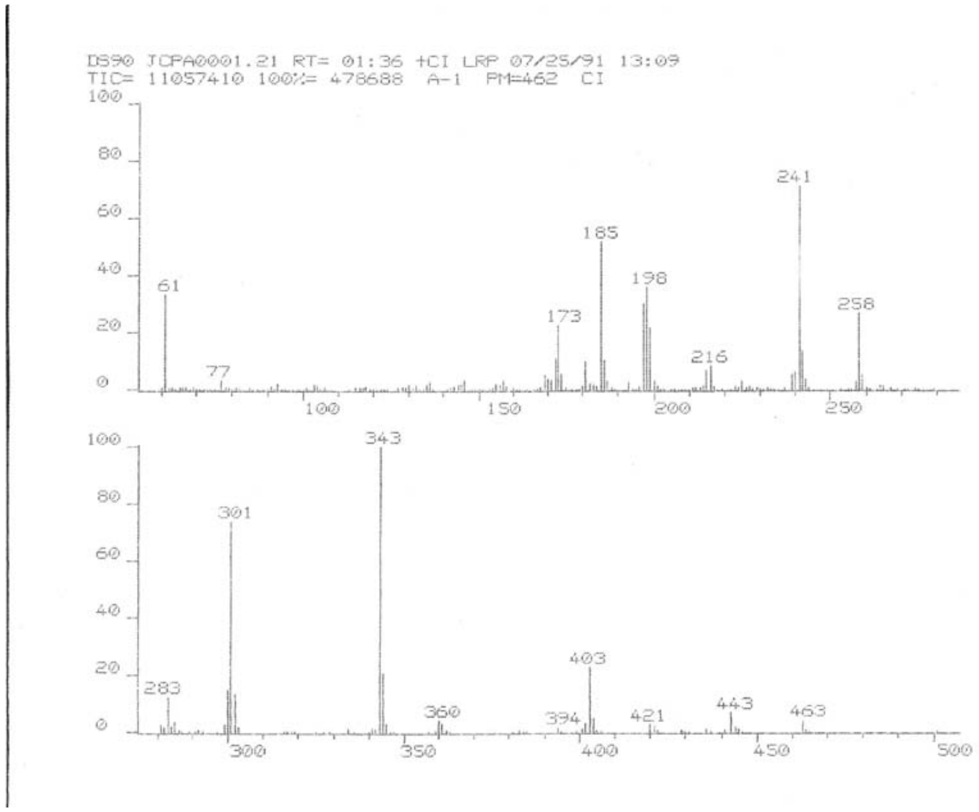

Figure S108. Mass spectrum (EI) of **43**.

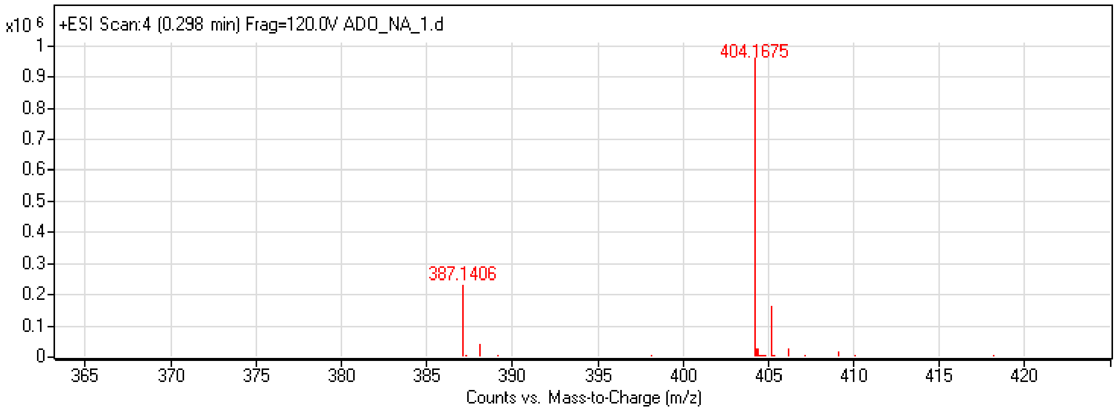

Figure S109. Mass spectrum of **47**.

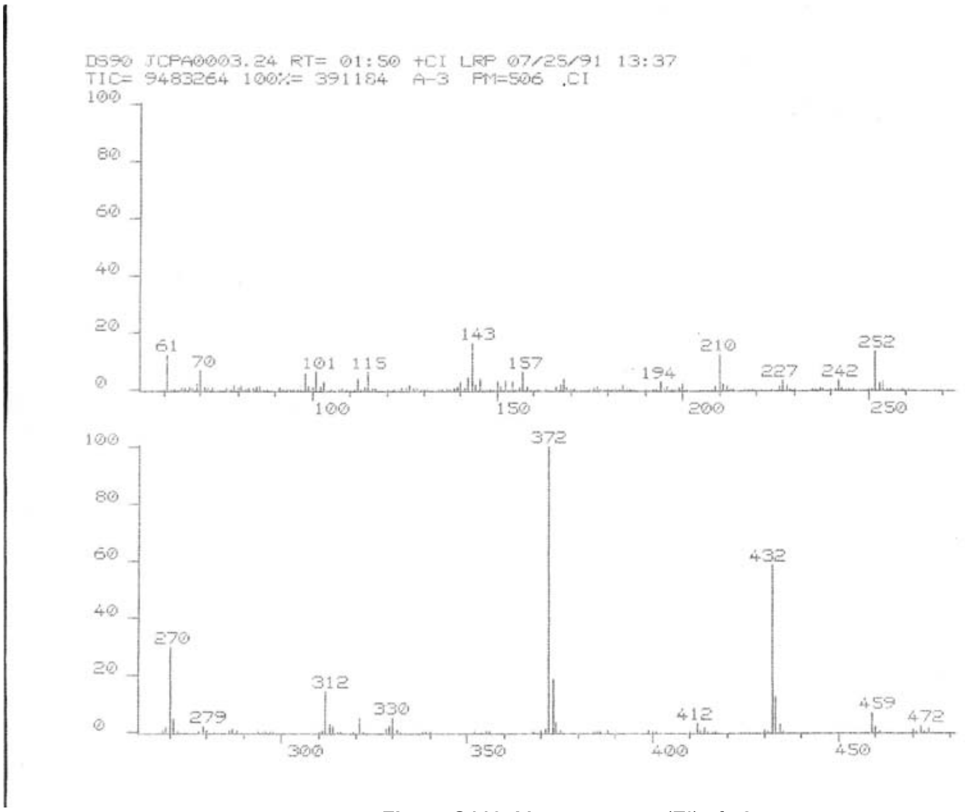

Figure S110. Mass spectrum (EI) of **58**.

Structure 13 A_1G^+Z (M06-2X, Gas Phase)

Energy (Hartrees): = -1755.1525901
No imaginary frequencies

Standard orientation:

Center Number	Atomic Number	Atomic Type	Coordinates (Angstroms) X	Y	Z
1	6	0	3.189721	1.650763	-0.244558
2	1	0	2.737969	2.440834	-0.846097
3	1	0	3.315478	1.983199	0.786914
4	6	0	2.315620	0.406248	-0.283086
5	1	0	2.474921	-0.104293	-1.235837
6	6	0	0.844192	0.753099	-0.165541
7	1	0	0.538950	1.271784	-1.074457
8	6	0	-0.038903	-0.478999	0.029917
9	1	0	0.253576	-1.018881	0.932404
10	6	0	-1.498428	-0.058720	0.145118
11	1	0	-1.647951	0.386680	1.130323
12	6	0	-2.505680	-1.204885	-0.081752
13	1	0	-2.419276	-1.521295	-1.123517
14	6	0	-2.203275	-2.345970	0.794032
15	7	0	-1.966782	-3.215881	1.504909
16	8	0	4.458853	1.380894	-0.840242
17	8	0	2.661597	-0.475690	0.784392
18	8	0	0.605219	1.602829	0.964288
19	8	0	0.154506	-1.317471	-1.109264
20	8	0	-1.778717	0.913859	-0.855446
21	6	0	5.430754	0.889262	-0.042260
22	6	0	3.521246	-1.482600	0.507229
23	6	0	0.800577	-2.506506	-0.916940
24	6	0	0.188016	2.871521	0.726777
25	6	0	-2.553450	1.971842	-0.524337
26	6	0	6.618557	0.468789	-0.855011
27	6	0	3.821939	-2.273435	1.738969
28	6	0	1.149975	-3.143333	-2.226721
29	6	0	-2.851346	2.794100	-1.737755
30	1	0	7.482534	0.360271	-0.204565
31	1	0	6.371612	-0.496217	-1.304558
32	1	0	6.811744	1.182114	-1.654408
33	1	0	1.176391	-4.223181	-2.101389
34	1	0	0.457286	-2.848897	-3.011829
35	1	0	2.155344	-2.798373	-2.485228
36	1	0	2.885610	-2.636332	2.163377
37	1	0	4.472681	-3.104845	1.482855
38	1	0	4.307273	-1.611126	2.457931
39	1	0	-3.506401	3.617763	-1.467042
40	1	0	-1.908741	3.175811	-2.133669
41	1	0	-3.311537	2.150758	-2.490513
42	6	0	-0.190991	3.557749	2.003605
43	1	0	0.459046	3.247246	2.819407
44	1	0	-0.157170	4.634170	1.855938
45	1	0	-1.217044	3.260841	2.236732
46	7	0	-3.865759	-0.734713	0.122235
47	6	0	-4.572537	-0.351239	-0.997613
48	6	0	-5.889829	0.354642	-0.756918
49	1	0	-6.392871	0.438805	-1.716570
50	1	0	-6.528823	-0.168482	-0.046551
51	1	0	-5.693645	1.356158	-0.362793
52	8	0	5.322370	0.780724	1.147712
53	8	0	3.970336	-1.673639	-0.592255
54	8	0	0.111728	3.346041	-0.372945
55	8	0	1.078132	-2.935616	0.163530
56	8	0	-2.929900	2.186172	0.597984
57	8	0	-4.141803	-0.521578	-2.119919
58	6	0	-4.334815	-0.518307	1.494605
59	1	0	-3.451723	-0.416702	2.129277
60	1	0	-4.846861	0.442723	1.548234
61	6	0	-5.208807	-1.656298	2.014285
62	1	0	-5.534897	-1.439635	3.033549
63	1	0	-6.096051	-1.790629	1.393111
64	1	0	-4.653411	-2.594641	2.024009

Structure 13 A_1G^+Z (M06-2X, $CHCl_3$)

Energy (Hartrees): = -1755.1837422
No imaginary frequencies

Standard orientation:

Center Number	Atomic Number	Atomic Type	Coordinates (Angstroms) X	Y	Z
1	6	0	3.225785	1.577620	-0.378356

2	1	0	2.808616	2.312859	-1.067614
3	1	0	3.312479	2.006757	0.620544
4	6	0	2.337038	0.343959	-0.348317
5	1	0	2.487021	-0.224859	-1.269230
6	6	0	0.871920	0.728941	-0.244497
7	1	0	0.579260	1.238517	-1.162877
8	6	0	-0.040045	-0.478401	-0.023210
9	1	0	0.271389	-1.030290	0.865172
10	6	0	-1.483585	-0.029028	0.147934
11	1	0	-1.581814	0.451513	1.122106
12	6	0	-2.510581	-1.174624	0.010477
13	1	0	-2.441437	-1.567506	-1.006223
14	6	0	-2.198108	-2.265076	0.945421
15	7	0	-1.932579	-3.102276	1.684441
16	8	0	4.519618	1.254683	-0.895029
17	8	0	2.656179	-0.479859	0.776206
18	8	0	0.669449	1.601727	0.874043
19	8	0	0.071966	-1.315085	-1.175629
20	8	0	-1.803386	0.902254	-0.879997
21	6	0	5.463746	0.851441	-0.022880
22	6	0	3.508058	-1.509450	0.582981
23	6	0	0.672322	-2.528917	-1.033701
24	6	0	0.270040	2.875696	0.633755
25	6	0	-2.526656	1.998049	-0.547852
26	6	0	6.701003	0.412361	-0.742453
27	6	0	3.709214	-2.277048	1.849363
28	6	0	0.771990	-3.226082	-2.353137
29	6	0	-2.901743	2.766333	-1.773257
30	1	0	7.512328	0.283566	-0.030076
31	1	0	6.483803	-0.540447	-1.231444
32	1	0	6.971346	1.134926	-1.512373
33	1	0	1.027114	-4.270422	-2.191054
34	1	0	-0.162729	-3.133172	-2.905908
35	1	0	1.562352	-2.744328	-2.934738
36	1	0	2.741519	-2.613364	2.223736
37	1	0	4.359282	-3.127705	1.659709
38	1	0	4.158205	-1.615804	2.593316
39	1	0	-3.461010	3.655614	-1.493152
40	1	0	-1.994443	3.042770	-2.312923
41	1	0	-3.498672	2.122740	-2.424231
42	6	0	0.002302	3.598525	1.915042
43	1	0	0.818533	3.436729	2.619310
44	1	0	-0.132158	4.658233	1.712765
45	1	0	-0.912661	3.190750	2.351449
46	7	0	-3.859609	-0.675107	0.200485
47	6	0	-4.592520	-0.401259	-0.931277
48	6	0	-5.886393	0.351363	-0.733301
49	1	0	-6.406963	0.374231	-1.687763
50	1	0	-6.527086	-0.099960	0.024101
51	1	0	-5.662874	1.376096	-0.420950
52	8	0	5.303297	0.837871	1.168895
53	8	0	4.028337	-1.739674	-0.477193
54	8	0	0.142050	3.326322	-0.472643
55	8	0	1.074325	-2.942532	0.017359
56	8	0	-2.808357	2.277996	0.587571
57	8	0	-4.193738	-0.701923	-2.042674
58	6	0	-4.312795	-0.361496	1.561808
59	1	0	-3.422060	-0.211449	2.175344
60	1	0	-4.833677	0.595811	1.550379
61	6	0	-5.179647	-1.458720	2.169257
62	1	0	-5.492744	-1.166394	3.174068
63	1	0	-6.075117	-1.635267	1.570056
64	1	0	-4.628476	-2.397816	2.243950

Structure 13 A_1G^+E (M06-2X, Gas Phase)

Energy (Hartrees): = -1755.1511944
No imaginary frequencies

Standard orientation:

Center Number	Atomic Number	Atomic Type	Coordinates (Angstroms) X	Y	Z
1	6	0	-3.220967	1.622364	0.299021
2	1	0	-2.774262	2.418517	0.896416
3	1	0	-3.402557	1.966832	-0.720017
4	6	0	-2.304481	0.409214	0.282786
5	1	0	-2.426701	-0.132759	1.222931
6	6	0	-0.843719	0.796812	0.143564
7	1	0	-0.523134	1.269867	1.072358
8	6	0	0.042156	-0.416340	-0.135934
9	1	0	-0.267944	-0.914634	-1.056149
10	6	0	1.498457	0.000638	-0.261199
11	1	0	1.623672	0.540566	-1.200661
12	6	0	2.482150	-1.195332	-0.205584

13	1	0	2.225744	-1.796026	0.666556
14	6	0	2.295955	-2.046747	-1.392405
15	7	0	2.151977	-2.678756	-2.339467
16	8	0	-4.453323	1.294470	0.942880
17	8	0	-2.648946	-0.455139	-0.801506
18	8	0	-0.650148	1.710239	-0.939938
19	8	0	-0.138930	-1.304599	0.971011
20	8	0	1.840446	0.856733	0.821527
21	6	0	-5.433037	0.762007	0.181058
22	6	0	-3.446891	-1.512337	-0.532240
23	6	0	-0.705251	-2.522411	0.715379
24	6	0	-0.245179	2.972214	-0.635005
25	6	0	2.538132	1.990874	0.522287
26	6	0	-6.564650	0.279534	1.038222
27	6	0	-3.776559	-2.259175	-1.783486
28	6	0	-1.096010	-3.208304	1.987973
29	6	0	3.016560	2.664687	1.767938
30	1	0	-7.447236	0.126941	0.422652
31	1	0	-6.250261	-0.672510	1.473239
32	1	0	-6.762594	0.979867	1.847835
33	1	0	-1.103514	-4.283637	1.827620
34	1	0	-0.437604	-2.931159	2.808707
35	1	0	-2.113527	-2.880383	2.220198
36	1	0	-2.852306	-2.519951	-2.299172
37	1	0	-4.340968	-3.152687	-1.531414
38	1	0	-4.367246	-1.599590	-2.422110
39	1	0	3.256972	3.700281	1.542842
40	1	0	2.260103	2.599456	2.548110
41	1	0	3.921144	2.144932	2.099420
42	6	0	0.059008	3.750244	-1.877308
43	1	0	-0.623731	3.482235	-2.681385
44	1	0	0.014428	4.813280	-1.654570
45	1	0	1.077556	3.489948	-2.176478
46	7	0	3.852568	-0.748924	-0.097714
47	6	0	4.467167	-0.576467	1.124756
48	6	0	3.799149	-1.167266	2.352151
49	1	0	4.451827	-0.959871	3.196135
50	1	0	2.818999	-0.719716	2.527472
51	1	0	3.678694	-2.248654	2.252020
52	8	0	-5.367452	0.664868	-1.012977
53	8	0	-3.826783	-1.777069	0.578743
54	8	0	-0.127689	3.368560	0.490882
55	8	0	-0.885837	-2.938809	-0.390726
56	8	0	2.737963	2.348820	-0.603592
57	8	0	5.519917	0.021855	1.206771
58	6	0	4.516089	-0.186898	-1.285230
59	1	0	3.750024	-0.007321	-2.042379
60	1	0	4.938995	0.778474	-1.007725
61	6	0	5.596452	-1.120367	-1.813561
62	1	0	6.079199	-0.678390	-2.687138
63	1	0	6.353452	-1.278926	-1.044516
64	1	0	5.170021	-2.082334	-2.104229

Structure 13 A_1G^+E (M06-2X, $CHCl_3$)

Energy (Hartrees): = -1755.1825417
No imaginary frequencies

Standard orientation:

Center Number	Atomic Number	Atomic Type	Coordinates (Angstroms) X	Y	Z
1	6	0	-3.238597	1.527747	0.435855
2	1	0	-2.822450	2.253888	1.135183
3	1	0	-3.360635	1.982330	-0.548016
4	6	0	-2.323500	0.316590	0.348377
5	1	0	-2.448778	-0.288519	1.249620
6	6	0	-0.866648	0.732179	0.240335
7	1	0	-0.570323	1.209710	1.174902
8	6	0	0.056315	-0.455633	-0.034257
9	1	0	-0.260205	-0.984962	-0.934445
10	6	0	1.494982	0.007611	-0.211749
11	1	0	1.584529	0.495065	-1.182697
12	6	0	2.525405	-1.140548	-0.095679
13	1	0	2.373371	-1.627563	0.867567
14	6	0	2.266564	-2.158881	-1.128423
15	7	0	2.054126	-2.935444	-1.946288
16	8	0	-4.511191	1.158463	0.974285
17	8	0	-2.643099	-0.472448	-0.801180
18	8	0	-0.691727	1.651077	-0.843421
19	8	0	-0.041918	-1.328706	1.094468
20	8	0	1.829219	0.930537	0.818531
21	6	0	-5.468588	0.762077	0.113055
22	6	0	-3.469550	-1.527238	-0.632800
23	6	0	-0.617732	-2.550413	0.910305

24	6	0	-0.335797	2.928525	-0.553284
25	6	0	2.485900	2.066962	0.455611
26	6	0	-6.677625	0.272962	0.847977
27	6	0	-3.682086	-2.246585	-1.925172
28	6	0	-0.741351	-3.278889	2.210523
29	6	0	2.946845	2.823100	1.658341
30	1	0	-7.510164	0.171425	0.155945
31	1	0	-6.434273	-0.702700	1.276132
32	1	0	-6.930124	0.949246	1.664364
33	1	0	-0.987805	-4.320247	2.018731
34	1	0	0.181195	-3.196154	2.785287
35	1	0	-1.545510	-2.814293	2.787055
36	1	0	-2.718575	-2.592091	-2.303214
37	1	0	-4.350181	-3.089491	-1.766873
38	1	0	-4.109638	-1.552623	-2.651290
39	1	0	3.120459	3.862230	1.388449
40	1	0	2.219824	2.744657	2.465159
41	1	0	3.890575	2.378261	1.990424
42	6	0	-0.146258	3.721275	-1.806225
43	1	0	-1.059403	3.686768	-2.403011
44	1	0	0.105231	4.747893	-1.552022
45	1	0	0.658992	3.270744	-2.389273
46	7	0	3.881709	-0.646080	-0.183816
47	6	0	4.657576	-0.411547	0.928067
48	6	0	4.136628	-0.854748	2.277628
49	1	0	4.884627	-0.579506	3.017412
50	1	0	3.188337	-0.372927	2.522505
51	1	0	3.992847	-1.937707	2.304474
52	8	0	-5.337127	0.787492	-1.081979
53	8	0	-3.957063	-1.814137	0.429093
54	8	0	-0.196771	3.330858	0.570141
55	8	0	-0.981612	-2.946210	-0.160589
56	8	0	2.667674	2.371801	-0.690816
57	8	0	5.740327	0.134190	0.814424
58	6	0	4.400227	-0.220383	-1.493018
59	1	0	3.551121	-0.121181	-2.171747
60	1	0	4.842075	0.768786	-1.372904
61	6	0	5.418284	-1.206124	-2.049503
62	1	0	5.786315	-0.854914	-3.016044
63	1	0	6.266879	-1.294139	-1.369583
64	1	0	4.971195	-2.192900	-2.190788

Structure 13 B_1G^+Z (M06-2X, Gas Phase)

Energy (Hartrees): = -1755.1479943
No imaginary frequencies

Standard orientation:

Center Number	Atomic Number	Atomic Type	Coordinates (Angstroms) X	Y	Z
1	6	0	3.190226	1.579712	-0.382641
2	1	0	2.762887	2.321612	-1.058401
3	1	0	3.310422	2.001103	0.616418
4	6	0	2.291057	0.353221	-0.321482
5	1	0	2.457214	-0.246515	-1.219817
6	6	0	0.825815	0.738337	-0.262597
7	1	0	0.556688	1.202463	-1.211943
8	6	0	-0.096598	-0.452368	-0.000533
9	1	0	0.144966	-0.915270	0.958570
10	6	0	-1.546234	0.004093	0.019506
11	1	0	-1.728448	0.570478	0.931086
12	6	0	-2.565761	-1.140486	-0.084380
13	1	0	-2.456543	-1.562832	-1.086781
14	6	0	-2.291357	-2.240596	0.856635
15	7	0	-2.059747	-3.145205	1.523825
16	8	0	4.460909	1.231175	-0.932738
17	8	0	2.599779	-0.429712	0.830405
18	8	0	0.583327	1.665436	0.802338
19	8	0	0.113189	-1.396918	-1.054457
20	8	0	-1.796411	0.832886	-1.124503
21	6	0	5.412053	0.798501	-0.076918
22	6	0	3.443244	-1.473865	0.668169
23	6	0	0.749721	-2.564478	-0.737284
24	6	0	0.223826	2.929660	0.477431
25	6	0	-2.448165	2.005452	-0.927579
26	6	0	6.593297	0.262105	-0.828418
27	6	0	3.691288	-2.158811	1.972749
28	6	0	1.113321	-3.332424	-1.971918
29	6	0	-2.552638	2.776958	-2.208348
30	1	0	7.447318	0.192088	-0.159773
31	1	0	6.316795	-0.735016	-1.180258
32	1	0	6.818081	0.883467	-1.693798
33	1	0	1.066363	-4.397196	-1.754486
34	1	0	0.476836	-3.068944	-2.813383

35	1	0	2.149822	-3.073908	-2.207152
36	1	0	2.739287	-2.525465	2.358493
37	1	0	4.383450	-2.982329	1.820693
38	1	0	4.103390	-1.431123	2.673201
39	1	0	-3.324714	3.535737	-2.108597
40	1	0	-1.586348	3.261432	-2.367803
41	1	0	-2.754016	2.113375	-3.047963
42	6	0	-0.198416	3.694891	1.692746
43	1	0	0.415846	3.427086	2.550561
44	1	0	-0.149245	4.760346	1.483476
45	1	0	-1.234092	3.414827	1.904211
46	7	0	-3.916436	-0.615874	0.028441
47	6	0	-4.295810	-0.111814	1.255424
48	6	0	-5.670721	0.506372	1.354663
49	1	0	-5.843210	0.737732	2.402177
50	1	0	-5.695975	1.431986	0.774678
51	1	0	-6.453658	-0.160718	0.990859
52	8	0	5.289774	0.815062	1.116194
53	8	0	3.914277	-1.773250	-0.397824
54	8	0	0.222385	3.342858	-0.651152
55	8	0	1.013194	-2.887478	0.381951
56	8	0	-2.846007	2.368938	0.144194
57	8	0	-3.535014	-0.148219	2.197686
58	6	0	-4.776391	-0.704267	-1.147430
59	1	0	-5.546353	0.062689	-1.072211
60	1	0	-4.171002	-0.449171	-2.022297
61	6	0	-5.401333	-2.086205	-1.310390
62	1	0	-6.021327	-2.126922	-2.208043
63	1	0	-4.628203	-2.853842	-1.393876
64	1	0	-6.020705	-2.329772	-0.444890

Structure 13 B_1G^+Z (M06-2X, $CHCl_3$)

Energy (Hartrees): =-1755.1821906
No imaginary frequencies

Standard orientation:

Center Number	Atomic Number	Atomic Type	Coordinates (Angstroms) X	Y	Z
1	6	0	3.228364	1.478007	-0.477055
2	1	0	2.833733	2.173455	-1.218423
3	1	0	3.318394	1.974540	0.490277
4	6	0	2.309555	0.270663	-0.362833
5	1	0	2.469803	-0.382487	-1.224537
6	6	0	0.852670	0.697762	-0.334261
7	1	0	0.605520	1.150491	-1.295181
8	6	0	-0.104027	-0.462226	-0.056977
9	1	0	0.156016	-0.949699	0.884443
10	6	0	-1.538896	0.035423	0.019472
11	1	0	-1.674454	0.595631	0.942633
12	6	0	-2.587382	-1.087487	-0.062354
13	1	0	-2.512020	-1.524606	-1.061507
14	6	0	-2.302734	-2.184140	0.881338
15	7	0	-2.048700	-3.078845	1.554002
16	8	0	4.518707	1.091895	-0.958106
17	8	0	2.577504	-0.453314	0.839897
18	8	0	0.641755	1.649978	0.715261
19	8	0	0.019579	-1.394954	-1.135560
20	8	0	-1.805354	0.878754	-1.106030
21	6	0	5.450164	0.740589	-0.050197
22	6	0	3.419907	-1.505387	0.767977
23	6	0	0.623393	-2.590006	-0.887862
24	6	0	0.337826	2.926363	0.378964
25	6	0	-2.417918	2.067426	-0.876948
26	6	0	6.685438	0.230801	-0.724811
27	6	0	3.564498	-2.159506	2.103596
28	6	0	0.754097	-3.394218	-2.142625
29	6	0	-2.606084	2.822809	-2.154287
30	1	0	7.496884	0.167341	-0.003709
31	1	0	6.462611	-0.765179	-1.115825
32	1	0	6.958296	0.872412	-1.562455
33	1	0	0.823166	-4.449258	-1.886987
34	1	0	-0.076400	-3.207495	-2.821077
35	1	0	1.681558	-3.090926	-2.636783
36	1	0	2.581185	-2.472193	2.457770
37	1	0	4.228529	-3.016281	2.020534
38	1	0	3.970343	-1.432145	2.809680
39	1	0	-3.208092	3.708248	-1.965108
40	1	0	-1.621324	3.117717	-2.522851
41	1	0	-3.076960	2.188133	-2.905777
42	6	0	0.052081	3.743660	1.597222
43	1	0	0.849823	3.615673	2.329776
44	1	0	-0.052972	4.788905	1.317592
45	1	0	-0.878447	3.382431	2.041353

46	7	0	-3.926887	-0.546026	0.081682
47	6	0	-4.294264	-0.079312	1.319099
48	6	0	-5.675713	0.508309	1.455584
49	1	0	-5.838547	0.728628	2.507820
50	1	0	-5.745433	1.434637	0.880409
51	1	0	-6.445343	-0.179248	1.100655
52	8	0	5.280022	0.816110	1.137690
53	8	0	3.974245	-1.833370	-0.248464
54	8	0	0.298788	3.312349	-0.759381
55	8	0	1.016743	-2.915929	0.195825
56	8	0	-2.731593	2.442362	0.219792
57	8	0	-3.515871	-0.133776	2.255029
58	6	0	-4.814545	-0.627613	-1.080452
59	1	0	-5.577657	0.143786	-0.987229
60	1	0	-4.222404	-0.377603	-1.964306
61	6	0	-5.446217	-2.005248	-1.229744
62	1	0	-6.090380	-2.035343	-2.111159
63	1	0	-4.678191	-2.774932	-1.344139
64	1	0	-6.045617	-2.251385	-0.350344

Structure 13 B_1G^+E (M06-2X, Gas Phase)

Energy (Hartrees): =-1755.1465438
No imaginary frequencies

Standard orientation:

Center Number	Atomic Number	Atomic Type	Coordinates (Angstroms) X	Y	Z
1	6	0	3.249372	1.498185	-0.405234
2	1	0	2.853388	2.256609	-1.081493
3	1	0	3.408478	1.919965	0.588494
4	6	0	2.286998	0.322471	-0.322596
5	1	0	2.408239	-0.289391	-1.219591
6	6	0	0.840205	0.773027	-0.244739
7	1	0	0.576051	1.233360	-1.197364
8	6	0	-0.115288	-0.385663	0.042551
9	1	0	0.124412	-0.846904	1.003009
10	6	0	-1.558997	0.104031	0.067353
11	1	0	-1.704296	0.690743	0.972952
12	6	0	-2.587090	-1.038516	-0.021023
13	1	0	-2.374742	-1.547487	-0.965119
14	6	0	-2.373584	-2.048898	1.031940
15	7	0	-2.213025	-2.838218	1.850211
16	8	0	4.490151	1.075750	-0.971905
17	8	0	2.573986	-0.471049	0.829161
18	8	0	0.650813	1.725153	0.808094
19	8	0	0.070896	-1.343993	-1.001415
20	8	0	-1.804207	0.926116	-1.071786
21	6	0	5.426671	0.587268	-0.130459
22	6	0	3.351802	-1.563814	0.656705
23	6	0	0.641822	-2.542425	-0.664655
24	6	0	0.411074	3.013454	0.457245
25	6	0	-2.329262	2.158943	-0.854087
26	6	0	6.568548	-0.006735	-0.899047
27	6	0	3.620770	-2.234130	1.964246
28	6	0	1.028744	-3.314077	-1.888273
29	6	0	-2.489134	2.912307	-2.136856
30	1	0	7.422090	-0.135489	-0.238723
31	1	0	6.232885	-0.981101	-1.262979
32	1	0	6.822312	0.612807	-1.757726
33	1	0	0.982730	-4.378202	-1.668678
34	1	0	0.405118	-3.054306	-2.740792
35	1	0	2.067459	-3.048671	-2.106942
36	1	0	2.675042	-2.417745	2.473619
37	1	0	4.150007	-3.166494	1.788487
38	1	0	4.226998	-1.557303	2.569631
39	1	0	-3.236250	3.690491	-2.002465
40	1	0	-1.523050	3.375139	-2.354843
41	1	0	-2.754834	2.243937	-2.953475
42	6	0	0.102322	3.850014	1.659478
43	1	0	0.701287	3.537684	2.513044
44	1	0	0.267920	4.897258	1.419970
45	1	0	-0.954061	3.697550	1.894136
46	7	0	-3.954893	-0.563019	-0.110569
47	6	0	-4.705244	-0.067655	0.948437
48	6	0	-3.996598	0.322941	2.226382
49	1	0	-4.758126	0.394346	2.999035
50	1	0	-3.225087	-0.376419	2.545028
51	1	0	-3.550637	1.309285	2.079081
52	8	0	5.318652	0.602564	1.064341
53	8	0	3.763723	-1.907672	-0.420501
54	8	0	0.425013	3.395157	-0.681324
55	8	0	0.833186	-2.880267	0.464997
56	8	0	-2.575845	2.580462	0.242044

57	8	0	-5.897289	0.099057	0.818313
58	6	0	-4.683435	-0.923445	-1.332046
59	1	0	-5.497039	-0.208906	-1.439609
60	1	0	-3.998068	-0.790043	-2.173017
61	6	0	-5.231124	-2.344557	-1.275846
62	1	0	-5.754521	-2.592530	-2.201060
63	1	0	-4.429630	-3.074548	-1.131897
64	1	0	-5.932711	-2.431534	-0.445226

Structure 13 A_1G^-Z (M06-2X, Gas Phase)

Energy (Hartrees): = - 1755.1499916
No imaginary frequencies

Standard orientation:

Center Number	Atomic Number	Atomic Type	Coordinates (Angstroms) X	Y	Z
1	6	0	3.242478	1.263626	0.109783
2	1	0	3.091142	2.204596	-0.419945
3	1	0	3.296960	1.436513	1.185536
4	6	0	2.108274	0.302630	-0.207375
5	1	0	2.281775	-0.130991	-1.193903
6	6	0	0.753998	0.990577	-0.222822
7	1	0	0.710035	1.620243	-1.112942
8	6	0	-0.390602	-0.022148	-0.263600
9	1	0	-0.362701	-0.665864	0.616509
10	6	0	-1.731551	0.688289	-0.325017
11	1	0	-1.859836	1.268161	0.588849
12	6	0	-2.973832	-0.222487	-0.421155
13	1	0	-3.827288	0.462858	-0.475779
14	6	0	-2.978777	-1.022702	-1.654131
15	7	0	-3.001021	-1.650580	-2.615031
16	8	0	4.478416	0.733195	-0.373676
17	8	0	2.068945	-0.750358	0.759480
18	8	0	0.556811	1.801694	0.937566
19	8	0	-0.164610	-0.796385	-1.442253
20	8	0	-1.737707	1.565948	-1.449196
21	6	0	5.166631	-0.092875	0.441192
22	6	0	2.674211	-1.915028	0.438931
23	6	0	0.011890	-2.133511	-1.319432
24	6	0	0.570286	3.150194	0.766301
25	6	0	-1.962884	2.886778	-1.205094
26	6	0	6.305792	-0.732701	-0.294637
27	6	0	2.620915	-2.871240	1.587024
28	6	0	0.346545	-2.742849	-2.643451
29	6	0	-1.738419	3.704220	-2.439180
30	1	0	7.023613	-1.128615	0.419242
31	1	0	5.880963	-1.551277	-0.881566
32	1	0	6.773375	-0.025475	-0.977381
33	1	0	0.330260	-3.825467	-2.553925
34	1	0	-0.368260	-2.399022	-3.391329
35	1	0	1.347553	-2.410952	-2.927796
36	1	0	1.591160	-2.962414	1.931503
37	1	0	3.007576	-3.836039	1.270508
38	1	0	3.232054	-2.458324	2.392220
39	1	0	-2.325519	4.617352	-2.377895
40	1	0	-0.677021	3.968363	-2.448522
41	1	0	-1.973538	3.136735	-3.337005
42	6	0	0.126999	3.851211	2.011172
43	1	0	0.517560	3.351605	2.896069
44	1	0	0.440965	4.890950	1.970257
45	1	0	-0.964892	3.801780	2.031409
46	7	0	-3.120791	-1.056193	0.773563
47	6	0	-3.592163	-0.381856	1.882216
48	6	0	-3.782873	-1.181099	3.153742
49	1	0	-4.177945	-0.498656	3.901432
50	1	0	-4.471645	-2.015263	3.018361
51	1	0	-2.827028	-1.581636	3.497990
52	8	0	4.865973	-0.304057	1.583890
53	8	0	3.190751	-2.113924	-0.628953
54	8	0	0.874295	3.672915	-0.270378
55	8	0	-0.085953	-2.716587	-0.272985
56	8	0	-2.252112	3.312741	-0.124580
57	8	0	-3.802487	0.813235	1.844969
58	6	0	-3.129033	-2.522259	0.687386
59	1	0	-2.382973	-2.824343	-0.043855
60	1	0	-2.776585	-2.912421	1.641731
61	6	0	-4.496928	-3.095532	0.327878
62	1	0	-4.450441	-4.186113	0.308658
63	1	0	-5.257884	-2.797300	1.052171
64	1	0	-4.813064	-2.756336	-0.659792

Structure 13 A_1G^-Z (M06-2X, $CHCl_3$)

Energy (Hartrees): = -1755.1802673
No imaginary frequencies

Standard orientation:

Center Number	Atomic Number	Atomic Type	Coordinates (Angstroms) X	Y	Z
1	6	0	3.235010	1.248901	-0.018582
2	1	0	3.070072	2.157783	-0.597898
3	1	0	3.300603	1.488211	1.043498
4	6	0	2.101994	0.267224	-0.270721
5	1	0	2.247155	-0.196443	-1.248537
6	6	0	0.750094	0.961316	-0.259418
7	1	0	0.697423	1.612550	-1.133165
8	6	0	-0.414171	-0.032360	-0.298228
9	1	0	-0.358980	-0.717330	0.548881
10	6	0	-1.740931	0.706710	-0.265073
11	1	0	-1.807688	1.257771	0.671939
12	6	0	-3.008982	-0.173927	-0.322458
13	1	0	-3.851313	0.524524	-0.264371
14	6	0	-3.130320	-0.868564	-1.611911
15	7	0	-3.245344	-1.400328	-2.622584
16	8	0	4.477512	0.715412	-0.487477
17	8	0	2.085950	-0.748892	0.736971
18	8	0	0.591088	1.741499	0.929293
19	8	0	-0.279317	-0.752970	-1.524975
20	8	0	-1.785252	1.614678	-1.364177
21	6	0	5.199387	-0.043108	0.358486
22	6	0	2.707450	-1.915039	0.464384
23	6	0	-0.007452	-2.079401	-1.479774
24	6	0	0.557714	3.093463	0.803488
25	6	0	-2.071609	2.917084	-1.095662
26	6	0	6.396472	-0.618960	-0.332561
27	6	0	2.615630	-2.852139	1.625029
28	6	0	0.138180	-2.633723	-2.859880
29	6	0	-1.977177	3.747006	-2.334819
30	1	0	7.074661	-1.045117	0.402801
31	1	0	6.047984	-1.402135	-1.010312
32	1	0	6.898296	0.145215	-0.925833
33	1	0	0.365167	-3.695177	-2.803288
34	1	0	-0.790964	-2.467923	-3.409509
35	1	0	0.937934	-2.103313	-3.379939
36	1	0	1.589175	-2.894104	1.988376
37	1	0	2.957836	-3.839130	1.323632
38	1	0	3.251660	-2.464881	2.424711
39	1	0	-2.453527	4.709530	-2.165201
40	1	0	-0.917219	3.901778	-2.552965
41	1	0	-2.429768	3.228452	-3.179285
42	6	0	0.200630	3.738765	2.102817
43	1	0	0.787666	3.307616	2.913886
44	1	0	0.361721	4.811595	2.033240
45	1	0	-0.855469	3.537567	2.299512
46	7	0	-3.082774	-1.095323	0.809907
47	6	0	-3.443121	-0.517064	2.003988
48	6	0	-3.533456	-1.416163	3.213225
49	1	0	-3.881879	-0.810059	4.046011
50	1	0	-4.217686	-2.250497	3.056716
51	1	0	-2.548491	-1.823864	3.452613
52	8	0	4.892484	-0.232451	1.506108
53	8	0	3.271825	-2.131203	-0.576302
54	8	0	0.775122	3.657365	-0.234828
55	8	0	0.089636	-2.696838	-0.452411
56	8	0	-2.330207	3.313141	0.006433
57	8	0	-3.641449	0.683894	2.081150
58	6	0	-3.036412	-2.551845	0.616209
59	1	0	-2.342767	-2.764049	-0.195465
60	1	0	-2.594762	-2.995111	1.507895
61	6	0	-4.402615	-3.160002	0.320255
62	1	0	-4.307575	-4.241736	0.202862
63	1	0	-5.107819	-2.963517	1.130566
64	1	0	-4.821660	-2.756782	-0.603686

Structure 13 A_1G^-E (M06-2X, Gas Phase)

Energy (Hartrees): = -1755.1482904
No imaginary frequencies

Standard orientation:

Center Number	Atomic Number	Atomic Type	Coordinates (Angstroms) X	Y	Z

1	6	0	-3.186157	1.450259	-0.121254
2	1	0	-2.965466	2.361432	0.436770
3	1	0	-3.191736	1.652361	-1.193240
4	6	0	-2.148720	0.383685	0.198655
5	1	0	-2.385416	-0.053950	1.171444
6	6	0	-0.748318	0.967339	0.259507
7	1	0	-0.678960	1.596501	1.147800
8	6	0	0.330012	-0.117177	0.335995
9	1	0	0.245985	-0.796930	-0.514583
10	6	0	1.708940	0.522984	0.351250
11	1	0	1.824915	1.075479	-0.581851
12	6	0	2.932332	-0.419855	0.424914
13	1	0	3.783257	0.233247	0.633376
14	6	0	2.869101	-1.351454	1.566931
15	7	0	2.842192	-2.089431	2.445503
16	8	0	-4.478438	1.028270	0.312892
17	8	0	-2.165039	-0.642701	-0.792167
18	8	0	-0.476123	1.762440	-0.900507
19	8	0	0.093540	-0.812856	1.557563
20	8	0	1.758688	1.431520	1.451144
21	6	0	-5.215242	0.295361	-0.550394
22	6	0	-2.899477	-1.750571	-0.528787
23	6	0	-0.285043	-2.118319	1.509242
24	6	0	-0.293026	3.094861	-0.719157
25	6	0	2.250929	2.671019	1.202662
26	6	0	-6.431325	-0.257030	0.129469
27	6	0	-2.844429	-2.702389	-1.679825
28	6	0	-0.524797	-2.644773	2.887989
29	6	0	2.093833	3.567042	2.389602
30	1	0	-7.155130	-0.570355	-0.618441
31	1	0	-6.101153	-1.123779	0.708054
32	1	0	-6.858830	0.473096	0.814559
33	1	0	-0.916801	-3.655437	2.819355
34	1	0	0.424387	-2.636251	3.427744
35	1	0	-1.226366	-1.994535	3.410947
36	1	0	-1.802309	-2.922047	-1.912258
37	1	0	-3.379019	-3.611930	-1.420584
38	1	0	-3.306620	-2.218394	-2.542169
39	1	0	2.810824	4.381355	2.323039
40	1	0	1.080857	3.975285	2.342920
41	1	0	2.207253	3.009058	3.316907
42	6	0	0.185311	3.750979	-1.976806
43	1	0	-0.221964	3.257870	-2.856954
44	1	0	-0.075244	4.806288	-1.953674
45	1	0	1.274926	3.656812	-1.987511
46	7	0	3.170231	-1.128330	-0.816130
47	6	0	4.003412	-0.625638	-1.799677
48	6	0	4.707328	0.691103	-1.537084
49	1	0	5.196540	0.973956	-2.465249
50	1	0	4.033269	1.488937	-1.219545
51	1	0	5.473000	0.563457	-0.766589
52	8	0	-4.897326	0.095527	-1.689801
53	8	0	-3.510214	-1.900469	0.494359
54	8	0	-0.462480	3.643241	0.335489
55	8	0	-0.390740	-2.731745	0.484351
56	8	0	2.713670	2.984475	0.138920
57	8	0	4.166051	-1.235005	-2.833051
58	6	0	2.561693	-2.446603	-1.058220
59	1	0	1.710761	-2.559856	-0.388233
60	1	0	2.184751	-2.445767	-2.081585
61	6	0	3.555136	-3.586280	-0.863665
62	1	0	3.062232	-4.541451	-1.054299
63	1	0	4.385289	-3.479605	-1.561771
64	1	0	3.938142	-3.599341	0.158408

Structure 13 A_1G^-E (M06-2X, $CHCl_3$)

Energy (Hartrees): = -1755.1789547
No imaginary frequencies

Standard orientation:

Center Number	Atomic Number	Atomic Type	Coordinates (Angstroms) X	Y	Z
1	6	0	-3.201792	1.341580	0.057207
2	1	0	-3.006998	2.199649	0.702139
3	1	0	-3.201629	1.652116	-0.988395
4	6	0	-2.136828	0.279761	0.288818
5	1	0	-2.331747	-0.223151	1.239466
6	6	0	-0.755660	0.912792	0.334660
7	1	0	-0.695428	1.545495	1.221227
8	6	0	0.373234	-0.122923	0.387261
9	1	0	0.273613	-0.836006	-0.433270
10	6	0	1.721349	0.573887	0.304320

11	1	0	1.757023	1.108782	-0.645023
12	6	0	2.984109	-0.318371	0.324836
13	1	0	3.829227	0.370561	0.401837
14	6	0	3.070238	-1.157678	1.533856
15	7	0	3.165222	-1.824230	2.463220
16	8	0	-4.494962	0.862263	0.431660
17	8	0	-2.140562	-0.682245	-0.767881
18	8	0	-0.549765	1.710255	-0.836750
19	8	0	0.258136	-0.784888	1.646861
20	8	0	1.799644	1.500492	1.385437
21	6	0	-5.229980	0.235229	-0.507010
22	6	0	-2.858902	-1.812175	-0.589119
23	6	0	-0.167632	-2.073159	1.677682
24	6	0	-0.415626	3.052288	-0.682013
25	6	0	2.244470	2.752362	1.103816
26	6	0	-6.489588	-0.317051	0.083503
27	6	0	-2.721482	-2.718568	-1.769555
28	6	0	-0.223789	-2.573244	3.084621
29	6	0	2.160892	3.633448	2.305921
30	1	0	-7.184463	-0.575245	-0.712035
31	1	0	-6.224178	-1.217396	0.643780
32	1	0	-6.935754	0.396353	0.775778
33	1	0	-0.599420	-3.593040	3.092596
34	1	0	0.780262	-2.531631	3.512608
35	1	0	-0.870141	-1.924005	3.677722
36	1	0	-1.665372	-2.888568	-1.981778
37	1	0	-3.226020	-3.659928	-1.566813
38	1	0	-3.173593	-2.229731	-2.635503
39	1	0	2.690057	4.563372	2.113764
40	1	0	1.104989	3.842634	2.493953
41	1	0	2.570149	3.123738	3.178185
42	6	0	-0.073623	3.705302	-1.981980
43	1	0	-0.723745	3.334527	-2.774656
44	1	0	-0.158713	4.784377	-1.880250
45	1	0	0.957453	3.442603	-2.233367
46	7	0	3.125860	-1.120586	-0.876427
47	6	0	3.797360	-0.651072	-1.984605
48	6	0	4.439438	0.715618	-1.911378
49	1	0	4.781785	0.964066	-2.913121
50	1	0	3.762764	1.495455	-1.556871
51	1	0	5.305122	0.687724	-1.243335
52	8	0	-4.887863	0.127590	-1.654884
53	8	0	-3.526632	-2.018195	0.389211
54	8	0	-0.541496	3.603872	0.378113
55	8	0	-0.442388	-2.695940	0.687775
56	8	0	2.623029	3.072911	0.008887
57	8	0	3.876574	-1.336678	-2.986560
58	6	0	2.586458	-2.488658	-0.936824
59	1	0	1.826692	-2.592566	-0.162967
60	1	0	2.091464	-2.606262	-1.901462
61	6	0	3.669720	-3.545020	-0.761584
62	1	0	3.227271	-4.541610	-0.824797
63	1	0	4.420373	-3.451695	-1.547501
64	1	0	4.157453	-3.448484	0.211013

Structure 13 B_1G^-Z (M06-2X, Gas Phase)

Energy (Hartrees): = -1755.146382
No imaginary frequencies

Standard orientation:

Center Number	Atomic Number	Atomic Type	Coordinates (Angstroms) X	Y	Z
1	6	0	-3.253446	1.266257	0.181607
2	1	0	-3.035575	2.110425	0.837846
3	1	0	-3.296530	1.598409	-0.856758
4	6	0	-2.163850	0.209281	0.332682
5	1	0	-2.318277	-0.343787	1.263400
6	6	0	-0.798890	0.879687	0.353849
7	1	0	-0.726398	1.521584	1.232408
8	6	0	0.385706	-0.097280	0.363359
9	1	0	0.251967	-0.862298	-0.404291
10	6	0	1.670297	0.676821	0.108716
11	1	0	1.617183	1.098725	-0.895380
12	6	0	3.004159	-0.089188	0.169330
13	1	0	3.763724	0.690884	0.053095
14	6	0	3.300231	-0.702730	1.476204
15	7	0	3.630645	-1.111126	2.496383
16	8	0	-4.522802	0.769565	0.591067
17	8	0	-2.167645	-0.705688	-0.760762
18	8	0	-0.667214	1.686946	-0.830140
19	8	0	0.480595	-0.703957	1.642194
20	8	0	1.733318	1.738766	1.063179
21	6	0	-5.283655	0.178022	-0.356083

22	6	0	-2.877068	-1.847975	-0.608609
23	6	0	0.016259	-1.979443	1.769050
24	6	0	-0.600586	3.029421	-0.689852
25	6	0	2.250207	2.913459	0.635232
26	6	0	-6.484838	-0.472018	0.261243
27	6	0	-2.536897	-2.824023	-1.691954
28	6	0	0.580552	-2.623566	2.993753
29	6	0	2.191661	3.952917	1.709673
30	1	0	-7.229990	-0.661687	-0.507247
31	1	0	-6.143763	-1.417637	0.688796
32	1	0	-6.889960	0.144940	1.061572
33	1	0	-0.006966	-3.502369	3.246149
34	1	0	1.607678	-2.909453	2.753007
35	1	0	0.618056	-1.915454	3.820382
36	1	0	-1.550478	-3.231678	-1.458523
37	1	0	-3.273036	-3.623164	-1.704608
38	1	0	-2.494686	-2.316069	-2.654704
39	1	0	2.810100	4.800374	1.426094
40	1	0	1.149816	4.266154	1.805763
41	1	0	2.514493	3.528565	2.659632
42	6	0	-0.329529	3.692655	-2.007484
43	1	0	-0.844454	3.173447	-2.813888
44	1	0	-0.630932	4.735732	-1.952725
45	1	0	0.748034	3.639966	-2.184987
46	7	0	3.180165	-0.993359	-0.958240
47	6	0	2.692216	-2.274904	-0.876706
48	6	0	2.983145	-3.204787	-2.034156
49	1	0	2.654074	-4.196596	-1.736389
50	1	0	2.423046	-2.895487	-2.919632
51	1	0	4.043365	-3.227817	-2.289225
52	8	0	-5.001309	0.167561	-1.521658
53	8	0	-3.653971	-2.027954	0.285548
54	8	0	-0.713866	3.593276	0.364582
55	8	0	-0.731342	-2.477862	0.978876
56	8	0	2.673824	3.064326	-0.480994
57	8	0	2.039036	-2.634879	0.080910
58	6	0	4.019174	-0.493204	-2.049229
59	1	0	3.744486	-1.012156	-2.966377
60	1	0	3.765278	0.560122	-2.200523
61	6	0	5.509157	-0.645398	-1.760071
62	1	0	6.101451	-0.225186	-2.574723
63	1	0	5.783104	-0.124890	-0.839584
64	1	0	5.776494	-1.697048	-1.641314

Structure 13 B_1G^-Z (M06-2X, $CHCl_3$)

Energy (Hartrees): = -1755.1803446
No imaginary frequencies

Standard orientation:

Center Number	Atomic Number	Atomic Type	Coordinates (Angstroms) X	Y	Z
1	6	0	-3.241745	1.194086	0.272713
2	1	0	-3.055773	1.995611	0.988542
3	1	0	-3.287942	1.600612	-0.738765
4	6	0	-2.127056	0.159733	0.371481
5	1	0	-2.238069	-0.411711	1.296512
6	6	0	-0.775800	0.859509	0.361674
7	1	0	-0.705257	1.501748	1.240346
8	6	0	0.428849	-0.092375	0.353120
9	1	0	0.310757	-0.856116	-0.417773
10	6	0	1.699351	0.706835	0.098885
11	1	0	1.638982	1.131248	-0.903213
12	6	0	3.043153	-0.044680	0.153439
13	1	0	3.801029	0.736002	0.030076
14	6	0	3.348484	-0.634844	1.470217
15	7	0	3.673846	-1.011170	2.504472
16	8	0	-4.507010	0.641487	0.636500
17	8	0	-2.146918	-0.732683	-0.744150
18	8	0	-0.684194	1.661680	-0.826887
19	8	0	0.538918	-0.703589	1.632417
20	8	0	1.752707	1.760240	1.063870
21	6	0	-5.251206	0.086561	-0.338575
22	6	0	-2.826117	-1.890327	-0.610904
23	6	0	0.093119	-1.979176	1.766827
24	6	0	-0.714280	3.009188	-0.704655
25	6	0	2.099395	2.995402	0.625874
26	6	0	-6.471000	-0.568204	0.230950
27	6	0	-2.543991	-2.803580	-1.762006
28	6	0	0.641209	-2.607049	3.006584
29	6	0	2.076133	3.985069	1.744280
30	1	0	-7.153217	-0.835361	-0.572508
31	1	0	-6.148396	-1.468699	0.758694
32	1	0	-6.957213	0.091918	0.949389
33	1	0	0.003248	-3.433516	3.312417

34	1	0	1.635249	-2.986126	2.754251
35	1	0	0.749476	-1.873463	3.804368
36	1	0	-1.514532	-3.158124	-1.670340
37	1	0	-3.227814	-3.648432	-1.735803
38	1	0	-2.641223	-2.260075	-2.702382
39	1	0	2.427356	4.949114	1.385073
40	1	0	1.050118	4.070429	2.107324
41	1	0	2.699681	3.628478	2.565248
42	6	0	-0.564097	3.667287	-2.039368
43	1	0	-1.299214	3.263142	-2.736821
44	1	0	-0.687986	4.741774	-1.929675
45	1	0	0.432518	3.446044	-2.427071
46	7	0	3.218798	-0.961594	-0.961382
47	6	0	2.767865	-2.250139	-0.858899
48	6	0	3.047856	-3.179298	-2.014063
49	1	0	2.741210	-4.178003	-1.712570
50	1	0	2.469530	-2.878456	-2.891029
51	1	0	4.104239	-3.185139	-2.287006
52	8	0	-4.948825	0.115348	-1.502493
53	8	0	-3.555008	-2.127993	0.313861
54	8	0	-0.837956	3.570995	0.351079
55	8	0	-0.638040	-2.501420	0.972527
56	8	0	2.369581	3.223395	-0.523796
57	8	0	2.151516	-2.618209	0.127159
58	6	0	4.013105	-0.451136	-2.085213
59	1	0	3.729710	-0.995469	-2.984089
60	1	0	3.720481	0.590226	-2.246122
61	6	0	5.510814	-0.554244	-1.831294
62	1	0	6.065526	-0.153356	-2.682109
63	1	0	5.798906	0.013783	-0.943116
64	1	0	5.807703	-1.594471	-1.680589

Structure 13 B_1G^-E (M06-2X, Gas Phase)

Energy (Hartrees): = -1755.1411518
No imaginary frequencies

Standard orientation:

Center Number	Atomic Number	Atomic Type	Coordinates (Angstroms) X	Y	Z
1	6	0	-3.189307	1.463807	-0.097061
2	1	0	-2.954999	2.362193	0.476209
3	1	0	-3.173312	1.681612	-1.165818
4	6	0	-2.174088	0.372927	0.218118
5	1	0	-2.419885	-0.071351	1.186587
6	6	0	-0.768743	0.944800	0.279291
7	1	0	-0.697244	1.596191	1.151342
8	6	0	0.310649	-0.138104	0.392399
9	1	0	0.199882	-0.867943	-0.412358
10	6	0	1.686451	0.503312	0.334487
11	1	0	1.765266	1.027324	-0.617973
12	6	0	2.929187	-0.419317	0.385393
13	1	0	3.743670	0.242280	0.706879
14	6	0	2.872471	-1.435297	1.447588
15	7	0	2.866925	-2.191356	2.310950
16	8	0	-4.497156	1.068660	0.310010
17	8	0	-2.196018	-0.642866	-0.782028
18	8	0	-0.494593	1.709280	-0.901231
19	8	0	0.121508	-0.755927	1.663536
20	8	0	1.760201	1.448517	1.401831
21	6	0	-5.242801	0.382132	-0.583981
22	6	0	-2.973364	-1.728145	-0.547969
23	6	0	-0.344150	-2.032000	1.709099
24	6	0	-0.270545	3.039096	-0.752294
25	6	0	2.320087	2.649205	1.109540
26	6	0	-6.488235	-0.145392	0.061238
27	6	0	-2.879145	-2.694943	-1.684484
28	6	0	-0.448438	-2.504247	3.124638
29	6	0	2.201675	3.599366	2.258433
30	1	0	-7.208604	-0.415451	-0.706604
31	1	0	-6.196994	-1.036085	0.623851
32	1	0	-6.903388	0.582401	0.756430
33	1	0	-1.008910	-3.434577	3.149013
34	1	0	0.565121	-2.662689	3.500374
35	1	0	-0.926453	-1.742935	3.740013
36	1	0	-1.831282	-2.921074	-1.881289
37	1	0	-3.427022	-3.598931	-1.433731
38	1	0	-3.310307	-2.220794	-2.568405
39	1	0	2.946643	4.384134	2.154281
40	1	0	1.202390	4.038570	2.203088
41	1	0	2.303626	3.075634	3.206998
42	6	0	0.210483	3.652296	-2.030826
43	1	0	-0.221975	3.150698	-2.894031

44	1	0	-0.020034	4.714772	-2.029375
45	1	0	1.297044	3.527352	-2.052592
46	7	0	3.303755	-0.905932	-0.932508
47	6	0	3.152292	-2.174123	-1.476235
48	6	0	2.304940	-3.199472	-0.763634
49	1	0	2.034318	-3.942958	-1.509945
50	1	0	2.886542	-3.687858	0.020033
51	1	0	1.401376	-2.794566	-0.313279
52	8	0	-4.912325	0.202067	-1.722961
53	8	0	-3.649618	-1.847905	0.436037
54	8	0	-0.409157	3.615991	0.291477
55	8	0	-0.606557	-2.668550	0.727366
56	8	0	2.809742	2.890884	0.039026
57	8	0	3.693782	-2.434022	-2.528641
58	6	0	4.153770	0.031350	-1.685194
59	1	0	3.942787	-0.117862	-2.742246
60	1	0	3.853685	1.046281	-1.412155
61	6	0	5.634708	-0.195326	-1.405623
62	1	0	6.239608	0.531045	-1.951219
63	1	0	5.859118	-0.086965	-0.340720
64	1	0	5.918713	-1.198019	-1.725352

Structure 13 B_1G^-E (M06-2X, $CHCl_3$)

Energy (Hartrees): = -1755.1721466
No imaginary frequencies

Standard orientation:

Center Number	Atomic Number	Atomic Type	Coordinates (Angstroms) X	Y	Z
1	6	0	-3.185818	1.486848	-0.037175
2	1	0	-2.948981	2.366685	0.562859
3	1	0	-3.172085	1.743173	-1.096793
4	6	0	-2.173264	0.387476	0.249258
5	1	0	-2.400002	-0.065460	1.217717
6	6	0	-0.765868	0.959193	0.280328
7	1	0	-0.688092	1.648961	1.122153
8	6	0	0.317700	-0.118134	0.424055
9	1	0	0.205546	-0.874845	-0.355199
10	6	0	1.696842	0.513717	0.340374
11	1	0	1.761299	1.055027	-0.604286
12	6	0	2.925327	-0.430994	0.339401
13	1	0	3.772000	0.211927	0.609573
14	6	0	2.901927	-1.430295	1.417793
15	7	0	2.921337	-2.177108	2.288856
16	8	0	-4.499673	1.090790	0.361512
17	8	0	-2.210707	-0.617053	-0.766590
18	8	0	-0.529723	1.666047	-0.942263
19	8	0	0.152091	-0.693204	1.720857
20	8	0	1.804553	1.429027	1.427226
21	6	0	-5.269307	0.463634	-0.549134
22	6	0	-2.986926	-1.701078	-0.551176
23	6	0	-0.365946	-1.941597	1.829280
24	6	0	-0.170574	2.972253	-0.867598
25	6	0	2.491984	2.574766	1.190748
26	6	0	-6.547807	-0.010456	0.067962
27	6	0	-2.856233	-2.678486	-1.674713
28	6	0	-0.434715	-2.360403	3.262053
29	6	0	2.408092	3.504431	2.356071
30	1	0	-7.262369	-0.257239	-0.713715
31	1	0	-6.318700	-0.907046	0.649882
32	1	0	-6.953266	0.741396	0.744321
33	1	0	-0.955678	-3.311418	3.337479
34	1	0	0.584082	-2.456347	3.644912
35	1	0	-0.945191	-1.593871	3.846559
36	1	0	-1.802649	-2.896320	-1.853392
37	1	0	-3.398867	-3.588275	-1.430426
38	1	0	-3.271874	-2.226988	-2.578483
39	1	0	3.135802	4.303541	2.237689
40	1	0	1.399083	3.923379	2.374017
41	1	0	2.571279	2.963187	3.287913
42	6	0	0.132245	3.521504	-2.224835
43	1	0	-0.589873	3.158004	-2.955287
44	1	0	0.136855	4.608015	-2.184114
45	1	0	1.124247	3.169026	-2.521350
46	7	0	3.223387	-0.945253	-0.989648
47	6	0	2.992735	-2.201787	-1.510629
48	6	0	2.178565	-3.207655	-0.737165
49	1	0	1.809372	-3.932564	-1.460772
50	1	0	2.818356	-3.729368	-0.022354
51	1	0	1.333890	-2.784419	-0.199150
52	8	0	-4.940548	0.299303	-1.694110
53	8	0	-3.695052	-1.820811	0.412712
54	8	0	-0.091853	3.572821	0.169946

55	8	0	-0.703611	-2.595341	0.879514
56	8	0	3.063648	2.779483	0.153410
57	8	0	3.452732	-2.488844	-2.601207
58	6	0	4.054399	-0.033178	-1.796136
59	1	0	3.777217	-0.173274	-2.839164
60	1	0	3.799204	0.988803	-1.507360
61	6	0	5.540948	-0.292972	-1.596559
62	1	0	6.129351	0.407768	-2.192669
63	1	0	5.825414	-0.165092	-0.548297
64	1	0	5.790861	-1.308283	-1.907991

Structure 13 R-X (M06-2X, Gas Phase)

Energy (Hartrees): = - 1755.1495784
No imaginary frequencies

Standard orientation:

Center Number	Atomic Number	Atomic Type	Coordinates (Angstroms) X	Y	Z
1	8	0	4.083786	0.431345	-2.127391
2	8	0	1.641957	-0.932021	-0.890158
3	8	0	2.588269	-2.431894	0.493053
4	8	0	-0.033079	1.431818	-1.096139
5	8	0	-0.414460	3.243722	0.186560
6	8	0	-0.813171	-1.413430	0.975632
7	8	0	-0.499185	-3.160297	-0.410316
8	8	0	-2.631561	0.964293	0.808512
9	8	0	-3.310110	2.481018	-0.711448
10	8	0	-4.673949	-0.577399	-0.376411
11	8	0	-5.608110	-2.501105	0.285202
12	7	0	2.376730	2.827795	1.937587
13	7	0	3.890909	0.369053	0.133123
14	6	0	5.405801	0.883636	2.049944
15	1	0	6.282411	0.977114	1.406421
16	1	0	5.734817	0.509285	3.021426
17	1	0	4.978134	1.876487	2.191521
18	6	0	4.375611	-0.067288	1.447312
19	1	0	4.764884	-1.081946	1.363292
20	1	0	3.508529	-0.139255	2.107188
21	6	0	4.512404	0.053400	-1.056829
22	6	0	5.727867	-0.844966	-0.964330
23	1	0	5.416739	-1.848196	-0.658932
24	1	0	6.463289	-0.486954	-0.244493
25	1	0	6.171971	-0.892315	-1.954992
26	6	0	2.598366	1.029146	0.048949
27	1	0	2.529779	1.492071	-0.937940
28	6	0	2.469883	2.060327	1.089010
29	6	0	1.457553	-0.004379	0.171445
30	1	0	1.529472	-0.526765	1.127256
31	6	0	2.263904	-2.101503	-0.616991
32	6	0	2.465312	-2.886421	-1.874347
33	1	0	3.055637	-3.772948	-1.657985
34	1	0	2.952493	-2.252290	-2.617228
35	1	0	1.482885	-3.175327	-2.252710
36	6	0	0.061185	0.592004	0.053422
37	1	0	-0.160631	1.170742	0.952139
38	6	0	-0.327435	2.749760	-0.899961
39	6	0	-0.557426	3.441891	-2.206668
40	1	0	-1.595857	3.247467	-2.487160
41	1	0	0.100331	3.047903	-2.979170
42	1	0	-0.418474	4.511627	-2.072579
43	6	0	-0.969204	-0.521104	-0.134878
44	1	0	-0.749571	-1.061325	-1.056262
45	6	0	-0.529851	-2.714106	0.702524
46	6	0	-0.228607	-3.472096	1.958681
47	1	0	-0.377287	-4.533910	1.779896
48	1	0	-0.843377	-3.118694	2.784296
49	1	0	0.823263	-3.292888	2.196643
50	6	0	-2.395227	-0.000375	-0.219605
51	1	0	-2.531728	0.480254	-1.189557
52	6	0	-3.108237	2.177902	0.431023
53	6	0	-3.284424	3.071043	1.620013
54	1	0	-3.894778	3.925352	1.339076
55	1	0	-2.290860	3.415699	1.915808
56	1	0	-3.727956	2.523480	2.450200
57	6	0	-3.395702	-1.122985	-0.057994
58	1	0	-3.159129	-1.955396	-0.727844
59	1	0	-3.394649	-1.489581	0.969755
60	6	0	-5.725944	-1.388907	-0.143794
61	6	0	-7.016547	-0.705659	-0.496926
62	1	0	-7.846611	-1.374366	-0.286731
63	1	0	-7.004886	-0.432654	-1.552880
64	1	0	-7.111088	0.215054	0.079802

Structure 37 A_1G^+Z (M06-2X, Gas Phase)

Energy (Hartrees): = -1946.8556253
No imaginary frequencies

Standard orientation:

Center Number	Atomic Number	Atomic Type	Coordinates (Angstroms) X	Y	Z
1	6	0	-4.287250	0.837451	-0.281266
2	1	0	-4.228215	1.718137	0.365130
3	1	0	-4.194319	1.159577	-1.320216
4	6	0	-3.202206	-0.158491	0.062088
5	1	0	-3.402350	-0.601420	1.039259
6	6	0	-1.839841	0.512392	0.108187
7	1	0	-1.805081	1.146111	0.994526
8	6	0	-0.681386	-0.482783	0.156499
9	1	0	-0.705440	-1.142074	-0.713384
10	6	0	0.645214	0.267021	0.179244
11	1	0	0.817022	0.693958	-0.810844
12	6	0	1.851877	-0.594806	0.601751
13	1	0	1.691282	-0.905464	1.636807
14	6	0	1.976685	-1.783942	-0.253328
15	7	0	2.088686	-2.686968	-0.953264
16	8	0	-5.530197	0.165074	-0.092840
17	8	0	-3.208458	-1.193803	-0.921986
18	8	0	-1.623431	1.322641	-1.053828
19	8	0	-0.845361	-1.254590	1.345297
20	8	0	0.560264	1.315254	1.137598
21	6	0	-6.626915	0.866322	-0.445522
22	6	0	-3.638263	-2.422039	-0.534025
23	6	0	-1.026833	-2.601592	1.208006
24	6	0	-1.547226	2.667991	-0.880898
25	6	0	1.093933	2.517916	0.824078
26	6	0	-7.873966	0.061116	-0.214788
27	6	0	-3.572420	-3.389489	-1.675199
28	6	0	-1.363009	-3.230174	2.523647
29	6	0	1.016773	3.440234	1.998743
30	1	0	-8.741689	0.657313	-0.483320
31	1	0	-7.836282	-0.848025	-0.816319
32	1	0	-7.924417	-0.239287	0.832331
33	1	0	-1.090464	-4.282382	2.495669
34	1	0	-0.873424	-2.712368	3.346032
35	1	0	-2.447070	-3.147641	2.640543
36	1	0	-2.522259	-3.656803	-1.812567
37	1	0	-4.143755	-4.279260	-1.423968
38	1	0	-3.939607	-2.929060	-2.591185
39	1	0	1.520150	4.373620	1.761835
40	1	0	-0.036860	3.625775	2.215442
41	1	0	1.470715	2.951272	2.862665
42	6	0	-1.156167	3.351129	-2.155244
43	1	0	-1.584545	2.843058	-3.017028
44	1	0	-1.467893	4.391734	-2.113160
45	1	0	-0.065884	3.306384	-2.222135
46	7	0	3.076159	0.189215	0.569905
47	6	0	3.516184	0.732485	1.763372
48	6	0	4.672795	1.700377	1.664185
49	1	0	4.955505	1.976820	2.676389
50	1	0	5.525134	1.264110	1.141598
51	1	0	4.359805	2.593834	1.116029
52	8	0	-6.574501	1.981600	-0.879449
53	8	0	-3.979632	-2.678388	0.586328
54	8	0	-1.739730	3.207386	0.172945
55	8	0	-0.958881	-3.165293	0.155328
56	8	0	1.551210	2.772248	-0.259307
57	8	0	2.979290	0.472234	2.819279
58	6	0	5.026854	-0.161554	-0.955446
59	6	0	5.913909	0.406054	-1.869159
60	6	0	5.385302	-1.338632	-0.303913
61	6	0	7.136770	-0.198232	-2.135682
62	1	0	5.645458	1.328890	-2.374024
63	6	0	6.610291	-1.941796	-0.567086
64	1	0	4.707699	-1.787674	0.412846
65	6	0	7.488206	-1.375337	-1.483711
66	1	0	7.817784	0.253068	-2.847504
67	1	0	6.876415	-2.858238	-0.054341
68	1	0	8.442428	-1.846401	-1.686289
69	6	0	3.684925	0.505086	-0.719359
70	1	0	2.988623	0.173320	-1.495177
71	1	0	3.767636	1.588245	-0.838097

Structure 37 A_1G^+Z (M06-2X, $CHCl_3$)

Energy (Hartrees): = -1946.8943881
No imaginary frequencies

Standard orientation:

Center Number	Atomic Number	Atomic Type	Coordinates (Angstroms) X	Y	Z
1	6	0	4.335244	-0.755981	-0.300746
2	1	0	4.330991	-1.641697	0.340333
3	1	0	4.265535	-1.069384	-1.344237
4	6	0	3.195918	0.168858	0.060844
5	1	0	3.362124	0.587365	1.053849
6	6	0	1.861665	-0.559347	0.070909
7	1	0	1.843020	-1.220977	0.937179
8	6	0	0.677325	0.403776	0.138511
9	1	0	0.691871	1.084056	-0.714933
10	6	0	-0.636893	-0.365263	0.136936
11	1	0	-0.781037	-0.806013	-0.850956
12	6	0	-1.860497	0.501577	0.511405
13	1	0	-1.679402	0.946559	1.492953
14	6	0	-2.050586	1.579958	-0.470162
15	7	0	-2.206079	2.395360	-1.262583
16	8	0	5.534972	-0.006085	-0.100586
17	8	0	3.161077	1.236235	-0.892912
18	8	0	1.687059	-1.333614	-1.120828
19	8	0	0.829271	1.141348	1.351207
20	8	0	-0.561647	-1.391797	1.117908
21	6	0	6.681720	-0.640330	-0.404572
22	6	0	3.474454	2.481161	-0.460838
23	6	0	0.841995	2.501158	1.278565
24	6	0	1.611431	-2.684237	-0.999688
25	6	0	-1.048454	-2.615280	0.799015
26	6	0	7.869221	0.237811	-0.152687
27	6	0	3.389434	3.467424	-1.581749
28	6	0	1.104058	3.097031	2.623197
29	6	0	-0.960466	-3.535196	1.973247
30	1	0	8.776667	-0.279728	-0.453292
31	1	0	7.761337	1.171453	-0.706570
32	1	0	7.912500	0.485460	0.909766
33	1	0	1.035271	4.179975	2.558610
34	1	0	0.389012	2.706833	3.349133
35	1	0	2.107272	2.805511	2.940415
36	1	0	2.349875	3.528352	-1.910690
37	1	0	3.725266	4.441472	-1.235009
38	1	0	3.994325	3.128466	-2.423693
39	1	0	-1.449880	-4.477107	1.738338
40	1	0	0.094510	-3.709469	2.196288
41	1	0	-1.417816	-3.062648	2.844283
42	6	0	1.281681	-3.318880	-2.312237
43	1	0	1.850815	-2.854430	-3.117019
44	1	0	1.479401	-4.386679	-2.259595
45	1	0	0.217844	-3.156117	-2.504600
46	7	0	-3.055768	-0.316800	0.621273
47	6	0	-3.462847	-0.679912	1.887876
48	6	0	-4.621451	-1.643328	1.966004
49	1	0	-4.903819	-1.740409	3.011554
50	1	0	-5.478594	-1.304352	1.381844
51	1	0	-4.312489	-2.620932	1.584163
52	8	0	6.708648	-1.769582	-0.815080
53	8	0	3.756062	2.733041	0.679831
54	8	0	1.767392	-3.258547	0.043547
55	8	0	0.671305	3.099041	0.253169
56	8	0	-1.471411	-2.887616	-0.293190
57	8	0	-2.893323	-0.270939	2.882299
58	6	0	-4.984797	0.098433	-0.918356
59	6	0	-5.757536	-0.266109	-2.022950
60	6	0	-5.369927	1.199200	-0.157743
61	6	0	-6.891587	0.460571	-2.363317
62	1	0	-5.465809	-1.125498	-2.619588
63	6	0	-6.507174	1.928189	-0.497925
64	1	0	-4.784448	1.497930	0.705562
65	6	0	-7.270265	1.562613	-1.599881
66	1	0	-7.481102	0.168006	-3.224807
67	1	0	-6.792299	2.785315	0.101272
68	1	0	-8.154232	2.131264	-1.864151
69	6	0	-3.752559	-0.728315	-0.592121
70	1	0	-3.044104	-0.663745	-1.422189
71	1	0	-4.019561	-1.784114	-0.517182

Structure 37 A_1G^+E (M06-2X, Gas Phase)

Energy (Hartrees): = -1946.8535335

No imaginary frequencies

Standard orientation:

Center Number	Atomic Number	Atomic Type	Coordinates (Angstroms) X	Y	Z
1	6	0	4.348100	-0.846599	-0.272672
2	1	0	4.284985	-1.739821	0.356335
3	1	0	4.269818	-1.150010	-1.318078
4	6	0	3.253534	0.133384	0.078233
5	1	0	3.447591	0.580387	1.053914
6	6	0	1.891410	-0.548911	0.117526
7	1	0	1.834586	-1.154984	1.022835
8	6	0	0.748396	0.461724	0.113456
9	1	0	0.831945	1.134428	-0.742414
10	6	0	-0.602155	-0.231368	0.056107
11	1	0	-0.688509	-0.758418	-0.895249
12	6	0	-1.789469	0.762215	0.202533
13	1	0	-1.572046	1.451455	1.018030
14	6	0	-1.878917	1.569047	-1.024062
15	7	0	-1.918370	2.152228	-2.011551
16	8	0	5.585453	-0.172594	-0.053594
17	8	0	3.248632	1.171063	-0.907699
18	8	0	1.712152	-1.392271	-1.021970
19	8	0	0.889239	1.200137	1.330658
20	8	0	-0.679596	-1.164535	1.123346
21	6	0	6.688831	-0.867234	-0.401976
22	6	0	3.446998	2.443212	-0.487359
23	6	0	0.821912	2.558266	1.255998
24	6	0	1.613441	-2.733435	-0.798316
25	6	0	-1.131867	-2.418819	0.826748
26	6	0	7.931914	-0.063093	-0.148063
27	6	0	3.298167	3.411313	-1.620519
28	6	0	1.167939	3.184438	2.570253
29	6	0	-1.312435	-3.225799	2.071098
30	1	0	8.803030	-0.655463	-0.414025
31	1	0	7.899924	0.852140	-0.740578
32	1	0	7.971499	0.225093	0.902969
33	1	0	0.838423	4.220134	2.571023
34	1	0	0.723158	2.626538	3.393491
35	1	0	2.255332	3.146543	2.665557
36	1	0	2.228701	3.609481	-1.734740
37	1	0	3.805037	4.339376	-1.368729
38	1	0	3.678489	2.988656	-2.548400
39	1	0	-1.387367	-4.276699	1.805590
40	1	0	-0.483117	-3.054449	2.755421
41	1	0	-2.242154	-2.898008	2.546353
42	6	0	1.301556	-3.465352	-2.065789
43	1	0	1.784485	-2.992484	-2.918728
44	1	0	1.605649	-4.503841	-1.962746
45	1	0	0.217795	-3.421994	-2.201288
46	7	0	-3.033430	0.077773	0.486612
47	6	0	-3.360650	-0.250499	1.787873
48	6	0	-2.696564	0.527167	2.907470
49	1	0	-3.155510	0.198541	3.836261
50	1	0	-1.623560	0.328856	2.942361
51	1	0	-2.856273	1.601238	2.787110
52	8	0	6.642997	-1.976832	-0.850160
53	8	0	3.660369	2.736163	0.657055
54	8	0	1.733419	-3.223676	0.288603
55	8	0	0.529320	3.135282	0.247543
56	8	0	-1.364400	-2.772242	-0.294370
57	8	0	-4.170784	-1.123795	2.021491
58	6	0	-5.092210	-0.124678	-0.888397
59	6	0	-6.212197	-0.668874	-0.260106
60	6	0	-5.260801	0.921587	-1.795764
61	6	0	-7.480924	-0.169188	-0.531360
62	1	0	-6.076786	-1.472646	0.453399
63	6	0	-6.529698	1.421063	-2.065473
64	1	0	-4.397895	1.344905	-2.297999
65	6	0	-7.642252	0.877289	-1.432725
66	1	0	-8.344819	-0.599643	-0.038821
67	1	0	-6.648980	2.230669	-2.775635
68	1	0	-8.631733	1.265095	-1.644574
69	6	0	-3.712927	-0.670116	-0.591446
70	1	0	-3.084745	-0.610538	-1.482234
71	1	0	-3.773570	-1.718594	-0.298676

Structure 37 A_1G^+E (M06-2X, $CHCl_3$)

Energy (Hartrees): = - 1946.8914331
No imaginary frequencies

Standard orientation:

Center Number	Atomic Number	Atomic Type	Coordinates (Angstroms) X	Y	Z
1	6	0	-4.320843	0.873277	-0.256503
2	1	0	-4.257082	1.751989	0.390902
3	1	0	-4.249140	1.191430	-1.298242
4	6	0	-3.227279	-0.114040	0.079966
5	1	0	-3.407515	-0.544358	1.064988
6	6	0	-1.857729	0.551391	0.089386
7	1	0	-1.787090	1.170373	0.984091
8	6	0	-0.722710	-0.470191	0.083595
9	1	0	-0.805737	-1.126282	-0.784454
10	6	0	0.633786	0.220572	0.052233
11	1	0	0.757520	0.713100	-0.913051
12	6	0	1.814858	-0.759668	0.289667
13	1	0	1.586952	-1.377690	1.158800
14	6	0	1.925228	-1.671779	-0.859432
15	7	0	1.993490	-2.354875	-1.778818
16	8	0	-5.557942	0.191108	-0.042741
17	8	0	-3.252809	-1.162073	-0.896622
18	8	0	-1.679891	1.371098	-1.069194
19	8	0	-0.874494	-1.227105	1.286487
20	8	0	0.676246	1.186906	1.093222
21	6	0	-6.669448	0.890405	-0.338172
22	6	0	-3.542881	-2.415636	-0.476403
23	6	0	-0.885937	-2.585439	1.192652
24	6	0	-1.580823	2.715189	-0.886806
25	6	0	1.109628	2.441172	0.781473
26	6	0	-7.903608	0.076174	-0.096236
27	6	0	-3.505722	-3.381905	-1.617029
28	6	0	-1.168215	-3.204899	2.522457
29	6	0	1.198298	3.289542	2.006404
30	1	0	-8.783749	0.672892	-0.322038
31	1	0	-7.880638	-0.815310	-0.725312
32	1	0	-7.925028	-0.251800	0.944258
33	1	0	-1.120055	-4.287239	2.435316
34	1	0	-0.447430	-2.846323	3.259382
35	1	0	-2.165781	-2.899032	2.842096
36	1	0	-2.466402	-3.492382	-1.935762
37	1	0	-3.891205	-4.344405	-1.290257
38	1	0	-4.082134	-2.996533	-2.458070
39	1	0	1.270620	4.334875	1.716755
40	1	0	0.332590	3.122135	2.646225
41	1	0	2.099501	2.998752	2.554891
42	6	0	-1.309010	3.406950	-2.182706
43	1	0	-2.029035	3.086661	-2.936473
44	1	0	-1.355574	4.482898	-2.035812
45	1	0	-0.310061	3.122772	-2.520925
46	7	0	3.060199	-0.056954	0.529489
47	6	0	3.408108	0.321719	1.810472
48	6	0	2.736948	-0.375039	2.973321
49	1	0	3.209112	-0.010820	3.882612
50	1	0	1.668386	-0.153408	3.010070
51	1	0	2.870119	-1.457452	2.909368
52	8	0	-6.633984	2.021961	-0.741420
53	8	0	-3.765078	-2.691677	0.672282
54	8	0	-1.687141	3.236787	0.189530
55	8	0	-0.688118	-3.163464	0.160286
56	8	0	1.389837	2.768526	-0.338508
57	8	0	4.252631	1.179800	1.987636
58	6	0	5.110552	0.088459	-0.878633
59	6	0	6.239206	0.675005	-0.303071
60	6	0	5.277162	-0.997824	-1.739764
61	6	0	7.509309	0.175964	-0.574236
62	1	0	6.111958	1.517836	0.365342
63	6	0	6.546730	-1.498012	-2.010492
64	1	0	4.410587	-1.450343	-2.209169
65	6	0	7.666014	-0.913031	-1.426181
66	1	0	8.377752	0.640705	-0.121520
67	1	0	6.661171	-2.339640	-2.683888
68	1	0	8.655929	-1.300157	-1.638851
69	6	0	3.728304	0.636470	-0.590471
70	1	0	3.095698	0.526702	-1.473082
71	1	0	3.786332	1.698974	-0.353484

Structure 37 B_1G^+Z (M06-2X, Gas Phase)

Energy (Hartrees): = -1946.8517449
No imaginary frequencies

Standard orientation:

Center Number	Atomic Number	Atomic Type	Coordinates (Angstroms) X	Y	Z

1	6	0	4.255481	0.556201	-0.409607
2	1	0	4.118400	1.281437	-1.216826
3	1	0	4.430269	1.098684	0.521706
4	6	0	3.046145	-0.343388	-0.275336
5	1	0	2.982209	-1.005206	-1.140938
6	6	0	1.767874	0.472810	-0.192450
7	1	0	1.586509	0.911971	-1.174210
8	6	0	0.547090	-0.343365	0.231019
9	1	0	0.707830	-0.783894	1.216987
10	6	0	-0.683891	0.548891	0.279192
11	1	0	-0.596991	1.236747	1.118699
12	6	0	-2.013263	-0.214072	0.386317
13	1	0	-2.135820	-0.786317	-0.537627
14	6	0	-2.026357	-1.195180	1.485859
15	7	0	-2.057500	-2.008796	2.294522
16	8	0	5.364506	-0.295757	-0.687288
17	8	0	3.200736	-1.138071	0.900466
18	8	0	1.891418	1.523204	0.774404
19	8	0	0.370742	-1.381365	-0.736059
20	8	0	-0.766500	1.287665	-0.947823
21	6	0	6.561010	0.318923	-0.779435
22	6	0	3.466624	-2.458410	0.730870
23	6	0	0.519464	-2.673153	-0.314322
24	6	0	1.881657	2.802931	0.325411
25	6	0	-1.021963	2.617580	-0.875270
26	6	0	7.652855	-0.677105	-1.050218
27	6	0	3.606158	-3.142091	2.055773
28	6	0	0.497197	-3.620121	-1.473821
29	6	0	-0.989714	3.241027	-2.237886
30	1	0	8.594894	-0.152496	-1.183073
31	1	0	7.721982	-1.371929	-0.212040
32	1	0	7.405893	-1.257937	-1.939211
33	1	0	0.160021	-4.594798	-1.129615
34	1	0	-0.127397	-3.245595	-2.282052
35	1	0	1.528775	-3.706862	-1.825766
36	1	0	2.603008	-3.246502	2.474708
37	1	0	4.044628	-4.125522	1.907637
38	1	0	4.207819	-2.539317	2.734590
39	1	0	-1.547251	4.174283	-2.220062
40	1	0	0.058300	3.451797	-2.464546
41	1	0	-1.380431	2.558453	-2.990777
42	6	0	1.794691	3.769964	1.464279
43	1	0	2.380409	3.422928	2.313578
44	1	0	2.122175	4.750560	1.129172
45	1	0	0.743381	3.820194	1.761276
46	7	0	-3.121476	0.725661	0.467224
47	6	0	-3.245363	1.468420	1.625995
48	6	0	-4.375483	2.468042	1.686188
49	1	0	-4.429097	2.830539	2.709128
50	1	0	-4.153954	3.307907	1.022955
51	1	0	-5.330876	2.026664	1.396820
52	8	0	6.696913	1.502448	-0.654208
53	8	0	3.537400	-2.984578	-0.344426
54	8	0	1.903012	3.087854	-0.841157
55	8	0	0.684192	-2.972026	0.830902
56	8	0	-1.212478	3.198961	0.156528
57	8	0	-2.464892	1.314039	2.538720
58	6	0	-5.038818	-0.272036	-0.778250
59	6	0	-5.722555	-0.453866	-1.981075
60	6	0	-5.343585	-1.087795	0.307642
61	6	0	-6.703440	-1.428742	-2.095032
62	1	0	-5.481671	0.173407	-2.834223
63	6	0	-6.324688	-2.069955	0.191469
64	1	0	-4.810877	-0.969658	1.245066
65	6	0	-7.007149	-2.241089	-1.005430
66	1	0	-7.226816	-1.560524	-3.034505
67	1	0	-6.549203	-2.703223	1.041273
68	1	0	-7.768351	-3.006695	-1.094770
69	6	0	-3.996783	0.827952	-0.686621
70	1	0	-4.487382	1.802161	-0.668045
71	1	0	-3.377132	0.817460	-1.589603

Structure 37 B_1G^+Z (M06-2X, $CHCl_3$)

Energy (Hartrees): = - 1946.892829
No imaginary frequencies

Standard orientation:

Center Number	Atomic Number	Atomic Type	Coordinates (Angstroms) X	Y	Z
1	6	0	4.292691	0.576772	-0.406993
2	1	0	4.164268	1.283935	-1.230792
3	1	0	4.457165	1.131833	0.518954
4	6	0	3.083079	-0.322652	-0.281119
5	1	0	3.017903	-0.974937	-1.153085

6	6	0	1.802134	0.488652	-0.190117
7	1	0	1.627031	0.946110	-1.164508
8	6	0	0.587587	-0.349682	0.210576
9	1	0	0.748810	-0.803228	1.190220
10	6	0	-0.657268	0.523139	0.266843
11	1	0	-0.577423	1.203235	1.113195
12	6	0	-1.978447	-0.260833	0.366572
13	1	0	-2.079032	-0.858080	-0.543841
14	6	0	-1.984969	-1.220692	1.485498
15	7	0	-1.993890	-2.021576	2.307289
16	8	0	5.407494	-0.280121	-0.659730
17	8	0	3.233361	-1.125869	0.892371
18	8	0	1.916192	1.514534	0.802724
19	8	0	0.433650	-1.370784	-0.778132
20	8	0	-0.752169	1.268472	-0.951554
21	6	0	6.601043	0.328990	-0.779814
22	6	0	3.509079	-2.441586	0.726162
23	6	0	0.553353	-2.670758	-0.385191
24	6	0	1.888731	2.807475	0.393509
25	6	0	-1.030424	2.594217	-0.869827
26	6	0	7.692737	-0.665147	-1.034857
27	6	0	3.672898	-3.115560	2.050924
28	6	0	0.508670	-3.589358	-1.563476
29	6	0	-1.048664	3.219464	-2.228420
30	1	0	8.640272	-0.145730	-1.153710
31	1	0	7.748379	-1.364385	-0.198517
32	1	0	7.459221	-1.239948	-1.932626
33	1	0	0.234755	-4.588411	-1.232172
34	1	0	-0.182228	-3.223458	-2.321501
35	1	0	1.515209	-3.618890	-1.990256
36	1	0	2.747714	-3.003139	2.618521
37	1	0	3.893810	-4.168936	1.898349
38	1	0	4.477162	-2.632620	2.608545
39	1	0	-1.588751	4.162696	-2.187830
40	1	0	-0.010109	3.413368	-2.509849
41	1	0	-1.486362	2.546533	-2.964743
42	6	0	1.850238	3.736579	1.563175
43	1	0	2.627379	3.470675	2.280278
44	1	0	1.974971	4.759509	1.217404
45	1	0	0.880510	3.627116	2.055105
46	7	0	-3.104312	0.658395	0.417477
47	6	0	-3.277124	1.400634	1.563700
48	6	0	-4.424737	2.376688	1.586031
49	1	0	-4.520896	2.749919	2.602827
50	1	0	-4.208344	3.215781	0.919466
51	1	0	-5.361621	1.913237	1.271174
52	8	0	6.732967	1.520270	-0.690079
53	8	0	3.592169	-2.967748	-0.350717
54	8	0	1.879421	3.125470	-0.765741
55	8	0	0.703401	-2.996955	0.757966
56	8	0	-1.200801	3.166036	0.172213
57	8	0	-2.519300	1.261241	2.505626
58	6	0	-5.095111	-0.277715	-0.770381
59	6	0	-5.806671	-0.462618	-1.957639
60	6	0	-5.449502	-1.016824	0.355748
61	6	0	-6.861085	-1.364173	-2.016809
62	1	0	-5.529651	0.106364	-2.840355
63	6	0	-6.505766	-1.924477	0.295451
64	1	0	-4.903554	-0.894055	1.285322
65	6	0	-7.213383	-2.100150	-0.886999
66	1	0	-7.404192	-1.498679	-2.945169
67	1	0	-6.769844	-2.496697	1.177410
68	1	0	-8.032850	-2.807882	-0.932365
69	6	0	-3.968705	0.739247	-0.751007
70	1	0	-4.377604	1.748951	-0.816098
71	1	0	-3.348068	0.607915	-1.641517

Structure 37 B_1G^+E (M06-2X, Gas Phase)

Energy (Hartrees): = -1946.85037
No imaginary frequencies

Standard orientation:

Center Number	Atomic Number	Atomic Type	Coordinates (Angstroms) X	Y	Z
1	6	0	4.200539	0.304945	-0.378031
2	1	0	4.152740	0.982726	-1.234984
3	1	0	4.425049	0.888456	0.517232
4	6	0	2.898395	-0.445731	-0.208624
5	1	0	2.766985	-1.147655	-1.033421
6	6	0	1.711777	0.505686	-0.186513
7	1	0	1.571522	0.894400	-1.195600
8	6	0	0.422930	-0.165450	0.282048
9	1	0	0.547234	-0.567828	1.289335

10	6	0	-0.732503	0.832916	0.276249
11	1	0	-0.572758	1.554145	1.076941
12	6	0	-2.109068	0.155331	0.411000
13	1	0	-2.162564	-0.589420	-0.389062
14	6	0	-2.217803	-0.606194	1.667933
15	7	0	-2.294159	-1.188731	2.654072
16	8	0	5.213102	-0.678619	-0.578527
17	8	0	2.955747	-1.180317	1.015646
18	8	0	1.943978	1.599319	0.708473
19	8	0	0.161725	-1.231952	-0.631420
20	8	0	-0.751039	1.510863	-0.978633
21	6	0	6.472318	-0.201227	-0.662482
22	6	0	3.027903	-2.532921	0.931049
23	6	0	0.077054	-2.499218	-0.125049
24	6	0	2.176544	2.820852	0.161175
25	6	0	-0.766082	2.868420	-0.962804
26	6	0	7.458140	-1.318297	-0.854439
27	6	0	3.078766	-3.148568	2.295187
28	6	0	-0.056426	-3.505424	-1.224968
29	6	0	-0.732970	3.423973	-2.351697
30	1	0	8.453370	-0.900342	-0.978452
31	1	0	7.430714	-1.977403	0.014473
32	1	0	7.177523	-1.909731	-1.726317
33	1	0	-0.559196	-4.388805	-0.839025
34	1	0	-0.584213	-3.089097	-2.080640
35	1	0	0.959227	-3.775945	-1.525844
36	1	0	2.057469	-3.156939	2.683286
37	1	0	3.441811	-4.169843	2.213877
38	1	0	3.703625	-2.557334	2.962309
39	1	0	-1.088131	4.451001	-2.333529
40	1	0	0.310626	3.408271	-2.676686
41	1	0	-1.324376	2.812602	-3.030983
42	6	0	2.295383	3.872314	1.219786
43	1	0	2.771369	3.472795	2.113153
44	1	0	2.846928	4.720063	0.821356
45	1	0	1.280465	4.190677	1.470875
46	7	0	-3.219507	1.063799	0.182940
47	6	0	-3.716187	1.988244	1.092295
48	6	0	-2.872850	2.340402	2.296564
49	1	0	-3.521409	2.867700	2.991563
50	1	0	-2.429795	1.481873	2.797790
51	1	0	-2.078933	3.015154	1.969634
52	8	0	6.731218	0.965716	-0.586967
53	8	0	3.006831	-3.130833	-0.108340
54	8	0	2.241749	3.004085	-1.022953
55	8	0	0.139332	-2.737792	1.044690
56	8	0	-0.758855	3.511242	0.050584
57	8	0	-4.776008	2.538836	0.888069
58	6	0	-4.717103	-0.530854	-0.960783
59	6	0	-4.435846	-1.557019	-1.858117
60	6	0	-5.662972	-0.742056	0.044939
61	6	0	-5.092369	-2.781849	-1.759639
62	1	0	-3.702953	-1.396359	-2.643486
63	6	0	-6.315367	-1.962360	0.145433
64	1	0	-5.881541	0.062628	0.740035
65	6	0	-6.030850	-2.985270	-0.756777
66	1	0	-4.868324	-3.574241	-2.463923
67	1	0	-7.048565	-2.118445	0.927615
68	1	0	-6.540217	-3.937900	-0.674817
69	6	0	-3.993725	0.793726	-1.033831
70	1	0	-4.700328	1.614682	-1.141689
71	1	0	-3.305707	0.804014	-1.884508

Structure 37 B_1G^+E (M06-2X, $CHCl_3$)

Energy (Hartrees): = -1946.8886364
No imaginary frequencies

Standard orientation:

Center Number	Atomic Number	Atomic Type	Coordinates (Angstroms) X	Y	Z
1	6	0	4.181040	0.339068	-0.480721
2	1	0	4.086071	0.954749	-1.379068
3	1	0	4.426223	0.979957	0.369067
4	6	0	2.902869	-0.427943	-0.221568
5	1	0	2.745899	-1.156602	-1.017805
6	6	0	1.700922	0.500760	-0.173952
7	1	0	1.538008	0.884397	-1.181274
8	6	0	0.430830	-0.189349	0.322892
9	1	0	0.574466	-0.562916	1.338600
10	6	0	-0.743126	0.788310	0.311339
11	1	0	-0.604186	1.501919	1.121902
12	6	0	-2.112850	0.090516	0.414623
13	1	0	-2.158180	-0.618671	-0.418002

14	6	0	-2.228900	-0.736500	1.628111
15	7	0	-2.318641	-1.387655	2.569021
16	8	0	5.208015	-0.637978	-0.658364
17	8	0	3.029579	-1.117663	1.024559
18	8	0	1.929362	1.594760	0.721359
19	8	0	0.172275	-1.282395	-0.558772
20	8	0	-0.765969	1.480431	-0.935772
21	6	0	6.453183	-0.156014	-0.826860
22	6	0	3.188739	-2.463271	0.986053
23	6	0	0.185199	-2.545331	-0.038448
24	6	0	2.142092	2.824446	0.185740
25	6	0	-0.807245	2.838776	-0.908048
26	6	0	7.444064	-1.265995	-1.001417
27	6	0	3.348818	-3.013588	2.367122
28	6	0	-0.036113	-3.558035	-1.114488
29	6	0	-0.825427	3.400806	-2.291773
30	1	0	8.440295	-0.848511	-1.125226
31	1	0	7.414352	-1.923091	-0.130541
32	1	0	7.172435	-1.860242	-1.875779
33	1	0	0.037302	-4.555653	-0.689280
34	1	0	-1.023883	-3.408488	-1.556588
35	1	0	0.711974	-3.424014	-1.897270
36	1	0	2.431933	-2.819405	2.927045
37	1	0	3.532821	-4.083674	2.313824
38	1	0	4.171757	-2.507048	2.873517
39	1	0	-0.799265	4.486146	-2.239613
40	1	0	0.039600	3.027573	-2.842006
41	1	0	-1.729457	3.070436	-2.807378
42	6	0	2.285212	3.854246	1.258944
43	1	0	2.989887	3.510361	2.016617
44	1	0	2.615473	4.792569	0.820519
45	1	0	1.310158	3.990758	1.732681
46	7	0	-3.229606	1.002366	0.230154
47	6	0	-3.667464	1.943544	1.141162
48	6	0	-2.846995	2.207767	2.380848
49	1	0	-3.523898	2.607870	3.133265
50	1	0	-2.330975	1.342146	2.790634
51	1	0	-2.109590	2.975340	2.134002
52	8	0	6.697089	1.020711	-0.834706
53	8	0	3.179551	-3.099726	-0.032216
54	8	0	2.185214	3.020578	-0.998680
55	8	0	0.350606	-2.766911	1.126973
56	8	0	-0.813724	3.468157	0.114968
57	8	0	-4.680367	2.582733	0.926519
58	6	0	-4.781345	-0.497938	-0.971872
59	6	0	-4.582265	-1.480985	-1.938620
60	6	0	-5.710579	-0.720968	0.047434
61	6	0	-5.306956	-2.670887	-1.896507
62	1	0	-3.858926	-1.313343	-2.730980
63	6	0	-6.430847	-1.907197	0.092444
64	1	0	-5.867007	0.043945	0.802082
65	6	0	-6.231613	-2.884792	-0.881461
66	1	0	-5.146584	-3.428757	-2.654828
67	1	0	-7.151590	-2.070463	0.885288
68	1	0	-6.794806	-3.810005	-0.845106
69	6	0	-3.996723	0.793914	-1.003671
70	1	0	-4.663987	1.647484	-1.108412
71	1	0	-3.298759	0.790525	-1.844876

Structure 37 A_1G^-Z (M06-2X, Gas Phase)

Energy (Hartrees): = - 1946.8560818
No imaginary frequencies

Standard orientation:

Center Number	Atomic Number	Atomic Type	Coordinates (Angstroms) X	Y	Z
1	6	0	4.261016	0.302857	0.138238
2	1	0	4.442504	1.246715	-0.376711
3	1	0	4.350950	0.434013	1.217638
4	6	0	2.877858	-0.220958	-0.211072
5	1	0	2.916484	-0.673843	-1.203155
6	6	0	1.826680	0.876958	-0.226605
7	1	0	1.991807	1.487556	-1.115962
8	6	0	0.414276	0.294815	-0.266673
9	1	0	0.228775	-0.316408	0.617362
10	6	0	-0.623822	1.401136	-0.335398
11	1	0	-0.554471	1.999882	0.572893
12	6	0	-2.092723	0.936858	-0.419081
13	1	0	-2.683053	1.858712	-0.481477
14	6	0	-2.366940	0.161313	-1.637017
15	7	0	-2.587163	-0.440394	-2.589344
16	8	0	5.257840	-0.603645	-0.339333
17	8	0	2.476680	-1.215071	0.735831
18	8	0	1.907369	1.707114	0.934027

19	8	0	0.381802	-0.518345	-1.440406
20	8	0	-0.352250	2.223910	-1.467809
21	6	0	5.615938	-1.624222	0.466789
22	6	0	2.658178	-2.511030	0.399205
23	6	0	0.081842	-1.832367	-1.314352
24	6	0	2.372896	2.973151	0.763532
25	6	0	-0.114731	3.544078	-1.232172
26	6	0	6.496055	-2.592721	-0.265548
27	6	0	2.285504	-3.408138	1.535917
28	6	0	0.189424	-2.525644	-2.634548
29	6	0	0.356426	4.233430	-2.474940
30	1	0	7.020408	-3.220005	0.450650
31	1	0	5.841906	-3.210691	-0.885985
32	1	0	7.192505	-2.067508	-0.916751
33	1	0	-0.031674	-3.581054	-2.503151
34	1	0	-0.517218	-2.065141	-3.327719
35	1	0	1.200642	-2.396596	-3.022572
36	1	0	1.284306	-3.152735	1.881914
37	1	0	2.326746	-4.442889	1.206978
38	1	0	2.998870	-3.233914	2.343978
39	1	0	0.118769	5.292410	-2.410406
40	1	0	1.443705	4.118396	-2.503286
41	1	0	-0.073138	3.779981	-3.365524
42	6	0	2.179723	3.783920	2.005732
43	1	0	2.391426	3.187888	2.892066
44	1	0	2.810796	4.667658	1.961802
45	1	0	1.129641	4.087188	2.027491
46	7	0	-2.486827	0.211417	0.791621
47	6	0	-2.750162	1.021138	1.883582
48	6	0	-3.257945	0.343515	3.135849
49	1	0	-3.423980	1.120684	3.876921
50	1	0	-4.190097	-0.192084	2.945624
51	1	0	-2.522157	-0.368642	3.515144
52	8	0	5.239045	-1.741788	1.600274
53	8	0	3.078870	-2.857982	-0.672707
54	8	0	2.843012	3.361150	-0.270200
55	8	0	-0.231981	-2.337009	-0.269004
56	8	0	-0.229593	4.047575	-0.152100
57	8	0	-2.560320	2.218266	1.829385
58	6	0	-4.479348	-1.253371	0.390971
59	6	0	-5.039830	-2.522696	0.240672
60	6	0	-5.293189	-0.134349	0.243029
61	6	0	-6.389021	-2.670226	-0.046911
62	1	0	-4.408534	-3.399728	0.345153
63	6	0	-6.647342	-0.281272	-0.048521
64	1	0	-4.881187	0.863626	0.354158
65	6	0	-7.198569	-1.546830	-0.192390
66	1	0	-6.810534	-3.661781	-0.162413
67	1	0	-7.268551	0.598862	-0.163500
68	1	0	-8.251510	-1.660374	-0.419623
69	6	0	-2.999918	-1.152800	0.716213
70	1	0	-2.421037	-1.702088	-0.024520
71	1	0	-2.792630	-1.647910	1.666379

Structure 37 A_1G^-Z (M06-2X, $CHCl_3$)

Energy (Hartrees): = -1946.8927614
No imaginary frequencies
Standard orientation:

Center Number	Atomic Number	Atomic Type	Coordinates (Angstroms) X	Y	Z
1	6	0	4.191914	0.171781	-0.084182
2	1	0	4.369164	1.033289	-0.728657
3	1	0	4.347921	0.446438	0.960107
4	6	0	2.774330	-0.337662	-0.291505
5	1	0	2.722895	-0.869074	-1.244537
6	6	0	1.775204	0.808050	-0.316118
7	1	0	1.959050	1.398934	-1.214948
8	6	0	0.325525	0.317164	-0.336021
9	1	0	0.129791	-0.342872	0.511335
10	6	0	-0.637486	1.492277	-0.296829
11	1	0	-0.477666	2.045446	0.628936
12	6	0	-2.138201	1.134833	-0.306708
13	1	0	-2.668037	2.092737	-0.333615
14	6	0	-2.540756	0.395977	-1.512528
15	7	0	-2.866727	-0.171150	-2.455561
16	8	0	5.145922	-0.814474	-0.486601
17	8	0	2.398585	-1.227270	0.764727
18	8	0	1.930312	1.635004	0.840098
19	8	0	0.172953	-0.393127	-1.567730
20	8	0	-0.367942	2.335356	-1.414691
21	6	0	5.560490	-1.706259	0.433118
22	6	0	2.556528	-2.551746	0.559489
23	6	0	-0.026120	-1.732374	-1.539014

24	6	0	2.434747	2.883905	0.663316
25	6	0	-0.107348	3.647609	-1.165554
26	6	0	6.434875	-2.747759	-0.192771
27	6	0	2.109121	-3.333576	1.752464
28	6	0	-0.113331	-2.281772	-2.925688
29	6	0	0.278247	4.360137	-2.421033
30	1	0	6.933027	-3.323643	0.583450
31	1	0	5.795231	-3.404556	-0.787698
32	1	0	7.161914	-2.286335	-0.860719
33	1	0	-0.332570	-3.345476	-2.881652
34	1	0	-0.891726	-1.749623	-3.475522
35	1	0	0.838573	-2.115740	-3.435004
36	1	0	1.142044	-2.970499	2.099578
37	1	0	2.054879	-4.388787	1.495496
38	1	0	2.840826	-3.184310	2.550434
39	1	0	0.222512	5.434065	-2.260387
40	1	0	1.309956	4.083758	-2.655347
41	1	0	-0.357328	4.053019	-3.250931
42	6	0	2.399171	3.656007	1.941767
43	1	0	2.902340	3.093583	2.729789
44	1	0	2.875318	4.622304	1.795680
45	1	0	1.356129	3.795263	2.233844
46	7	0	-2.527550	0.435924	0.915415
47	6	0	-2.766523	1.245475	2.005182
48	6	0	-3.250635	0.579389	3.268544
49	1	0	-3.461887	1.363167	3.991977
50	1	0	-4.152752	-0.009308	3.090708
51	1	0	-2.482552	-0.083837	3.672589
52	8	0	5.239806	-1.663078	1.591747
53	8	0	3.023233	-3.009937	-0.450082
54	8	0	2.838997	3.279066	-0.396735
55	8	0	-0.113151	-2.362305	-0.517783
56	8	0	-0.156855	4.124336	-0.066279
57	8	0	-2.576058	2.446473	1.937286
58	6	0	-4.265713	-1.316817	0.474582
59	6	0	-4.584004	-2.649107	0.206083
60	6	0	-5.254741	-0.344681	0.350457
61	6	0	-5.870681	-3.003867	-0.177376
62	1	0	-3.812916	-3.409111	0.291713
63	6	0	-6.545023	-0.699449	-0.037930
64	1	0	-5.024678	0.698127	0.545680
65	6	0	-6.856799	-2.027415	-0.301412
66	1	0	-6.103821	-4.041608	-0.387140
67	1	0	-7.304753	0.067344	-0.138001
68	1	0	-7.859771	-2.302370	-0.606173
69	6	0	-2.843970	-0.987559	0.895314
70	1	0	-2.143907	-1.492270	0.227630
71	1	0	-2.646601	-1.397184	1.886901

Structure 37 A_1G^-E (M06-2X, Gas Phase)

Energy (Hartrees): = -1946.8533995
No imaginary frequencies

Standard orientation:

Center Number	Atomic Number	Atomic Type	Coordinates (Angstroms) X	Y	Z
1	6	0	-4.166917	0.226172	-0.111708
2	1	0	-4.369093	1.126923	0.469848
3	1	0	-4.256473	0.434456	-1.178850
4	6	0	-2.769252	-0.288551	0.198755
5	1	0	-2.790751	-0.809110	1.158687
6	6	0	-1.759487	0.843330	0.284428
7	1	0	-1.955300	1.407318	1.196969
8	6	0	-0.321316	0.320265	0.320310
9	1	0	-0.127802	-0.294257	-0.560174
10	6	0	0.659067	1.482914	0.366062
11	1	0	0.518604	2.063478	-0.546485
12	6	0	2.172216	1.154782	0.421583
13	1	0	2.654410	2.099174	0.683360
14	6	0	2.508121	0.239843	1.528513
15	7	0	2.762796	-0.444226	2.413794
16	8	0	-5.145853	-0.729975	0.293053
17	8	0	-2.338348	-1.199447	-0.812901
18	8	0	-1.870602	1.720123	-0.841608
19	8	0	-0.235908	-0.462181	1.508968
20	8	0	0.327006	2.287673	1.498192
21	6	0	-5.490904	-1.683776	-0.599538
22	6	0	-2.518758	-2.521590	-0.579205
23	6	0	0.046012	-1.785996	1.410337
24	6	0	-2.310002	2.984071	-0.610627
25	6	0	0.182392	3.620453	1.290059
26	6	0	-6.352929	-2.725344	0.047361
27	6	0	-2.069307	-3.329047	-1.753923

28	6	0	0.060512	-2.426977	2.760444
29	6	0	-0.325269	4.308775	2.516603
30	1	0	-6.852835	-3.310408	-0.720138
31	1	0	-5.688348	-3.369254	0.629024
32	1	0	-7.070455	-2.268313	0.726728
33	1	0	0.089438	-3.506461	2.640778
34	1	0	0.952117	-2.078430	3.287397
35	1	0	-0.819254	-2.123348	3.327021
36	1	0	-1.028475	-3.089838	-1.973807
37	1	0	-2.178135	-4.385892	-1.527089
38	1	0	-2.685543	-3.054098	-2.611724
39	1	0	-0.086356	5.367686	2.460543
40	1	0	-1.411410	4.185543	2.519622
41	1	0	0.084387	3.853276	3.416020
42	6	0	-2.194689	3.828524	-1.841036
43	1	0	-2.378354	3.240055	-2.737865
44	1	0	-2.882500	4.666964	-1.766818
45	1	0	-1.170541	4.210393	-1.870172
46	7	0	2.701513	0.697464	-0.849004
47	6	0	3.224789	1.582709	-1.776500
48	6	0	3.246311	3.057118	-1.423950
49	1	0	3.552055	3.587178	-2.321914
50	1	0	2.283603	3.440961	-1.081878
51	1	0	3.988468	3.243848	-0.642452
52	8	0	-5.113356	-1.694441	-1.738275
53	8	0	-2.990331	-2.945078	0.441202
54	8	0	-2.701595	3.355820	0.461555
55	8	0	0.263915	-2.333300	0.363483
56	8	0	0.405735	4.141189	0.230358
57	8	0	3.667202	1.185085	-2.831755
58	6	0	3.941957	-1.468401	-0.660017
59	6	0	3.786171	-2.619255	0.108325
60	6	0	5.226882	-1.018515	-0.965601
61	6	0	4.898915	-3.310352	0.574981
62	1	0	2.786140	-2.966846	0.348216
63	6	0	6.338132	-1.706703	-0.494710
64	1	0	5.349713	-0.140676	-1.589969
65	6	0	6.176896	-2.853085	0.277413
66	1	0	4.766568	-4.200961	1.177701
67	1	0	7.332906	-1.352222	-0.737791
68	1	0	7.045240	-3.387590	0.644026
69	6	0	2.719690	-0.737922	-1.174618
70	1	0	1.822733	-1.206556	-0.770811
71	1	0	2.676767	-0.800059	-2.262145

Structure 37 A_1G^-E (M06-2X, $CHCl_3$)

Energy (Hartrees): = - 1946.8904742
No imaginary frequencies

Standard orientation:

Center Number	Atomic Number	Atomic Type	Coordinates (Angstroms) X	Y	Z
1	6	0	-4.150182	0.252801	0.131560
2	1	0	-4.284751	1.090475	0.817315
3	1	0	-4.334648	0.574929	-0.894212
4	6	0	-2.737864	-0.295872	0.266802
5	1	0	-2.671473	-0.888308	1.182539
6	6	0	-1.720219	0.830122	0.336927
7	1	0	-1.869069	1.367828	1.273878
8	6	0	-0.277889	0.316413	0.278008
9	1	0	-0.134249	-0.283812	-0.621639
10	6	0	0.695379	1.485096	0.279421
11	1	0	0.512073	2.069051	-0.623286
12	6	0	2.211510	1.169903	0.275774
13	1	0	2.698145	2.128838	0.469312
14	6	0	2.612436	0.323228	1.416099
15	7	0	2.941452	-0.301911	2.320377
16	8	0	-5.114029	-0.726197	0.526724
17	8	0	-2.408679	-1.121677	-0.853745
18	8	0	-1.900307	1.726273	-0.764396
19	8	0	-0.093317	-0.474110	1.452256
20	8	0	0.415822	2.276745	1.433421
21	6	0	-5.574260	-1.574635	-0.412966
22	6	0	-2.592275	-2.453973	-0.727374
23	6	0	0.093626	-1.811451	1.328609
24	6	0	-2.292827	2.998084	-0.496181
25	6	0	0.346186	3.622555	1.264914
26	6	0	-6.460278	-2.614338	0.198690
27	6	0	-2.216057	-3.166393	-1.985944
28	6	0	0.241121	-2.452222	2.669436
29	6	0	-0.047689	4.302848	2.533941
30	1	0	-6.976300	-3.163722	-0.584996

31	1	0	-5.828077	-3.295388	0.773674
32	1	0	-7.172749	-2.153950	0.883125
33	1	0	0.296787	-3.531271	2.550120
34	1	0	1.152027	-2.073941	3.139561
35	1	0	-0.605873	-2.179937	3.301072
36	1	0	-1.260845	-2.797953	-2.359440
37	1	0	-2.170660	-4.236364	-1.798287
38	1	0	-2.984569	-2.954254	-2.733853
39	1	0	0.102444	5.374950	2.433530
40	1	0	-1.106150	4.094223	2.708061
41	1	0	0.523824	3.904119	3.372143
42	6	0	-2.324208	3.820478	-1.743616
43	1	0	-2.825975	3.276815	-2.544312
44	1	0	-2.822586	4.765701	-1.543626
45	1	0	-1.293214	4.009915	-2.053356
46	7	0	2.700415	0.647390	-0.988200
47	6	0	3.236348	1.479131	-1.953742
48	6	0	3.257062	2.966486	-1.685571
49	1	0	3.553706	3.455317	-2.610402
50	1	0	2.293213	3.359310	-1.356864
51	1	0	4.000419	3.197390	-0.916832
52	8	0	-5.281092	-1.497141	-1.576794
53	8	0	-3.031664	-2.963049	0.269803
54	8	0	-2.554164	3.381398	0.612084
55	8	0	0.135878	-2.370788	0.264799
56	8	0	0.556748	4.152232	0.206544
57	8	0	3.688564	1.017928	-2.983630
58	6	0	3.861918	-1.538321	-0.633266
59	6	0	3.647446	-2.767598	-0.011411
60	6	0	5.153684	-1.015825	-0.683176
61	6	0	4.708468	-3.464900	0.556961
62	1	0	2.640043	-3.169653	0.042067
63	6	0	6.213923	-1.709659	-0.110013
64	1	0	5.331132	-0.065551	-1.175419
65	6	0	5.993736	-2.934702	0.513551
66	1	0	4.528984	-4.415720	1.045829
67	1	0	7.214304	-1.294030	-0.151060
68	1	0	6.820027	-3.471095	0.965661
69	6	0	2.690042	-0.797575	-1.243378
70	1	0	1.760139	-1.225812	-0.869615
71	1	0	2.695935	-0.917447	-2.326790

Structure 37 B_1G^-Z (M06-2X, Gas Phase)

Energy (Hartrees): = -1946.8534931
No imaginary frequencies

Standard orientation:

Center Number	Atomic Number	Atomic Type	Coordinates (Angstroms) X	Y	Z
1	6	0	-4.136704	1.138598	-0.008869
2	1	0	-4.013109	1.974604	0.681250
3	1	0	-4.109176	1.494320	-1.039960
4	6	0	-3.014491	0.127707	0.211374
5	1	0	-3.223428	-0.456136	1.112001
6	6	0	-1.690984	0.858696	0.369529
7	1	0	-1.729651	1.479953	1.264624
8	6	0	-0.464942	-0.060332	0.468189
9	1	0	-0.480636	-0.800621	-0.335164
10	6	0	0.796877	0.785456	0.374270
11	1	0	0.834921	1.230798	-0.620013
12	6	0	2.150501	0.080492	0.565025
13	1	0	2.890754	0.888992	0.556298
14	6	0	2.329811	-0.564121	1.878322
15	7	0	2.572597	-0.999653	2.911981
16	8	0	-5.412077	0.577125	0.282366
17	8	0	-2.882481	-0.756511	-0.899119
18	8	0	-1.490765	1.700866	-0.779875
19	8	0	-0.468858	-0.710674	1.729018
20	8	0	0.704315	1.823508	1.351090
21	6	0	-6.059104	-0.033258	-0.734881
22	6	0	-3.552227	-1.930309	-0.832540
23	6	0	-0.873285	-2.012441	1.765238
24	6	0	-1.510497	3.041198	-0.606579
25	6	0	1.182742	3.039595	0.996574
26	6	0	-7.274817	-0.750458	-0.231413
27	6	0	-3.102456	-2.854317	-1.921234
28	6	0	-0.415330	-2.670363	3.026571
29	6	0	0.973543	4.040423	2.088740
30	1	0	-7.925263	-0.993608	-1.067639
31	1	0	-6.922542	-1.668457	0.244318
32	1	0	-7.795694	-0.151669	0.514145
33	1	0	-1.006974	-3.564205	3.206107

34	1	0	0.633661	-2.937286	2.874888
35	1	0	-0.465554	-1.979031	3.866186
36	1	0	-2.106734	-3.212833	-1.651497
37	1	0	-3.791192	-3.691844	-1.991425
38	1	0	-3.044667	-2.316303	-2.866818
39	1	0	1.546584	4.937351	1.869547
40	1	0	-0.092509	4.275275	2.119240
41	1	0	1.261787	3.612345	3.048175
42	6	0	-1.156883	3.751426	-1.879161
43	1	0	-1.567522	3.227488	-2.740385
44	1	0	-1.518197	4.775487	-1.829985
45	1	0	-0.066445	3.759502	-1.956957
46	7	0	2.488091	-0.781710	-0.560487
47	6	0	2.103275	-2.103433	-0.549943
48	6	0	2.638694	-2.984209	-1.655942
49	1	0	2.375690	-4.008420	-1.405414
50	1	0	2.176229	-2.723412	-2.610612
51	1	0	3.721690	-2.890452	-1.757670
52	8	0	-5.677332	-0.010061	-1.871594
53	8	0	-4.380947	-2.169335	-0.000570
54	8	0	-1.751539	3.571013	0.443909
55	8	0	-1.496713	-2.522132	0.880540
56	8	0	1.685896	3.247912	-0.075993
57	8	0	1.366318	-2.529760	0.313760
58	6	0	4.853162	-0.228914	-1.148525
59	6	0	5.768562	0.489657	-1.918837
60	6	0	5.309700	-0.955835	-0.052849
61	6	0	7.119991	0.474062	-1.605488
62	1	0	5.417471	1.067202	-2.768613
63	6	0	6.666257	-0.966765	0.264747
64	1	0	4.613666	-1.510574	0.567799
65	6	0	7.572991	-0.256763	-0.509990
66	1	0	7.820196	1.036455	-2.211438
67	1	0	7.007553	-1.531546	1.123892
68	1	0	8.627216	-0.266156	-0.261018
69	6	0	3.387948	-0.202296	-1.547122
70	1	0	3.249265	-0.718717	-2.497710
71	1	0	3.077256	0.834440	-1.716084

Structure 37 B_1G^-Z (M06-2X, $CHCl_3$)

Energy (Hartrees): = - 1946.8932059
No imaginary frequencies

Standard orientation:

Center Number	Atomic Number	Atomic Type	Coordinates (Angstroms) X	Y	Z
1	6	0	-4.126124	1.107596	0.040471
2	1	0	-4.033453	1.908098	0.775082
3	1	0	-4.093066	1.521490	-0.968308
4	6	0	-2.990941	0.109188	0.234992
5	1	0	-3.175417	-0.484020	1.133882
6	6	0	-1.671351	0.853050	0.375205
7	1	0	-1.714396	1.480723	1.265669
8	6	0	-0.438461	-0.056546	0.478584
9	1	0	-0.443181	-0.803724	-0.317368
10	6	0	0.821505	0.793794	0.384795
11	1	0	0.853285	1.244460	-0.606863
12	6	0	2.178379	0.087435	0.570604
13	1	0	2.921752	0.892332	0.551995
14	6	0	2.355204	-0.529057	1.898647
15	7	0	2.579858	-0.925396	2.952180
16	8	0	-5.399782	0.508997	0.283157
17	8	0	-2.869959	-0.761569	-0.891250
18	8	0	-1.487123	1.680485	-0.785129
19	8	0	-0.446511	-0.695259	1.748900
20	8	0	0.728741	1.818740	1.375556
21	6	0	-6.027587	-0.067001	-0.759290
22	6	0	-3.522288	-1.941314	-0.842805
23	6	0	-0.850814	-1.990723	1.806533
24	6	0	-1.546513	3.024966	-0.638492
25	6	0	1.107188	3.071268	1.019085
26	6	0	-7.271329	-0.768257	-0.310063
27	6	0	-3.114523	-2.817323	-1.985010
28	6	0	-0.431999	-2.619805	3.094922
29	6	0	0.922103	4.027002	2.151822
30	1	0	-7.869090	-1.045427	-1.175035
31	1	0	-6.966647	-1.666113	0.232565
32	1	0	-7.842675	-0.135617	0.369123
33	1	0	-1.031708	-3.507735	3.281258
34	1	0	0.619387	-2.898771	2.986443
35	1	0	-0.510361	-1.911085	3.918490
36	1	0	-2.071907	-3.108422	-1.838931

37	1	0	-3.745522	-3.702318	-2.011854
38	1	0	-3.191475	-2.263281	-2.921417
39	1	0	1.281525	5.010507	1.860268
40	1	0	-0.140839	4.071452	2.395553
41	1	0	1.459256	3.664398	3.029414
42	6	0	-1.264140	3.715119	-1.935178
43	1	0	-1.860400	3.272849	-2.733655
44	1	0	-1.476639	4.776549	-1.834926
45	1	0	-0.207982	3.573508	-2.176209
46	7	0	2.508308	-0.796214	-0.538373
47	6	0	2.112493	-2.110361	-0.506630
48	6	0	2.585584	-3.003832	-1.625673
49	1	0	2.309437	-4.023643	-1.368977
50	1	0	2.096465	-2.727659	-2.562969
51	1	0	3.665775	-2.938110	-1.768698
52	8	0	-5.616060	-0.022228	-1.888696
53	8	0	-4.321751	-2.220628	0.009353
54	8	0	-1.780885	3.562526	0.410966
55	8	0	-1.456812	-2.524394	0.920347
56	8	0	1.521613	3.334308	-0.078881
57	8	0	1.403320	-2.521817	0.394154
58	6	0	4.859115	-0.236523	-1.178842
59	6	0	5.737929	0.575231	-1.899188
60	6	0	5.360216	-1.038209	-0.155484
61	6	0	7.095716	0.579606	-1.606837
62	1	0	5.352308	1.208836	-2.692082
63	6	0	6.721805	-1.030203	0.141120
64	1	0	4.693529	-1.669664	0.422635
65	6	0	7.591922	-0.224723	-0.583157
66	1	0	7.766904	1.215388	-2.172580
67	1	0	7.097955	-1.655114	0.942703
68	1	0	8.650111	-0.218125	-0.349979
69	6	0	3.387846	-0.231530	-1.555697
70	1	0	3.238283	-0.774023	-2.489266
71	1	0	3.067695	0.797147	-1.747031

Structure 37 B_1G^-E (M06-2X, Gas Phase)

Energy (Hartrees): = -1946.8497931
No imaginary frequencies

Standard orientation:

Center Number	Atomic Number	Atomic Type	Coordinates (Angstroms) X	Y	Z
1	6	0	-3.951981	1.539559	-0.150143
2	1	0	-3.686660	2.446793	0.395133
3	1	0	-3.931629	1.723440	-1.225077
4	6	0	-2.974485	0.425162	0.198257
5	1	0	-3.239476	0.016302	1.176916
6	6	0	-1.551138	0.949839	0.254773
7	1	0	-1.461552	1.610081	1.118411
8	6	0	-0.510527	-0.168923	0.383469
9	1	0	-0.634643	-0.893635	-0.423867
10	6	0	0.887147	0.425657	0.340174
11	1	0	0.989924	0.939960	-0.614918
12	6	0	2.103662	-0.529518	0.418054
13	1	0	2.927185	0.102188	0.778965
14	6	0	1.985563	-1.562425	1.456755
15	7	0	1.924404	-2.334133	2.304063
16	8	0	-5.271195	1.201572	0.272676
17	8	0	-3.029457	-0.615723	-0.774826
18	8	0	-1.246423	1.690754	-0.933547
19	8	0	-0.744656	-0.780875	1.649140
20	8	0	0.975437	1.378399	1.400671
21	6	0	-6.042624	0.510196	-0.595043
22	6	0	-3.832815	-1.673950	-0.507025
23	6	0	-1.219273	-2.054781	1.682132
24	6	0	-0.953603	3.008195	-0.794175
25	6	0	1.608834	2.541646	1.118833
26	6	0	-7.305565	0.052759	0.069670
27	6	0	-3.783573	-2.665425	-1.624710
28	6	0	-1.384615	-2.518663	3.094228
29	6	0	1.516874	3.502081	2.261188
30	1	0	-8.035796	-0.220665	-0.687577
31	1	0	-7.047291	-0.825515	0.666560
32	1	0	-7.693861	0.821001	0.736115
33	1	0	-1.952338	-3.444891	3.099997
34	1	0	-0.388189	-2.682794	3.511006
35	1	0	-1.883019	-1.750787	3.684790
36	1	0	-2.745310	-2.926492	-1.829134
37	1	0	-4.354461	-3.547596	-1.348542
38	1	0	-4.211961	-2.195972	-2.512419
39	1	0	2.284051	4.264636	2.154285
40	1	0	0.529959	3.968099	2.204111

41	1	0	1.603933	2.979815	3.212227
42	6	0	-0.442021	3.588033	-2.076706
43	1	0	-0.883969	3.090991	-2.937608
44	1	0	-0.635893	4.657727	-2.090096
45	1	0	0.640069	3.427706	-2.087869
46	7	0	2.522061	-1.002833	-0.893378
47	6	0	2.376650	-2.267746	-1.448125
48	6	0	1.439791	-3.258166	-0.801680
49	1	0	1.156996	-3.963104	-1.580299
50	1	0	1.964224	-3.802890	-0.014512
51	1	0	0.545482	-2.816600	-0.368795
52	8	0	-5.721290	0.276497	-1.726942
53	8	0	-4.494702	-1.755311	0.490961
54	8	0	-1.061346	3.599362	0.245113
55	8	0	-1.447018	-2.694046	0.693613
56	8	0	2.137288	2.750336	0.058199
57	8	0	2.987435	-2.559847	-2.453932
58	6	0	4.778623	0.019363	-0.936240
59	6	0	5.220414	1.201238	-0.345175
60	6	0	5.600742	-1.110275	-0.911580
61	6	0	6.472941	1.258082	0.262005
62	1	0	4.579699	2.077950	-0.363157
63	6	0	6.847149	-1.052225	-0.303971
64	1	0	5.255250	-2.024424	-1.383250
65	6	0	7.285897	0.132446	0.283946
66	1	0	6.810629	2.181536	0.717502
67	1	0	7.481950	-1.930371	-0.292673
68	1	0	8.260106	0.175524	0.756200
69	6	0	3.414462	-0.054069	-1.582113
70	1	0	3.490281	-0.391944	-2.613338
71	1	0	2.949283	0.935213	-1.563677

Structure 37 B_1G^-E (M06-2X, $CHCl_3$)

Energy (Hartrees): = -1946.8856788

No imaginary frequencies

Standard orientation:

Center Number	Atomic Number	Atomic Type	Coordinates (Angstroms) X	Y	Z
1	6	0	-3.984230	1.504757	-0.069280
2	1	0	-3.744715	2.381596	0.533939
3	1	0	-3.959500	1.762252	-1.128452
4	6	0	-2.985495	0.394996	0.224305
5	1	0	-3.225869	-0.056917	1.190009
6	6	0	-1.571903	0.949547	0.271002
7	1	0	-1.496781	1.638676	1.113441
8	6	0	-0.506128	-0.143225	0.428408
9	1	0	-0.612604	-0.889555	-0.361145
10	6	0	0.882418	0.471138	0.377281
11	1	0	0.977909	0.993094	-0.575479
12	6	0	2.107432	-0.477183	0.445196
13	1	0	2.932272	0.162820	0.785772
14	6	0	2.018588	-1.496906	1.499751
15	7	0	1.985413	-2.265909	2.350847
16	8	0	-5.304704	1.119782	0.318714
17	8	0	-3.026281	-0.607502	-0.793645
18	8	0	-1.307657	1.651603	-0.948452
19	8	0	-0.713371	-0.729063	1.713715
20	8	0	0.963958	1.409167	1.448238
21	6	0	-6.073551	0.500062	-0.597663
22	6	0	-3.814972	-1.684127	-0.585415
23	6	0	-1.233673	-1.979812	1.790788
24	6	0	-0.953346	2.959162	-0.871890
25	6	0	1.672725	2.540792	1.212193
26	6	0	-7.359111	0.034911	0.011396
27	6	0	-3.701901	-2.651422	-1.719337
28	6	0	-1.369201	-2.408040	3.215702
29	6	0	1.585512	3.483711	2.366117
30	1	0	-8.071171	-0.205512	-0.774509
31	1	0	-7.139793	-0.864115	0.593377
32	1	0	-7.762575	0.788839	0.686630
33	1	0	-1.911490	-3.349028	3.261176
34	1	0	-0.368470	-2.530016	3.637299
35	1	0	-1.886448	-1.636086	3.786785
36	1	0	-2.651741	-2.870423	-1.915284
37	1	0	-4.244264	-3.561782	-1.476577
38	1	0	-4.129621	-2.188999	-2.612007
39	1	0	2.316640	4.278938	2.243120
40	1	0	0.577711	3.905709	2.375164
41	1	0	1.742868	2.951419	3.304171
42	6	0	-0.629146	3.504576	-2.225733
43	1	0	-1.347596	3.147597	-2.963146

44	1	0	-0.615408	4.590992	-2.185443
45	1	0	0.362019	3.142388	-2.512599
46	7	0	2.512977	-0.972229	-0.863319
47	6	0	2.294709	-2.214487	-1.427281
48	6	0	1.389732	-3.205009	-0.740576
49	1	0	1.054994	-3.903333	-1.505516
50	1	0	1.958910	-3.761603	0.007434
51	1	0	0.521676	-2.767471	-0.254280
52	8	0	-5.738797	0.334182	-1.740722
53	8	0	-4.520913	-1.804230	0.380119
54	8	0	-0.896320	3.564215	0.164429
55	8	0	-1.525238	-2.627778	0.821994
56	8	0	2.264236	2.725255	0.181077
57	8	0	2.839679	-2.508170	-2.475940
58	6	0	4.795255	-0.011408	-0.977628
59	6	0	5.312290	1.167762	-0.443612
60	6	0	5.571184	-1.173919	-0.948080
61	6	0	6.592172	1.190678	0.106992
62	1	0	4.705945	2.068354	-0.454891
63	6	0	6.845564	-1.151357	-0.396486
64	1	0	5.170711	-2.091015	-1.368442
65	6	0	7.359700	0.032280	0.131174
66	1	0	6.985757	2.113064	0.518772
67	1	0	7.442309	-2.056221	-0.381320
68	1	0	8.355133	0.048104	0.559659
69	6	0	3.404807	-0.037987	-1.571474
70	1	0	3.432918	-0.353775	-2.611940
71	1	0	2.968229	0.962338	-1.519732

Structure 13 R-X (M06-2X, Gas Phase)

Energy (Hartrees): = -1946.8575287
No imaginary frequencies

Standard orientation:

Center Number	Atomic Number	Atomic Type	Coordinates (Angstroms) X	Y	Z
1	6	0	-5.897709	-0.550001	-1.915356
2	1	0	-5.775511	0.209141	-2.682491
3	6	0	-6.910733	-1.490477	-2.030121
4	1	0	-7.577531	-1.464894	-2.883713
5	6	0	-7.068260	-2.466467	-1.048717
6	1	0	-7.857197	-3.203472	-1.137196
7	6	0	-6.209412	-2.489882	0.041414
8	1	0	-6.324958	-3.246240	0.808501
9	6	0	-5.193481	-1.542973	0.158894
10	1	0	-4.530845	-1.570160	1.017460
11	6	0	-5.031475	-0.568466	-0.820936
12	6	0	-3.938291	0.481911	-0.746266
13	1	0	-3.287622	0.392408	-1.621813
14	1	0	-4.381196	1.478694	-0.806058
15	6	0	-4.591766	1.954651	1.643729
16	1	0	-4.756293	2.244899	2.677929
17	1	0	-5.468671	1.432370	1.257166
18	1	0	-4.419255	2.854100	1.046861
19	6	0	-3.352164	1.093873	1.610319
20	6	0	-1.880061	-0.380832	0.371622
21	1	0	-1.894779	-0.938065	-0.568436
22	6	0	-1.789151	-1.391154	1.439830
23	6	0	-0.657843	0.548684	0.328360
24	1	0	-0.702964	1.241871	1.166590
25	6	0	-1.198232	2.558415	-0.851922
26	6	0	-1.204865	3.170435	-2.219798
27	1	0	-1.807626	4.075027	-2.206877
28	1	0	-1.571179	2.460336	-2.960125
29	1	0	-0.170427	3.424699	-2.462615
30	6	0	0.674691	-0.181096	0.352592
31	1	0	0.854831	-0.557703	1.361737
32	6	0	1.111801	-2.479122	-0.125751
33	6	0	1.269100	-3.444547	-1.259577
34	1	0	2.273950	-3.297464	-1.666309
35	1	0	0.540242	-3.258971	-2.045667
36	1	0	1.192600	-4.458854	-0.875908
37	6	0	1.802700	0.760314	-0.072523
38	1	0	1.600322	1.135930	-1.076491
39	6	0	1.648594	3.103351	0.365203
40	6	0	1.432564	4.093838	1.467486
41	1	0	1.692917	5.088399	1.114415
42	1	0	2.005301	3.821450	2.352035
43	1	0	0.368539	4.067435	1.718003
44	6	0	3.159602	0.088761	-0.088799

45	1	0	3.142427	-0.693620	-0.849654
46	6	0	4.004383	-1.708972	1.210650
47	6	0	4.103276	-2.228436	2.611150
48	1	0	4.759651	-3.094335	2.629167
49	1	0	3.097783	-2.519114	2.921466
50	1	0	4.462504	-1.448302	3.281068
51	6	0	4.270183	1.072325	-0.418566
52	1	0	4.050960	1.582121	-1.359078
53	1	0	4.380469	1.795274	0.388752
54	6	0	5.703000	-0.372017	-1.596626
55	6	0	6.948107	-1.197403	-1.479778
56	1	0	7.260568	-1.523047	-2.468673
57	1	0	6.694218	-2.068394	-0.870946
58	1	0	7.737981	-0.639876	-0.979253
59	8	0	-2.593787	1.002477	2.549881
60	8	0	-1.490162	3.115778	0.169787
61	8	0	-0.780058	1.269647	-0.905596
62	8	0	0.649298	-1.272524	-0.569996
63	8	0	1.390769	-2.692450	1.016726
64	8	0	1.672756	3.353614	-0.809414
65	8	0	1.778877	1.849117	0.858257
66	8	0	3.410089	-0.493918	1.191252
67	8	0	4.367383	-2.282196	0.218887
68	8	0	5.519205	0.390129	-0.493409
69	8	0	4.935133	-0.380376	-2.517852
70	7	0	-3.100373	0.406951	0.437618
71	7	0	-1.712852	-2.236137	2.212798

Structure 42 $_1G^+{}_5G^-$ (M06-2X, Gas Phase)

Energy (Hartrees): = -1487.7521796
No imaginary frequencies

Standard orientation:

Center Number	Atomic Number	Atomic Type	Coordinates (Angstroms) X	Y	Z
1	6	0	-2.078094	-0.503828	-2.264113
2	1	0	-2.007488	-0.611160	-3.346868
3	6	0	-0.745564	-0.856956	-1.635196
4	1	0	-0.564894	-1.928737	-1.737530
5	6	0	-0.686661	-0.442052	-0.163971
6	1	0	-0.743963	0.647032	-0.117640
7	6	0	0.607817	-0.887880	0.491912
8	1	0	1.432055	-0.444389	-0.070247
9	6	0	0.802373	-0.401091	1.947092
10	1	0	1.801172	-0.728802	2.250706
11	6	0	-0.132725	-1.056724	2.874874
12	7	0	-0.858263	-1.574799	3.597473
13	8	0	-2.396647	0.861557	-1.985699
14	8	0	0.284414	-0.124302	-2.309139
15	8	0	-1.825235	-1.017168	0.475677
16	8	0	0.696981	-2.302484	0.408845
17	6	0	-3.533271	1.120132	-1.300546
18	6	0	-2.556554	-0.226290	1.294366
19	6	0	1.444483	-0.762280	-2.586671
20	6	0	1.956204	-2.804809	0.341646
21	6	0	-3.653497	2.582266	-0.991158
22	6	0	-3.712905	-0.969111	1.874839
23	6	0	1.938303	-4.250754	-0.039594
24	1	0	-4.370822	-0.265300	2.376935
25	1	0	-4.241144	-1.495856	1.081751
26	1	0	-3.317596	-1.692401	2.592099
27	1	0	-3.208000	3.187527	-1.777827
28	1	0	-4.701380	2.831230	-0.842224
29	1	0	-3.112479	2.756655	-0.057416
30	1	0	2.901434	-4.699513	0.187831
31	1	0	1.128462	-4.770774	0.470044
32	1	0	1.756879	-4.291032	-1.116111
33	6	0	2.458360	0.203198	-3.123968
34	1	0	2.747771	0.878938	-2.313927
35	1	0	3.326441	-0.344749	-3.479797
36	1	0	2.019139	0.805892	-3.918333
37	7	0	0.704747	1.036094	2.081322
38	8	0	-4.310542	0.271817	-0.958654
39	8	0	1.621357	-1.932421	-2.386855
40	8	0	-2.263272	0.921802	1.518224
41	8	0	2.928890	-2.133338	0.547133
42	1	0	-2.858842	-1.152504	-1.870569
43	1	0	-0.254753	1.365661	2.070332
44	6	0	1.595774	1.849466	1.370730
45	6	0	1.218362	3.168209	1.084573
46	6	0	2.860349	1.406089	0.962138
47	6	0	2.082893	4.019732	0.415682
48	1	0	0.240117	3.516176	1.399589

49	6	0	3.718047	2.274185	0.293784
50	1	0	3.184444	0.387857	1.147864
51	6	0	3.341625	3.581534	0.012862
52	1	0	1.768760	5.035480	0.205888
53	1	0	4.693923	1.912762	-0.009840
54	1	0	4.015888	4.249024	-0.508232

Structure 42 $_1G^+{}_5G^-$ (M06-2X, $CHCl_3$)

Energy (Hartrees): = -1487.7794703
No imaginary frequencies

Standard orientation:

Center Number	Atomic Number	Atomic Type	Coordinates (Angstroms) X	Y	Z
1	6	0	2.335857	-0.883629	1.962560
2	1	0	2.374575	-1.282909	2.976298
3	6	0	0.988855	-1.200442	1.345623
4	1	0	0.921046	-2.274112	1.162583
5	6	0	0.758745	-0.419125	0.049690
6	1	0	0.746247	0.646623	0.285342
7	6	0	-0.562004	-0.781988	-0.604346
8	1	0	-1.358495	-0.560655	0.109587
9	6	0	-0.906443	0.023889	-1.879524
10	1	0	-1.899329	-0.309401	-2.198884
11	6	0	-0.011926	-0.312984	-3.000528
12	7	0	0.673198	-0.572608	-3.883761
13	8	0	2.503200	0.536401	2.050745
14	8	0	-0.035078	-0.798458	2.264419
15	8	0	1.863876	-0.721131	-0.804653
16	8	0	-0.556416	-2.173737	-0.894843
17	6	0	3.533780	1.102201	1.388363
18	6	0	2.490667	0.302576	-1.426977
19	6	0	-1.064347	-1.646846	2.489272
20	6	0	-1.759860	-2.797318	-0.846971
21	6	0	3.496812	2.593056	1.515392
22	6	0	3.652810	-0.179240	-2.227804
23	6	0	-1.605303	-4.277863	-0.962154
24	1	0	4.249887	0.672534	-2.544091
25	1	0	4.249587	-0.872183	-1.636224
26	1	0	3.265423	-0.706333	-3.103673
27	1	0	3.334326	2.881142	2.553983
28	1	0	4.425186	3.013314	1.136186
29	1	0	2.658100	2.965595	0.922099
30	1	0	-2.578440	-4.735681	-1.122037
31	1	0	-0.920936	-4.524507	-1.774068
32	1	0	-1.174458	-4.641940	-0.026398
33	6	0	-2.067898	-1.031005	3.412553
34	1	0	-2.591375	-0.236528	2.873667
35	1	0	-2.780667	-1.786509	3.733423
36	1	0	-1.566061	-0.579623	4.268365
37	7	0	-0.920455	1.453646	-1.684476
38	8	0	4.348073	0.471953	0.766642
39	8	0	-1.146339	-2.733041	1.980920
40	8	0	2.108804	1.443391	-1.342402
41	8	0	-2.790783	-2.193737	-0.717851
42	1	0	3.134929	-1.316913	1.363799
43	1	0	0.004039	1.864496	-1.605397
44	6	0	-1.892369	2.043698	-0.869792
45	6	0	-1.679698	3.357772	-0.428171
46	6	0	-3.080247	1.394367	-0.510093
47	6	0	-2.633119	4.005122	0.341805
48	1	0	-0.759892	3.863183	-0.704517
49	6	0	-4.029982	2.060062	0.260613
50	1	0	-3.275993	0.371345	-0.811470
51	6	0	-3.820018	3.363535	0.692039
52	1	0	-2.446915	5.021697	0.669487
53	1	0	-4.943833	1.539894	0.524813
54	1	0	-4.564719	3.872048	1.291660

Structure 42 $_1G^-{}_5G^-$ (M06-2X, Gas Phase)

Energy (Hartrees): = -1487.7463848
No imaginary frequencies

Standard orientation:

Center Number	Atomic Number	Atomic Type	Coordinates (Angstroms) X	Y	Z
1	6	0	-3.126738	1.104582	-1.198370
2	1	0	-3.519177	2.080130	-1.484179

3	6	0	-1.681884	1.250062	-0.752701
4	1	0	-1.051361	1.416796	-1.627373
5	6	0	-1.190535	0.012775	-0.000419
6	1	0	-1.873138	-0.229699	0.817207
7	6	0	0.194548	0.227404	0.583565
8	1	0	0.141188	1.039850	1.310873
9	6	0	0.765881	-1.021802	1.303749
10	1	0	0.619759	-1.907884	0.686008
11	6	0	-0.006097	-1.221252	2.542253
12	7	0	-0.613167	-1.298466	3.512868
13	8	0	-3.930072	0.619730	-0.120246
14	8	0	-1.557023	2.363384	0.137511
15	8	0	-1.189812	-1.040092	-0.967730
16	8	0	1.067014	0.593292	-0.473925
17	6	0	-4.415185	-0.638988	-0.221711
18	6	0	-1.681728	-2.246511	-0.584652
19	6	0	-0.670443	3.337027	-0.193614
20	6	0	1.993450	1.551458	-0.211563
21	6	0	-5.019269	-1.093117	1.071449
22	6	0	-1.811968	-3.171467	-1.753984
23	6	0	2.845361	1.800599	-1.412870
24	1	0	-1.836849	-4.198770	-1.399354
25	1	0	-2.761520	-2.931904	-2.238172
26	1	0	-1.003946	-3.014688	-2.466599
27	1	0	-5.447427	-0.257800	1.621256
28	1	0	-5.760784	-1.863127	0.872905
29	1	0	-4.209495	-1.531460	1.663142
30	1	0	3.650061	2.480324	-1.146483
31	1	0	3.244660	0.850616	-1.774643
32	1	0	2.215906	2.242408	-2.187750
33	6	0	-0.510710	4.318970	0.926575
34	1	0	0.246066	3.911737	1.602781
35	1	0	-0.150723	5.263988	0.527362
36	1	0	-1.442786	4.444825	1.473591
37	7	0	2.171041	-0.896033	1.629618
38	8	0	-4.317428	-1.306250	-1.215901
39	8	0	-0.075746	3.363486	-1.234541
40	8	0	-1.974500	-2.494545	0.551479
41	8	0	2.076731	2.097930	0.855469
42	1	0	-3.187598	0.414351	-2.038537
43	1	0	2.353967	-0.091835	2.217768
44	6	0	3.158969	-1.081728	0.651664
45	6	0	4.384443	-0.421933	0.811778
46	6	0	2.986926	-1.911898	-0.460654
47	6	0	5.408304	-0.595676	-0.108167
48	1	0	4.521664	0.237602	1.662242
49	6	0	4.018746	-2.075358	-1.376707
50	1	0	2.050428	-2.425829	-0.632676
51	6	0	5.234983	-1.421782	-1.213700
52	1	0	6.346400	-0.073862	0.040100
53	1	0	3.862035	-2.723062	-2.231347
54	1	0	6.031921	-1.554881	-1.933970

Structure 42 $_{1}G^{-}{}_{5}G^{-}$ (M06-2X, $CHCl_3$)

Energy (Hartrees): = -1487.7756752
No imaginary frequencies

Standard orientation:

Center Number	Atomic Number	Atomic Type	Coordinates (Angstroms) X	Y	Z
1	6	0	-3.121811	1.121298	-1.169599
2	1	0	-3.481892	2.105999	-1.467252
3	6	0	-1.680256	1.226054	-0.710548
4	1	0	-1.049934	1.417725	-1.579665
5	6	0	-1.199908	-0.041362	-0.002214
6	1	0	-1.879819	-0.306647	0.811160
7	6	0	0.193367	0.147356	0.577923
8	1	0	0.145852	0.917329	1.350197
9	6	0	0.803401	-1.133228	1.200071
10	1	0	0.712664	-1.964519	0.499280
11	6	0	0.037222	-1.488683	2.407625
12	7	0	-0.544484	-1.709292	3.371585
13	8	0	-3.955137	0.672275	-0.095304
14	8	0	-1.546391	2.304169	0.222749
15	8	0	-1.214443	-1.057104	-1.007222
16	8	0	1.052542	0.572007	-0.472638
17	6	0	-4.465570	-0.574652	-0.174200
18	6	0	-1.700304	-2.278570	-0.669604
19	6	0	-0.731384	3.334518	-0.109209
20	6	0	1.940948	1.562453	-0.202146
21	6	0	-5.165577	-0.957237	1.091672
22	6	0	-1.830729	-3.152161	-1.874557

23	6	0	2.791040	1.847758	-1.395768
24	1	0	-2.120081	-4.153958	-1.567371
25	1	0	-2.596264	-2.724054	-2.524227
26	1	0	-0.888651	-3.174610	-2.424327
27	1	0	-5.657419	-0.098185	1.544947
28	1	0	-5.875429	-1.755640	0.887629
29	1	0	-4.408190	-1.330646	1.787602
30	1	0	3.577552	2.547379	-1.123787
31	1	0	3.216699	0.915126	-1.772441
32	1	0	2.157844	2.278980	-2.174508
33	6	0	-0.614208	4.311027	1.017433
34	1	0	0.099351	3.902235	1.738065
35	1	0	-0.234427	5.257442	0.639158
36	1	0	-1.573116	4.443646	1.516751
37	7	0	2.192874	-0.967416	1.575453
38	8	0	-4.329920	-1.285894	-1.135361
39	8	0	-0.166545	3.412939	-1.166886
40	8	0	-1.969556	-2.576782	0.461679
41	8	0	1.996225	2.111333	0.865990
42	1	0	-3.194661	0.432748	-2.009892
43	1	0	2.324604	-0.204114	2.229965
44	6	0	3.226266	-1.057788	0.631348
45	6	0	4.400827	-0.329917	0.867136
46	6	0	3.151827	-1.861886	-0.511894
47	6	0	5.471114	-0.408747	-0.012629
48	1	0	4.460194	0.304677	1.745872
49	6	0	4.229452	-1.927923	-1.388174
50	1	0	2.260862	-2.436174	-0.732565
51	6	0	5.395002	-1.206052	-1.151292
52	1	0	6.367898	0.164517	0.193928
53	1	0	4.151280	-2.555587	-2.268876
54	1	0	6.227838	-1.263805	-1.841195

Structure 42 $_5G^-$ (M06-2X, Gas Phase)

Energy (Hartrees): = -1487.746178
No imaginary frequencies

Standard orientation:

Center Number	Atomic Number	Atomic Type	Coordinates (Angstroms) X	Y	Z
1	6	0	1.795103	2.299104	-0.976062
2	1	0	2.765019	2.579575	-1.389646
3	6	0	1.810077	0.827361	-0.610889
4	1	0	1.855445	0.237210	-1.526975
5	6	0	0.596221	0.425308	0.218600
6	1	0	0.587794	1.001253	1.145326
7	6	0	0.636874	-1.052409	0.604326
8	1	0	1.380899	-1.169048	1.392696
9	6	0	-0.731103	-1.567777	1.101160
10	1	0	-1.177578	-0.764212	1.694481
11	6	0	-0.520407	-2.724893	2.002750
12	7	0	-0.344694	-3.627939	2.689285
13	8	0	1.576276	3.078960	0.201224
14	8	0	2.946946	0.536169	0.208693
15	8	0	-0.558432	0.765008	-0.549500
16	8	0	1.031620	-1.856491	-0.510674
17	6	0	0.504901	3.903805	0.221095
18	6	0	-1.525482	1.498010	0.072965
19	6	0	4.001828	-0.082063	-0.374347
20	6	0	2.180594	-2.587575	-0.396565
21	6	0	0.336374	4.536765	1.569214
22	6	0	-2.583119	1.935785	-0.886375
23	6	0	2.486033	-3.299907	-1.676946
24	1	0	-3.442913	2.289301	-0.323436
25	1	0	-2.165754	2.751281	-1.481052
26	1	0	-2.865958	1.113894	-1.542968
27	1	0	1.297968	4.681484	2.057110
28	1	0	-0.198507	5.476993	1.459125
29	1	0	-0.269635	3.852719	2.168996
30	1	0	3.223056	-4.075275	-1.485568
31	1	0	1.580903	-3.719271	-2.113286
32	1	0	2.905325	-2.563404	-2.366666
33	6	0	5.094575	-0.328357	0.619670
34	1	0	4.801389	-1.193338	1.218829
35	1	0	6.018311	-0.547538	0.090531
36	1	0	5.210759	0.529718	1.279686
37	7	0	-1.598165	-1.926592	0.010095
38	8	0	-0.224663	4.064782	-0.718327
39	8	0	4.014349	-0.401721	-1.531631
40	8	0	-1.477167	1.759553	1.245181
41	8	0	2.847525	-2.598372	0.596122
42	1	0	1.011229	2.499536	-1.705037
43	1	0	-1.107549	-2.202446	-0.826691

44	6	0	-2.879194	-1.425166	-0.168697
45	6	0	-3.470277	-1.534870	-1.437193
46	6	0	-3.615430	-0.833704	0.863711
47	6	0	-4.743753	-1.041906	-1.668473
48	1	0	-2.914038	-2.007153	-2.240209
49	6	0	-4.888861	-0.333665	0.613787
50	1	0	-3.213092	-0.767589	1.866632
51	6	0	-5.462635	-0.424048	-0.647609
52	1	0	-5.176221	-1.137450	-2.657752
53	1	0	-5.436765	0.127752	1.427115
54	1	0	-6.455620	-0.034161	-0.830303

Structure 42 $_5G^-$ (M06-2X, $CHCl_3$)

Energy (Hartrees): = -1487.7750899
No imaginary frequencies

Standard orientation:

Center Number	Atomic Number	Atomic Type	Coordinates (Angstroms) X	Y	Z
1	6	0	1.859171	2.291857	-0.980605
2	1	0	2.834605	2.550213	-1.395746
3	6	0	1.832838	0.819379	-0.624729
4	1	0	1.860786	0.237653	-1.546483
5	6	0	0.607220	0.441750	0.201839
6	1	0	0.595061	1.022812	1.125055
7	6	0	0.626617	-1.032362	0.599222
8	1	0	1.366182	-1.145327	1.391780
9	6	0	-0.745904	-1.527538	1.102798
10	1	0	-1.174269	-0.730028	1.716784
11	6	0	-0.543787	-2.699647	1.989322
12	7	0	-0.384355	-3.620221	2.656417
13	8	0	1.665595	3.074178	0.202648
14	8	0	2.964846	0.491095	0.190684
15	8	0	-0.536028	0.786034	-0.583347
16	8	0	1.008568	-1.846680	-0.511651
17	6	0	0.623321	3.930062	0.237276
18	6	0	-1.497181	1.546763	0.006049
19	6	0	3.982315	-0.195579	-0.380362
20	6	0	2.106276	-2.646697	-0.380034
21	6	0	0.499818	4.581224	1.578965
22	6	0	-2.538322	1.967568	-0.977467
23	6	0	2.357941	-3.408482	-1.640232
24	1	0	-3.417606	2.317683	-0.441688
25	1	0	-2.118679	2.786455	-1.567418
26	1	0	-2.792396	1.145428	-1.645607
27	1	0	1.475960	4.720766	2.040241
28	1	0	-0.023462	5.529301	1.474436
29	1	0	-0.096607	3.917300	2.210878
30	1	0	3.279031	-3.976662	-1.538348
31	1	0	1.519122	-4.081472	-1.829330
32	1	0	2.434409	-2.709802	-2.474271
33	6	0	5.083199	-0.433835	0.602850
34	1	0	4.687542	-0.997928	1.448856
35	1	0	5.884800	-0.988884	0.122003
36	1	0	5.452986	0.523473	0.974406
37	7	0	-1.627938	-1.873345	0.021108
38	8	0	-0.117669	4.110391	-0.693049
39	8	0	3.966266	-0.556120	-1.527033
40	8	0	-1.460668	1.845958	1.170788
41	8	0	2.755099	-2.695593	0.626494
42	1	0	1.082590	2.515576	-1.710317
43	1	0	-1.157628	-2.176904	-0.819394
44	6	0	-2.912450	-1.375033	-0.140296
45	6	0	-3.523948	-1.503235	-1.398481
46	6	0	-3.630483	-0.767243	0.896574
47	6	0	-4.802187	-1.013894	-1.614354
48	1	0	-2.978407	-1.986033	-2.203248
49	6	0	-4.910249	-0.273377	0.661854
50	1	0	-3.208266	-0.684218	1.890349
51	6	0	-5.505870	-0.383474	-0.588845
52	1	0	-5.250929	-1.121814	-2.595609
53	1	0	-5.444100	0.200681	1.477928
54	1	0	-6.502752	0.003337	-0.760276

Structure 42 R-X (M06-2X, Gas Phase)

Energy (Hartrees): = -1487.747819
No imaginary frequencies

Standard orientation:

Center	Atomic	Atomic	Coordinates (Angstroms)

Number	Number	Type	X	Y	Z
1	8	0	-0.603280	-1.801717	0.677977
2	8	0	-1.950736	-3.147480	-0.524408
3	8	0	-0.079492	1.122153	0.825614
4	8	0	1.132487	2.049936	-0.822436
5	8	0	-3.139180	-0.162866	-0.514603
6	8	0	-4.087030	-1.414003	1.094911
7	8	0	-2.504322	2.728514	-0.384231
8	8	0	-1.081341	3.991720	0.822356
9	7	0	1.578677	-3.389195	-1.880599
10	7	0	1.922029	-0.746975	0.289113
11	6	0	1.360834	-2.347340	-1.449759
12	6	0	1.078719	-1.020029	-0.847957
13	1	0	1.243766	-0.252663	-1.609807
14	6	0	3.304604	-0.579612	0.146213
15	6	0	4.100448	-0.585763	1.299306
16	1	0	3.634739	-0.764535	2.263283
17	6	0	5.464756	-0.363219	1.212894
18	1	0	6.062759	-0.374684	2.116609
19	6	0	6.066572	-0.127029	-0.020535
20	1	0	7.133002	0.045707	-0.086862
21	6	0	5.279374	-0.123494	-1.163590
22	1	0	5.731763	0.050101	-2.132801
23	6	0	3.908711	-0.349638	-1.091657
24	1	0	3.326005	-0.350624	-2.003647
25	6	0	-0.405366	-0.951978	-0.454163
26	1	0	-1.005725	-1.326448	-1.284519
27	6	0	-1.435603	-2.873455	0.520023
28	6	0	-1.645337	-3.587834	1.817819
29	1	0	-2.450934	-3.065741	2.340870
30	1	0	-0.747196	-3.564560	2.431625
31	1	0	-1.957545	-4.609026	1.614368
32	6	0	-0.888981	0.462264	-0.138949
33	1	0	-0.882535	1.027504	-1.073264
34	6	0	0.950654	1.866946	0.351494
35	6	0	1.759660	2.439233	1.469060
36	1	0	1.915474	1.684623	2.239116
37	1	0	1.191431	3.270211	1.894180
38	1	0	2.709234	2.795188	1.077186
39	6	0	-2.293047	0.469521	0.449673
40	1	0	-2.313476	-0.101228	1.378873
41	6	0	-3.997479	-1.110728	-0.062754
42	6	0	-4.759192	-1.729683	-1.193547
43	1	0	-4.122592	-2.510678	-1.616001
44	1	0	-4.973286	-0.992952	-1.965480
45	1	0	-5.671484	-2.178595	-0.808760
46	6	0	-2.769388	1.884133	0.738526
47	1	0	-3.847651	1.878428	0.899402
48	1	0	-2.260280	2.276185	1.617483
49	6	0	-1.593545	3.717408	-0.227452
50	6	0	-1.285020	4.375075	-1.539448
51	1	0	-2.153048	4.369257	-2.195348
52	1	0	-0.481176	3.798669	-2.005696
53	1	0	-0.929945	5.386814	-1.358883
54	1	0	1.638661	-1.256492	1.115862

Structure 42 $_1G^+{}_5G^-$ (M06-2X, Gas Phase)

Energy (Hartrees): =-1409.3879334
No imaginary frequencies

Standard orientation:

Center	Atomic	Atomic	Coordinates (Angstroms)		
Number	Number	Type	X	Y	Z
1	6	0	0.456049	2.860554	-0.038276
2	1	0	-0.085360	3.757643	-0.339001
3	6	0	-0.515368	1.698066	0.063104
4	1	0	-1.121419	1.833585	0.959604
5	6	0	0.196828	0.350115	0.125995
6	1	0	0.824409	0.219836	-0.757003
7	6	0	-0.803807	-0.798057	0.180838
8	1	0	-1.338064	-0.823601	-0.769859
9	6	0	-0.188075	-2.196058	0.370666
10	1	0	-1.003735	-2.919123	0.271540
11	6	0	0.405712	-2.372792	1.709321
12	7	0	0.888433	-2.518267	2.740660
13	8	0	1.443876	2.597730	-1.038616
14	8	0	-1.367124	1.650052	-1.084485
15	8	0	1.012013	0.411682	1.297566
16	8	0	-1.723616	-0.560057	1.241740
17	6	0	2.725977	2.463411	-0.630524
18	6	0	2.279112	-0.048701	1.224596

19	6	0	-2.662233	2.020858	-0.914341
20	6	0	-3.046999	-0.518714	0.927207
21	6	0	3.614212	2.043406	-1.763238
22	6	0	3.004422	0.158314	2.511499
23	6	0	-3.866746	-0.116995	2.112821
24	1	0	4.023322	-0.203890	2.409128
25	1	0	2.997621	1.222486	2.747515
26	1	0	2.478658	-0.390087	3.295243
27	1	0	3.233569	2.399506	-2.717817
28	1	0	4.623864	2.402439	-1.578210
29	1	0	3.628661	0.950214	-1.766013
30	1	0	-4.884784	-0.475430	1.982893
31	1	0	-3.426884	-0.488297	3.036043
32	1	0	-3.873834	0.976109	2.134294
33	6	0	-3.451574	1.804912	-2.167908
34	1	0	-3.731209	0.748785	-2.190321
35	1	0	-4.351391	2.413778	-2.134740
36	1	0	-2.851594	2.030097	-3.047602
37	7	0	0.763703	-2.507298	-0.667778
38	6	0	0.459642	-3.435821	-1.632326
39	6	0	1.577598	-3.722608	-2.607530
40	1	0	2.084402	-4.635917	-2.289704
41	1	0	1.142023	-3.899301	-3.588911
42	1	0	2.307966	-2.915232	-2.659910
43	8	0	3.083216	2.620563	0.504720
44	8	0	-3.092627	2.432682	0.126997
45	8	0	2.725719	-0.566179	0.229470
46	8	0	-3.461867	-0.738011	-0.175521
47	8	0	-0.615379	-3.988847	-1.681053
48	1	0	0.943071	3.023451	0.921625
49	1	0	1.673552	-2.064370	-0.626934

Structure 42 $_1G^+{}_5G^-$ (M06-2X, $CHCl_3$)

Energy (Hartrees): = -1409.4123463
No imaginary frequencies

Standard orientation:

Center Number	Atomic Number	Atomic Type	Coordinates (Angstroms) X	Y	Z
1	6	0	0.899162	2.804278	0.033026
2	1	0	0.499014	3.784077	-0.227755
3	6	0	-0.234875	1.800346	0.105918
4	1	0	-0.830702	2.019477	0.992473
5	6	0	0.262825	0.356511	0.166049
6	1	0	0.904539	0.146218	-0.691774
7	6	0	-0.900552	-0.625893	0.144429
8	1	0	-1.395725	-0.537003	-0.823014
9	6	0	-0.518858	-2.111012	0.294249
10	1	0	-1.434472	-2.692199	0.147537
11	6	0	-0.019495	-2.422677	1.647417
12	7	0	0.383892	-2.674924	2.692074
13	8	0	1.828497	2.438143	-0.993077
14	8	0	-1.057340	1.900579	-1.062011
15	8	0	1.017954	0.271728	1.376908
16	8	0	-1.805263	-0.282826	1.188946
17	6	0	3.069344	2.061193	-0.617356
18	6	0	2.201923	-0.377570	1.355565
19	6	0	-2.332978	2.332226	-0.903618
20	6	0	-3.124694	-0.181093	0.871509
21	6	0	3.882162	1.641777	-1.802446
22	6	0	2.903492	-0.266129	2.666217
23	6	0	-3.922811	0.251074	2.057350
24	1	0	3.861998	-0.775220	2.608168
25	1	0	3.043916	0.788598	2.905384
26	1	0	2.279397	-0.719386	3.439445
27	1	0	3.848789	2.415667	-2.570109
28	1	0	4.906376	1.451740	-1.491892
29	1	0	3.449835	0.731313	-2.223479
30	1	0	-4.982455	0.196521	1.821042
31	1	0	-3.683954	-0.374195	2.918102
32	1	0	-3.648568	1.281511	2.293786
33	6	0	-3.080205	2.277528	-2.197146
34	1	0	-3.291934	1.228345	-2.418322
35	1	0	-4.016210	2.821754	-2.099236
36	1	0	-2.474122	2.684457	-3.006275
37	7	0	0.415909	-2.537274	-0.716158
38	6	0	0.042802	-3.410375	-1.701022
39	6	0	1.129331	-3.760752	-2.684585
40	1	0	1.118807	-4.839188	-2.841991
41	1	0	0.896859	-3.280363	-3.637447
42	1	0	2.119235	-3.444992	-2.355621
43	8	0	3.444025	2.056844	0.525563
44	8	0	-2.780479	2.680606	0.155642

45	8	0	2.597801	-0.974764	0.384719
46	8	0	-3.543556	-0.400362	-0.231461
47	8	0	-1.087893	-3.848219	-1.786352
48	1	0	1.408668	2.858737	0.993387
49	1	0	1.372696	-2.210910	-0.648168

Structure 42 $_1G^-{}_5G^-$ (M06-2X, Gas Phase)

Energy (Hartrees): =-1409.3787268
No imaginary frequencies

Standard orientation:

Center Number	Atomic Number	Atomic Type	Coordinates (Angstroms) X	Y	Z
1	6	0	-2.579844	1.285724	0.411472
2	1	0	-2.849183	0.946455	1.413856
3	6	0	-1.095053	1.115904	0.145497
4	1	0	-0.868703	1.339481	-0.896815
5	6	0	-0.538055	-0.265953	0.504350
6	1	0	-0.867043	-0.559666	1.505159
7	6	0	0.984840	-0.255421	0.462640
8	1	0	1.361855	0.299487	1.323420
9	6	0	1.612892	-1.663282	0.432858
10	1	0	1.468599	-2.065488	-0.572831
11	6	0	0.960354	-2.559533	1.398699
12	7	0	0.435385	-3.234436	2.164592
13	8	0	-3.279657	0.513546	-0.562389
14	8	0	-0.418642	2.043150	1.004971
15	8	0	-0.986741	-1.236990	-0.439147
16	8	0	1.396883	0.391075	-0.737892
17	6	0	-4.604797	0.379387	-0.326417
18	6	0	-2.080020	-1.971831	-0.108408
19	6	0	0.260867	3.070786	0.434659
20	6	0	2.475381	1.206343	-0.694989
21	6	0	-5.221541	-0.626184	-1.255111
22	6	0	-2.395179	-2.944921	-1.205098
23	6	0	2.819679	1.691459	-2.065548
24	1	0	-3.215273	-3.587529	-0.895381
25	1	0	-2.666430	-2.383015	-2.101057
26	1	0	-1.511842	-3.540215	-1.438017
27	1	0	-5.010280	-1.619307	-0.850143
28	1	0	-6.297425	-0.475366	-1.287056
29	1	0	-4.786798	-0.554101	-2.251229
30	1	0	3.634202	2.407841	-2.001116
31	1	0	3.111129	0.824826	-2.662860
32	1	0	1.936652	2.150232	-2.510483
33	6	0	0.989626	3.867786	1.475132
34	1	0	1.939893	3.361980	1.664991
35	1	0	1.188261	4.863917	1.087557
36	1	0	0.421167	3.909442	2.402104
37	7	0	3.039580	-1.616223	0.674643
38	6	0	3.921234	-1.465218	-0.372499
39	6	0	5.327585	-1.114580	0.048040
40	1	0	6.004147	-1.335532	-0.773582
41	1	0	5.357421	-0.043084	0.264381
42	1	0	5.630537	-1.661039	0.941976
43	8	0	-5.172897	0.978647	0.539072
44	8	0	0.288847	3.269665	-0.746353
45	8	0	-2.688411	-1.829985	0.911223
46	8	0	3.055373	1.467580	0.326942
47	8	0	3.577050	-1.579626	-1.527458
48	1	0	-2.848703	2.340219	0.312426
49	1	0	3.340810	-1.304218	1.586648

Structure 42 $_1G^-{}_5G^-$ (M06-2X, $CHCl_3$)

Energy (Hartrees): = -1409.4072708
No imaginary frequencies

Standard orientation:

Center Number	Atomic Number	Atomic Type	Coordinates (Angstroms) X	Y	Z
1	6	0	2.598209	1.262887	-0.348326
2	1	0	2.882553	0.933233	-1.349365
3	6	0	1.102346	1.127215	-0.123930
4	1	0	0.857302	1.330052	0.918517
5	6	0	0.526304	-0.229511	-0.536060
6	1	0	0.852176	-0.480913	-1.548667
7	6	0	-0.996778	-0.207117	-0.505646
8	1	0	-1.361334	0.354338	-1.366189

9	6	0	-1.630704	-1.611041	-0.489378
10	1	0	-1.427643	-2.062502	0.485029
11	6	0	-1.034110	-2.473418	-1.520477
12	7	0	-0.534062	-3.122341	-2.324364
13	8	0	3.248259	0.456902	0.636414
14	8	0	0.470271	2.090139	-0.976554
15	8	0	0.966321	-1.242443	0.368561
16	8	0	-1.438367	0.417156	0.696866
17	6	0	4.567367	0.262093	0.445847
18	6	0	2.025804	-2.003893	-0.002643
19	6	0	-0.181228	3.133493	-0.406163
20	6	0	-2.448980	1.316394	0.622903
21	6	0	5.129363	-0.707235	1.440690
22	6	0	2.286577	-3.064137	1.020414
23	6	0	-2.867069	1.748342	1.990234
24	1	0	3.163270	-3.642009	0.738044
25	1	0	2.425256	-2.603006	1.999798
26	1	0	1.414177	-3.719213	1.079468
27	1	0	4.938167	-1.718937	1.072497
28	1	0	6.204281	-0.560298	1.520828
29	1	0	4.649276	-0.593506	2.411803
30	1	0	-3.628814	2.520430	1.913540
31	1	0	-3.253604	0.879006	2.527670
32	1	0	-1.996187	2.122197	2.530099
33	6	0	-0.803530	4.001774	-1.453248
34	1	0	-1.522761	3.410128	-2.022283
35	1	0	-1.304437	4.840907	-0.976905
36	1	0	-0.033693	4.356809	-2.140347
37	7	0	-3.063622	-1.528569	-0.635479
38	6	0	-3.885067	-1.572858	0.463385
39	6	0	-5.312908	-1.178508	0.187331
40	1	0	-5.952853	-1.587356	0.966360
41	1	0	-5.379304	-0.086599	0.209286
42	1	0	-5.646466	-1.522840	-0.792257
43	8	0	5.181022	0.799352	-0.435797
44	8	0	-0.240134	3.301586	0.781002
45	8	0	2.632694	-1.831281	-1.022609
46	8	0	-2.920854	1.684764	-0.420398
47	8	0	-3.475851	-1.878095	1.565564
48	1	0	2.891494	2.307912	-0.219347
49	1	0	-3.427538	-1.132997	-1.492668

Structure 42 $_5G^-$ (M06-2X, Gas Phase)

Energy (Hartrees): = -1409.3826589
No imaginary frequencies

Standard orientation:

Center Number	Atomic Number	Atomic Type	Coordinates (Angstroms) X	Y	Z
1	6	0	1.079380	-2.316540	-0.926493
2	1	0	0.748182	-3.270786	-1.340224
3	6	0	-0.131400	-1.491618	-0.543011
4	1	0	-0.668140	-1.223393	-1.453774
5	6	0	0.232124	-0.236529	0.244852
6	1	0	0.707599	-0.526299	1.183440
7	6	0	-1.009861	0.583297	0.594326
8	1	0	-1.517789	0.079497	1.417108
9	6	0	-0.680133	2.033746	0.998603
10	1	0	0.238983	2.054880	1.593579
11	6	0	-1.788070	2.570753	1.813091
12	7	0	-2.673697	2.976573	2.419913
13	8	0	1.866483	-2.579508	0.235116
14	8	0	-0.992535	-2.245352	0.317685
15	8	0	1.169520	0.502267	-0.536441
16	8	0	-1.912740	0.655010	-0.515573
17	6	0	3.186542	-2.288989	0.175246
18	6	0	2.395227	0.737414	0.027836
19	6	0	-2.092406	-2.809962	-0.233251
20	6	0	-3.150709	0.096916	-0.368397
21	6	0	3.851611	-2.536199	1.494599
22	6	0	3.345874	1.354614	-0.947849
23	6	0	-3.924941	0.169354	-1.648011
24	1	0	4.025667	0.563328	-1.272273
25	1	0	2.822169	1.763946	-1.808671
26	1	0	3.905536	2.132733	-0.433843
27	1	0	3.420858	-3.404143	1.990054
28	1	0	4.920692	-2.658209	1.339892
29	1	0	3.675040	-1.651537	2.110853
30	1	0	-4.982825	0.048118	-1.430118
31	1	0	-3.736290	1.105779	-2.169753
32	1	0	-3.588665	-0.661154	-2.273833
33	6	0	-2.893742	-3.541755	0.798726
34	1	0	-3.432076	-2.793687	1.384546
35	1	0	-3.603500	-4.198619	0.302829

36	1	0	-2.237501	-4.099810	1.464709
37	7	0	-0.465306	2.882350	-0.149569
38	6	0	0.742077	3.478053	-0.389644
39	6	0	0.834290	4.180669	-1.724267
40	1	0	1.747379	4.769800	-1.746068
41	1	0	0.863896	3.434372	-2.522222
42	1	0	-0.029350	4.826356	-1.890209
43	8	0	3.722634	-1.858719	-0.809144
44	8	0	-2.373605	-2.695581	-1.395388
45	8	0	2.654778	0.427680	1.155514
46	8	0	-3.519227	-0.411846	0.649385
47	8	0	1.659402	3.424449	0.400575
48	1	0	1.676312	-1.786543	-1.667344
49	1	0	-1.156023	2.836365	-0.883059

Structure 42 $_5G^-$ (M06-2X, $CHCl_3$)

Energy (Hartrees): =-1409.4095537
No imaginary frequencies

Standard orientation:

Center Number	Atomic Number	Atomic Type	Coordinates (Angstroms) X	Y	Z
1	6	0	1.337384	-2.198527	-0.902042
2	1	0	1.115373	-3.188320	-1.306459
3	6	0	0.044452	-1.506772	-0.530025
4	1	0	-0.506704	-1.305338	-1.449077
5	6	0	0.259475	-0.209487	0.246409
6	1	0	0.767627	-0.424505	1.187952
7	6	0	-1.073288	0.457694	0.582517
8	1	0	-1.524185	-0.107540	1.398492
9	6	0	-0.926332	1.931719	1.003845
10	1	0	-0.049737	2.045073	1.649455
11	6	0	-2.120040	2.333473	1.775179
12	7	0	-3.064383	2.640779	2.350448
13	8	0	2.151374	-2.360327	0.262403
14	8	0	-0.740839	-2.342379	0.329322
15	8	0	1.092624	0.628696	-0.554366
16	8	0	-1.955918	0.426668	-0.543752
17	6	0	3.440973	-1.967854	0.189905
18	6	0	2.304384	0.988855	-0.043276
19	6	0	-1.786026	-3.006088	-0.216257
20	6	0	-3.145394	-0.225567	-0.409154
21	6	0	4.133055	-2.160248	1.502268
22	6	0	3.128057	1.722467	-1.051996
23	6	0	-3.911025	-0.196866	-1.691881
24	1	0	3.830520	1.000168	-1.477646
25	1	0	2.512867	2.138988	-1.847250
26	1	0	3.688389	2.506057	-0.545777
27	1	0	3.966565	-3.173073	1.870234
28	1	0	5.195853	-1.963601	1.384374
29	1	0	3.704874	-1.458238	2.220594
30	1	0	-4.877265	-0.673472	-1.546747
31	1	0	-4.040294	0.834792	-2.022793
32	1	0	-3.340934	-0.733493	-2.452534
33	6	0	-2.489642	-3.831990	0.812459
34	1	0	-2.878579	-3.169097	1.587250
35	1	0	-3.304386	-4.378299	0.343615
36	1	0	-1.782325	-4.522147	1.275000
37	7	0	-0.760806	2.819149	-0.120333
38	6	0	0.374145	3.555902	-0.299001
39	6	0	0.410700	4.336901	-1.587592
40	1	0	1.354915	4.872671	-1.652455
41	1	0	0.308474	3.660457	-2.439295
42	1	0	-0.417289	5.048674	-1.618202
43	8	0	3.937544	-1.515713	-0.807969
44	8	0	-2.086916	-2.911387	-1.376506
45	8	0	2.658737	0.692159	1.064221
46	8	0	-3.487761	-0.747955	0.614042
47	8	0	1.273884	3.565435	0.520112
48	1	0	1.868602	-1.616743	-1.654014
49	1	0	-1.443116	2.764237	-0.863717

Structure 42 R-X (M06-2X, Gas Phase)

Energy (Hartrees): = -1409.3803048
No imaginary frequencies

Standard orientation:

Center Number	Atomic Number	Atomic Type	Coordinates (Angstroms) X	Y	Z

1	6	0	-1.576754	1.218634	-0.922864
2	1	0	-1.749201	0.514540	-1.741909
3	6	0	-0.131391	1.101562	-0.420917
4	1	0	0.544635	1.511843	-1.172771
5	6	0	0.312411	-0.328183	-0.138240
6	1	0	0.251743	-0.889974	-1.072363
7	6	0	1.741219	-0.391541	0.386056
8	1	0	1.851791	0.216956	1.287528
9	6	0	2.158172	-1.813453	0.724007
10	1	0	3.236732	-1.846257	0.874161
11	1	0	1.643403	-2.153161	1.621162
12	6	0	0.937600	-3.659583	-0.161820
13	6	0	0.613861	-4.375063	-1.439026
14	1	0	-0.196216	-3.821628	-1.921517
15	1	0	0.265036	-5.378939	-1.209209
16	1	0	1.474417	-4.395864	-2.104368
17	6	0	3.687713	0.772381	-0.268256
18	6	0	4.330061	1.485749	-1.417232
19	1	0	4.284895	0.877087	-2.319349
20	1	0	5.357403	1.731326	-1.161064
21	1	0	3.762270	2.403059	-1.587126
22	6	0	-1.490582	-1.766849	0.401514
23	6	0	-2.279195	-2.327496	1.542406
24	1	0	-1.665854	-3.097030	2.016206
25	1	0	-3.194929	-2.771529	1.159738
26	1	0	-2.492290	-1.548807	2.274785
27	6	0	0.938078	2.830046	0.841909
28	6	0	0.788578	3.665277	2.077216
29	1	0	0.532632	3.039424	2.931073
30	1	0	-0.026263	4.374199	1.915344
31	1	0	1.713230	4.207209	2.255134
32	6	0	-3.815197	0.569009	-0.219279
33	6	0	-4.763243	0.366813	0.940495
34	1	0	-4.340148	0.655258	1.902885
35	1	0	-5.043039	-0.687352	0.971283
36	1	0	-5.666380	0.944977	0.747608
37	6	0	-1.778287	2.594564	-1.430398
38	7	0	-2.528158	0.915289	0.123120
39	1	0	-2.311541	1.251771	1.049507
40	7	0	-1.899004	3.682596	-1.775566
41	8	0	1.840186	-2.676913	-0.371590
42	8	0	0.441483	-3.890304	0.907598
43	8	0	2.543901	0.154168	-0.658636
44	8	0	4.083981	0.740582	0.861725
45	8	0	-0.506734	-0.945596	0.849636
46	8	0	-1.659511	-2.007668	-0.761091
47	8	0	-0.053664	1.900116	0.765883
48	8	0	1.791251	2.946285	0.011560
49	8	0	-4.140951	0.407641	-1.370466
